# Synthesis of Fused-Housane Derivatives via Intramolecular [2 + 2] Photocycloaddition

**DOI:** 10.1021/acs.orglett.5c00468

**Published:** 2025-04-09

**Authors:** David Suárez-García, Miguel A. Rodríguez, Iratxe Barbolla, Rubén Vicente

**Affiliations:** † Departamento de Química Orgánica e Inorgánica, Instituto Universitario de Química Organometálica“Enrique Moles”, 16763 Universidad de Oviedo, 33006 Oviedo, Spain; ‡ Centro de Innovación en Química Avanzada (ORFEO−CINQA), 16763 Universidad de Oviedo, 33006 Oviedo, Spain; § Departamento de Química, Instituto de Investigación en Química de la Universidad de La Rioja (IQUR), 16764 Universidad de la Rioja, 26006 Logroño, Spain

## Abstract

Herein, we describe a [2 + 2] photocycloaddition of cyclopropenes bearing a styryl group to prepare housane derivatives. The reaction occurs via selective excitation of the styrene fragment, enabling a completely stereoselective stepwise [2 + 2] cycloaddition. Fused-housane derivatives with an unprecedented substitution pattern are prepared in a simple manner using convenient visible light.

## Graphical abstract

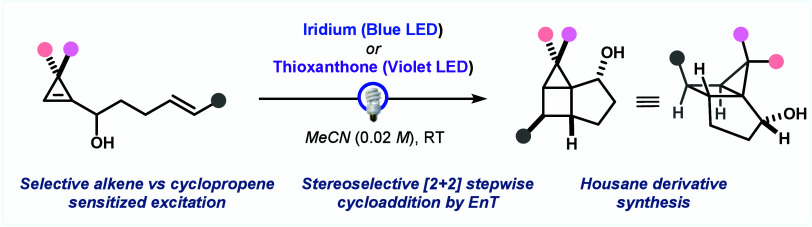

Strained hydrocarbons were considered exotic molecules,^1^ but they are gaining importance in medicinal chemistry,^2^ in the search for well-defined rigid 3D structures with a high Fsp^3^ parameter.^2a^ Unlike popular bicyclo[1.1.1]­pentanes,^3^ their fused constitutional isomers, namely, bicyclo[2.1.0]­pentanes or housanes, have received little attention. The lack of general methods to prepare housane derivatives could explain this situation. The synthesis of these compounds can be organized according to a preexisting ring in the starting material (Scheme 1). Methods using suitable functionalized 5-membered skeletons include base-promoted transannular cyclizations of cyclopentanes^4^ or N_2_ extrusion in diazabicycles.^5^ Another strategy involves the use of cyclobutenes as 4-membered starting material for a subsequent cyclopropanation with diazo compounds.^6^ Regrettably, these methods suffer from limitations regarding the scope or availability/stability of starting materials.

**Scheme 1:**
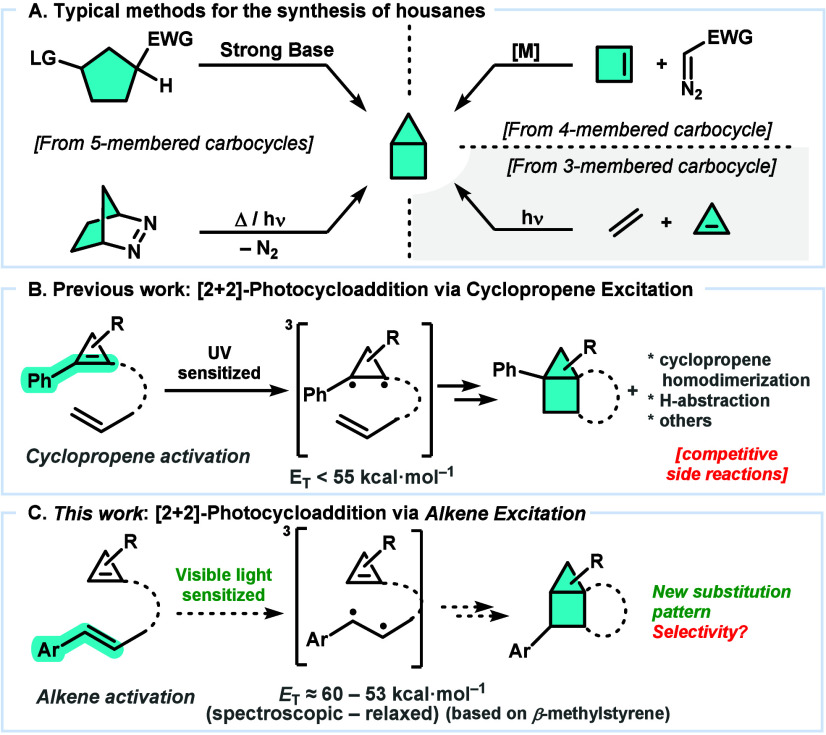
Synthesis of Housanes

Besides, a scarcely explored strategy comprises the use of cyclopropenes as a 3-membered ring to prepare housanes through a [2 + 2]-photocycloaddition with alkenes.^7^ This reactivity was previously described with emphasis on mechanistic studies and was limited to aryl-substituted cyclopropenes (Scheme 1B).^8^ The arene conjugation enabled access to a low energy ^3^π,π* excited state (<55 kcal·mol^–1^)^9^ by sensitized excitation under UV irradiation. The resulting triplet excited state can undergo a stepwise [2 + 2] cycloaddition with the alkene, but self-dimerization or H abstraction can be competitive.^9,10^

With these precedents in hand, we wondered if alternative [2 + 2]-photocycloadditions to prepare housanes could also be feasible. In particular, we focused on the [2 + 2]-cycloaddition of 1-cyclopropen-5-enes promoted with more convenient *visible light* and *through the sensitized excitation of the styrene fragment rather than the cyclopropene* (Scheme 1C).

Selective excitation should occur according to the substantial difference in triplet state energy (*E*_T_) of alkyl-substituted cyclopropenes^11^ and styrene derivatives.^12^ Interestingly, the successful realization of this approach would open access to housanes with a substitution pattern differing from those previously reported. It should be noted that visible-light-promoted [2 + 2]-cycloadditions of styrene derivatives with unbiased alkenes are well-documented,^13^ yet those involving highly strained alkenes are still challenging. Moreover, the cyclopropyl radical intermediates involved might undergo competitive H-abstraction or radical disproportionation reactions, compromising the desired reaction outcome.^14^

We started our study with cyclopropene **1a** as a candidate for the desired [2 + 2]-photocycloaddition using visible light (Scheme 2A). Based on Yoon’s work,^13b^ iridium complex **Ir-1** ([Ir­(dF­(CF_3_)­ppy)_2_(dtbbpy)]­[PF_6_]) was selected as a suitable sensitizer according to *E*_T_ values.^15^ Thus, irradiation of (*E*)-**1a** (0.02 M in MeCN, 24 h) with a standard 20 W compact fluorescent light bulb in the presence of **Ir-1** (1.0 mol %) afforded desired housane **2a** in a promising yield (20% NMR yield; *dr* = 6:1) along with *E*-to-Z isomerization of **1a** (57%; *Z:E* = 4:1).^16^ Importantly, the [2 + 2]-photocycloaddition took place with complete stereoselectivity since the isomers arise from configurations at the carbinol moiety (starred atom). This selectivity is remarkable since [2 + 2]-photocycloadditions usually provide mixtures of diasetereoisomers.^13^ Gratifyingly, the use of blue LED light (451 nm) led to housane **2a** as a single product in a synthetically useful yield (78% NMR; *dr* = 5:1, 72% isolated) after 2 h irradiation.^17^ Modifications of the concentration (MeCN) or solvent led to inferior results. Moreover, iridium complexes (**Ir-2,4**) with slightly lower *E*_T_ values^15^ were less efficient to afford housane **2a**, and noticeable *E*/*Z*-isomerization of **1a** was eventually observed. In contrast, the use of ruthenium complex **Ru-1** led to the recovery of unaltered starting cyclopropene **1a**. Representative organic sensitizers were also evaluated, yet housane **2a** was only detected when using 4CzlPN (19%, *dr* = 3:1). Remarkably, using inexpensive thioxanthone (**TXT**) as a sensitizer under irradiation with deep violet LED (390–410 nm) housane **2a** was obtained with comparable results (65% NMR yield, *dr* = 4:1, 60% isolated), although the reaction was markedly less clean, hampering purification. Finally, control experiments indicated that the formation of **2a** does not take place in the absence of a sensitizer after extensive irradiation or in the dark.

**Scheme 2:**
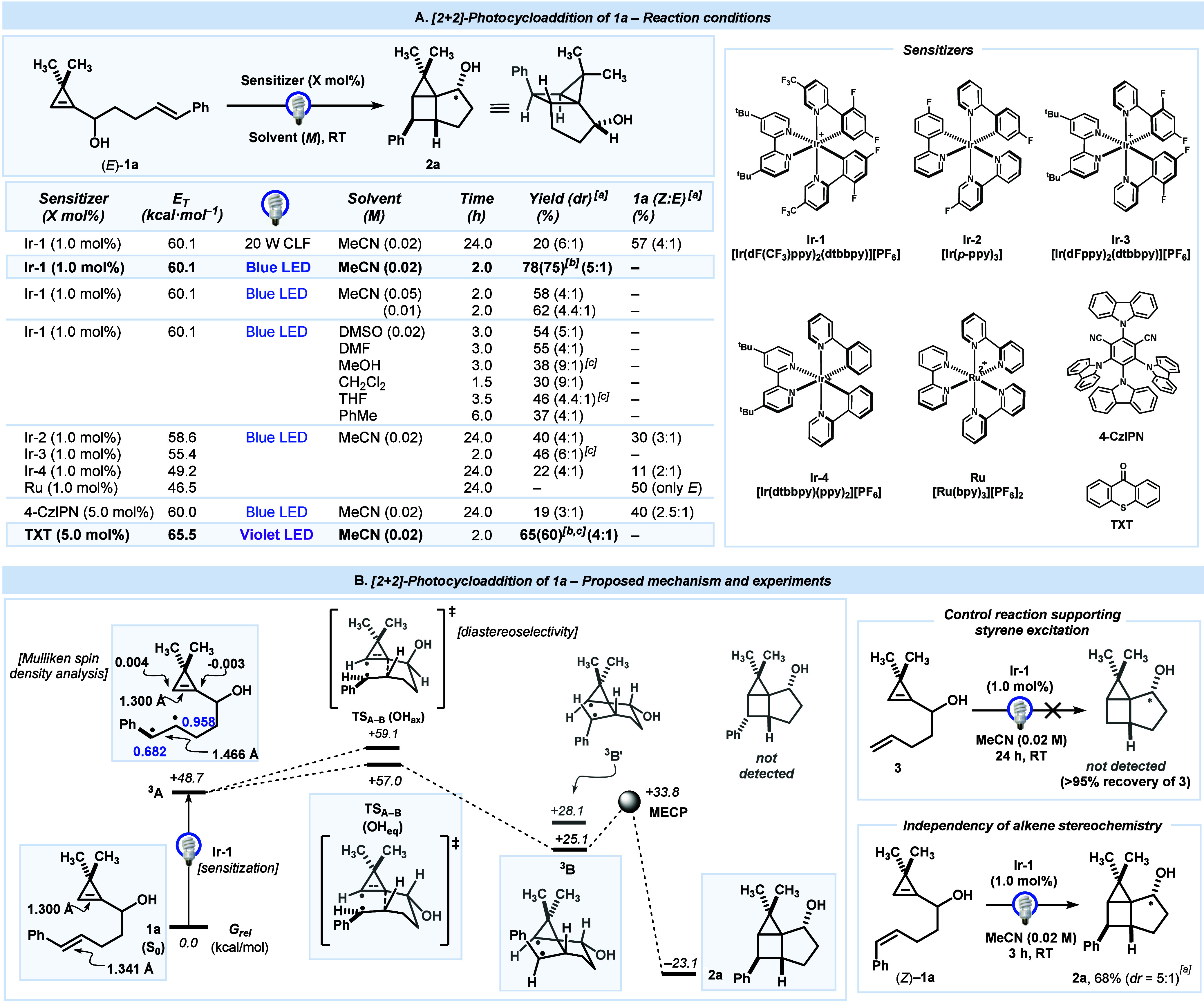
[2 + 2]-Photocycloaddition of Cyclopropene **1a** to housane **2a**: Screening Summary (A) and Proposed Mechanism (B)

a Determined by ^1^H NMR of the crude reaction performed at 0.15–0.2 mmol scale.
b Isolated yield.
c Complex mixtures of side products were observed (starred C atom denotes the difference with the minor diastereoisomer).

The proposed mechanism for the formation of **2a** depicted in Scheme 2B is based on reported [2 + 2]-photocycloadditions,^13,18^ as well as on our experimental and computational study. Cyclic voltammetry indicates that a SET process seems unlikely since the excited triplet state oxidation potential of **Ir-1** (*E*(PC*/PC^•–^ = +0.89 V vs SCE) or **TXT** (*E*(PC*/PC^•–^ = +1.18 V vs SCE) is insufficient to oxidize **1a** (*E*(**1a** ^**+**^/**1a** = +1.80 V vs Ag/AgCl).^19^ Besides, considering the *E*_T_ values of the sensitizers evaluated and the calculated *E*_T_ (relaxed) for **1a** (+48.7 kcal/mol),^20^ the process is compatible with a sensitization mechanism to generate excited triplet species ^**3**^ **A**. According to our calculations, optimized structures **1a** and ^**3**^ **A** show virtually the same C–C bond distance (1.300 Å) for the cyclopropene double bond.^21^ In contrast, structure ^**3**^ **A** shows a significant lengthening of the C–C bond (1.466 Å vs 1.341 Å in **1a**) in the styrene fragment. Moreover, calculated spin densities in ^**3**^ **A** indicated a substantial increase in the styrene fragment (0.958 and 0.682 au). These results are in agreement with the styrene fragment being responsible for the excitation. This fact is experimentally supported by the lack of reactivity of cyclopropene **3**, bearing a terminal alkene instead of a styrene. Then, a stepwise [2 + 2]-cycloaddition takes place starting with a favored 5-*exo*-cyclization via **TS**_**A‑B**_(OH_eq_) (+57.0 kcal/mol) to generate intermediate ^**3**^ **B** (+25.1 kcal/mol), in accordance with the intrinsic reaction coordinate (IRC) calculation. The subsequent cyclobutane ring closure takes place via ICS at MECP (+33.8 kcal/mol), which enables the 1,4-biradical coupling to afford housane **2a** (−23.1 kcal/mol). The negligible influence on the reaction outcome observed with (*Z*)-**1a** is compatible with the generation of the photostationary state equilibrium of *E*/*Z*-isomers and a typical irreversible stepwise photopromoted [2 + 2]-cycloaddition.^13^ Further calculations were performed to explain the stereoselectivity observed in the reaction. First, we considered structure ^**3**^ **B′**, a conformer of proposed ^**3**^ **B**, which potentially leads to a different cyclobutane diastereoisomer. However, ^**3**^ **B′** (+28.1 kcal/mol) is less stable since the phenyl group is located in a pseudoaxial position. Moreover, the corresponding transition state for the formation of ^**3**^ **B′** places the phenyl group in the vicinity of the cyclopropane ring, resulting in a TS structure with higher energy (+61 kcal/mol, see the SI for additional details). With respect to the observed selectivity at the carbinol moiety, we found a slightly higher barrier for calculated **TS**_**A‑B**_(OH_ax_) (+59.1 kcal/mol) due to the presence of the OH group at pseudoaxial position. This result is compatible with the diastereoselectivity observed.

With suitable conditions for the synthesis of housanes, we next explored the scope of the reaction using both **Ir-1** (blue LED) and **TXT** (violet LED) as appropriate sensitizers (Scheme 3). First, housanes **2a−f** showing para-substituted arenes with different bias were prepared in reasonable yields (65−80%) and similar diastereoselectivities. The results were similar when comparing both reaction conditions, yet, as stated before, the use of **Ir-1** (blue LED) resulted in cleaner reactions. Notably, housane **2d** could not be obtained when using TXT (violet LED) due to the complete degradation of the starting cyclopropene. Moreover, the preparation of **2a** was accomplished at a larger scale (1.0 mmol), pointing out the viability for a further scale-up of the process.

**Scheme 3:**
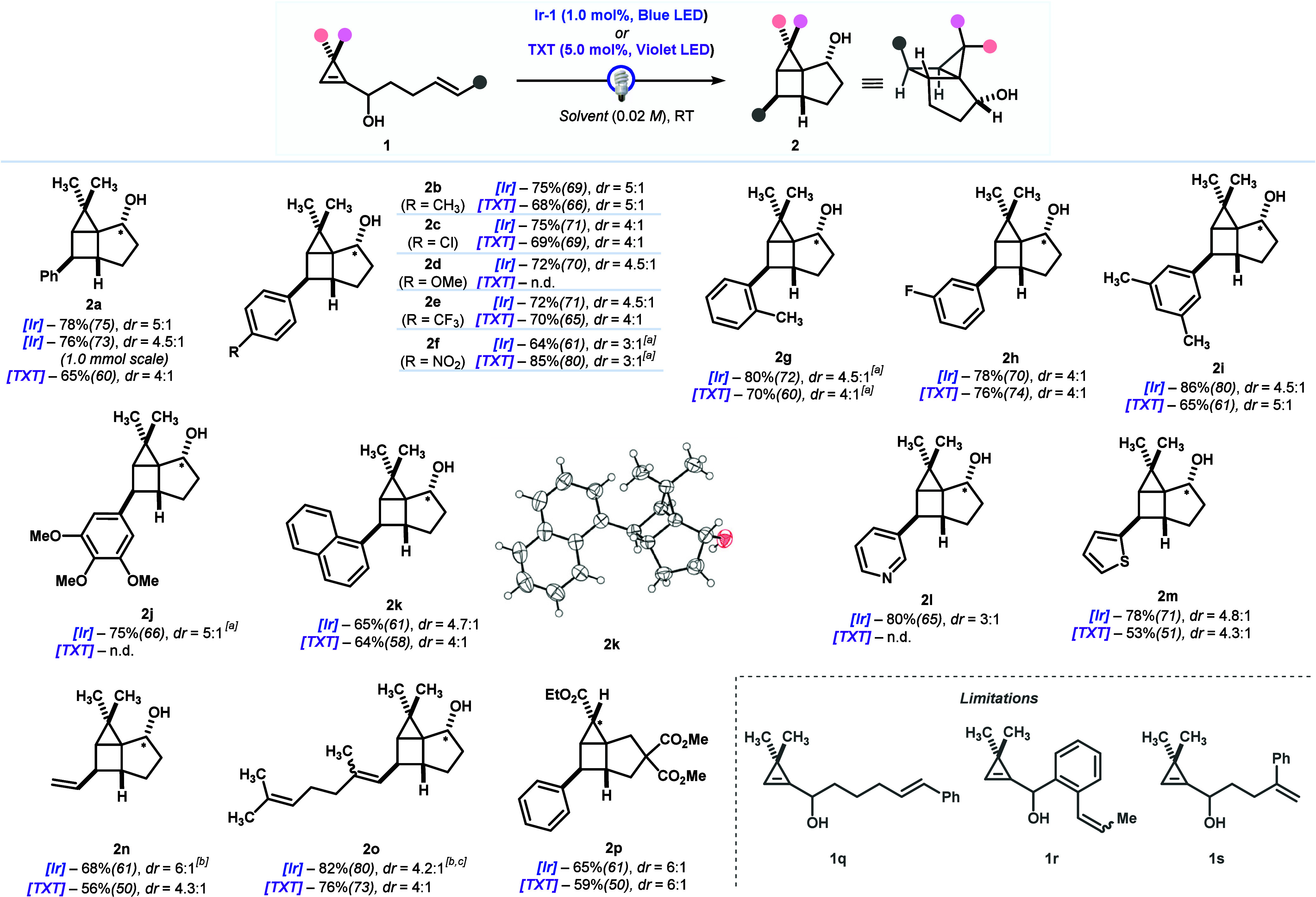
Synthesis of Housanes **2** via [2 + 2]-Photocycloaddition of Cyclopropenes **1**: Scope^a^

a From *Z*-alkene.
b Unknown side products were detected.
c Alkene isomerization observed (ellipsoid contours of **2k** at 50% probability level).
d Yields and *dr* determined by ^1^H NMR of the crude reaction performed at 0.15–0.2 mmol scale. Isolated yields in parentheses (n.d. = not detected).

Likewise, housanes **2g** (80%, blue LED) and **2h** (78%, blue LED) bearing arenes with *meta*- and *ortho*-substituents were also prepared in an efficient manner using both types of irradiations. Multisubstituted arenes such as 3,5-xylyl- or 3,4,5-trimethoxyphenyl led to the formation of housanes **2i** (86%, blue LED) and **2j** (75%, blue LED), respectively. Unsurprisingly, housane **2j** was only available by means of blue LED irradiation with an **Ir-1** sensitizer. Housane **2k** bearing a 1-naphthyl substituent was prepared in good yield (65%, *dr* = 4.7:1, blue LED). Importantly, X-ray analysis of **2k** served to endorse unambiguously the structure of the housanes. The feasibility of using electron-rich as well as electron-deficient heteroarenes was demonstrated with the preparation of 3-pyridyl- and 2-thiophenyl housane derivatives **2l** (80%, *dr* = 3:1, blue LED only) and **2m** (78% *dr* = 4.8:1, blue LED), respectively. Since conjugated dienes hold similar *E*_T_ values as styrenes, we studied the reaction with cyclopropenes where the styrene was replaced by a 1,3-butadienyl group. Gratifyingly, we were able to obtain the corresponding vinyl-substituted housane derivative **2n** in decent yield (67%, *dr* = 6:1). In a similar manner, a cyclopropene decorated with a polyene derived from geraniol was converted into housane **2o** in good yield (82%, blue LED), although a concomitant alkene isomerization was observed as well. We also demonstrated that substitution on the cyclopropane ring of the housane was also feasible, as shown with the preparation of housane **2p**, which was obtained in similar efficiency (65%, *dr* = 6:1) as observed in previous examples. At this stage, we have found also limitations to this process. Thus, with cyclopropenes **1q** and **1r** bearing a modified tethering (longer and inserted arene, respectively) only alkene isomerization was observed, while cyclopropene **1s** with a 1,1-disubstituted styrene fragment was unreactive.

Finally, we preliminarily explored derivatizations of housanes **2** (Scheme 4A). Thus, a typical Steglich coupling of **2a** with 6-chloronicotinic acid (**4**) led to the formation of ester **5** (79%). Also, Swern oxidation of housane **2m** enabled the preparation of the corresponding ketone **6**, albeit in a modest yield (30%). Currently, other handlings of the OH group such as Appel or Mitsunobu reactions, performed under standard conditions, have regrettably failed, leading to complex reaction mixtures.

**Scheme 4:**
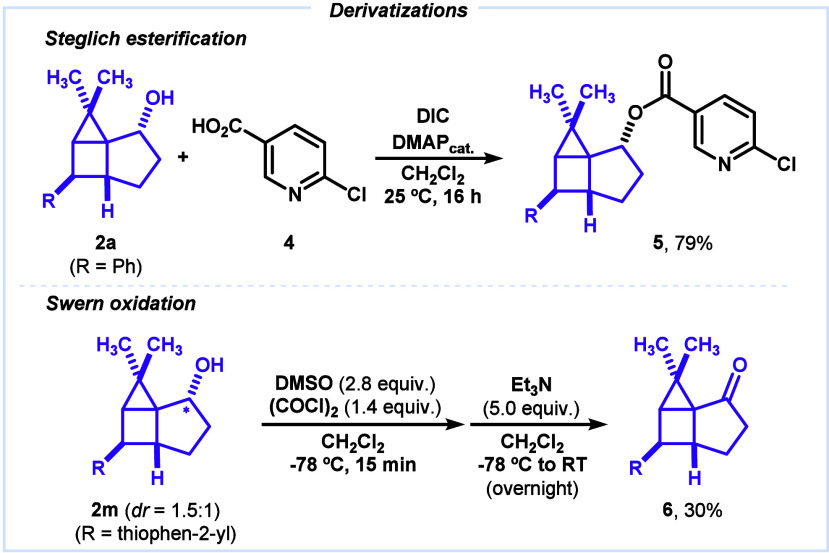


In summary, we have described herein the intramolecular [2 + 2]-photocycloaddition of 1,5-cyclopropenes for the efficient preparation of housane derivatives with a substantial scope. Two important features of this reaction deserve recognition. First, the stepwise [2 + 2]-photocycloaddition takes place with complete diastereoselectivity, in contrast to the typically observed mixture of diastereoisomers in reported similar [2 + 2]-1,*n*-diene photocycloadditions. Moreover, in the case of cyclopropenes, while previous related works relied on the photochemical activation of the cyclopropene, this method is based on alkene activation. This fundamental mechanistic difference was experimentally validated. Besides, considering the increasing relevance of strained carbocycles with high Fsp^3^ fraction in medicinal chemistry, this work could find utility since it enables a facile preparation of structurally new compounds which might be interesting as potential bioisosters, among others. The extension of the scope and the feasibility to accomplish the reaction in an intermolecular fashion are also under investigation in our laboratories.

## Supplementary Material



## Data Availability

The data underlying this study are available in the published article and its Supporting Information.

## Notes

Experimental procedures, characterization data, computational details, and copies of NMR spectra (PDF)
