# Supplementary material for: Synthesis of Fused-Housane Derivatives via Intramolecular [2 + 2] Photocycloaddition
Source: Org Lett. 2025 Apr 9;27(15):3825–30. doi: 10.1021/acs.orglett.5c00468 (PMC12426990; doi:10.1021/acs.orglett.5c00468)

Supporting Information for

Synthesis of Fused-Housane Derivatives via Intramolecular [2+2] Photocycloaddition

David Suárez-García, Miguel A. Rodríguez, Iratxe Barbolla, and Rubén Vicente*

TABLE OF CONTENTS

1. General Remarks. S-3

2. Synthesis of cyclopropenes **1** and cyclopropene **3**. S-4

2.1. Representative procedures. S-4

2.2. Characterization data for cyclopropenes **1** and cyclopropene **3**. S-6

3. Screening for the [2+2] photocycloaddition of cyclopropene **1a**. S-19

4. Synthesis of housanes **2** via [2+2] photocycloaddition. S-22

4.1. Representative procedures. S-22

4.2. Characterization data for housanes **2**. S-23

5. Proposed mechanism. S-41

5.1. Mechanistic experiments. S-41

5.2. Computational studies. S-43

6. Synthesis of housane **2a** at 1.0 mmol scale. S-56

7. Derivatization of housanes **2**: Steglich reaction and Swern oxidation. S-57

8. Crystallographic data for compound **2k**. S-59

9. Synthesis and characterization of starting aldehydes **S2**. S-60

10. ^{1}H-, ^{13}C-NMR and selected 2D-NMR spectra for new compounds S-64

1. General Remarks

All reactions were carried out under Argon atmosphere using standard Schlenck techniques. All solvents were distilled according to reported procedures[1] under N_2 atmosphere and were degassed by bubbling Ar during 10 minutes. Solvents for column chromatography were obtained from commercial supplier and used without further purification. TLC was performed on aluminium-backed plates coated with silica gel 60 with F_{254} indicator. Flash column chromatography was carried out on silica gel (60-120 mesh) or deactivated silica gel (60-120 mesh, see below the deactivation procedure). ^{1}H NMR (300, 400 MHz) and ^{13}C NMR (75.5 and 100 MHz) spectra were recorded at room temperature in the indicated solvent on a Bruker DPX-300, or Bruker AVANCE-300 MHz and 400 MHz instruments. Chemical shifts (δ) are given in ppm relative to TMS (^{1}H, 0.0 ppm) or $CDCl_3$ (^{13}C, 77.0 ppm). Carbon multiplicities were assigned by DEPT experiments. Structural assignments were made with additional information from gCOSY, gHSQC, gNOESY and gHMBC experiments using a Bruker AVANCE-400 MHz.

High resolution mass spectra (HRMS) were determined by the University of Oviedo with IMPACT II (BRUKER) or 6545 Q-TOF (AGILENT) mass spectrometers.

Commercially available catalysts and reagents were used as received and stored under N_2 atmosphere.

[1] Armarego, W. L. F.. *Purification of Laboratory Chemicals.* 3rd ed. Oxford ; New York, Pergamon Press, **1988**.

2. Synthesis of cyclopropenes 1

2.1. Representative procedures.

Representative procedure A.

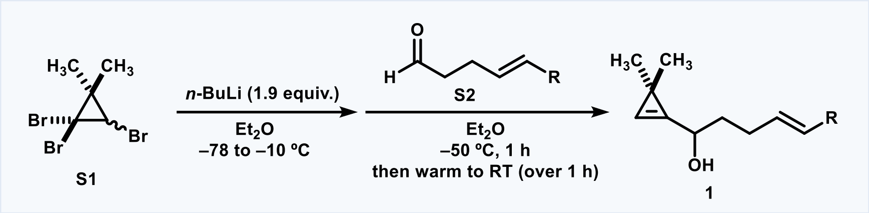

Following the procedure described by Cossy,[2,3] to a solution of 1,1,3-tribromo-2,2-dimethylcyclopropane[2] (**S1**, 1.0-5.0 mmol, 1.0 equiv.) in Et_2O (ca. 0.33 M) at –78 °C, was added dropwise *n*-BuLi (1.6 M in hexanes, 1.9-9.5 mmol, 1.9 equiv.). The resulting mixture was warmed to –10 °C, stirred for 1 h at this temperature and cooled to –50 °C. A solution of the the corresponding aldehyde[4] (**S2**, 0.8-1.0 mmol, 0.8-1.0 equiv. in ca. 5 mL of Et_2O) was added and the reaction mixture was allowed to warm to ambient temperature. After 1 h, NH_4Cl sat. (10 mL) and Et_2O (10 mL) were added, the layers were separated and the aqueous phase was extracted with Et_2O (3 x 10 mL). The combined organic extracts were washed with brine, dried over Na_2SO_4, filtered and concentrated under reduced pressure. The residue was purified by flash chromatography (SiO_2, hexanes/EtOAc) to afford the corresponding cyclopropenes **1**.

Representative procedure B*: Synthesis of cyclopropene* ***1p****.*

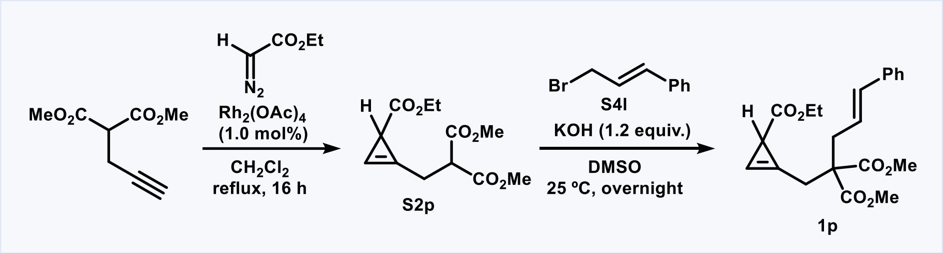

Following a reported procedure,[5] to a refluxing mixture of dimethyl 2-(prop-2-yn-1-yl)malonate (1.71 g, 10 mmol) and rhodium (II) acetate dimer (22.1 mg, 0.05 mmol) in dry DCM (20 mL), a

---

[2] (a) Miege, F.; Meyer, C.; Cossy, J. Synthesis of 3-Oxa- and 3-Azabicyclo[4.1.0]Heptanes by Gold-Catalyzed Cycloisomerization of Cyclopropenes. *Org. Lett.* **2010**, *12*, 4144–4147. (b) Miege, F.; Meyer, C.; Cossy, J. Gold(I)-Catalysed Cycloisomerisation of 1,6-Cyclopropene-Enes. *Chem. Eur. J.* **2012**, *18*, 7810–7822.

[3] Ernouf, G.; Brayer, J. L.; Folléas, B.; Demoute, J. P.; Meyer, C.; Cossy, J. Synthesis of Functionalized Alkylidenecyclopropanes by Ireland-Claisen Rearrangement of Cyclopropenylcarbinyl Esters. *Org. Lett.* **2015**, *17*, 3786–3789.

[4] For the synthesis of new aldehydes, see Section 8.

[5] Zhu, P. L.; Zhang, Z.; Tang, X. Y.; Marek, I.; Shi, M. Gold- and Silver-Catalyzed Intramolecular Cyclizations of Indolylcyclopropenes for the Divergent Synthesis of Azepinoindoles and Spiroindoline Piperidines. *ChemCatChem* **2015**, *7*, 595–600.

solution of ethyl diazoacetate (1.33 mL of a 80% solution in CH_2Cl_2 and 17 mL of dry DCM) was added over 16 hours while the mixture is under constant reflux. After the addition, the mixture was stirred for an additional 1 h. The mixture was filtered through a short pad of celite and the solvent was removed under reduced pressure. The residue was purified by flash chromatography (SiO_2, hexanes: EtOAc = 5:1) to afford **S2p** (754 mg, 30%) as a pale yellow oil. Following a reported procedure,[6] Freshly powdered KOH (194 mg, 3.46 mmol, 1.2 equiv) was dissolved in dry DMSO (14.0 mL), and the resulting suspension was stirred under argon atmosphere at room temperature for 5 min. Then, a solution of dimethyl 2-((3-(ethoxycarbonyl)cycloprop-1-en-1-yl)methyl)malonate (724 mg, 2.83 mmol, 1.0 equiv) in dry DMSO (14.0 mL) was added to the suspension. Immediately after, (*E*)-(3-bromoprop-1-en-1-yl)benzene (0.42 mL, 2.83 mmol, 1.0 equiv) was added dropwise, and the reaction was stirred overnight. NH_4Cl (saturated, 10 mL) was added to the crude mixture and the organic phase was extracted with Et_2O (3 x 20 mL). The organic layers were collected and washed with $NaHCO_3$ (saturated, 10 mL). The organic phases were collected and washed with brine, dried over Na_2CO_3. After filtration, the solvent was removed under vacuum. Purification by flash chromatography (SiO_2, hexanes: EtOAc = 30:1) afforded **1p** (403 mg, 38%) as a pale yellow oil.

[6] Martínez, Á. M.; Puet, A.; Domínguez, G.; Alonso, I.; Castro-Biondo, R.; Pérez-Castells, J. Intramolecular Diels-Alder Reaction of Cyclopropenyl Vinylarenes: Access to Benzonorcarane Derivatives. *Org. Lett.* **2023**, *25*, 5923–5928.

2.2. Characterization data for cyclopropenes 1.

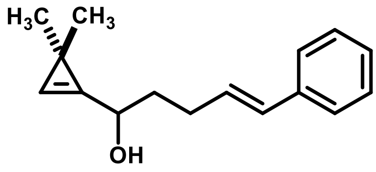

(*E*)-**1a**

(*E*)-1-(3,3-Dimethylcycloprop-1-en-1-yl)-5-phenylpent-4-en-1-ol ((*E*)-**1a**): The representative procedure A was followed using **S1** (1.54 g, 4.98 mmol), butyllithium [1.6 M in Hexanes] (6.2 mL, 9.96 mmol) and (*E*)-5-phenyl-4-pentenal[7] (639 mg, 3.98 mmol). Purification by flash chromatography (SiO_2, hexanes:EtOAc = 10:1) afforded (*E*)-**1a** (528.8 mg, 58 %) as a colorless oil.

^{1}H-NMR (300 MHz, $CDCl_3$) δ = 7.43 – 7.25 (m, 4H), 7.29 – 7.17 (m, 1H), 7.09 (s, 1H), 6.47 (d, *J* = 15.8 Hz, 1H), 6.28 (dt, *J* = 15.8, 6.9 Hz, 1H), 4.76 (t, *J* = 6.5 Hz, 1H), 2.49 – 2.33 (m, 2H), 1.95 – 1.75 (m, 3H), 1.25 (s, 3H), 1.23 (s, 3H).

^{13}C-NMR (75 MHz, $CDCl_3$) δ = 137.6, 136.7, 130.6, 129.9, 128.5, 127.0, 126.0, 114.5, 68.5, 35.6, 28.8, 27.9, 27.6, 21.0.

HRMS (ESI) m/z: $[M + H]^+$ Calcd for $[C_{16}H_{21}O]^+$ 229.1587; found 229.1584.

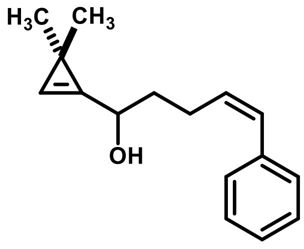

(Z)-**1a**

(*Z*)-1-(3,3-Dimethylcycloprop-1-en-1-yl)-5-phenylpent-4-en-1-ol ((*Z*)-**1a**): The representative procedure A was followed using **S1** (309 mg, 1.00 mmol), butyllithium [1.6 M in Hexanes] (1.3 mL, 9.96 mmol) and (*Z*)-5-phenyl-4-pentenal[8] (129 mg, 0.80 mmol). Purification by flash chromatography (SiO_2, hexanes:EtOAc = 5:1) afforded (*Z*)-**1a** (147 mg, 80 %) as a colorless oil.

^{1}H-NMR (300 MHz, $CDCl_3$) δ = 7.42 – 7.19 (m, 5H), 7.01 (s, 1H), 6.50 (d, *J* = 11.6 Hz, 1H), 5.72 (dt, *J* = 11.6, 7.3 Hz, 1H), 4.72 (td, *J* = 6.5, 1.4 Hz, 1H), 2.54 (qd, *J* = 7.5, 1.8 Hz, 2H), 1.97 – 1.77 (m, 3H), 1.18 (s, 6H).

[7] Musacchio, A. J.; Nguyen, L. Q.; Beard, G. H.; Knowles, R. R. Catalytic Olefin Hydroamination with Aminium Radical Cations: A Photoredox Method for Direct C-N Bond Formation. *J. Am. Chem. Soc.* **2014**, *136*, 12217–12220.

[8] Line, N. J.; Witherspoon, B. P.; Hancock, E. N.; Brown, M. K. Synthesis of Ent-[3]-Ladderanol: Development and Application of Intramolecular Chirality Transfer [2+2] Cycloadditions of Allenic Ketones and Alkenes. *J. Am. Chem. Soc.* **2017**, *139*, 14392–14395.

^{13}C-NMR (75 MHz, $CDCl_3$) δ = 137.8, 137.0, 132.3, 130.0, 129.2, 128.6, 114.9, 69.0, 36.6, 28.3, 27.9, 24.9, 21.4.

HRMS (ESI) m/z: $[M + H]^+$ Calcd for $[C_{16}H_{21}O]^+$ 229.1587; found 229.1580.

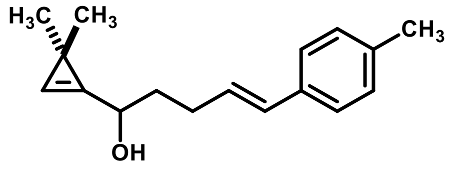

1b

(*E*)-1-(3,3-Dimethylcycloprop-1-en-1-yl)-5-(*p*-tolyl)pent-4-en-1-ol (**1b**): The representative procedure A was followed using **S1** (327.3 mg, 1.07 mmol), butyllithium [1.6 M in Hexanes] (1.3 mL, 2.13 mmol) and 5-(*p*-tolyl)-4-pentenal[9] (148.7 mg, 0.85 mmol, *E/Z* = 15:1). Purification by flash chromatography (SiO_2, hexanes:EtOAc = 10:1) afforded **1b** (97 mg, 47 %) as a colorless oil.

^{1}H-NMR (300 MHz, $CDCl_3$) δ = 7.30 (d, *J* = 8.2 Hz, 2H), 7.15 (d, *J* = 8.0 Hz, 2H), 7.10 (s, 1H), 6.46 (d, *J* = 15.7 Hz, 1H), 6.24 (dt, *J* = 15.8, 6.9 Hz, 1H), 4.77 (t, *J* = 6.5 Hz, 1H), 2.47 – 2.39 (m, 2H), 2.37 (s, 3H), 2.12 (bs, 1H), 1.95 – 1.81 (m, 2H), 1.27 (s, 3H), 1.26 (s, 3H). *[Small signals correspond to Z-isomer].*

^{13}C-NMR (75 MHz, $CDCl_3$) δ = 136.8, 136.7, 134.8, 130.4, 129.3, 128.8, 125.9, 114.4, 68.5, 35.7, 28.9, 28.0, 27.7, 21.2, 21.0. *[Small signals correspond to Z-isomer].*

HRMS (ESI) m/z: $[M+H]^+$ Calcd for $[C_{17}H_{23}O]^+$ 243.1743; found 243.1740.

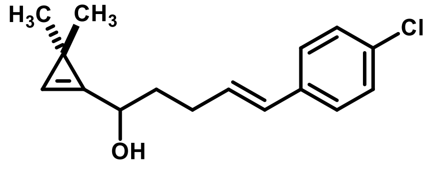

1c

(*E*)-5-(4-Chlorophenyl)-1-(3,3-dimethylcycloprop-1-en-1-yl)pent-4-en-1-ol (**1c**): The representative procedure A was followed using **S1** (307 mg, 1.00 mmol), butyllithium [1.6 M in Hexanes] (1.25 mL, 2.00 mmol) and (*E*)-5-(4-chlorophenyl)-4-pentenal[9] (156 mg, 0.8 mmol). Purification by flash chromatography (SiO_2, hexanes:EtOAc = 10:1) afforded **1c** (142 mg, 68%) as a colorless oil.

[9] Džambaski, Z.; Tzaras, D. I.; Lee, S.; Kokotos, C. G.; Bondzic, B. P. Enantioselective Organocatalytic Enamine C–H Oxidation/Diels- Alder Reaction. *Adv. Synth. Catal.* **2019**, *361*, 1792–1797.

^{1}H-NMR (300 MHz, $CDCl_3$) δ = 7.28 (s, 4H), 7.08 (s, 1H), 6.41 (d, *J* = 15.8 Hz, 1H), 6.24 (dt, *J* = 15.8, 6.8 Hz, 1H), 4.74 (t, *J* = 6.4 Hz, 1H), 2.40 (qd, *J* = 7.7, 1.3 Hz, 2H), 1.98 – 1.74 (m, 3H), 1.24 (s, 3H), 1.23 (s, 3H).

^{13}C-NMR (75 MHz, $CDCl_3$) δ = 136.7, 136.1, 132.5, 130.6, 129.4, 128.6, 127.2, 114.6, 68.4, 35.5, 28.8, 27.9, 27.6, 21.0.

HRMS (ESI) m/z: $[M+H]^+$ Calcd for $[C_{16}H_{20}ClO]^+$ 263.1197; found 263.1191.

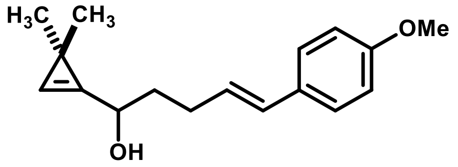

1d

(*E*)-1-(3,3-Dimethylcycloprop-1-en-1-yl)-5-(4-methoxyphenyl)pent-4-en-1-ol (**1d**): The representative procedure A was followed using **S1** (312 mg, 1.02 mmol), butyllithium [1.6 M in Hexanes] (1.30 mL, 2.00 mmol) and (*E*)-5-(4-methoxyphenyl)-4-pentenal[10] (155 mg, 0.81 mmol). Purification by flash chromatography (SiO_2, hexanes:EtOAc = 10:1) afforded **1d** (90 mg, 43%) as a colorless oil.

^{1}H-NMR (300 MHz, $CDCl_3$) δ = 7.31 (d, *J* = 8.7 Hz, 2H), 7.07 (s, 1H), 6.86 (d, *J* = 8.7 Hz, 2H), 6.41 (d, *J* = 16.0 Hz, 1H), 6.13 (dt, *J* = 15.8, 7.0 Hz, 1H), 4.75 (t, *J* = 6.4 Hz, 1H), 3.82 (s, 3H), 2.39 (qd, *J* = 7.5, 1.4 Hz, 2H), 1.93 – 1.75 (m, 3H), 1.24 (s, 3H), 1.23 (s, 3H).

^{13}C-NMR (75 MHz, $CDCl_3$) δ = 158.8, 136.8, 130.4, 129.9, 127.6, 127.1, 114.4, 113.9, 68.5, 55.3, 35.8, 28.8, 27.9, 27.6, 21.0.

HRMS (ESI) m/z: $[M+H]^+$ Calcd for $[C_{17}H_{23}O_2]^+$ 259.1693; found 259.1692.

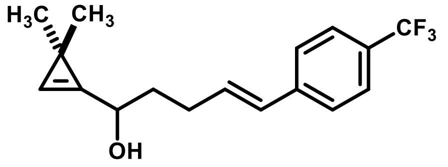

1e

(*E*)-1-(3,3-Dimethylcycloprop-1-en-1-yl)-5-(4-(trifluoromethyl)phenyl)pent-4-en-1-ol (**1e**): The representative procedure A was followed using **S1** (225 mg, 0.73 mmol), butyllithium [1.6 M in

[10] Hayashi, Y.; Itoh, T.; Ishikawa, H. Oxidative and Enantioselective Cross-Coupling of Aldehydes and Nitromethane Catalyzed by Diphenylprolinol Silyl Ether. *Angew. Chem. Int. Ed.* **2011**, *50*, 3920–3924.

Hexanes] (0.95 mL, 1.48 mmol) and (*E*)-5-[4-(trifluoromethyl)phenyl]-4-pentenal[11] (135 mg, 0.59 mmol). Purification by flash chromatography (SiO_2, hexanes:EtOAc = 5:1) afforded **1e** (90 mg, 43%) as a colorless oil.

^{1}H-NMR (300 MHz, $CDCl_3$) δ = 7.54 (d, *J* = 8.2 Hz, 2H), 7.43 (d, *J* = 7.9 Hz, 2H), 7.07 (s, 1H), 6.47 (d, *J* = 15.9 Hz, 1H), 6.36 (dt, *J* = 15.9, 6.7 Hz, 1H), 4.74 (ddd, *J* = 7.1, 5.6, 1.4 Hz, 1H), 2.75 – 2.62 (m, 2H), 1.95 – 1.77 (m, 2H), 1.75 (bs, 1H), 1.23 (s, 3H), 1.21 (s, 3H).

^{13}C-NMR (75 MHz, $CDCl_3$) δ = 141.2, 136.8, 132.9, 129.5, 128.98 (d, *J* = 32.2 Hz), 126.3, 125.60 (q, *J* = 3.8 Hz), 124.42 (d, *J* = 271.7 Hz), 114.8, 68.5, 35.5, 29.0, 28.0, 27.7, 21.2.

^{19}F-NMR (282 MHz, $CDCl_3$) δ = – 63.7 (s).

HRMS (ESI) m/z: $[M+H]^+$ Calcd for $[C_{17}H_{20}F_3O]^+$ 297.1461; found 297.1458.

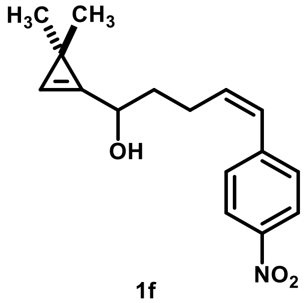

1f

(*Z*)-1-(3,3-Dimethylcycloprop-1-en-1-yl)-5-(4-nitrophenyl)pent-4-en-1-ol (**1f**): The representative procedure A was followed using **S1** (137 mg, 0.44 mmol), butyllithium [1.6 M in Hexanes] (0.70 mL, 1.1 mmol) and (*Z*)-5-(4-nitrophenyl)-4-pentenal[12] (90 mg, 0.43 mmol). Purification by flash chromatography (SiO_2, hexanes:EtOAc = 5:1) afforded **1f** (72.3 mg, 56%) as a colorless oil.

^{1}H-NMR (300 MHz, $CDCl_3$) δ = 8.18 (d, *J* = 8.5 Hz, 2H), 7.44 (d, *J* = 8.6 Hz, 2H), 7.02 (s, 1H), 6.51 (d, *J* = 11.8 Hz, 1H), 5.90 (dt, *J* = 11.7, 7.4 Hz, 1H), 4.71 (t, *J* = 6.4 Hz, 1H), 2.52 (qd, *J* = 7.5, 1.8 Hz, 2H), 2.00 (s, 1H), 1.90 – 1.79 (m, 2H), 1.15 (bs, 6H).

^{13}C-NMR (75 MHz, $CDCl_3$) δ = 146.2, 144.2, 136.4, 135.8, 129.4, 127.7, 123.5, 114.8, 68.3, 35.8, 27.8, 27.5, 24.6, 21.0.

HRMS (ESI) m/z: $[M+H]^+$ Calcd for $[C_{16}H_{20}NO_3]^+$ 274.1438; found 274.1437.

[11] Oh, H.; Ryou, B.; Park, J.; Kim, M.; Choi, J. H.; Park, C. M. Synthesis of Bicyclic N-Heterocycles via Photoredox Cycloaddition of Imino-Alkynes and Imino-Alkenes. *ACS Catal.* **2021**, *11*, 13670–13679.

[12] For the synthesis of the corresponding aldehyde, see Section 8.

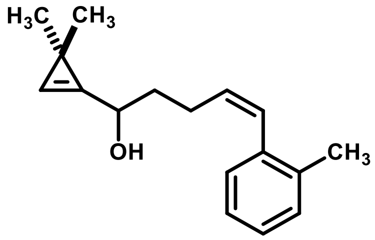

1g

(*Z*)-1-(3,3-Dimethylcycloprop-1-en-1-yl)-5-(*o*-tolyl)pent-4-en-1-ol (**1g**): The representative procedure A was followed using **S1** (462 mg, 1.51 mmol), butyllithium [1.6 M in Hexanes] (1.90 mL, 3.02 mmol) and (*Z*)-5-(*o*-tolyl)-4-pentenal[13] (210 mg, 1.20 mmol). Purification by flash chromatography (SiO_2, hexanes:EtOAc = 5:1) afforded **1g** (129.6 mg, 44%, *Z:E* = 17:1) as a colorless oil.

^{1}H-NMR (300 MHz, $CDCl_3$, data for major isomer) δ = 7.22 – 7.13 (m, 4H), 6.94 (s, 1H), 6.50 (d, *J* = 11.4 Hz, 1H), 5.75 (dt, *J* = 11.4, 7.4 Hz, 1H), 4.65 (t, *J* = 6.6 Hz, 1H), 2.33 (qd, *J* = 7.7, 1.7 Hz, 1H), 2.26 (s, 3H), 1.82 – 1.69 (m, 2H), 1.12 (bs, 6H), (the signal corresponding to the OH could not be allocated).

^{13}C-NMR (75 MHz, $CDCl_3$) δ = 136.6, 136.5, 136.2, 131.6, 129.8, 129.0, 128.9, 126.9, 125.4, 114.4, 68.6, 36.1, 27.8, 27.5, 24.3, 20.9, 19.9.

HRMS (ESI) m/z: $[M+H]^+$ Calcd for $[C_{17}H_{23}O]^+$ 243.1743; found 243.1743.

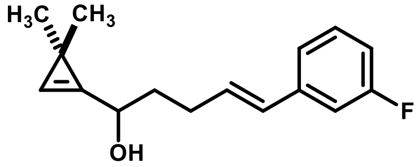

1h

(*E*)-1-(3,3-Dimethylcycloprop-1-en-1-yl)-5-(3-fluorophenyl)pent-4-en-1-ol (**1h**): The representative procedure A was followed using **S1** (1.02 mg, 3.37 mmol), butyllithium [1.6 M in Hexanes] (4.20 mL, 6.75 mmol) and (*E*)-(3-fluorophenyl)-4-pentenal[14] (480 mg, 2.70 mmol). Purification by flash chromatography (SiO_2, hexanes:EtOAc = 5:1) afforded **1h** (263.7 mg, 40%, estimated yield) as a colorless oil.

[13] Nie, Y.; Chen, J.; Zhang, W. Rh(I)-Catalyzed Ring-Opening of Cyclobutanols via C–C Bond Activation: Synthesis of Cis-Olefin with a Remote Aldehyde. *Tetrahedron* **2019**, *75*, 130563.

[14] The aldehyde was obtained by Swern oxidation of (*E*)-5-((3-fluoro)phenyl)pent-4-en-1-ol (Zheng, T.; Chen, R.; Huang, J.; Gonçalves, T. P.; Huang, K. W.; Yeung, Y. Y. Cross-Assembly Confined Bifunctional Catalysis via Non-Covalent Interactions for Asymmetric Halogenation. *Chem* **2023**, *9*), 1255–1269) contained appreciable impurities and was directly used from the crude mixture due to degradation during the purification steps.

^{1}H-NMR (300 MHz, $CDCl_3$) δ = 7.32 – 7.18 (m, 1H), 7.12 – 7.00 (m, 3H), 6.89 (tdd, *J* = 8.4, 2.6, 1.0 Hz, 1H), 6.40 (d, *J* = 15.9 Hz, 1H), 6.26 (dt, *J* = 15.8, 6.7 Hz, 1H), 4.78 – 4.67 (m, 1H), 2.44-2.33 (m, 2H), 1.91 (bs, 1H), 1.89 – 1.77 (m, 2H), 1.22 (s, 3H). 1.21 (s, 3H).
^{13}C-NMR (75 MHz, $CDCl_3$) δ = 163.13 (d, *J* = 244.8 Hz), 139.99 (d, *J* = 7.8 Hz), 136.7, 131.4, 129.91 (d, *J* = 8.4 Hz), 129.5 (d, *J* = 2.6 Hz), 121.9 (d, *J* = 2.7 Hz), 114.6, 113.8 (d, *J* = 21.3 Hz), 112.4 (d, *J* = 21.6 Hz), 68.4, 35.5, 28.8, 27.9, 27.6, 21.0.
^{19}F-NMR (282 MHz, $CDCl_3$) δ = -113.7 – -113.9 (m).
HRMS (ESI) m/z: $[M+H]^+$ Calcd for $[C_{16}H_{20}FO]^+$ 247.1493; found 247.1492.

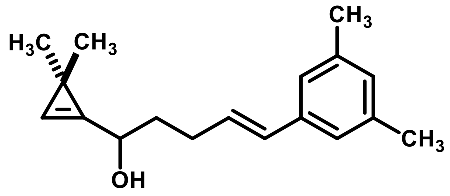

1i

(*E*)-1-(3,3-Dimethylcycloprop-1-en-1-yl)-5-(3,5-dimethylphenyl)pent-4-en-1-ol (**1i**): The representative procedure A was followed using **S1** (307 mg, 1.00 mmol), butyllithium [1.6 M in Hexanes] (1.25 mL, 1.95 mmol) and (*E*)-((3,5-dimethyl)phenyl)-4-pentenal[15] (151 mg, 0.80 mmol). Purification by flash chromatography (SiO_2, hexanes:EtOAc = 5:1) afforded **1i** (124 mg, 60%, estimated yield) as a colorless oil.
^{1}H-NMR (300 MHz, $CDCl_3$) δ = 7.07 (s, 1H), 7.01 (s, 2H), 6.88 (s, 1H), 6.41 (d, *J* = 15.8 Hz, 1H), 6.25 (dt, *J* = 15.8, 6.8 Hz, 1H), 4.75 (t, *J* = 6.2 Hz, 1H), 2.40 (q, *J* = 7.5 Hz, 2H), 2.32 (s, 6H), 1.98 (bs, 1H), 1.91 – 1.76 (m, 2H), 1.27 (s, 3H), 1.26 (s, 3H).
^{13}C-NMR (75 MHz, $CDCl_3$) δ = 138.0, 137.5, 136.8, 130.7, 129.5, 128.8, 123.9, 114.4, 68.5, 35.7, 28.9, 28.0, 27.7, 21.3, 21.0.
HRMS (ESI) m/z: $[M+H]^+$ Calcd for $[C_{18}H_{25}O]^+$ 257.1900; found 257.1901.

[15] The aldehyde was obtained by Swern oxidation of (*E*)-5-((3,5-dimethyl)phenyl)pent-4-en-1-ol (Wang, X.; Miao, H. Z.; Lin, G. Q.; He, Z. T. Ligand-Dictated Regiodivergent Allylic Functionalizations via Palladium-Catalyzed Remote Substitution. *Angew. Chem. Int. Ed.* **2023**, *62*, e202301556) contained appreciable impurities and was directly used from the crude mixture due to degradation during the purification steps.

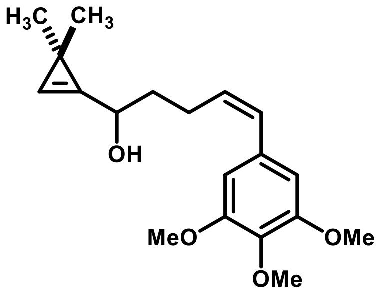

1j

(*Z*)-1-(3,3-Dimethylcycloprop-1-en-1-yl)-5-(3,4,5-trimethoxyphenyl)pent-4-en-1-ol (**1j**): The representative procedure A was followed using **S1** (536 g, 1.75 mmol), butyllithium [1.6 M in Hexanes] (2.20 mL, 3.50 mmol) and (*Z*)-(3,4,5-trimethoxyphenyl)-4-pentenal[16] (359 mg, 1.40 mmol, *E:Z* = 10:1). Purification by flash chromatography (SiO_2, hexanes:EtOAc = 5:1) afforded **1j** (206 mg, 45%, *Z:E* = 10:1) as a colorless oil.

^{1}H-NMR (300 MHz, $CDCl_3$, only data for *Z*-isomer are given) δ = 6.97 (s, 1H), 6.51 (s, 2H), 6.38 (d, *J* = 11.5 Hz, 1H), 5.64 (dt, *J* = 11.5, 7.3 Hz, 1H), 4.69 (t, *J* = 6.6 Hz, 1H), 3.83 (bs, 9H), 2.57 – 2.45 (m, 2H), 1.87 (bs, 1H), 1.86 – 1.73 (m, 2H), 1.12 (bs, 6H).

^{13}C-NMR (75 MHz, $CDCl_3$, only data for *Z*-isomer are given) δ = 152.9, 136.7, 133.1, 131.5, 129.6, 114.4, 105.9, 68.5, 60.9, 56.1, 56.0, 36.1, 27.8, 27.5, 24.6, 20.9.

HRMS (ESI) m/z: $[M+H]^+$ Calcd for $[C_{20}H_{23}O]^+$ 279.1743; found 279.1745.

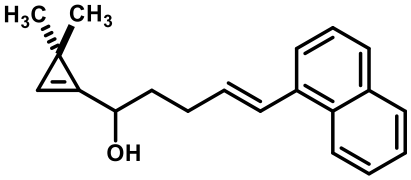

1k

(*E*)-1-(3,3-Dimethylcycloprop-1-en-1-yl)-5-(naphthalen-1-yl)pent-4-en-1-ol (**1k**): The representative procedure A was followed using **S1** (1.17 g, 3.82 mmol), butyllithium [1.6 M in Hexanes] (4.80 mL, 7.65 mmol) and 5-(naphthalene-1-yl)-4-pentenal[17] (643 mg, 3.06 mmol, *E:Z* = 10:1). Purification by flash chromatography (SiO_2, hexanes:EtOAc = 10:1) afforded **1k** (604 mg, 54%, *E:Z* = 10:1) as a colorless oil.

^{1}H-NMR (300 MHz, $CDCl_3$, only data for *E*-isomer are given) δ = 8.25 – 8.11 (m, 1H), 7.97 – 7.86 (m, 1H), 7.81 (d, *J* = 8.2 Hz, 1H), 7.63 (d, *J* = 7.1 Hz, 1H), 7.61 – 7.42 (m, 3H), 7.25 (d, *J* = 15.4 Hz,

[16] For the synthesis of the corresponding aldehyde, see Section 8.
[17] For the synthesis of the corresponding aldehyde, see Section 8.

1H), 7.14 (s, 1H), 6.33 (dt, *J* = 15.5, 7.0 Hz, 1H), 4.85 (t, *J* = 6.5 Hz, 1H), 2.57 (qd, *J* = 7.4, 1.5 Hz, 2H), 2.10 (s, 1H), 2.04 – 1.91 (m, 2H), 1.32 (s, 3H), 1.30 (s, 3H).

^{13}C-NMR (75 MHz, $CDCl_3$, only data for *E*-isomer are given) δ = 136.8, 135.4, 133.7, 133.2, 131.1, 128.6, 127.8, 127.5, 125.9, 125.7, 123.9, 123.6, 114.6, 68.5, 35.7, 29.3, 28.0, 28.0, 27.7, 21.1 (one signal corresponds to two carbon atoms).

HRMS (ESI) m/z: $[M+H]^+$ Calcd for $[C_{20}H_{23}O]^+$ 279.1743; found 279.1745.

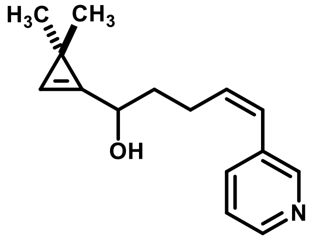

1l

(*Z*)-1-(3,3-Dimethylcycloprop-1-en-1-yl)-5-(pyridin-3-yl)pent-4-en-1-ol (**1l**): The representative procedure A was followed using **S1** (617 g, 2.00 mmol), butyllithium [1.6 M in Hexanes] (2.50 mL, 7.65 mmol) and (*Z*)-5-(pyridin-3-yl)-4-pentenal[18] (260 mg, 1.60, estimated). Purification by flash chromatography (SiO_2, hexanes:EtOAc = 10:1) to afford **1l** (90 mg, 24%, estimated yield) as a colorless oil.

^{1}H-NMR (300 MHz, $CDCl_3$) δ = 8.50 (d, *J* = 2.2 Hz, 1H), 8.41 (dd, *J* = 4.8, 1.7 Hz, 1H), 7.61 (dt, *J* = 7.9, 2.2 Hz, 1H), 7.31 – 7.18 (m, 1H), 6.97 (d, *J* = 0.7 Hz, 1H), 6.39 (d, *J* = 11.6 Hz, 1H), 5.83 (dt, *J* = 11.6, 7.4 Hz, 1H), 4.70 (t, *J* = 6.2 Hz, 1H), 3.20 (bs, 1H), 2.48 (qd, *J* = 7.6, 1.8 Hz, 2H), 1.91 – 1.73 (m, 2H), 1.13 (s, 3H), 1.12 (s, 3H).

^{13}C-NMR (75 MHz, $CDCl_3$) δ = 150.0, 147.7, 137.1, 136.3, 135.0, 133.6, 126.2, 123.6, 114.8, 68.5, 36.4, 28.2, 27.9, 25.0, 21.2.

HRMS (ESI) m/z: $[M+H]^+$ Calcd for $[C_{15}H_{20}NO]^+$ 230.1539; found 230.1537.

---

[18] The aldehyde was obtained by Swern oxidation of (*Z*)-5-(pyridin-3-yl)pent-4-en-1-ol (Fanourakis, A.; Hodson, N. J.; Lit, A. R.; Phipps, R. J. Substrate-Directed Enantioselective Aziridination of Alkenyl Alcohols Controlled by a Chiral Cation. *J. Am. Chem. Soc.* **2023**, *145*, 7516–7527) contained appreciable impurities and was directly used from the crude mixture due to extensive degradation during purification attempts.

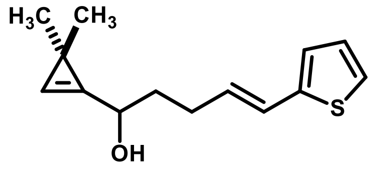

1m

(*E*)-1-(3,3-Dimethylcycloprop-1-en-1-yl)-5-(thiophen-2-yl)pent-4-en-1-ol (**1m**): The representative procedure A was followed using **S1** (312 mg, 1.02 mmol), butyllithium [1.6 M in Hexanes] (1.30 mL, 7.65 mmol) and (*E*)-5-(thiophen-2-yl)-4-pentenal[19] (135 mg, 0.81 mmol). Purification by flash chromatography (SiO_2, hexanes:EtOAc = 10:1) to afford **1m** (94 mg, 49%) as a colorless oil.

^{1}H-NMR (300 MHz, $CDCl_3$) δ = 7.12 (d, *J* = 5.1 Hz, 1H), 7.08 (s, 1H), 6.99 – 6.93 (m, 1H), 6.90 (d, *J* = 3.3 Hz, 1H), 6.59 (d, *J* = 15.6 Hz, 1H), 6.11 (dt, *J* = 15.6, 7.0 Hz, 1H), 4.74 (q, *J* = 5.9 Hz, 1H), 2.31 – 2.45 (m, 2H), 1.99 – 1.71 (m, 3H), 1.24 (s, 3H), 1.23 (s, 3H).

^{13}C-NMR (75 MHz, $CDCl_3$) δ = 143.2, 137.1, 130.2, 127.7, 124.9, 124.2, 123.7, 115.0, 68.8, 35.9, 29.0, 28.3, 28.0, 21.4.

HRMS (ESI) m/z: $[M+H]^+$ Calcd for $[C_{14}H_{19}OS]^+$ 235.1151; found 235.1150.

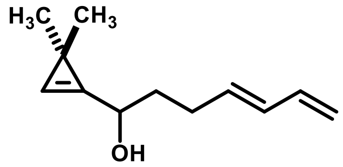

1n

(*E*)-1-(3,3-Dimethylcycloprop-1-en-1-yl)hepta-4,6-dien-1-ol (**1n**): The representative procedure A was followed using **S1** (729.8 mg, 2.35 mmol), butyllithium [1.6 M in Hexanes] (3.00 mL, 4.70 mmol) and (*E*)-4,6-heptadienal[20] (207 mg, 1.88 mmol). Purification by flash chromatography (SiO_2, hexanes:EtOAc = 5:1) to afford **1n** (220 mg, 52%) as a colorless oil.

^{1}H-NMR (300 MHz, $CDCl_3$) δ = 7.04 (s, 1H), 6.78 – 6.55 (m, 1H), 6.05 (t, *J* = 10.9 Hz, 1H), 5.58 – 5.40 (m, 1H), 5.21 (d, *J* = 17.0 Hz, 1H), 5.12 (d, *J* = 10.2 Hz, 1H), 4.68 (t, *J* = 6.5 Hz, 1H), 2.36 (dtd, *J* = 11.5, 7.6, 4.1 Hz, 2H), 2.26 – 2.03 (m, 1H), 1.90 – 1.62 (m, 2H), 1.21 (s, 3H), 1.20 (s, 3H).

^{13}C-NMR (75 MHz, $CDCl_3$) δ = 136.8, 132.2, 131.6, 130.1, 117.4, 114.5, 68.4, 35.8, 28.0, 27.6, 23.7, 21.0.

HRMS (ESI) m/z: $[M+H]^+$ Calcd for $[C_{12}H_{19}O]^+$ 179.1430; found 179.1431.

---

[19] Yu, H.; Lee, R.; Kim, H.; Lee, D. Diastereoselective Construction of Trans-2-Alkyl-6-Aryl-3,6-Dihydro-2H-Pyrans via Dehydrogenative Cycloetherification Promoted by DDQ. *Org. Lett.* **2021**, *23*, 1135–1140.

[20] Serba, C.; Lagoutte, R.; Winssinger, N. Rapid and Scalable Synthesis of Cis-Fused Guaiane-Type Sesquiterpenes. *Eur. J. Org. Chem.* **2016**, *2016*, 644–646.

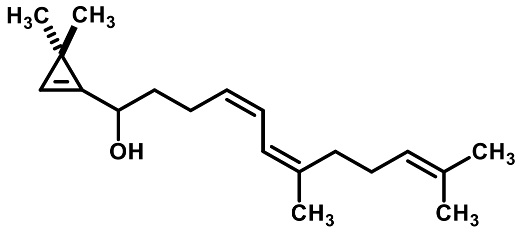

1o

(4*Z*,6*Z*)-1-(3,3-Dimethylcycloprop-1-en-1-yl)-7,11-dimethyldodeca-4,6,10-trien-1-ol (**1o**): The representative procedure A was followed using **S1** (459 mg, 1.50 mmol), butyllithium [1.6 M in Hexanes] (1.90 mL, 3.04 mmol) and (4*Z*,6*E*)-7,11-dimethyldodeca-4,6,10-trienal[21] (247 mg, 1.20 mmol). Purification by flash chromatography (SiO_2, hexanes:EtOAc = 10:1) to afford **1o** (220 mg, 67%; *Z:E* = 10:1) as a colorless oil.

^{1}H-NMR (300 MHz, $CDCl_3$, only data for the *Z*-isomer are listed) δ = 7.04 (s, 1H), 6.23 (t, *J* = 11.1 Hz, 1H), 6.11 (d, *J* = 11.4 Hz, 1H), 5.38 (dt, *J* = 10.8, 7.6 Hz, 1H), 5.10 (bs, 1H), 4.69 (bs, 1H), 2.35 (dt, *J* = 13.1, 6.8 Hz, 2H), 2.11 (bs, 4H), 1.88 – 1.72 (m, 6H), 1.69 (s, 3H), 1.62 (s, 3H), 1.21 (s, 3H), 1.20 (s, 3H).

^{13}C-NMR (101 MHz, $CDCl_3$, only data for the *Z*-isomer are listed) δ = 139.2, 136.8, 131.6, 128.5, 125.5, 124.0, 119.7, 114.3, 68.6, 40.3, 35.9, 27.9, 27.6, 26.7, 25.7, 23.4, 20.9, 17.7, 16.5.

HRMS (ESI) m/z: $[M+H]^+$ Calcd for $[C_{19}H_{31}O]^+$ 275.2369; found 275.2368.

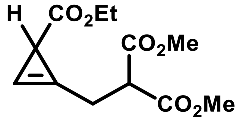

S2p

Dimethyl 2-((3-(ethoxycarbonyl)cycloprop-1-en-1-yl)methyl)malonate (**S2p**): This compound was prepared following representative procedure B (first step, see pages S4-S5).

^{1}H-NMR (300 MHz, $CDCl_3$) δ = 6.48 (q, *J* = 1.4 Hz, 1H), 4.11 (qd, *J* = 7.2, 1.1 Hz, 2H), 3.76 (s, 3H), 3.75 (s, 3H), 3.69 (t, *J* = 7.5 Hz, 1H), 3.12 (d, *J* = 7.6 Hz, 2H), 2.15 (d, *J* = 1.5 Hz, 1H), 1.25 (t, *J* = 7.2 Hz, 3H).

^{13}C-NMR (75 MHz, $CDCl_3$) δ = 175.7, 168.5, 112.2, 97.0, 60.4, 52.8, 49.3, 24.5, 19.9, 14.3.

HRMS (ESI) m/z: $[M+Na]^+$ Calcd for $[C_{12}H_{16}NaO_6]^+$ 279.0839; found 279.0847.

[21] For the synthesis of the corresponding aldehyde, see Section 8.

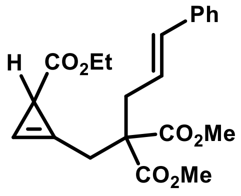

1p

Dimethyl 2-cinnamyl-2-((3-(ethoxycarbonyl)cycloprop-1-en-1-yl)methyl)malonate (**1p**): This compound was prepared following representative procedure B (second step, see pages S4-S5).

^{1}H-NMR (300 MHz, $CDCl_3$) δ = 7.41 – 7.22 (m, 5H), 6.57 (q, *J* = 1.5 Hz, 1H), 6.50 (d, *J* = 15.7 Hz, 1H), 6.04 (dt, *J* = 15.5, 7.6 Hz, 1H), 4.17 (q, *J* = 6.9 Hz, 2H), 3.79 (s, 3H), 3.79 (s, 3H), 3.21 (s, 2H), 3.01 – 2.88 (m, 2H), 2.18 (d, *J* = 1.6 Hz, 1H), 1.29 (t, *J* = 7.1 Hz, 3H).

^{13}C-NMR (75 MHz, $CDCl_3$) δ = 175.6, 170.5, 170.4, 136.9, 134.7, 128.5, 127.5, 126.3, 123.1, 111.3, 97.8, 60.4, 56.7, 52.8, 36.4, 28.5, 19.7, 14.4.

HRMS (ESI) m/z: $[M+Na]^+$ Calcd for $[C_{21}H_{24}NaO_6]^+$ 395.1465; found 395.1462.

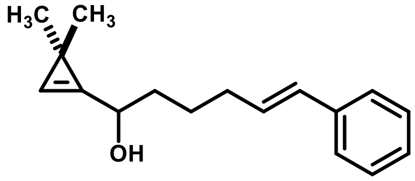

1q

(*E*)-1-(3,3-Dimethylcycloprop-1-en-1-yl)-6-phenylhex-5-en-1-ol (**1q**): The representative procedure A was followed using **S1** (390 g, 1.27 mmol), butyllithium [1.6 M in Hexanes] (1.60 mL, 2.55 mmol) and 6-phenyl-5-pentenal[7] (210 mg, 1.20 mmol, *E:Z* = 7:1). Purification by flash chromatography (SiO_2, hexanes:EtOAc = 10:1) afforded **1q** (151 mg, 52%, *E:Z* = 6:1) as a colorless oil.

^{1}H-NMR (300 MHz, $CDCl_3$, only data for *E*-isomer are given) δ = 7.56 – 7.18 (m, 5H), 7.06 (s, 1H), 6.43 (d, *J* = 15.8 Hz, 1H), 6.25 (dt, *J* = 15.8, 6.8 Hz, 1H), 4.73 (bs, 1H), 2.30 (q, *J* = 6.9 Hz, 2H), 1.85 – 1.61 (m, 4H), 1.23 (s, 3H), 1.22 (s, 3H).

^{13}C-NMR (75 MHz, $CDCl_3$, only data for *E*-isomer are given) δ = 137.8, 136.8, 130.4, 130.2, 128.5, 126.9, 126.0, 114.3, 69.0, 35.6, 32.8, 27.9, 27.6, 25.0, 21.0.

HRMS (ESI) m/z: $[M+H]^+$ Calcd for $[C_{17}H_{23}O]^+$ 243.1743; found 243.1740.

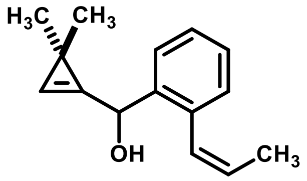

1r

(*Z*)-(3,3-Dimethylcycloprop-1-en-1-yl)(2-(prop-1-en-1-yl)phenyl)methanol (**1r**): The representative procedure A was followed using **S1** (637 g, 2.07 mmol), butyllithium [1.6 M in Hexanes] (2.60 mL, 4.15 mmol) and 2-(1-propen-1-yl)benzaldehyde[22] (243 mg, 1.66 mmol, *E:Z* = 4:1). Purification by flash chromatography (SiO_2, hexanes:EtOAc = 10:1) afforded **1r** (238 mg, 66%, *Z:E* = 2.5:1) as a colorless oil.

^{1}H-NMR (300 MHz, $CDCl_3$, * denotes signal corresponding to the *E*-isomer) δ = 7.61 – 7.39 (m, 1H, overlapped with *E*-isomer), 7.38 – 7.18 (m, 3H, overlapped with *E*-isomer), 7.12 (s, 1H*), 7.10 (s, 1H), 6.76 (dd, *J* = 15.5, 1.6 Hz, 1H*), 6.63 (dd, *J* = 11.4, 1.7 Hz, 1H), 6.17 (dq, *J* = 15.5, 6.6 Hz, 1H*), 6.01 (d, *J* = 3.0 Hz, 1H*), 5.99 – 5.85 (m, 2H), 2.55 – 2.36 (m, 1H, overlapped with *E*-isomer), 1.95 (dd, *J* = 6.6, 1.7 Hz, 3H*), 1.76 (dd, *J* = 7.0, 1.8 Hz, 3H), 1.21 (s, 3H*), 1.18 (s, 3H), 1.00 (s, 3H*), 0.99 (s, 1H).

^{13}C-NMR (75 MHz, $CDCl_3$, * denotes signal corresponding to the *E*-isomer when allocated) δ = 138.9, 137.2*, 136.3*, 136.1, 136.0*, 135.0, 129.7, 128.4, 127.9*, 127.9*, 127.7, 127.3, 127.2, 127.1*, 126.2, 126.0*, 114.6, 114.5, 68.50*, 68.46, 27.54*, 27.49, 26.9, 26.8*, 22.0*, 21.7, 18.9, 14.5.

HRMS (ESI) m/z: $[M+H]^+$ Calcd for $[C_{15}H_{19}O]^+$ 215.1430; found 215.1414.

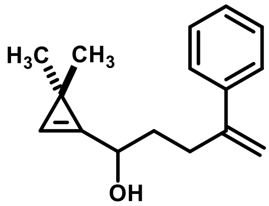

1s

1-(3,3-Dimethylcycloprop-1-en-1-yl)-4-phenylpent-4-en-1-ol (**1s**): The representative procedure A was followed using **S1** (615 g, 2.00 mmol), butyllithium [1.6 M in Hexanes] (2.50

[22] Yuan, J.; Liu, C.; Chen, Y.; Zhang, Z.; Yan, D.; Zhang, W. Rhodium-Catalyzed Intramolecular Hydroacylation of 1,2-Disubstituted Alkenes for the Synthesis of 2-Substituted Indanones. *Tetrahedron* **2019**, *75*, 269–277.

mL, 4.00 mmol) and γ-methylenebenzenebutanal[23] (301 mg, 1.90 mmol). Purification by flash chromatography (SiO_2, hexanes:EtOAc = 10:1) afforded **1s** (305 mg, 70%) as a colorless oil.

^{1}H-NMR (300 MHz, $CDCl_3$) δ = 7.44 (d, *J* = 7.1 Hz, 2H), 7.37 – 7.32 (m, 2H), 7.32 – 7.25 (m, 1H), 7.05 (s, 1H), 5.33 (s, 1H), 5.13 (s, 1H), 4.85 – 4.61 (m, 1H), 2.74 – 2.58 (m, 2H), 1.94 – 1.72 (m, 3H), 1.21 (bs, 6H).

^{13}C-NMR (101 MHz, $CDCl_3$) δ = 148.0, 141.0, 136.7, 128.3, 127.5, 126.1, 114.5, 112.6, 68.5, 34.8, 31.0, 27.9, 27.6, 20.9.

HRMS (ESI) m/z: $[M+H]^+$ Calcd for $[C_{16}H_{21}O]^+$ 229.1587; found 229.1584.

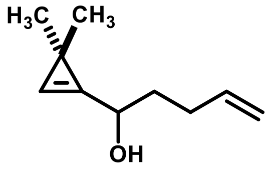

3

1-(3,3-Dimethylcycloprop-1-en-1-yl)pent-4-en-1-ol (**3**): The representative procedure A was followed using **S1** (621 mg, 2.02 mmol), butyllithium [1.6 M in Hexanes] (2.5 mL, 4.05 mmol) and aldehyde 4-pentenal (136 mg, 1.60 mmol). Purification by flash chromatography (SiO_2, hexanes:EtOAc = 5:1) afforded **3** (121 mg, 50 %) as a colorless oil. The characterization data are in agreement with those reported in the literature.[2]

[23] Chen, L. Y.; Chen, J. R.; Cheng, H. G.; Lu, L. Q.; Xiao, W. J. Enantioselective Synthesis of Tetrahydrofuran Derivatives by Sequential Henry Reaction and Iodocyclization of γ,δ-Unsaturated Alcohols. *Eur. J. Org. Chem.* **2014**, *2014*, 4714–4719.

3. Screening for the [2+2] photocycliaddition of cyclopropene 1a.

Photo-reactor employed.

Photochemical reactions were performed with 451 nm LEDs (OSRAM Oslon® SSL 80 royal-blue LEDs) or UV-Vis LEDs (LED-TECH® LT-1877 395-410 nm) which were installed at the bottom of a custom-made 8 flat-bottom Schlenk tubes holder (the distance between the flat-bottom Schlenk tube and the light source was measured to be ~7 mm), equipped with a water recirculator and a fun cooling system (the temperature measured in the solution when irradiated was 25–30 °C) and a magnetic stirrer (~ 300 rpm). The material of Schlenk tubes is borosilicate glass and they were used without any filter.

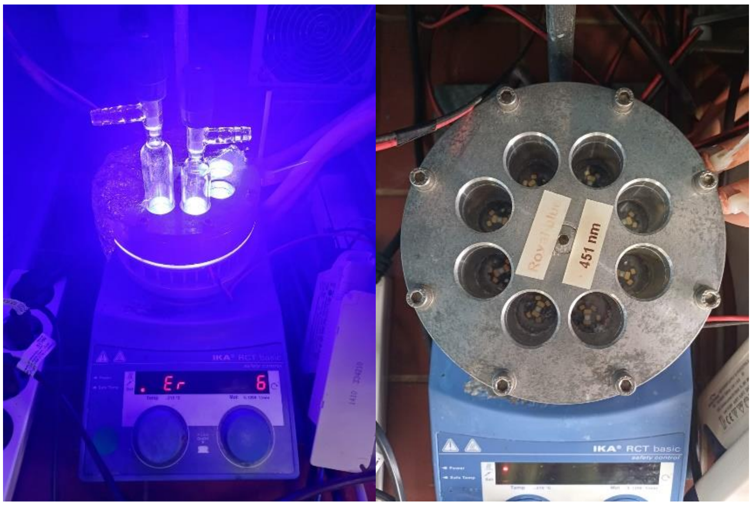

Sensitizers employed.

All the sensitizers were commercially available and used as received, except 4CzIPN, which was prepared according to a reported procedure.[24] E_T values (kcal/mol) given are obtained from literature.[25]

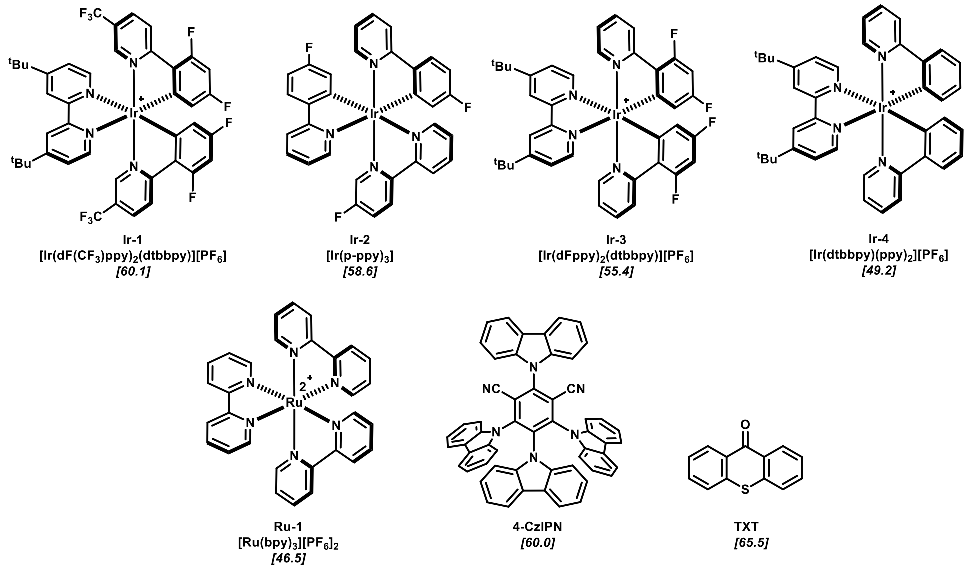

[24] Engle, S. M.; Kirkner, T. R.; Kelly, C. B. Preparation of 2,4,5,6-Tetra(9*H*-Carbazol-9-Yl)Isophthalonitrile. *Org. Synth.* **2019**, *96*, 455–473.

[25] (a) For iridium complexes, see: Singh, A.; Teegardin, K.; Kelly, M.; Prasad, K. S.; Krishnan, S.; Weaver, J. D. Facile Synthesis and Complete Characterization of Homoleptic and Heteroleptic Cyclometalated Iridium(III) Complexes for Photocatalysis. *J. Organomet. Chem.* **2015**, *776*, 51–59. (b) For ruthenium complexes, see: Demas, J. N.; Adamson, A. W. A New Photosensitizes Tris(2,2'-Bipyridine)Ruthenium(II) Chloride. *J. Am. Chem. Soc.* **1971**, *93*, 1800–1801. (c) For 4CzIPN, see: Martínez-Haya, R.; Marzo, L.; König, B. Reinventing the De Mayo reaction: synthesis of 1,5-diketones or 1,5-ketoesters via visible light [2+2] cycloaddition of β-diketones or β-ketoesters with styrenes. *Chem. Commun.* 2018, **54**, 11602–11605. (d) For thioxanthone, see: Calvert, J.; Pitts, J. Photochemistry, John Wiley and Sons, Inc., New York, N. Y., **1966**, p. 298.

***Representative procedure C**: Screening reaction conditions.*

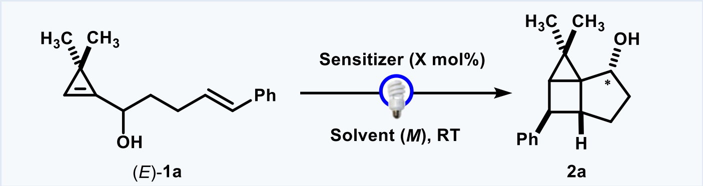

A solution of cyclopropene **1a** (0.1-0.15 mmol) and the sensitizer (1.0-5.0 mol%) (the solvent was previously degassed by Ar bubbling for 10 min) was irradiated until the disappearance of **1a** (TLC analysis) or after 24 h (when **1a** was not completely consumed) (by cooling with water recirculation and a fun system, the temperature was bellow 30 °C). The solvent was removed under reduced pressure and the resulting residue was analyzed by ^{1}H NMR (CH_2Br_2 used as internal standard). The results are summarized in Table S1.

Table S1. Screening results summary.

**Sensitizer** (x mol%)	**Irradiation source**	**Solvent** (x *M*)	**t** (h)	**2a** NMR Yield (%)[a]	**1a** NMR Yield (%)[a]
Ir-1 (1.0 mol%)	20W CLF	CH_3CN (0.02 M)	24	20 (*dr* = 6:1)	57 (*Z/E* = 4.2:1)
Ir-1 (1.0 mol%)	Blue LED	CH_3CN (0.02 M)	2.0	78 (*dr* = 5.0:1)	
Ir-1 (1.0 mol%)	Blue LED	CH_3CN (0.05 M)	2.0	58 (*dr* = 4.0:1)	
Ir-1 (1.0 mol%)	Blue LED	CH_3CN (0.01 M)	2.0	62 (*dr* = 4.4:1)	
Ir-1 (1.0 mol%)	Blue LED	CH_3CN (0.0063 M)	1.5	53 (*dr* = 4:1)	
Ir-1 (1.0 mol%)	Blue LED	Toluene (0.02 M)	6.0	37 (*dr* = 4:1)	
Ir-1 (1.0 mol%)	Blue LED	THF (0.02 M)	3.5	46 (*dr* = 4.4:1)[b]	
Ir-1 (1.0 mol%)	Blue LED	DMSO (0.02 M)	3.0	54 (*dr* = 5:1)	
Ir-1 (1.0 mol%)	Blue LED	DMF (0.02 M)	3.0	55 (*dr* = 4:1)	
Ir-1 (1.0 mol%)	Blue LED	MeOH (0.02 M)	3.0	38 (*dr* = 9:1)[b]	
Ir-1 (1.0 mol%)	Blue LED	CH_2Cl_2 (0.02 M)	1.5	30 (*dr* = 9:1)[b]	
Ir-1 (1.0 mol%)	Blue LED	CH_3CN (0.02 M)[c]	2	53 (*dr* = 7:1)[b]	
Ir-2 (1.0 mol%)	Blue LED	CH_3CN (0.02 M)	24	40 (*dr* = 4:1)	30 (*Z/E* = 3:1)
Ir-3 (1.0 mol%)	Blue LED	CH_3CN (0.02 M)	2	46 (*dr* = 6:1)[b]	
Ir-4 (1.0 mol%)	Blue LED	CH_3CN (0.02 M)	24	22 (*dr* = 4.0:1)	
Ru-1 (1.0 mol%)	Blue LED	CH_3CN (0.02 M)	24	n.d.	50 (only *E*)
Ru-2 (1.0 mol%)	Blue LED	CH_3CN (0.02 M)	24	n.d.	30 (only *E*)
4CzIPN (5.0 mol%)	Blue LED	CH_3CN (0.02 M)	24	19 (*dr* =3:1)	40 (*Z/E* = 2.5:1)
TXT (5.0 mol%)	Blue LED	CH_3CN (0.02 M)	24	15 (*dr* = 4:1)	66 (*Z/E* =1.4:1)
TXT (5.0 mol%)	Violet LED	CH_3CN (0.02 M)	2	65 (*dr* = 4:1))[b]	
	Blue LED	CH_3CN (0.02 M)	24	n.d.	>95% (only *E*)
Ir-1 (1.0 mol%)	dark	CH_3CN (0.02 M)	24	n.d.	>95% (only *E*)

[a] Determined by ^{1}H NMR using dibromomethane as internal standard [b] Complex reaction mixture was observed. [c] With undestilled MeCN under open air conditions. (n.d. = not detected).

4. Synthesis of housanes 2 via [2+2] photocycloaddition.

4.1. Representative procedures.

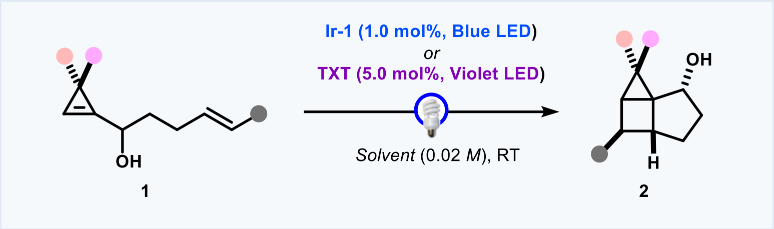

Representative procedure D.

To a solution of cyclopropene **1** (0.10-0.30 mmol) in previously degassed (Ar bubbling for 10 min) MeCN (0.02 M), [Ir(dF(CF_3)ppy)$_2$(dtbbpy)][PF_6] (**Ir-1**, 1.0 mol%) was added. The resulting solution was irradiated with blue LED (451 nm) until de disappearance of the starting cyclopropene. The solvent was removed under reduced pressure and the resulting residue was purified by flash chromatography (deactivated SiO_2,[26] hexanes/EtOAc) to afford the corresponding housanes **2** (mixture of diastereoisomers). For the isolation of pure major diastereoisomers further purification was required.

Representative procedure E.

To a solution of cyclopropene **1** (0.10-0.30 mmol) in previously degassed (Ar bubbling for 10 min) MeCN (0.02 M), thioxanthone (**TXT**, 5.0 mol%) was added. The resulting solution was irradiated with violet LED (395-410 nm) until de disappearance of the starting cyclopropene. The solvent was removed under reduced pressure and the resulting residue was purified by flash chromatography (deactivated SiO_2,[26] hexanes/EtOAc) to afford the corresponding housanes **2** (mixture of diastereoisomers). For the isolation of pure major diastereoisomers further purification was required.

[26] SiO_2 was treated for 3 h with a solution of NaH_2PO_4 (4% w/w) filtered and dried in an oven at 120 °C for 72 h.

4.2. Characterization data for housanes 2.

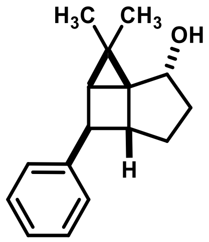

2a

(1*R,3*S**,4*R**,5*R**,8*R**)-2,2-Dimethyl-4-phenyltricyclo[3.3.0.0^{1,3}]octan-8-ol** (**2a**): The representative procedure D was followed using cyclopropene **1a** (30.0 mg, 0.13 mmol) and **Ir-1** (1.0 mg, 1.0 mol%) in MeCN (0.02 M). After 2 h, analysis of the crude mixture indicated the disappearance of **1a** (NMR yield = 78%, *dr* = 5:1). Purification by flash chromatography (deactivated SiO_2, hexanes:EtOAc = 20:1) enabled the isolation of both diastereoisomers: **2a** (19.7 mg, 66%, *anti*-OH isomer, *8R**, colorless oil); **2a'** (2.7 mg, 9%, *syn*-OH isomer, *8S**, colorless oil). Combined yield = 75%.

The representative procedure E was followed using cyclopropene **1a** (22.5 mg, 0.10 mmol) and **TXT** (1.1 mg, 5.0 mol%) in MeCN (0.02 M). After 2 h, analysis of the crude mixture indicated the disappearance of **1a** (NMR yield = 65%, *dr* = 4:1). Purification by flash chromatography (deactivated SiO_2, hexanes:EtOAc = 20:1) enabled the isolation of two fractions containing **2a** with a mixture of diastereoisomers (13.5 mg, 60% combined yield, colorless oil).

Characterization data for the mayor isomer (**2a**):

^{1}H-NMR (300 MHz, $CDCl_3$) δ = 7.33 – 7.24 (m, 2H), 7.23 – 7.12 (m, 3H), 4.34 (d, *J* = 3.7 Hz, 1H), 3.25 (t, *J* = 4.5 Hz, 1H), 2.42 – 2.30 (m, 1H), 2.23 – 2.10 (m, 1H), 2.10 – 1.97 (m, 1H), 1.97 – 1.79 (m, 2H), 1.65 (bs, 1H), 1.58 (dd, *J* = 5.1, 1.4 Hz, 1H), 1.14 (s, 3H), 0.82 (s, 3H).

^{13}C-NMR (75 MHz, $CDCl_3$) δ = 143.7, 127.8, 126.0, 125.2, 69.7, 45.6, 43.7, 41.5, 39.0, 32.0, 25.7, 23.4, 21.6, 18.7.

HRMS (ESI) m/z: $[M+H]^+$ Calcd for $[C_{16}H_{21}O]^+$ 229.1587; found 229.1584.

Characterization data for the minor isomer (**2a'**):

^{1}H-NMR (600 MHz, $CDCl_3$) δ = 7.24 (d, *J* = 7.6 Hz, 2H), 7.17 (dt, *J* = 8.2, 1.3 Hz, 2H), 7.13 (td, *J* = 7.3, 1.2 Hz, 1H), 4.15 (d, *J* = 4.1 Hz, 1H), 3.18 (t, *J* = 4.5 Hz, 1H), 2.40 (dd, *J* = 8.5, 3.7 Hz, 1H), 2.33 – 2.25 (m, 1H), 2.23 – 2.14 (m, 1H), 2.06 – 2.01 (m, 1H), 2.01 (s, 1H), 1.96 (dd, *J* = 12.9, 7.7 Hz, 1H), 1.36 (dd, *J* = 5.3, 1.0 Hz, 1H), 1.18 (s, 3H), 0.95 (s, 3H).

^{13}C-NMR (101 MHz, $CDCl_3$) δ = 144.1, 128.0, 126.0, 125.4, 74.2, 44.5, 41.5, 39.1, 36.6, 29.6, 28.6, 26.9, 22.4, 20.1.

HRMS (ESI) m/z: $[M+H]^+$ Calcd for $[C_{16}H_{21}O]^+$ 229.1587; found 229.1583.

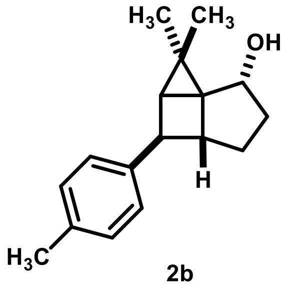

2b

(1*R,3*S**,4*R**,5*R**,8*R**)-2,2-Dimethyl-4-(*p*-tolyl)tricyclo[3.3.0.0^{1,3}]octan-8-ol** (**2b**): The representative procedure D was followed using cyclopropene **1b** (24.5 mg, 0.10 mmol) and **Ir-1** (0.9 mg, 1.0 mol%) in MeCN (0.02 M). After 3.5 h, analysis of the crude mixture indicated the disappearance of **1b** (NMR yield = 75%, *dr* = 5:1). Purification by flash chromatography (deactivated SiO_2, hexanes:EtOAc = 20:1) enabled the isolation of both diastereoisomers: **2b** (13.8 mg, 56%, *anti*-OH isomer, *8R**, colorless oil); **2b'** (3.1 mg, 13%, *syn*-OH isomer, *8S**, colorless oil). Combined yield = 69%.

The representative procedure E was followed using cyclopropene **1b** (26.2 mg, 0.11 mmol) and **TXT** (1.0 mg, 5.0 mol%) in MeCN (0.02 M). After 3.5 h, analysis of the crude mixture indicated the disappearance of **1b** (NMR yield = 68%, *dr* = 4:1). Purification by flash chromatography (deactivated SiO_2, hexanes:EtOAc = 10:1) enabled the isolation of two fractions containing **2b** with a mixture of diastereoisomers (16.0 mg, 66% combined yield, colorless oil).

Characterization data for the mayor isomer (**2b**):

^{1}H-NMR (300 MHz, $CDCl_3$) δ = 7.09 (s, 4H), 4.33 (dd, *J* = 5.0, 2.2 Hz, 1H), 3.22 (t, *J* = 4.1 Hz, 1H), 2.33 (s, 3H, overlapped signal), 2.40 – 2.27 (m, 1H, overlapped signal), 2.19 – 2.09 (m, 1H), 2.06 – 1.98 (m, 1H), 1.93 – 1.80 (m, 2H), 1.56 (dd, *J* = 5.1, 1.5 Hz, 1H), 1.14 (s, 3H), 0.83 (s, 3H) (the signal corresponding to the OH could not be allocated).

^{13}C-NMR (75 MHz, $CDCl_3$) δ = 140.6, 134.6, 128.5, 125.9, 69.7, 45.6, 43.5, 41.5, 39.0, 32.1, 25.6, 23.4, 21.6, 21.1, 18.8.

HRMS (ESI) m/z: $[M+H]^+$ Calcd for $[C_{17}H_{23}O]^+$ 243.1743; found 243.1742.

Characterization data for the minor isomer (**2b'**):

^{1}H-NMR (400 MHz, $CDCl_3$) δ = 7.05 (s, 4H), 4.14 (d, *J* = 3.5 Hz, 1H), 3.14 (t, *J* = 4.5 Hz, 1H), 2.42 – 2.31 (m, 2H), 2.30 (s, 3H), 2.28 – 2.12 (m, 2H), 2.07 – 1.90 (m, 2H), 1.34 (dd, *J* = 5.3, 1.0 Hz, 1H), 1.17 (s, 3H), 0.96 (s, 3H).

^{13}C-NMR (101 MHz, $CDCl_3$) δ = 141.0, 134.8, 128.6, 125.9, 74.3, 44.5, 41.2, 39.1, 36.6, 29.6, 28.6, 26.9, 22.4, 21.2, 20.1.

HRMS (ESI) m/z: $[M+H]^+$ Calcd for $[C_{17}H_{23}O]^+$ 243.1743; found 243.1738.

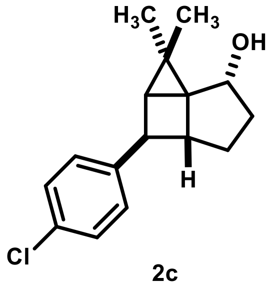

2c

(1*R,3*S**,4*R**,5*R**,8*R**)-4-(4-Chlorophenyl)-2,2-dimethyltricyclo[3.3.0.0^{1,3}]octan-8-ol** (**2c**): The representative procedure D was followed using cyclopropene **1c** (53.2 mg, 0.20 mmol) and **Ir-1** (1.8 mg, 1.0 mol%) in MeCN (0.02 M). After 2 h, analysis of the crude mixture indicated the disappearance of **1c** (NMR yield = 75%, *dr* = 4:1). Purification by flash chromatography (deactivated SiO_2, hexanes:EtOAc = 10:1) enabled the isolation of **2c** (38 mg, 71%, colorless oil) as a mixture of diastereoisomers. *Samples of isolated diastereoisomers of **2c** were obtained after a further purification by flash chromatography (deactivated SiO_2, hexanes:EtOAc = 50:1 to 10:1).*

The representative procedure E was followed using cyclopropene **1c** (52.3 mg, 0.19 mmol) and **TXT** (2.0 mg, 5.0 mol%) in MeCN (0.02 M). After 2 h, analysis of the crude mixture indicated the disappearance of **1c** (NMR yield = 69%, *dr* = 4:1). Purification by flash chromatography (deactivated SiO_2, hexanes:EtOAc = 10:1) enabled the isolation of **2c** (36.0 mg, 69%, colorless oil) as a mixture of diastereoisomers.

Characterization data for the mayor isomer (**2c**):

^{1}H-NMR (300 MHz, $CDCl_3$) δ = 7.24 (d, *J* = 8.4 Hz, 2H), 7.13 (dd, *J* = 8.5, 1.2 Hz, 2H), 4.33 (d, *J* = 3.4 Hz, 1H), 3.19 (t, *J* = 4.0 Hz, 1H), 2.49 – 2.27 (m, 1H), 2.19 – 2.06 (m, 1H), 2.06 – 1.96 (m, 1H), 1.95 – 1.76 (m, 2H), 1.56 (dd, *J* = 5.1, 1.5 Hz, 1H), 1.21 (bs, 1H), 1.13 (s, 3H), 0.80 (s, 3H).

^{13}C-NMR (75 MHz, $CDCl_3$) δ = 142.3, 130.9, 127.9, 127.4, 69.6, 45.4, 43.1, 41.5, 39.0, 31.9, 25.7, 23.2, 21.5, 18.7.

HRMS (ESI) m/z: $[M+H]^+$ Calcd for $[C_{16}H_{21}ClO]^+$ 263.1197; found 263.1196.

Characterization data for the minor isomer (**2c'**):

^{1}H-NMR (400 MHz, $CDCl_3$) δ = 7.24 (d, *J* = 8.3 Hz, 2H), 7.11 (dd, *J* = 8.5, 1.2 Hz, 2H), 4.17 (d, *J* = 3.7 Hz, 1H), 3.15 (t, *J* = 4.5 Hz, 1H), 2.52 – 2.13 (m, 3H), 2.1 – 1.86 (m, 2H), 1.36 (d, *J* = 4.3 Hz, 2H), 1.28 (bs, 1H), 1.20 (s, 3H), 0.96 (s, 3H).

^{13}C-NMR (75 MHz, $CDCl_3$) δ = 142.5, 130.9, 127.9, 127.3, 73.9, 44.4, 40.8, 39.0, 36.4, 29.4, 28.1, 26.8, 22.2, 19.9.

HRMS (ESI) m/z: $[M+H]^+$ Calcd for $[C_{16}H_{21}ClO]^+$ 263.1197; found 263.1201.

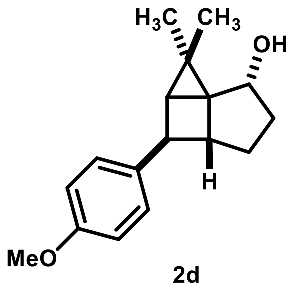

2d

(1*R,3*S**,4*R**,5*R**,8*R**)-4-(4-Methoxyphenyl)-2,2-dimethyltricyclo[3.3.0.0^{1,3}]octan-8-ol** (**2d**): The representative procedure D was followed using cyclopropene **1d** (44.4 mg, 0.17 mmol) and **Ir-1** (1.5 mg, 1.0 mol%) in MeCN (0.02 M). After 2 h, analysis of the crude mixture indicated the disappearance of **1d** (NMR yield = 72%, *dr* = 4.5:1). Purification by flash chromatography (deactivated SiO_2, hexanes:EtOAc = 10:1) enabled the isolation of two fractions containing **2d** (F1: 28.2 mg, *dr* = 14:1, colorless oil/ F2: 2.9 mg, colorless oil, minor diastereoisomer. Total: 31.1 mg, 70%, combined yield) as a mixture of diastereoisomers. *Samples of isolated diastereoisomers of **2d** were obtained after a further purification by flash chromatography (deactivated SiO_2, hexanes:EtOAc = 50:1 to 10:1).*

The representative procedure E was followed using cyclopropene **1d** (52.3 mg, 0.20 mmol) and **TXT** (2.0 mg, 5.0 mol%) in MeCN (0.02 M). Under these reaction conditions, **1d** was completely degradated after 0.5 h, the formation of **2d** was not observed.

Characterization data for the mayor isomer (**2d**):

^{1}H-NMR (300 MHz, $CDCl_3$) δ = 7.11 (d, *J* = 8.7 Hz, 1H), 6.82 (d, *J* = 8.6 Hz, 1H), 4.32 (bs, 1H), 3.80 (s, 3H), 3.20 (t_{app}, *J* = 4.0 Hz, 1H), 2.36 – 2.23 (m, 1H), 2.19 – 2.06 (m, 1H), 2.04 – 1.95 (m, 1H), 1.92 – 1.80 (m, 2H), 1.54 (dd, *J* = 5.1, 1.5 Hz, 1H), 1.19 (bs, 1H), 1.13 (s, 3H), 0.83 (s, 3H).

^{13}C-NMR (75 MHz, $CDCl_3$) δ = 157.3, 135.7, 127.0, 113.2, 69.7, 55.2, 45.6, 43.1, 41.5, 39.0, 32.0, 25.6, 23.4, 21.6, 18.7.

HRMS (ESI) m/z: $[M+H]^+$ Calcd for $[C_{17}H_{23}O_2]^+$ 259.1693; found 259.1694.

Characterization data for the minor isomer (**2d'**):

^{1}H-NMR (400 MHz, $CDCl_3$) δ = 7.09 (dd, *J* = 8.8, 1.1 Hz, 2H), 6.82 (d, *J* = 8.6 Hz, 2H), 4.16 (d, *J* = 3.7 Hz, 1H), 3.80 (s, 3H), 3.16 (d, *J* = 4.6 Hz, 1H), 2.43 – 2.13 (m, 3H), 2.09 – 1.87 (m, 2H), 1.35 (dd, *J* = 5.3, 0.9 Hz, 1H), 1.28 (bs, 1H), 1.20 (s, 3H), 0.99 (s, 3H).

^{13}C-NMR (101 MHz, $CDCl_3$) δ = 157.5, 136.1, 127.0, 113.4, 74.2, 55.4, 44.5, 40.8, 39.1, 36.6, 29.6, 28.6, 26.8, 22.4, 20.1.

HRMS (ESI) m/z: $[M+H]^+$ Calcd for $[C_{17}H_{23}O_2]^+$ 259.1693; found 259.1699.

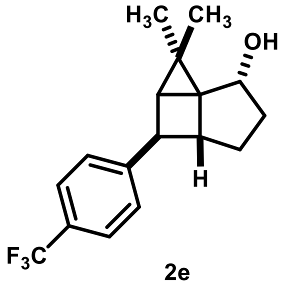

2e

(1*R,3*S**,4*R**,5*R**,8*R**)-2,2-Dimethyl-4-(4-(trifluoromethyl)phenyl)tricyclo[3.3.0.0^{1,3}]octan-8-ol** (**2e**): The representative procedure D was followed using cyclopropene **1e** (29.7 mg, 0.10 mmol) and **Ir-1** (1.0 mg, 1.0 mol%) in MeCN (0.02 M). After 2 h, analysis of the crude mixture indicated the disappearance of **1e** (NMR yield = 72%, *dr* = 4.5:1). Purification by flash chromatography (deactivated SiO_2, hexanes:EtOAc = 10:1) enabled the isolation of two fractions containing **2e** (F1: 17.2 mg, *dr* = 10:1, colorless oil/ F2: 4.0 mg, colorless oil, with a mixture of diastereoisomers. Total: 21.2 mg, 71%, combined yield) as a mixture of diastereoisomers. *A sample of isolated major diastereoisomer of* ***2e*** *was obtained after a further purification by flash chromatography (deactivated SiO_2, hexanes:EtOAc = 50:1 to 10:1).*

The representative procedure E was followed using cyclopropene **1e** (36.2 mg, 0.12 mmol) and **TXT** (1.3 mg, 5.0 mol%) in MeCN (0.02 M). After 2 h, analysis of the crude mixture indicated the disappearance of **1e** (NMR yield = 70%, *dr* = 4.5:1). Purification by flash chromatography (deactivated SiO_2, hexanes:EtOAc = 10:1) enabled the isolation of **2e** (23.5 mg, 65%, colorless oil) as a mixture of diastereoisomers.

Characterization data for the mayor isomer (**2e**):

^{1}H-NMR (300 MHz, $CDCl_3$) δ = 7.52 (d, *J* = 8.0 Hz, 2H), 7.31 (d, *J* = 8.0 Hz, 2H), 4.35 (dd, *J* = 5.1, 2.2 Hz, 1H), 3.26 (t, *J* = 4.3 Hz, 1H), 2.47 – 2.29 (m, 1H), 2.23 – 2.11 (m, 1H), 2.07 – 2.00 (m, 1H), 2.01 – 1.82 (m, 2H), 1.61 (dd, *J* = 5.1, 1.5 Hz, 1H), 1.27 (bs, 1H), 1.15 (s, 3H), 0.78 (s, 3H).

^{13}**C-NMR** (75 MHz, $CDCl_3$) δ = 148.0, 126.3, 127.61 (q, *J* = 32.2 Hz), 124.78 (q, *J* = 3.8 Hz), 120.84 (*d*, *J* = 271.8 Hz, estimated shift value due to the overlapping of signals corresponding to the quartet), 69.6, 45.4, 43.5, 41.6, 39.0, 31.9, 25.7, 23.2, 21.4, 18.7.

^{19}**F NMR** (282 MHz, $CDCl_3$) δ = – 62.1.

HRMS (ESI) m/z: $[M+H]^+$ Calcd for $[C_{17}H_{20}F_3O]^+$ 297.1461; found 297.1459.

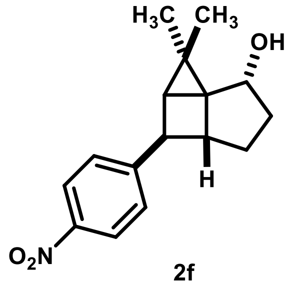

2f

(1*R,3*S**,4*R**,5*R**,8*R**)-2,2-Dimethyl-4-(4-nitrophenyl)tricyclo[3.3.0.0^{1,3}]octan-8-ol** (**2f**): The representative procedure D was followed using cyclopropene **1f** (27.5 mg, 0.10 mmol) and **Ir-1** (1.0 mg, 1.0 mol%) in MeCN (0.02 M). After 2 h, analysis of the crude mixture indicated the disappearance of **1f** (NMR yield = 64%, *dr* = 3:1). Purification by flash chromatography (deactivated SiO_2, hexanes:EtOAc = 5:1 to 3:1) enabled the isolation of two fractions containing **2f** (F1: 12.1 mg, colorless oil, and F2: 4.6 mg, colorless oil; 61%, combined yield) as a mixture of diastereoisomers. *A sample of isolated major diastereoisomer of **2f** was obtained after a further purification by flash chromatography (deactivated SiO_2, hexanes:EtOAc = 10:1 to 3:1).*

The representative procedure E was followed using cyclopropene **1f** (20.0 mg, 0.073 mmol) and **TXT** (1.0 mg, 5.0 mol%) in MeCN (0.02 M). After 2 h, analysis of the crude mixture indicated the disappearance of **1f** (NMR yield = 85%, *dr* = 3:1). Purification by flash chromatography (deactivated SiO_2, hexanes:EtOAc = 10:1 to 3:1) enabled the isolation of **2f** (16 mg, 80%, colorless oil) as a mixture of diastereoisomers.

Characterization data for the mayor isomer (**2f**):

^{1}**H-NMR** (300 MHz, CD_2Cl_2) δ = 8.11 (d, *J* = 8.6 Hz, 2H), 7.38(d, *J* = 8.6 Hz, 2H), 4.32 (s, 1H), 3.29 (t, *J* = 4.2 Hz, 1H), 2.44 – 2.27 (m, 1H), 2.22 –2.19 (m, 1H), 2.03 – 1.83 (m, 2H), 1.62 (d, *J* = 4.3 Hz, 1H, overlapped with residual H_2O), 1.30 (bs, 1H), 1.13 (s, 3H), 0.76 (s, 3H).

^{13}**C-NMR** (75 MHz, CD_2Cl_2) δ = 152.5, 145.9, 126.9, 123.1, 69.3, 45.2, 43.6, 41.7, 38.9, 31.5, 25.6, 23.2, 20.9, 18.4.

HRMS (ESI) m/z: $[M+H]^+$ Calcd for $[C_{16}H_{20}NO_3]^+$ 274.1438; found 274.1435.

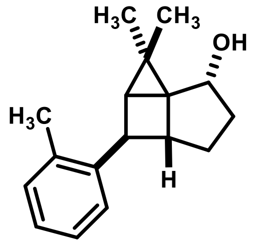

2g

(1*R,3*S**,4*R**,5*R**,8*R**)-2,2-Dimethyl-4-(*o*-tolyl)tricyclo[3.3.0.0^{1,3}]octan-8-ol** (**2g**): The representative procedure D was followed using cyclopropene **1g** (45.8 mg, 0.19 mmol) and **Ir-1** (1.8 mg, 1.0 mol%) in MeCN (0.02 M). After 2.5 h, analysis of the crude mixture indicated the disappearance of **1g** (NMR yield = 80%, *dr* = 4.5:1). Purification by flash chromatography (deactivated SiO_2, hexanes:EtOAc = 10:1) enabled the isolation of both diastereoisomers: **2g** (26.5 mg, 58%, *anti*-OH isomer, *8R**, colorless oil); **2g'** (6.6 mg, 14%, *syn*-OH isomer, *8S**, colorless oil). Combined yield = 72%.

The representative procedure E was followed using cyclopropene **1g** (46.4 mg, 0.19 mmol) and **TXT** (1.8 mg, 5.0 mol%) in MeCN (0.02 M). After 2.5 h, analysis of the crude mixture indicated the disappearance of **1g** (NMR yield = 70%, *dr* = 4:1). Purification by flash chromatography (deactivated SiO_2, hexanes:EtOAc = 10:1) enabled the isolation of both diastereoisomers: **2g** (21.5 mg, 46%, *anti*-OH isomer, *8R**, colorless oil); **2g'** (6.7 mg, 14%, *syn*-OH isomer, *8S**, colorless oil). Combined yield = 60%.

Characterization data for the mayor isomer (**2g**):

^{1}H-NMR (300 MHz, $CDCl_3$) δ = 7.37 – 7.25 (m, 1H), 7.22 – 7.01 (m, 3H), 4.08 – 4.53 (m, 1H), 3.19 (t, *J* = 4.3 Hz, 1H), 2.30 – 2.77 (m, 1H), 2.23 (s, 3H), 2.12 – 1.97 (m, 2H), 1.96 – 1.80 (m, 2H), 1.52 (dd, *J* = 4.9, 1.4 Hz, 1H), 1.28 (bs, 1H), 1.18 (s, 3H), 0.91 (s, 3H).

^{13}C-NMR (75 MHz, $CDCl_3$) δ = 140.4, 135.7, 129.8, 127.5, 125.8, 125.1, 69.9, 45.5, 44.3, 41.7, 39.0, 32.4, 25.5, 23.1, 21.7, 19.9, 18.9.

HRMS (ESI) m/z: $[M+H]^+$ Calcd for $[C_{17}H_{23}O]^+$ 243.1743; found 243.1746.

Characterization data for the minor isomer (**2g'**):

^{1}H-NMR (400 MHz, $CDCl_3$) δ = 7.26 (d, *J* = 8.1 Hz, 1H, overlapped with residual $CHCl_3$), 7.15 – 6.97 (m, 3H), 4.19 (d, *J* = 3.8 Hz, 1H), 3.12 (t, *J* = 4.2 Hz, 1H), 2.43 – 2.26 (m, 3H), 2.22 (s, 3H),2.07 – 1.98 (m, 2H), 1.36 (d, *J* = 5.1 Hz, 1H), 1.24 (s, 3H), 1.04 (s, 3H), (the signal corresponding to the OH could not be allocated).

^{13}C-NMR (101 MHz, $CDCl_3$) δ = 141.0, 135.9, 130.0, 127.5, 125.9, 125.2, 74.5, 44.4, 41.8, 39.0, 36.4, 29.7, 28.4, 26.6, 22.4, 20.5, 20.3.

HRMS (ESI) m/z: $[M+H]^+$ Calcd for $[C_{17}H_{23}O]^+$ 243.1743; found 243.1741.

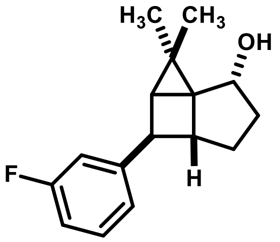

2h

(1*R,3*S**,4*R**,5*R**,8*R**)-2,2-Dimethyl-4-(3-fluorophenyl)tricyclo[3.3.0.0^{1,3}]octan-8-ol** (**2h**): The representative procedure D was followed using cyclopropene **1h** (47.9 mg, 0.19 mmol) and **Ir-1** (1.6 mg, 1.0 mol%) in MeCN (0.02 M). After 2.5 h, analysis of the crude mixture indicated the disappearance of **1h** (NMR yield = 78%, *dr* = 4:1). Purification by flash chromatography (deactivated SiO_2, hexanes:EtOAc = 10:1 to 5:1) enabled the isolation of **2h** (33.7 mg, 70%, colorless oil) as a mixture of diastereoisomers. *Samples of isolated diastereoisomers of* ***2h*** *were obtained after a further purification by flash chromatography (deactivated SiO_2, hexanes:EtOAc = 20:1 to 5:1).*

The representative procedure E was followed using cyclopropene **1h** (47.2 mg, 0.19 mmol) and **TXT** (1.9 mg, 5.0 mol%) in MeCN (0.02 M). After 3 h, analysis of the crude mixture indicated the disappearance of **1h** (NMR yield = 76%, *dr* = 4:1). Purification by flash chromatography (deactivated SiO_2, hexanes:EtOAc = 10:1) enabled the isolation of **2h** (35.5 mg, 74%, colorless oil) as a mixture of diastereoisomers.

Characterization data for the mayor isomer (**2h**):

^{1}H-NMR (400 MHz, $CDCl_3$) δ = 7.20 (td, *J* = 7.9, 6.0 Hz, 1H), 6.95 (d, *J* = 7.6 Hz, 1H), 6.87 (d, *J* = 10.1 Hz, 1H), 6.81 (td, *J* = 8.5, 2.6 Hz, 1H), 4.32 (d, *J* = 4.0 Hz, 1H), 3.20 (t, *J* = 4.2 Hz, 1H), 2.42 – 2.26 (m, 1H), 2.19 – 2.08 (m, 1H), 2.03 – 1.95 – 2.03 (m, 1H), 1.92 – 1.80 (m, 2H), 1.55 (d, *J* = 5.1 Hz, 1H), 1.23 (bs, 1H), 1.12 (s, 3H), 0.80 (s, 3H).

^{13}C-NMR (101 MHz, $CDCl_3$) δ = 163.0 (d, *J* = 245.2 Hz), 146.8 (d, *J* = 6.8 Hz), 129.4 (d, *J* = 8.3 Hz), 121.8 (d, *J* = 2.8 Hz), 113.1 (d, *J* = 20.9 Hz), 112.2 (d, *J* = 21.2 Hz), 69.7, 45.5, 43.6, 41.6, 39.1, 31.9, 25.8, 18.7, 23.4, 21.6.

^{19}F-NMR (282 MHz, $CDCl_3$) δ = –114.22 (td, *J* = 9.3, 5.8 Hz).

HRMS (ESI) m/z: $[M+H]^+$ Calcd for $[C_{16}H_{20}FO]^+$ 247.1493; found 247.1490.

Characterization data for the minor isomer (**2h'**):

^{1}H-NMR (400 MHz, $CDCl_3$) δ = 7.20 (td, *J* = 7.9, 6.0 Hz, 1H), 6.93 (d, *J* = 7.6 Hz, 1H), 6.89 – 6.78 (m, 2H), 4.15 (d, *J* = 4.0 Hz, 1H), 3.15 (t, *J* = 4.6 Hz, 1H), 2.39 (dd, *J* = 8.3, 3.6 Hz, 1H), 2.37 – 2.22 (m, 1H), 2.25 – 2.12 (m, 1H), 2.02 (dd, *J* = 13.1, 7.2 Hz, 1H), 1.94 (dd, *J* = 12.6, 7.4 Hz, 1H), 1.35 (d, *J* = 5.2 Hz, 1H), 1.26 (bs, 1H), 1.18 (s, 3H), 0.95 (s, 3H).

^{13}C-NMR (101 MHz, $CDCl_3$) δ = 162.8 (d, *J* = 244.9 Hz) 146.9 (d, *J* = 6.8 Hz), 129.3 (d, *J* = 8.3 Hz), 121.4 (d, *J* = 2.6 Hz), 112.8 (d, *J* = 20.8 Hz), 112.1 (d, *J* = 21.1 Hz), 73.9, 44.4, 41.1, 39.0, 36.4, 29.3, 28.2, 26.8, 22.1, 19.8.

^{19}F-NMR (282 MHz, $CDCl_3$) δ –114.22 (td, J = 9.5, 6.0 Hz)

HRMS (ESI) m/z: $[M+H]^+$ Calcd for $[C_{16}H_{20}FO]^+$ 247.1493; found 247.1495.

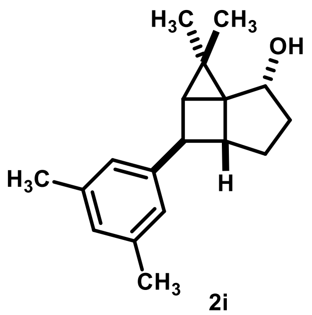

2i

(1*R,3*S**,4*R**,5*R**,8*R**)-4-(3,5-Dimethylphenyl)-2,2-dimethyltricyclo[3.3.0.0^{1,3}]octan-8-ol** (**2i**): The representative procedure D was followed using cyclopropene **1f** (53.0 mg, 0.20 mmol) and **Ir-1** (2.0 mg, 1.0 mol%) in MeCN (0.02 M). After 2 h, analysis of the crude mixture indicated the disappearance of **1i** (NMR yield = 86%, *dr* = 4.5:1). Purification by flash chromatography (deactivated SiO_2, hexanes:EtOAc = 10:1) enabled the isolation of **2i** (34.0 mg, 80%, colorless oil) as a mixture of diastereoisomers. *Samples of isolated diastereoisomers of* ***2i*** *were obtained after a further purification by flash chromatography (deactivated SiO_2, hexanes:EtOAc = 50:1 to 10:1).*

The representative procedure E was followed using cyclopropene **1i** (54.1 mg, 0.21 mmol) and **TXT** (2.2 mg, 5.0 mol%) in MeCN (0.02 M). After 2 h, analysis of the crude mixture indicated the disappearance of **1i** (NMR yield = 65%, *dr* = 5:1). Purification by flash chromatography (deactivated SiO_2, hexanes:EtOAc = 10:1) enabled the isolation of **2i** (33.1 mg, 61%, colorless oil) as a mixture of diastereoisomers.

Characterization data for the mayor isomer (**2i**):

^{1}H-NMR (300 MHz, $CDCl_3$) δ = 6.81 (bs, 3H), 4.33 (dd, *J* = 4.9, 2.2 Hz, 1H), 3.19 (t, *J* = 4.2 Hz, 1H), 2.43 – 2.32 (m, 1H), 2.31 (s, 6H), 2.21 – 2.08 (m, 1H), 2.08 – 1.97 (m, 1H), 1.97 – 1.76 (m, 2H), 1.55 (dd, *J* = 5.1, 1.5 Hz, 1H), 1.28 (bs, 1H), 1.15 (s, 3H), 0.85 (s, 3H).

^{13}C-NMR (75 MHz, $CDCl_3$) δ = 143.6, 137.2, 126.8, 123.8, 69.8, 45.5, 43.7, 41.5, 39.1, 32.1, 25.7, 23.4, 21.6, 21.3, 18.8.

HRMS (ESI) m/z: $[M+H]^+$ Calcd for $[C_{18}H_{25}O]^+$ 257.1900; found 257.1904.

Characterization data for the minor isomer (**2i'**):

^{1}H-NMR (400 MHz, $CDCl_3$) δ = 6.77 (s, 3H), 4.14 (d, *J* = 3.8 Hz, 1H), 3.11 (t, *J* = 4.5 Hz, 1H), 2.37 (dd, *J* = 8.3, 3.6 Hz, 1H), 2.28 (s, 6H, overlapped signal), 2.33 – 2.13 (m, 2H, overlapped signal), 2.01 (dd, *J* = 13.0, 7.1 Hz, 1H), 1.94 (dd, *J* = 12.4, 7.4 Hz, 1H), 1.33 (d, *J* = 5.2 Hz, 1H), 1.18 (s, 3H), 0.97 (s, 3H), (the signal corresponding to the OH could not be allocated).

^{13}C-NMR (101 MHz, $CDCl_3$) δ = 144.0, 137.3, 127.0, 123.8, 74.3, 44.5, 41.4, 39.1, 36.6, 29.6, 28.7, 26.9, 22.4, 21.5, 20.2.

HRMS (ESI) m/z: $[M+H]^+$ Calcd for $[C_{18}H_{25}O]^+$ 257.1900; found 257.1902.

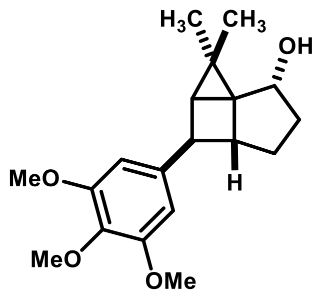

2j

(1*R,3*S**,4*R**,5*R**,8*R**)-2,2-Dimethyl-4-(3,4,5-trimethoxyphenyl)-tricyclo[3.3.0.0^{1,3}]octan-8-ol** (**2j**): The representative procedure D was followed using cyclopropene **1j** (69.4 mg, 0.22 mmol) and **Ir-1** (2.0 mg, 1.0 mol%) in MeCN (0.02 M). After 2.5 h, analysis of the crude mixture indicated the disappearance of **1j** (NMR yield = 75%, *dr* = 5:1). Purification by flash chromatography (deactivated SiO_2, hexanes:EtOAc = 5:1 to 3:1) enabled the isolation of **2j** (46 mg, 66%, colorless oil) as a mixture of diastereoisomers. *A pure sample of major diastereoisomers of **2j** was obtained after a further purification by flash chromatography (deactivated SiO_2, hexanes:EtOAc = 20:1 to 3:1); however, the minor diastereoisomer could not be obtained in a pure form due to degradation during the purification).*

Characterization data for the mayor isomer (**2j**):

^{1}H-NMR (300 MHz, $CDCl_3$) δ = 6.34 (s, 2H), 4.29 (dd, *J* = 4.9, 2.4 Hz, 1H), 3.82 (s, 6H), 3.79 (s, 3H), 3.15 (t, *J* = 4.2 Hz, 1H), 2.36 – 2.24 (m, 1H), 2.12 – 2.04 (m, 1H), 2.02 – 1.76 (m, 3H), 1.49 (dd, *J* = 5.1, 1.3 Hz, 1H), 1.37 (bs, 1H), 1.10 (s, 3H), 0.84 (s, 3H).

^{13}C-NMR (75 MHz, $CDCl_3$) δ = 152.9, 139.6, 135.7, 103.1, 69.6, 61.0, 56.1, 45.1, 44.0, 41.6, 39.0, 31.9, 25.7, 23.3, 21.6, 19.0.

HRMS (ESI) m/z: $[M+H]^+$ Calcd for $[C_{19}H_{27}O_4]^+$ 319.1904; found 319.1902.

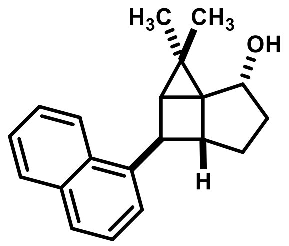

2k

(1*R,3*S**,4*R**,5*R**,8*R**)-2,2-Dimethyl-4-(naphthalene-1-yl)tricyclo[3.3.0.0^{1,3}]octan-8-ol** (**2k**): The representative procedure D was followed using cyclopropene **1k** (70.7 mg, 0.25 mmol) and **Ir-1** (2.3 mg, 1.0 mol%) in MeCN (0.02 M). After 2 h, analysis of the crude mixture indicated the disappearance of **1k** (NMR yield = 65%, *dr* = 4.7:1). Purification by flash chromatography (deactivated SiO_2, hexanes:EtOAc = 20:1) enabled the isolation of both diastereoisomers: **2k** (39.2 mg, 55%, *anti*-OH isomer, *8R**, colorless oil); **2k'** (4.3 mg, 6%, *syn*-OH isomer, *8S**, colorless oil). Combined yield = 61%.

The representative procedure E was followed using cyclopropene **1k** (28.4 mg, 0.10 mmol) and **TXT** (1.1 mg, 5.0 mol%) in MeCN (0.02 M). After 3 h, analysis of the crude mixture indicated the disappearance of **1k** (NMR yield = 64%, *dr* = 4:1). Purification by flash chromatography (deactivated SiO_2, hexanes:EtOAc = 10:1) enabled the isolation of **2k** (16.5 mg, 58%, colorless oil) as a mixture of diastereoisomers.

Characterization data for the mayor isomer (**2k**):

^{1}H-NMR (300 MHz, $CDCl_3$) δ = 7.94 – 7.86 (m, 2H), 7.72 (d, *J* = 7.9 Hz, 1H), 7.58 – 7.37 (m, 4H), 4.41 (d, *J* = 5.4 Hz, 1H), 3.68 (t, *J* = 4.2 Hz, 1H), 2.74 – 2.52 (m, 1H), 2.42 – 2.23 (m, 1H), 2.21 – 2.09 (m, 2H), 2.04 – 1.85 (m, 1H), 1.69 (d, *J* = 5.4 Hz, 1H), 1.34 (bs, 1H), 1.24 (s, 3H), 0.92 (s, 3H).

^{13}C-NMR (75 MHz, $CDCl_3$) δ = 138.5, 133.7, 131.5, 128.8, 126.5, 125.6, 125.4, 125.3, 125.2, 124.3, 70.1, 46.2, 43.8, 42.5, 39.2, 33.0, 25.8, 23.1, 21.9, 19.4.

HRMS (ESI) m/z: $[M+H]^+$ Calcd for $[C_{20}H_{23}O]^+$ 279.1743; found 279.1744.

Characterization data for the minor isomer (**2k'**):

^{1}H-NMR (400 MHz, $CDCl_3$) δ = 7.89 (d, *J* = 8.2 Hz, 1H), 7.85 (d, *J* = 7.6 Hz, 1H), 7.68 (d, *J* = 7.6 Hz, 1H), 7.55 – 7.36 (m, 4H), 4.25 (d, *J* = 3.6 Hz, 1H), 3.60 (bs, 1H), 2.59 – 2.41 (m, 1H), 2.30 – 2.25 (m, 2H), 2.21 – 2.01 (m, 1H), 1.51 (d, *J* = 5.2 Hz, 1H), 1.27 (s, 3H), 1.02 (s, 3H) (the signal corresponding to the OH could not be allocated).

^{13}C-NMR (101 MHz, $CDCl_3$) δ = 139.2, 133.9, 131.6, 128.9, 126.5, 125.6, 125.4, 125.3, 125.2, 124.8, 74.5, 44.9, 41.2, 39.6, 36.5, 30.2, 28.5, 26.8, 22.5, 20.6.

HRMS (ESI) m/z: $[M+H]^+$ Calcd for $[C_{20}H_{23}O]^+$ 279.1743; found 279.1741.

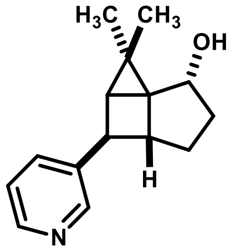

2l

(1*R,3*S**,4*R**,5*R**,8*R**)-2,2-Dimethyl-4-(pyridine-3-yl)tricyclo[3.3.0.0^{1,3}]octan-8-ol** (**2l**): The representative procedure D was followed using cyclopropene **1l** (23.5 mg, 0.10 mmol) and **Ir-1** (1.1 mg, 1.0 mol%) in MeCN (0.02 M). After 2 h, analysis of the crude mixture indicated the disappearance of **1l** (NMR yield = 80%, *dr* = 3:1). Purification by flash chromatography (deactivated SiO_2, EtOAc:MeOH = 20:1) enabled the isolation of **2l** (39.2 mg, 65%, colorless oil) as a mixture of diastereoisomers. *Attempts to obtain pure diastereoisomers were unsuccessful.*

The representative procedure E was followed using cyclopropene **1l** (23.5 mg, 0.10 mmol) and **TXT** (1.0 mg, 5.0 mol%) in MeCN (0.02 M). Under these reaction conditions, **1l** was completely degradated after 0.5 h, the formation of **2l** was not observed.

Characterization data for the mayor isomer (**2l**, * denotes estimated integration due to overlapping signals from the minor diastereoisomer **2l'**):

^{1}H-NMR (400 MHz, $CDCl_3$) δ = 0.80 (s, 3H), 1.14 (s, 3H), 1.61 (dd, *J* = 5.1, 1.4 Hz, 1H), 1.82 – 2.23 (m, 4H*), 2.29 – 2.45 (m, 1H*), 3.25 (t, *J* = 4.2 Hz, 1H), 4.35 (d, *J* = 4.5 Hz, 1H), 7.19 (dd, J = 7.8, 4.8 Hz, 1H*), 7.42 – 7.61 (m, 1H*), 8.39 (d, J = 4.8 Hz, 1H*), 8.45 (bs, 1H*) (the signal corresponding to the OH could not be allocated). Data for the minor isomer (**2l'**, only clearly assignable signals are given): δ = 0.95 (s, 3H), 1.19 (s, 3H), 1.39 (d, *J* = 5.6 Hz, 1H), 3.19 (t, *J* = 4.5 Hz, 1H), 4.17 (d, *J* = 4.0 Hz, 1H).

^{13}**C-NMR** (101 MHz, $CDCl_3$, only signals corresponding to the major diastereoisomer are indicated) δ = 147.9, 146.7, 138.9, 133.7, 122.8, 69.5, 45.7, 41.3, 38.9, 31.8, 25.7, 23.0, 21.4, 18.8 (signal at 41.3 ppm belong to two C atom signals).

HRMS (ESI) m/z: $[M+H]^+$ Calcd for $[C_{15}H_{20}NO]^+$ 230.1539; found 230.1535 (for the mixture of diastereoisomers).

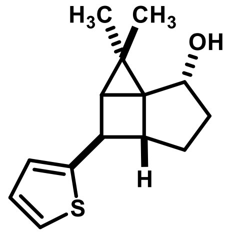

2m

(1*R,3*S**,4*R**,5*R**,8*R**)-2,2-Dimethyl-4-(thiophen-2-yltricyclo[3.3.0.0^{1,3}]octan-8-ol** (**2m**): The representative procedure D was followed using cyclopropene **1m** (47.9 mg, 0.20 mmol) and **Ir-1** (2.4 mg, 1.0 mol%) in MeCN (0.02 M). After 2 h, analysis of the crude mixture indicated the disappearance of **1m** (NMR yield = 78%, *dr* = 4.8:1). Purification by flash chromatography (deactivated SiO_2, hexanes:EtOAc = 10:1) enabled the isolation of **2m** (33.5 mg, 71%, colorless oil) as a mixture of diastereoisomers. *Samples of isolated diastereoisomers of* ***2m*** *were obtained after a further purification by flash chromatography (deactivated* SiO_2*, hexanes:EtOAc = 50:1 to 10:1).*

The representative procedure E was followed using cyclopropene **1m** (48.5 mg, 0.21 mmol) and **TXT** (2.1 mg, 5.0 mol%) in MeCN (0.02 M). After 3 h, analysis of the crude mixture indicated the disappearance of **1m** (NMR yield = 53%, *dr* = 4.3:1). Purification by flash chromatography (deactivated SiO_2, hexanes:EtOAc = 10:1) enabled the isolation of **2m** (24.9 mg, 51%, colorless oil) as a mixture of diastereoisomers.

Characterization data for the mayor isomer (**2m**):

^{1}**H-NMR** (300 MHz, $CDCl_3$) δ = 7.12 (dt, *J* = 5.0, 1.1 Hz, 1H), 6.90 (dd, *J* = 5.1, 3.4 Hz, 1H), 6.75 (dt, *J* = 3.3, 1.4 Hz, 1H), 4.33 (dd, *J* = 5.2, 2.6 Hz, 1H), 3.35 (bs, 1H), 2.36 – 2.26 (m, 1H), 2.24 – 2.11 (m, 1H), 1.75 – 2.06 (m, 3H), 1.57 (dd, *J* = 5.0, 1.4 Hz, 1H), 1.22 (bs, 1H), 1.12 (s, 3H), 1.00 (s, 3H).

^{13}**C-NMR** (75 MHz, $CDCl_3$) δ = 147.9, 126.5, 123.1, 122.8, 69.7, 45.9, 42.3, 39.9, 38.8, 31.5, 25.9, 24.5, 21.5, 18.5.

HRMS (ESI) m/z: $[M+H]^+$ Calcd for $[C_{14}H_{19}OS]^+$ 235.1151; found 235.1154.

Characterization data for the minor isomer (**2m'**):

^{1}H-NMR (400 MHz, $CDCl_3$) δ = 7.12 (dt, *J* = 5.0, 1.1 Hz, 1H), 6.90 (dd, *J* = 5.1, 3.4 Hz, 1H), 6.74 (dd, *J* = 3.2, 1.6 Hz, 1H), 4.15 (d, *J* = 4.1 Hz, 1H), 3.31 (t, *J* = 4.5 Hz, 1H), 2.46 (dd, *J* = 8.2, 3.7 Hz, 1H), 2.33 – 2.11 (m, 2H), 2.07 – 1.89 (m, 2H), 1.38 (dd, *J* = 5.3, 1.0 Hz, 1H), 1.18 (s, 3H), 1.15 (s, 3H), (the signal corresponding to the OH could not be allocated).

^{13}C-NMR (101 MHz, $CDCl_3$) δ = 148.1, 143.7, 126.4, 123.0, 122.8, 73.9, 45.0, 39.9, 37.7, 36.4, 29.4, 29.0, 27.0, 22.2, 19.8.

HRMS (ESI) m/z: $[M+H]^+$ Calcd for $[C_{14}H_{19}OS]^+$ 235.1151; found 235.1161.

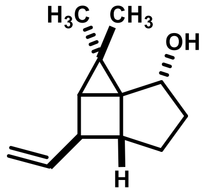

2n

(1*R,3*S**,4*R**,5*R**,8*R**)-2,2-Dimethyl-4-vinyltricyclo[3.3.0.0^{1,3}]octan-8-ol** (**2n**): The representative procedure D was followed using cyclopropene **1n** (34.4 mg, 0.19 mmol) and **Ir-1** (1.9 mg, 1.0 mol%) in MeCN (0.02 M). After 4 h, analysis of the crude mixture indicated the disappearance of **1n** (NMR yield = 68%, *dr* = 6:1, the formation of other side products was detected in the reaction crude mixture). Purification by flash chromatography (deactivated SiO_2, hexanes:EtOAc = 10:1) enabled the isolation of **2n** (21.0 mg, 61%, colorless oil) as a mixture of diastereoisomers. *Samples of isolated diastereoisomers of **2n** were obtained after a further purification by flash chromatography (deactivated SiO_2, hexanes:EtOAc = 50:1 to 10:1).*

The representative procedure E was followed using cyclopropene **1n** (22.6 mg, 0.12 mmol) and **TXT** (1.2 mg, 5.0 mol%) in MeCN (0.02 M). After 4 h, analysis of the crude mixture indicated the disappearance of **1n** (NMR yield = 56%, *dr* = 4.3:1, the formation of other side products was detected in the reaction crude mixture). Purification by flash chromatography (deactivated SiO_2, hexanes:EtOAc = 10:1) enabled the isolation of **2n** (11.3 mg, 50%, colorless oil) as a mixture of diastereoisomers.

Characterization data for the mayor isomer (**2n**):

^{1}H-NMR (300 MHz, $CDCl_3$) δ = 5.91 (ddd, *J* = 17.4, 10.4, 4.9 Hz, 1H), 5.03 – 4.90 (m, 2H), 4.25 (dd, *J* = 5.6, 2.4 Hz, 1H), 2.54 (bs, 1H), 2.29 – 2.07 (m, 1H), 2.00 – 1.86, 1.84 – 1.71 (m, 1H), 1.57 – 1.71 (m, 1H), 1.24 (d, *J* = 5.2 Hz, 1H), 1.18 (s, 3H), 1.10 (bs, 1H), 1.06 (s, 3H).

^{13}C-NMR (101 MHz, $CDCl_3$) δ = 140.0, 113.9, 69.9, 45.9, 43.6, 40.2, 39.0, 31.8, 25.8, 24.1, 21.8, 19.0.

HRMS (ESI) m/z: $[M+H]^+$ Calcd for $[C_{12}H_{19}O]^+$ 179.1430; found 179.1428.

Characterization data for the minor isomer (**2n'**):

^{1}H-NMR (400 MHz, $CDCl_3$) δ = 5.97 (ddd, *J* = 16.6, 10.5, 5.6 Hz, 1H), 5.11 – 5.00 (m, 2H), 4.15 (bs, 1H), 2.40 (t, *J* = 5.6 Hz, 1H), 1.95 (ddd, *J* = 7.6, 5.6, 1.9 Hz, 1H), 1.89 – 1.78 (m, 1H), 1.77 – 1.63 (m, 3H), 1.23 (bs, 2H), 1.20 (s, 3H), 1.09 (s, 3H).

^{13}C-NMR (101 MHz, $CDCl_3$) δ = 138.4, 114.4, 69.7, 47.5, 38.8, 37.9, 34.9, 26.1, 25.5, 24.8, 21.1, 15.3.

HRMS (ESI) m/z: $[M+H]^+$ Calcd for $[C_{12}H_{19}O]^+$ 179.1430; found 179.1427.

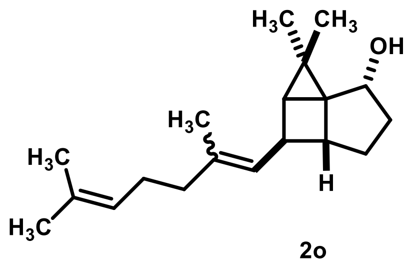

2o

(1*R,3*S**,4*R**,5*R**,8*R**)-4-(2,6-Dimethylhepta-1,5-dien-1-yl)-2,2-dimethyltricyclo[3.3.0.0^{1,3}]octan-8-ol** (**2o**): The representative procedure D was followed using cyclopropene **1o** (54.3 mg, 0.20 mmol) and **Ir-1** (1.8 mg, 1.0 mol%) in MeCN (0.02 M). After 3 h, analysis of the crude mixture indicated the disappearance of **1o** (NMR yield = 82%, *dr* = 4.2:1, estimated value due to the overlapping of signals and the presence of side products in the reaction crude mixture). Purification by flash chromatography (deactivated SiO_2, hexanes:EtOAc = 30:1 to 10:1) enabled the isolation of four different fractions: (43.4 mg, 80% combined yield):

Fraction-1: 10.4 mg, colorless oil; (contains an unknown side product in 7:1 ratio).

Fraction-2: 14.1 mg, colorless oil; estimated *dr* = 1:1 (diastereoisomers from F1 and F3).

Fraction-3: 11.0 mg, colorless oil (pure diastereoisomer).

Fraction-4: 7.9 mg, colorless oil; estimated *dr* = 2:1 *(also contains an inseparable unknown impurity)*.

The representative procedure E was followed using cyclopropene **1o** (57.6 mg, 0.21 mmol) and **TXT** (2.0 mg, 5.0 mol%) in MeCN (0.02 M). After 3 h, analysis of the crude mixture indicated the disappearance of **1o** (NMR yield = 75%, *dr* = 4:1, estimated value due to the overlapping of

signals and the presence of side products in the reaction crude mixture). Purification by flash chromatography (deactivated SiO_2, hexanes:EtOAc = 30:1) enabled the isolation of four different fractions (42.0 mg, 73%, combined yield, colorless oil) as a mixture of diastereoisomers.

Characterization data for the mayor diastereoisomer from F1 (**2o**-*F1*):

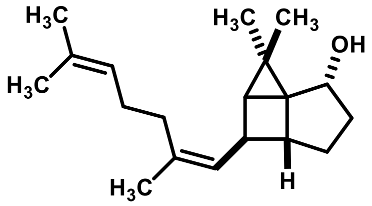

2o-*F1*

^{1}H-NMR (400 MHz, $CDCl_3$) δ = 5.30 (dd, *J* = 6.4, 1.4 Hz, 1H), 5.19 – 5.13 (m, 1H), 4.25 (t, *J* = 4.9 Hz, 1H), 2.65 (bs, 1H), 2.26 – 2.13 (m, 1H), 2.09 – 1.89 (m, 5H), 1.83 – 1.73 (m, 3H), 1.73 – 1.66 (m, 4H), 1.66 (t, *J* = 1.4 Hz, 3H), 1.63 (s, 3H), 1.26 (s, 3H), 1.24 (bs, 1H, overlapped signal), 1.06 (s, 3H).

^{13}C-NMR (101 MHz, $CDCl_3$) δ = 135.8, 131.4, 127.3, 124.3, 69.9, 46.0, 42.7, 39.0, 38.9, 32.3, 32.1, 26.4, 25.7, 25.5, 25.4, 23.1, 21.6, 18.7, 17.7.

HRMS (ESI) m/z: $[M+H]^+$ Calcd for $[C_{19}H_{31}O]^+$ 275.2369; found 275.2367.

Characterization data for F3 (**2o**-*F3*):

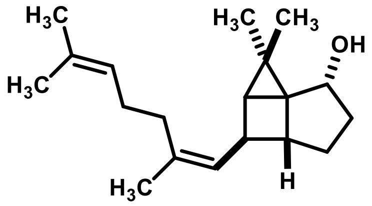

2o-*F3*

^{1}H-NMR (400 MHz, $CDCl_3$) δ = 5.27 (dq, *J* = 5.7, 1.4 Hz, 1H), 5.10 – 5.05 (m, 1H), 4.25 (s, 1H), 2.63 (s, 1H), 2.33 – 2.15 (m, 1H), 2.12 – 2.01 (m, 2H), 1.99 – 1.89 (m, 3H), 1.82 – 1.69 (m, 4H, overlapped), 1.68 (s, 3H, overlapped), 1.61 (s, 3H), 1.56 (s, 3H), 1.27 (dd, *J* = 4.8, 1.4 Hz, 1H), 1.24 (s, 3H), 1.06 (s, 3H).

^{13}C-NMR (75 MHz, $CDCl_3$) δ = 135.2, 131.3, 126.7, 124.4, 69.9, 46.1, 42.7, 39.4, 39.2, 38.9, 32.2, 26.5, 25.7, 25.4, 25.1, 21.6, 18.5, 17.7, 16.4.

HRMS (ESI) m/z: $[M+H]^+$ Calcd for $[C_{19}H_{31}O]^+$ 275.2369; found 275.2368.

Characterization data for F4 (**2o**-*F4*):

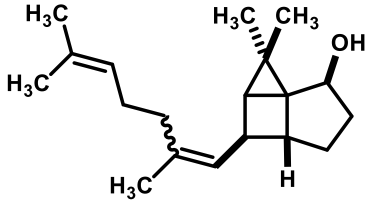

2o-*F4*

^{1}H-NMR (400 MHz, $CDCl_3$, * indicates overlapping of both diastereoisomers, *M*: major diastereoisomer; *m*: minor diastereoisomer) δ = 5.26 (d, J = 6.5 Hz, 1H, *m*), 5.23 (d, J = 5.9 Hz, 1H, *M*), 5.17 – 4.99 (m, 2H*), 4.05 (bs, 2H*), 2.59 (s, 2H*), 2.17 – 1.87 (m, 16H*), 1.86 – 1.71 (m, 6H*), 1.69 (s, 3H, *m*), 1.68 (s, 3H, *M*), 1.62 (s, 3H, *m*), 1.60 (s, 3H, *M*), 1.57 (s, 3H, *m*), 1.55 (s, 3H, *M*), 1.38 (s, 3H, *m*), 1.36 (s, 3H, *M*), 1.12 (s, 6H*).

^{13}C-NMR (101 MHz, $CDCl_3$) δ = 135.7, 135.1, 131.4, 131.3, 127.7, 127.2, 124.4, 74.3, 74.3, 44.6, 39.8, 39.7, 39.4, 37.0, 36.6, 36.3, 36.3, 32.4, 30.7, 30.4, 29.4, 29.2, 26.6, 26.5, 26.5, 25.7, 25.7, 23.1, 22.3, 22.2, 19.9, 19.7, 17.7, 16.6.

HRMS (ESI) m/z: $[M+H]^+$ Calcd for $[C_{19}H_{31}O]^+$ 275.2369; found 275.2368.

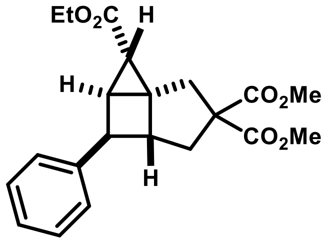

2p

2-Ethyl 7,7-dimethyl (1*R,2*S**,3*S**,4*R**,5*R**)-4-phenyltricyclo[3.3.0.01,3]octane-2,7,7-tricarboxylate** (**2p**): The representative procedure D was followed using cyclopropene **1p** (36.5 mg, 0.10 mmol) and **Ir-1** (1.0 mg, 1.0 mol%) in MeCN (0.02 M). After 0.5 h, analysis of the crude mixture indicated the disappearance of **1p** (estimated NMR yield = 65%, *dr* = 6:1, the formation of other side products was detected in the reaction crude mixture). Purification by flash chromatography (deactivated SiO_2, hexanes:EtOAc = 10:1) enabled the isolation of **2p** (21.9 mg, 61%, pale yellow oil) as a mixture of diastereoisomers. *A pure sample of the major diastereoisomer of* ***2p*** *could not be obtained. However, an enriched sample of the major diastereoisomer of* ***2p*** *was obtained after a further purification by flash chromatography (deactivated* SiO_2*, hexanes:EtOAc = 50:1 to 10:1).*

The representative procedure E was followed using cyclopropene **1p** (36.0 mg, 0.10 mmol) and **TXT** (1.0 mg, 5.0 mol%) in MeCN (0.02 M). After 0.5 h, analysis of the crude mixture indicated

the disappearance of **1p** (estimated NMR yield = 59%, *dr* = 6:1, the formation of other side products was detected in the reaction crude mixture). Purification by flash chromatography (deactivated SiO_2, hexanes:EtOAc = 10:1) enabled the isolation of **2p** (18.1 mg, 50%, pale yellow oil) as a mixture of diastereoisomers.

Characterization data for the mayor isomer (**2p**):

^{1}H-NMR (300 MHz, $CDCl_3$) δ = 7.35 – 7.26 (m, 2H), 7.20 (d, *J* = 7.8 Hz, 1H), 7.15 – 7.08 (m, 2H), 4.13 (q, *J* = 7.1 Hz, 2H), 3.82 (s, 3H), 3.77 (s, 3H), 3.47 (bs, 1H), 2.80 – 2.60 (m, 4H), 2.37 – 2.24 (m, 1H), 2.20 (d, *J* = 4.4 Hz, 1H), 2.12 (s, 1H), 1.27 (t, *J* = 7.1 Hz, 3H).

^{13}C-NMR (75 MHz, $CDCl_3$) δ = 172.2, 172.2, 171.8, 140.8, 128.3, 126.7, 125.9, 62.9, 60.5, 52.9, 52.9, 44.4, 43.1, 40.4, 38.5, 33.5, 29.2, 28.1, 14.2.

HRMS (ESI) m/z: $[M+H]^+$ Calcd for $[C_{21}H_{25}O_6]^+$ 373.1646; found 373.1648 (for mixture of diastereoisomers).

5. Proposed mechanism.

5.1. Mechanistic experiments.

Light on-off experiment.

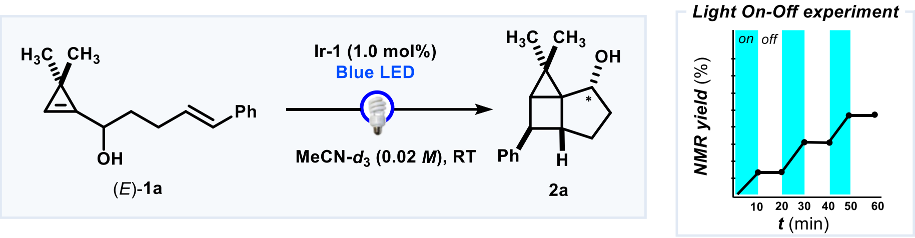

The representative procedure D was followed using cyclopropene **1a** (30.0 mg, 0.13 mmol) and **Ir-1** (1.0 mg, 1.0 mol%) in MeCN-d_3 (0.02 M) using hexamethylcyclotrisiloxane as internal standard. Light on-off cycles of 10 minutes each were perfomed and 0.25 mL samples of the reaction crude mixture were taken every 10 minutes for ^{1}H-NMR analysis. The samples were protected from light until measured.

Styrene excitation probe.

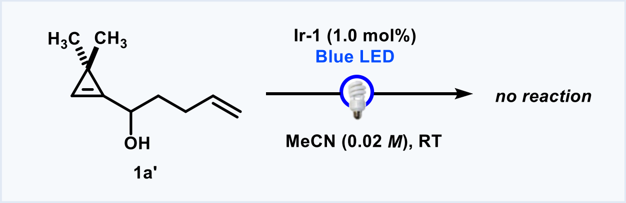

The representative procedure D was followed using cyclopropene **1a’** (41.0 mg, 0.27 mmol) and **Ir-1** (2.5 mg, 1.0 mol%) in MeCN (0.02 M). After 24 h, TLC and ^{1}H NMR analysis of the reaction crude miture showed only the presence of **1a’** (>90% NMR).

Indepedency of the alkene stereochemistry.

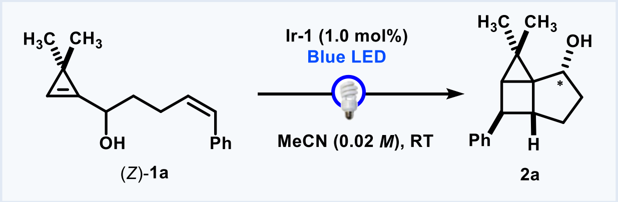

The representative procedure D was followed using cyclopropene (*Z*)-**1a** (23.3 mg, 0.10 mmol) and **Ir-1** (1.0 mg, 1.0 mol%) in MeCN (0.02 M). After 2 h, analysis of the crude mixture indicated the disappearance of **1a** and the formation of **2a** (NMR yield = 68%, *dr* = 4.6:1). Purification by flash chromatography (deactivated SiO_2, hexanes:EtOAc = 10:1) enabled the isolation of **2a** (15.4 mg, 65%) as a mixture of diastereoisomers.

Cyclic voltammetry of 1a.

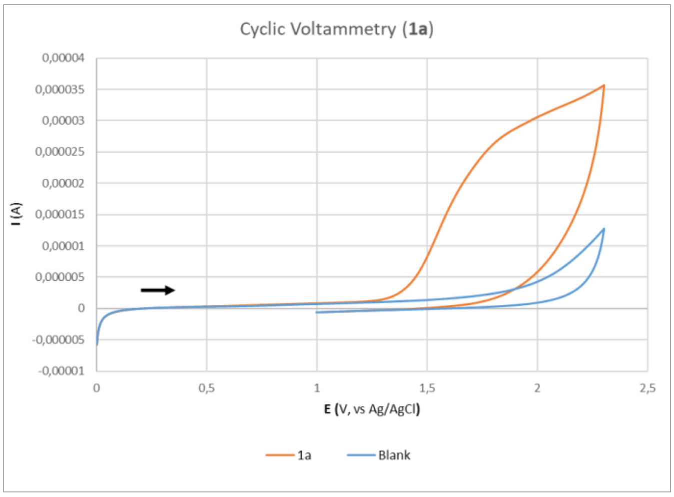

Cyclic voltammograms (CV, IUPAC plotting convention) were accomplished at ambient temperature using a Eco Chemie BV Metrohm FRA2 Autolab Potentiostat/Galvanostat. A glassy carbon disc electrode[27] (1.5 mm of radius, circular geometry, 7.07 mm^2 working electrode surface), an Ag/AgCl reference electrode and a platinum wire counter electrode were used. The electrolyte solution contained 1.0 mL of a 0.1 M tetrabutylammonium hexafluorophosphate and 0.150 μL of a 0.131 M solution of **1a** in MeCN (final concentration of **1a**: 3.82 mM). The scan rate was set at 50 mV/s in a potential window of 0.0 V (starting potential) to + 2.30 V (ending potential).

The value obtained for E (**1a**$^+$/**1a**) is +1.80 V (vs Ag/AgCl) (which corresponds to +1.85 vs SCE).

[27] Polishing procedure: Hold the glassy carbon electrode at a right angle to the alumina polishing pad. Apply a small amount of polishing alumina (3 µm) and polish the electrode in a figure-eight motion for 2 minutes. Then, switch to a rough polishing pad (35 µm particle size), moisten it with water, and continue polishing in the same figure-eight motion for an additional 2 minutes. Finally, thoroughly rinse the electrode surface with distilled water to remove any remaining residues.

5.2. Computational studies.

Computational details.

All calculations were carried out using the Gaussian 16 program package.[28] In view of the large molecules investigated experimentally, the molecular geometries of the S_0 and/or T_1 states were fully optimized without any molecular symmetry constraints for all model compounds using the B3LYP functional,[29] including the dispersion effect by means of the Grimme's dispersion with the original D3 damping function,[30] in combination with the 6-31G(d,p) basis set[31] for the rest of the atoms. A similar level of theory demostrated to be useful in the elucidation of reaction mechanisms of light induced [2+2] cycloadditons.[32] The optimized structures were characterized as minima or saddle points by frequency calculations, which also provide thermochemical analysis of the system for $T = 298.15$ K. In order to fully prove the relevance of the transition structures we also computed the IRC (intrinsic reaction coordinate)[33] connecting the critical points to confirm that the TS really relate the minima. The potential energy surface crossing between T_1 and S_0 states was calculated by MECP (mínimum energy crossing point) using a modified version of Harvey's MECP program.[34] Finally, the effects of solvation were taken into account by performing puntual calculations on the optimized structures including the Polarizable Continuum Model (PCM),[35] using the integral equation formalism variant and the dielectric constant of acetonitrile ($\varepsilon = 35.688$).

[28] Gaussian 16, Revision C.01, Gaussian, Inc., Wallingford CT, 2019.

[29] (a) Becke, A. D. Density-functional Thermochemistry. III. The Role of Exact Exchange. *J. Chem. Phys.* **1993**, *98*, 5648–5652. (b) Stephens, P. J.; Devlin, F. J.; Chabalowski, C. F.; Frisch, M. J. Ab Initio Calculation of Vibrational Absorption and Circular Dichroism Spectra Using Density Functional Force Fields. *J. Phys. Chem.* **1994**, *98*, 11623–11627.

[30] Grimme, S.; Antony, J.; Ehrlich, S.; Krieg, H. A Consistent and Accurate Ab Initio Parametrization of Density Functional Dispersion Correction (DFT-D) for the 94 Elements H-Pu. *J. Chem. Phys.* **2010**, *132*, 154104.

[31] (a) Ditchfield, R.; Hehre, W. J.; Pople, J. A. Self-Consistent Molecular-Orbital Methods. IX. An Extended Gaussian-Type Basis for Molecular-Orbital Studies of Organic Molecules. *J. Chem. Phys.* **1971**, *54*, 724–728. (b) Hehre, W. J.; Ditchfield, K.; Pople, J. A. Self—Consistent Molecular Orbital Methods. XII. Further Extensions of Gaussian—Type Basis Sets for Use in Molecular Orbital Studies of Organic Molecules. *J. Chem. Phys.* **1972**, *56*, 2257–2261. (c) Hariharan, P. C.; Pople, J. A. The Influence of Polarization Functions on Molecular Orbital Hydrogenation Energies. *Theor. Chim. Acta* **1973**, *28*, 213–222.

[32] Jiao, M.; Ju, Y. wen; Chen, B. Z. Energy Transfer or Electron Transfer?—DFT Study on the Mechanism of [2+2] Cycloadditions Induced by Visible Light Photocatalysts. *Tetrahedron Lett.* **2018**, *59*, 1651–1660.

[33] (a) Truhlar, D. G.; Kuppermann, A. Exact Tunneling Calculations. *J. Am. Chem. Soc.* **1971**, *93*, 1840–1851. (b) Fukui, K. The path of chemical reactions - the IRC approach. *Acc. Chem. Res.* 1981, **14**, 363–368.

[34] (a) Harvey, J. N.; Aschi, M. Spin-Forbidden Dehydrogenation of Methoxy Cation: A Statistical View. *Phys. Chem. Chem. Phys.* **1999**, *1*, 5555–5563. (b) Harvey, J. N.; Aschi, M.; Schwarz, H.; Koch, W. The Singlet and Triplet States of Phenyl Cation. A Hybrid Approach for Locating Minimum Energy Crossing Points between Non-Interacting Potential Energy Surfaces. *Theor. Chem. Acc.* **1998**, *99*, 95–99.

[35] Tomasi, J.; Mennucci, B.; Cammi, R. Quantum Mechanical Continuum Solvation Models. *Chem. Rev.* **2005**, *105*, 2999–3093.

Proposed mechanism.

Our calculations indicate that, after sensitization with the appropriate photocatalyst, **1a** leads to the triplet state **^{3}A**, whose relaxed structure lies +48.7 kcal/mol higher in energy. The Mulliken density analysis and bond distances are consistent with sensitized excitation of the styryl fragment instead of cyclopropene. From **^{3}A** onwards, two possible diastereomeric transition states can be reached by overcoming free energy barriers of +8.3 kcal/mol [**$TS_{A–B}(OH_{eq})$**] or +10.4 kcal/mol [**$TS_{A–B}(OH_{ax})$**]. **$TS_{A–B}(OH_{eq})$** evolves to intermediate **^{3}B** (+25.1 kcal/mol above **1a**) by formation of a new C–C bond. However, the following step from **^{3}B** to **^{3}C** on the triplet state PES via **$TS_{B–C}$** has a high free energy barrier of 41.6 kcal/mol, which prevents the reaction from proceeding. In addition, **^{3}C** must be +7.8 kcal/mol more energetic than **^{3}A**. Instead, a potential energy surface crossing must occur between T_1 and S_0 states at the MECP point, which has an energy +8.7 kcal/mol higher than that of **^{3}B**, from which the more stable housane **2a** (-23.1 kcal/mol) is formed.

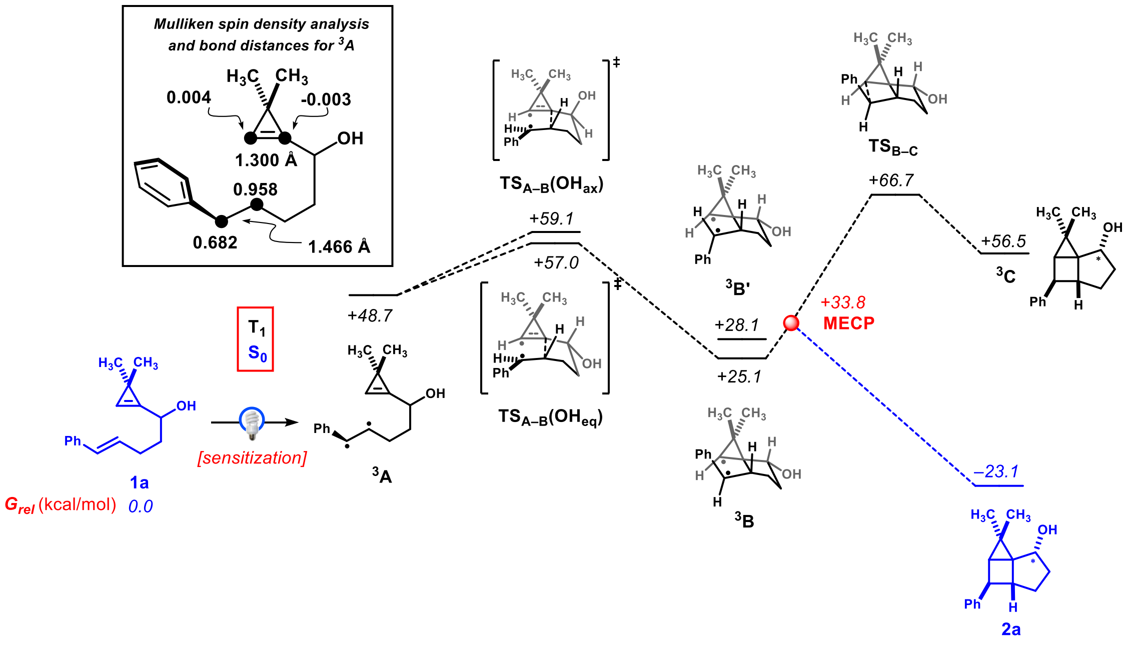

Scheme S1. Proposed mechanism.

Moreover, an alternative more energetic reaction path is calculated from **3A'** (+49.5 kcal/mol above **1a**), a conformer of **3A**. Rotation around the C–C bond of the biradical interconverts the **3A** and **3A'** conformations by overcoming the **$TS_{A–A'}$** transition state (+56.9 kcal/mol). Structure **3A'** evolves through **$TS_{A'–B'}$(OHeq)** (+61.0 kcal/mol) to **3B'** (+28.1 kcal/mol), in accordance with the intrinsic reaction coordinate (IRC) calculation. From **3B'** the potential energy surface crossing between **T1** and **S0** states at **MECP'** (+38.4 kcal/mol) gives diastereoisomer **2a'** (-21.9 kcal/mol).

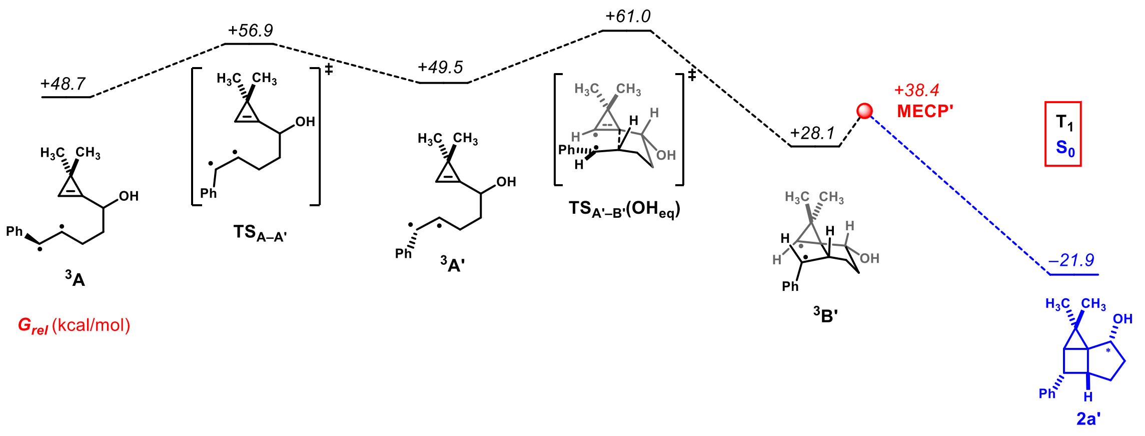

Scheme S2. Alternative more energy demanding reaction pathway.

Cartesian coordinates.

Symbolic Z-Matrix, Absolute Free-Energies, in atomic units, and Number of Imaginary Frequencies [PCM(acetonitrile)-B3LYP/6-31G**//B3LYP/6-31G** level]

Model compound 1a (S_0)

C	2.34039300	1.73897200	-0.12198400
C	2.09534200	0.25093700	-0.09210400
C	1.12408500	0.96349300	-0.58089400
C	2.28065300	2.54921900	1.17048500
C	3.31306700	2.31539700	-1.14595100
C	2.71618900	-1.05454800	0.25196300
H	0.12639400	1.00550300	-0.99342500
H	1.94709800	3.57663600	0.97624600
H	1.58887000	2.09959400	1.89028500
H	3.26924400	2.61329000	1.64249400
H	4.33039100	2.34946000	-0.73804300
H	3.33618700	1.70473000	-2.05242900
H	3.03359100	3.33976800	-1.42449400
C	0.69634900	-2.67891400	0.57002300
H	0.26862700	-3.58608800	0.12724900
H	1.03954400	-2.95243500	1.57827400
C	-0.36252600	-1.61826600	0.67327100
H	-0.12810900	-0.77608700	1.31870300
C	-1.51866300	-1.63079000	-0.00510500
H	-1.73426700	-2.49426200	-0.63595400
C	-2.55419400	-0.58653400	-0.00391400
C	-3.78712800	-0.84500200	-0.62836800
C	-2.36584500	0.68112200	0.58116000
C	-4.79802700	0.11420600	-0.65877000
H	-3.95145800	-1.81442000	-1.09232300
C	-3.37467100	1.63964100	0.55030500
H	-1.41836000	0.92393900	1.05146500
C	-4.59736000	1.36212700	-0.06778800
H	-5.74198700	-0.11298700	-1.14592300
H	-3.20456800	2.61115600	1.00567700
H	-5.38128300	2.11315100	-0.09227200

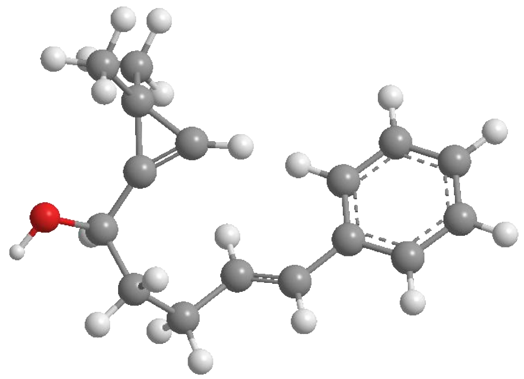

H	2.78181600	-1.12018300	1.35464900
O	4.03300700	-1.00650100	-0.31100300
H	4.50215900	-1.79960300	-0.01932100
C	1.92907100	-2.27190400	-0.27024500
H	2.61918000	-3.12635800	-0.28900300
H	1.63246200	-2.07773500	-1.30632600

Absolute Free Energy: -696.662151 u.a.
Number of Imaginary Frequencies: 0

Model compound ^{3}A (T$_1$)

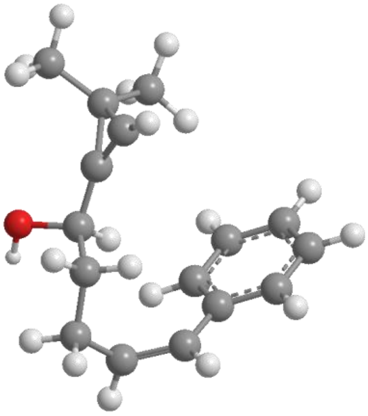

C	2.72083200	1.22357000	-0.28505600
C	1.95496100	-0.05836300	-0.49110700
C	2.29054000	0.56715700	-1.58008300
C	1.98517400	2.46647500	0.21309300
C	4.17856700	1.16788600	0.15951600
C	1.19808800	-1.11930800	0.21187100
H	2.24093400	0.64867500	-2.65653200
H	2.43889800	3.38187300	-0.18849600
H	0.93039300	2.45051200	-0.07895900
H	2.03178500	2.53658500	1.30753300
H	4.25239000	1.17038100	1.25400200
H	4.66724600	0.26158200	-0.20856000
H	4.73901900	2.03662700	-0.20994700
C	-0.30833600	-3.17419600	-0.07085700
H	-0.53229600	-3.98292100	-0.79022700
H	0.20173700	-3.67447000	0.76349300
C	-1.60903300	-2.57805100	0.40549500
H	-2.16395600	-3.14350200	1.15675300
C	-2.32004800	-1.57296700	-0.39002400
H	-2.78538300	-1.90455000	-1.32345700
C	-2.39675900	-0.18722800	-0.10020800
C	-2.94346400	0.72282600	-1.05109900
C	-1.92594700	0.35967000	1.12962000
C	-2.97356400	2.08610600	-0.80408000
H	-3.32184800	0.33139100	-1.99187100
C	-1.95964900	1.72576600	1.36596900
H	-1.54698800	-0.31787600	1.88891300
C	-2.47486700	2.60227300	0.40105700
H	-3.38262200	2.75950600	-1.55192700
H	-1.58715400	2.11734200	2.30818500
H	-2.49590000	3.67116200	0.58881200
H	0.35207800	-0.61289800	0.69817600
O	2.05296500	-1.69428100	1.21102100
H	1.48368800	-2.06394400	1.89704400
C	0.65216000	-2.18344400	-0.74828200
H	1.50438000	-2.72407100	-1.17340300
H	0.13794700	-1.67618800	-1.57263800

Absolute Free Energy: -696.584492 u.a.
Number of Imaginary Frequencies: 0

Model compound TS$_{A-B}$(OH$_{eq}$) (T$_1$)

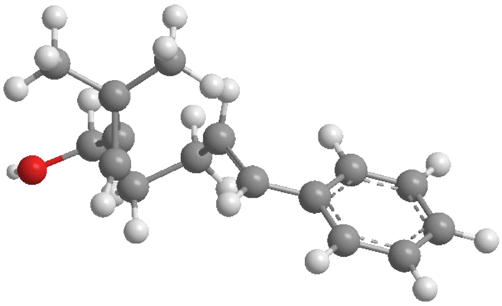

C	2.66058000	-1.46029200	-0.15678400
C	2.18892400	-0.05979400	0.19913600
C	2.19864300	-0.95605900	1.17790600
C	1.78672700	-2.39468700	-0.98745200
C	4.15616800	-1.69272600	-0.36805800
C	2.56331300	1.37622700	-0.01940500
H	2.23547700	-1.12199900	2.24495800
H	2.06156500	-3.44006200	-0.79930800
H	0.72813700	-2.27788300	-0.74429000

H	1.91894400	-2.21258100	-2.06189400
H	4.41570200	-1.61209800	-1.43155200
H	4.74811000	-0.95707300	0.18364500
H	4.45001400	-2.69719500	-0.03827900
C	0.22599600	1.89860000	-0.70836200
H	-0.69525500	2.42860900	-0.43324100
H	0.49644000	2.21783000	-1.72270700
C	0.00120900	0.40207900	-0.68818100
H	0.15965500	-0.12994400	-1.62578400
C	-0.90986800	-0.20440000	0.28972200
H	-0.47706400	-0.69497600	1.16093800
C	-2.32378900	-0.18865100	0.17787700
C	-3.13400900	-0.78039300	1.19035700
C	-2.99455000	0.40884700	-0.92889300
C	-4.51679900	-0.77064500	1.10041600
H	-2.64607100	-1.24401800	2.04382800
C	-4.37932900	0.41220600	-1.00846800
H	-2.40383100	0.86024700	-1.72037900
C	-5.15317900	-0.17421100	0.00212000
H	-5.11090100	-1.22866600	1.88635300
H	-4.86705100	0.87292000	-1.86315700
H	-6.23674100	-0.16798800	-0.06518300
H	2.86552800	1.50209700	-1.07316900
C	1.35920200	2.28180200	0.25872200
H	1.63557500	3.33848800	0.14743800
H	1.04757300	2.12676300	1.29786700
O	3.66757200	1.66090300	0.84707200
H	3.95818100	2.56289900	0.65342400

Absolute Free Energy: -696.571262 u.a.
Number of Imaginary Frequencies: 1 (309i)

Model compound $TS_{A\text{–}B}(OH_{ax})$ (T_1)

C	-1.67672900	1.80260800	0.32044200
C	-1.70290700	0.31567000	0.44348300
C	-1.38816500	1.15263700	1.63453300
C	-0.47346000	2.39548100	-0.42319700
C	-2.97271800	2.57639300	0.10778700
C	-2.83643100	-0.62351200	0.25678200
H	-1.95963900	1.26193200	2.55711200
H	-0.31450000	3.43282800	-0.10841300
H	0.43984700	1.83132300	-0.22502100
H	-0.66277100	2.38399700	-1.50236600
H	-3.25323500	2.56251600	-0.95063100
H	-3.79784300	2.13940600	0.67728400
H	-2.85402700	3.61950600	0.42369200
C	-1.31654000	-2.52427500	-0.49769300
H	-0.94396300	-3.50925800	-0.19356500
H	-1.79723500	-2.64614400	-1.47400300
C	-0.16670300	-1.55615300	-0.63533500
H	-0.25512200	-0.81700200	-1.42607300
C	0.96940100	-1.63923300	0.09259900
H	1.02180400	-2.40640300	0.86613200
C	2.14900800	-0.77968600	-0.01089500
C	3.14213800	-0.85261100	0.98439600
C	2.35001600	0.12704900	-1.07170400
C	4.27558600	-0.04423400	0.93699700
H	3.01163100	-1.54874100	1.80916700
C	3.48119000	0.93609400	-1.11812500
H	1.61818700	0.19106700	-1.87038600
C	4.45044000	0.85829500	-0.11348200
H	5.02307900	-0.11820800	1.72181600
H	3.61169800	1.62738000	-1.94600200

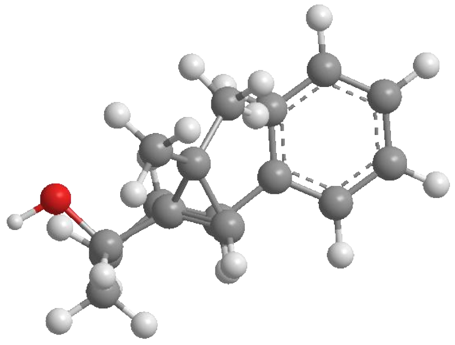

H	5.33307800	1.48954000	-0.15383800
C	-2.38428000	-2.06729100	0.51926800
H	-3.24732600	-2.74729100	0.48780800
H	-1.97380000	-2.11334000	1.53473400
O	-3.33863800	-0.45580500	-1.07360800
H	-4.06531400	-1.08248400	-1.19173200
H	-3.62453300	-0.36758000	0.99800700

Absolute Free Energy: -696.567961 u.a.
Number of Imaginary Frequencies: 1 (56i)

Model compound ^{3}B (T$_1$)

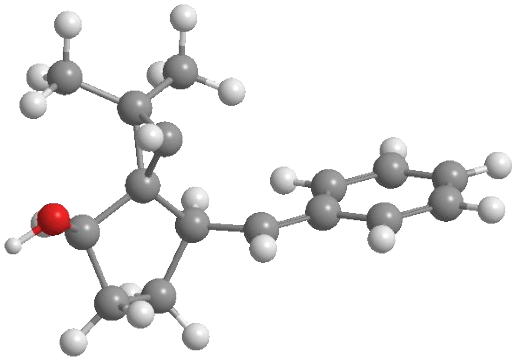

C	1.77583800	1.60532900	-0.16660800
C	1.63501800	0.06389300	-0.06909900
C	1.55202200	0.77330200	-1.35990700
C	0.62522100	2.45282100	0.36648000
C	3.14512800	2.23602000	0.05266000
C	2.84105300	-0.84845900	0.20495800
H	2.15659800	0.63775700	-2.24845000
H	0.66515500	3.45211400	-0.08170200
H	-0.35026500	2.02308800	0.12877300
H	0.69730300	2.57281800	1.45471600
H	3.32125400	2.41968600	1.12050900
H	3.94424700	1.59584400	-0.32674900
H	3.20417100	3.20235600	-0.46164100
C	0.95962800	-1.91520400	1.23929700
H	0.21777300	-2.71790200	1.22663200
H	1.23867100	-1.74552300	2.28633400
C	0.39605100	-0.58433300	0.63130600
H	0.06325500	0.05948700	1.44839300
C	-0.72871400	-0.82727400	-0.32141600
H	-0.46613000	-1.36586300	-1.22996500
C	-2.07601600	-0.42541100	-0.17630400
C	-3.01807500	-0.73810800	-1.20351100
C	-2.57195100	0.29431700	0.95240000
C	-4.34705400	-0.36082000	-1.10763400
H	-2.67088500	-1.28317000	-2.07746300
C	-3.90500800	0.66842000	1.03605800
H	-1.89761700	0.55451500	1.76110600
C	-4.80638100	0.34722100	0.01261400
H	-5.03688800	-0.61450700	-1.90783300
H	-4.25176800	1.21668100	1.90779800
H	-5.84816500	0.64349600	0.08582800
H	3.32643300	-0.52245600	1.14209000
C	2.20892400	-2.22846900	0.41232300
H	2.88372100	-2.94629900	0.89204000
H	1.93739100	-2.62373800	-0.57384900
O	3.77494800	-0.79207100	-0.87022200
H	4.55206400	-1.30128700	-0.60648700

Absolute Free Energy: -696.622139 u.a.
Number of Imaginary Frequencies: 0

Model compound ^{3}B' (T$_1$)

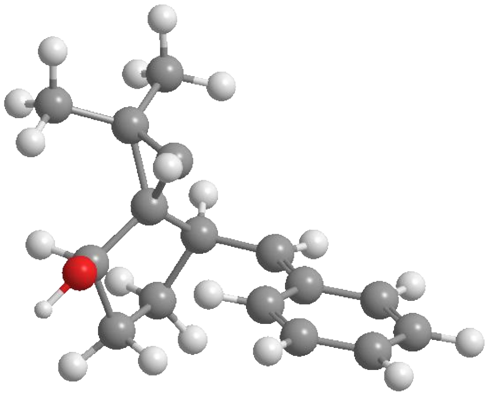

C	-2.42855300	-0.81966100	-0.85047900
C	-1.37422600	-0.06472300	0.01876900
C	-1.12797100	-0.40806600	-1.39121200
C	-2.68724100	-2.29366800	-0.56537000
C	-3.67071400	-0.06084200	-1.30114100
C	-1.63391100	1.39921200	0.43409300
H	-0.86299900	0.23506500	-2.22124000

H	-3.14536500	-2.76443100	-1.44258000
H	-1.76438900	-2.83655800	-0.34778500
H	-3.37525800	-2.42013300	0.27968600
H	-4.45470800	-0.09584300	-0.53355800
H	-3.44465400	0.98624500	-1.51847200
H	-4.07901300	-0.51357600	-2.21181900
C	-0.81666900	0.24438900	2.38042900
H	-0.04167600	0.12423200	3.14188600
H	-1.78758200	0.08394400	2.86634400
C	-0.66149100	-0.77532400	1.20300000
H	-1.21555400	-1.67930900	1.47212000
C	0.76335600	-1.21434800	0.99369900
H	1.02567900	-2.11590900	1.54616000
C	1.84452500	-0.60923100	0.30260300
C	3.12061200	-1.25076300	0.37159100
C	1.77961200	0.59701700	-0.45977400
C	4.23828900	-0.73002900	-0.25878500
H	3.20692300	-2.17337400	0.94023600
C	2.90859200	1.10846300	-1.08511300
H	0.84401700	1.12588000	-0.57217600
C	4.14580200	0.45930000	-0.99473200
H	5.19029900	-1.24787700	-0.17990400
H	2.82270300	2.02835900	-1.65735800
H	5.02075200	0.86952600	-1.48974300
H	-2.70131700	1.51083200	0.68713500
C	-0.79353300	1.61547600	1.70160900
H	-1.18285100	2.42016700	2.33556200
H	0.22800600	1.87928500	1.41105900
O	-1.30730200	2.27729000	-0.64470300
H	-1.46347200	3.18193800	-0.34304100

Absolute Free Energy: -696.617340 u.a.
Number of Imaginary Frequencies: 0

Model compound TS_{B-C} (T_1)

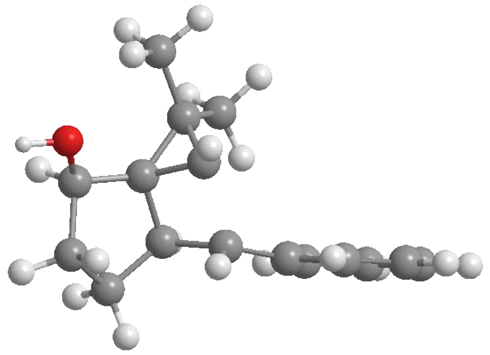

C	-1.09847700	1.57455400	-0.14781000
C	-1.59813700	0.14220000	-0.22649800
C	-0.72004600	0.58618500	0.91412900
C	-0.11238900	2.13795700	-1.16058300
C	-2.10830000	2.62856300	0.30420500
C	-2.98716200	-0.45675800	-0.10667300
H	-0.96867800	0.67561100	1.96690200
H	0.37929800	3.02369000	-0.74259400
H	0.67223700	1.43546900	-1.43517000
H	-0.63686500	2.44686600	-2.07274500
H	-2.65614100	3.02083300	-0.56190200
H	-2.83629600	2.22460900	1.00783300
H	-1.58429000	3.46880500	0.77379100
C	-1.47735800	-2.20645600	-0.91523000
H	-0.89898200	-3.09428400	-0.64100000
H	-1.82338600	-2.35055800	-1.94535700
C	-0.64216400	-0.90566200	-0.83561100
H	-0.22474600	-0.64183900	-1.80981800
C	0.38056800	-0.82194600	0.30365500
H	0.10752100	-1.45900500	1.15205400
C	1.76552500	-0.60765900	0.17296000
C	2.58348700	-0.52855800	1.41774300
C	2.46133100	-0.27844300	-1.08235800
C	3.88982500	-0.14932300	1.38250600
H	2.09795900	-0.77221400	2.35865500
C	3.76523900	0.08881700	-1.08857800
H	1.92364300	-0.37891700	-2.01977900
C	4.52633000	0.18248400	0.14582700

H	4.46386600	-0.09404800	2.30387300
H	4.26545500	0.30104100	-2.02940900
H	5.56543200	0.49005500	0.12352900
H	-3.55917400	-0.25954400	-1.03033000
C	-2.68165800	-1.96646100	0.01455900
H	-3.54299200	-2.60092500	-0.22147800
H	-2.40866900	-2.14924200	1.06049400
O	-3.68030800	0.06291600	1.02401600
H	-4.57240600	-0.30668400	1.01686800

Absolute Free Energy: -696.555803 u.a.
Number of Imaginary Frequencies: 1 (878i)

Model compound ^{3}C (T_1)

C	-1.11704400	1.48558000	-0.34722900
C	-1.63167000	0.08014500	-0.29644000
C	-0.71949100	0.59041500	0.82766700
C	-0.15053000	1.94269700	-1.42984100
C	-2.10297500	2.58203800	0.03880300
C	-2.99310300	-0.55720000	-0.14375200
H	-1.03523100	0.86861900	1.82881900
H	0.38260000	2.84398500	-1.10728300
H	0.60255900	1.19469400	-1.67995800
H	-0.70353100	2.19157000	-2.34357200
H	-2.68180200	2.89653000	-0.83733000
H	-2.80888300	2.24900200	0.80044200
H	-1.56159900	3.45920800	0.41192900
C	-1.34551700	-2.33363100	-0.54729300
H	-0.74051400	-3.11506100	-0.07401400
H	-1.61980000	-2.69924700	-1.54375800
C	-0.59380700	-0.99489800	-0.68811200
H	-0.11083100	-0.89167300	-1.66333300
C	0.29767100	-0.52073500	0.53635400
H	0.19976300	-1.30937700	1.30563600
C	1.73385900	-0.23615000	0.32815100
C	2.35210000	0.99522200	0.60477500
C	2.61097900	-1.39677400	-0.13033900
C	3.71300400	1.16188500	0.49716900
H	1.72372000	1.82413100	0.92083900
C	3.98258300	-1.23258000	-0.21359200
H	2.13125200	-2.33440400	-0.39009000
C	4.57793400	-0.00700900	0.08164100
H	4.18473300	2.11345600	0.71417400
H	4.60613400	-2.06734300	-0.52550800
H	5.65208900	0.12521300	0.01724700
H	-3.51742100	-0.56007300	-1.11577500
C	-2.61813800	-2.00350600	0.26030900
H	-3.42975100	-2.72124300	0.09730300
H	-2.40079800	-1.98060800	1.33456300
O	-3.77667600	0.11346300	0.83747300
H	-4.63439300	-0.32807700	0.88026200

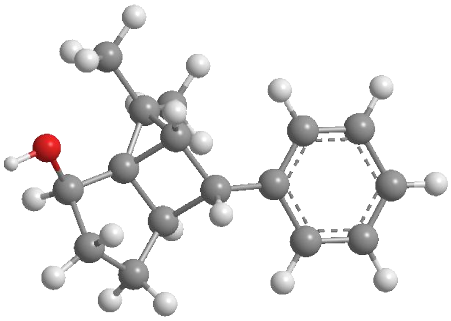

Absolute Free Energy: -696.572055 u.a.
Number of Imaginary Frequencies: 0

Model compound MECP (T_1/S_0)

C	-1.61993400	1.60751600	0.09438900
C	-1.63277800	0.07581300	-0.05430800
C	-1.23838400	0.72621200	1.21803400
C	-0.56860900	2.42631100	-0.64577200
C	-2.96322400	2.32717200	0.19350400
C	-2.87585900	-0.80040900	-0.20256000

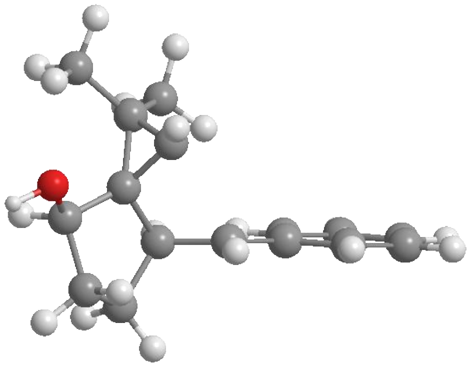

H	-1.65150100	0.63544300	2.21593200
H	-0.50004100	3.42571300	-0.20095100
H	0.42238200	1.97394500	-0.59079500
H	-0.84484800	2.55088000	-1.70008200
H	-3.33794300	2.57751600	-0.80750900
H	-3.71255200	1.71640600	0.69899300
H	-2.84204000	3.26684400	0.74456500
C	-1.02977700	-2.02896700	-1.17566700
H	-0.30381400	-2.83954700	-1.06313600
H	-1.30991200	-1.98348400	-2.23506700
C	-0.45009700	-0.64858400	-0.73179500
H	-0.09101300	-0.10192000	-1.60713100
C	0.62117800	-0.74405900	0.32312800
H	0.34108500	-1.30175500	1.21313500
C	1.99036500	-0.39545700	0.19239200
C	2.88751600	-0.65407600	1.26991500
C	2.53881400	0.21471400	-0.97183700
C	4.23312300	-0.33532600	1.18335500
H	2.49540600	-1.11290700	2.17349800
C	3.88786700	0.53127800	-1.04851100
H	1.89499900	0.43020500	-1.81750300
C	4.74820900	0.25806200	0.02197300
H	4.89197400	-0.54955600	2.02003800
H	4.27808000	0.99370000	-1.95075900
H	5.80273600	0.50533800	-0.04598300
H	-3.39699700	-0.54440100	-1.14225500
C	-2.28480200	-2.21698500	-0.31091300
H	-2.99111900	-2.94821400	-0.71958600
H	-2.01587700	-2.53484100	0.70389600
O	-3.75436200	-0.63480300	0.90725700
H	-4.55576600	-1.14023300	0.72069200

Absolute Free Energy: -696.608313 u.a.
Number of Imaginary Frequencies: 0

Model compound 2a (S_0)

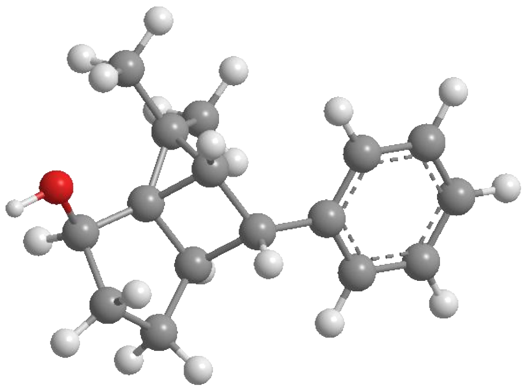

C	-0.99897200	1.45634100	-0.40726000
C	-1.60960500	0.09183200	-0.30974200
C	-0.69345200	0.59282700	0.81570700
C	0.03281400	1.79925000	-1.47209200
C	-1.91698200	2.63354900	-0.09997400
C	-3.01303100	-0.44753000	-0.16654000
H	-1.01412300	0.93598300	1.79509200
H	0.63831000	2.65395100	-1.15084000
H	0.72085600	0.98168000	-1.68900600
H	-0.47147600	2.07756400	-2.40525200
H	-2.45062700	2.94465700	-1.00550700
H	-2.66324300	2.38501100	0.65546100
H	-1.32633300	3.48893600	0.24821800
C	-1.47358100	-2.34370200	-0.41746300
H	-0.93507500	-3.13638000	0.11308100
H	-1.74648800	-2.74360700	-1.40110100
C	-0.62951000	-1.06497300	-0.60825300
H	-0.12162300	-1.05108500	-1.57711600
C	0.25980000	-0.59217500	0.59343400
H	0.13030800	-1.30657200	1.41857200
C	1.73827700	-0.36049800	0.38730200
C	2.36113000	0.80146400	0.85680000
C	2.52091500	-1.32305600	-0.26438900
C	3.73206500	0.99716400	0.68462700
H	1.75911100	1.56113300	1.34745000
C	3.89093800	-1.12996900	-0.44262400
H	2.04957000	-2.22914800	-0.63771000

C	4.50218000	0.03221900	0.03303400
H	4.19800700	1.90657700	1.05399500
H	4.48131900	-1.88520100	-0.95414000
H	5.56832400	0.18557600	-0.10663600
H	-3.50725200	-0.47563500	-1.15392100
C	-2.74328100	-1.88795400	0.33133600
H	-3.59467100	-2.56197300	0.18508500
H	-2.55390400	-1.81500100	1.40865300
O	-3.78173100	0.32688300	0.74783900
H	-4.66537900	-0.06026700	0.78948800

Absolute Free Energy: -696.698902 u.a.
Number of Imaginary Frequencies: 0

Model compound TSA–A' (T1)

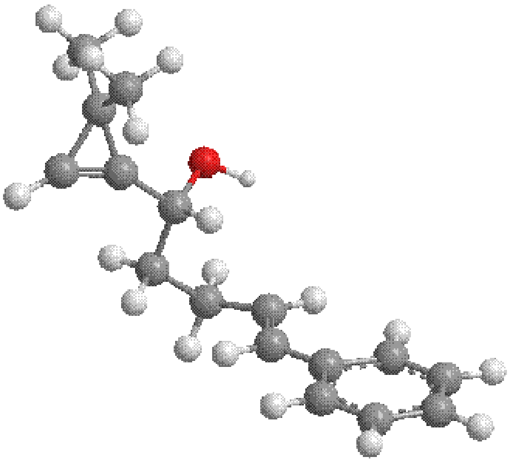

C	4.08420500	-1.04972800	-0.05970900
C	2.95056100	-0.16695000	0.39744400
C	3.76828400	-0.54926100	1.33371900
C	3.81682000	-2.50791000	-0.42450100
C	5.23342000	-0.43622200	-0.85346000
C	1.73956900	0.56358900	-0.04561000
H	4.04469600	-0.55983100	2.37837900
H	4.69868900	-3.13103400	-0.22784500
H	2.97921800	-2.91531100	0.15023800
H	3.57975900	-2.60860100	-1.49112500
H	5.02087500	-0.46516900	-1.92888800
H	5.39350000	0.60788300	-0.57084500
H	6.16838200	-0.98669600	-0.68632100
C	0.11132900	2.47080000	0.50044800
H	-0.24150800	3.07428800	1.35709700
H	0.51606000	3.18425200	-0.22429600
C	-1.03420800	1.73311200	-0.11331300
H	-1.47662000	2.12224600	-1.02373400
C	-1.71571500	0.63899700	0.60879900
H	-1.22475800	0.27777800	1.50980200
C	-2.90852100	0.04329800	0.25427100
C	-3.46208000	-1.02885600	1.06296200
C	-3.67354800	0.42375000	-0.92145400
C	-4.63954200	-1.64619000	0.72676100
H	-2.91453500	-1.33658800	1.94992200
C	-4.85618100	-0.21140100	-1.23464800
H	-3.30909700	1.22033100	-1.55954600
C	-5.36000600	-1.25015200	-0.42795100
H	-5.02677700	-2.44669000	1.35080300
H	-5.40851700	0.09327200	-2.11943800
H	-6.29047600	-1.74397400	-0.68776300
H	0.93700200	-0.17969000	-0.19677200
O	2.04512800	1.19435200	-1.29317300
H	1.19795700	1.38612900	-1.71786300
C	1.26568400	1.57605400	1.00964100
H	2.10929800	2.21298000	1.29615200
H	0.96541200	1.02203300	1.90702500

Absolute Free Energy: -696.571534 u.a.
Number of Imaginary Frequencies: 1 (83i)

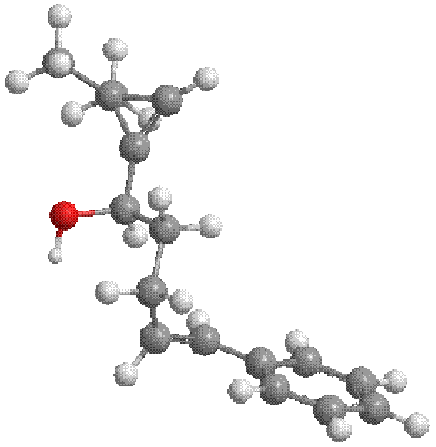

Model compound 3A' (T1)

C	-4.15291500	-0.90060800	-0.20858000
C	-2.78246200	-0.30513200	-0.41002800
C	-3.17267300	-1.12505900	-1.34057900
C	-4.36664500	-1.98841700	0.84116800
C	-5.38445300	-0.02588600	-0.42153100
C	-1.76303700	0.59752700	0.17674000

H	-2.96545700	-1.65177500	-2.26110200
H	-5.18132700	-2.66329300	0.54881400
H	-3.46293900	-2.58984700	0.98125200
H	-4.63978000	-1.55139700	1.80999500
H	-5.66798100	0.47823000	0.51019500
H	-5.19486200	0.74338200	-1.17512400
H	-6.24438300	-0.62505800	-0.74809300
C	0.30314500	2.03606200	-0.31899200
H	1.02878100	2.24717900	-1.12396500
H	-0.22359700	2.98040600	-0.13885300
C	1.04376200	1.61780300	0.92313100
H	1.30038300	2.38912500	1.65093300
C	1.62905900	0.28298400	1.06957300
H	1.07333900	-0.46773800	1.63610000
C	2.86360600	-0.13011500	0.50552000
C	3.32216600	-1.46996000	0.66683600
C	3.68981800	0.75951900	-0.24118300
C	4.52161600	-1.88853000	0.11330900
H	2.71050300	-2.16744400	1.23335200
C	4.88861800	0.32925300	-0.78943700
H	3.37041100	1.78913000	-0.36994100
C	5.31551400	-0.99507800	-0.62026300
H	4.84797300	-2.91577300	0.24923300
H	5.50182600	1.02618800	-1.35391100
H	6.25435500	-1.32653200	-1.05297700
H	-1.27374800	0.05179100	1.00215900
O	-2.45092100	1.73829700	0.70165500
H	-1.85326600	2.16140000	1.33165200
C	-0.69219800	0.99085800	-0.85313900
H	-1.19373900	1.39054200	-1.74087100
H	-0.15664700	0.08378100	-1.15736200

Absolute Free Energy: -696.583291 u.a.
Number of Imaginary Frequencies: 0

Model compound TSA'–B'(OHeq) (T1)

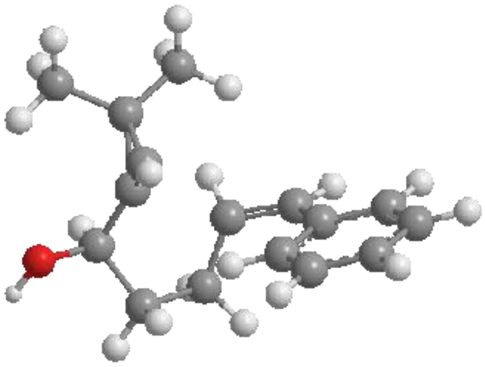

C	2.03860800	1.59874700	-0.09871600
C	1.67866800	0.12756600	-0.23241000
C	1.15175200	0.96088600	-1.12071600
C	1.45178200	2.45450600	1.02033000
C	3.43715200	2.04694900	-0.52512700
C	2.35001700	-1.21264500	-0.14439000
H	0.65064300	1.08656100	-2.06876100
H	1.44285300	3.51094100	0.72454600
H	0.42440100	2.16087400	1.24938400
H	2.05090600	2.38112200	1.93760900
H	4.11819800	2.06674800	0.33559800
H	3.85411300	1.36731100	-1.27339700
H	3.41580500	3.06057300	-0.94486300
C	0.56687000	-2.07294900	1.40523400
H	-0.35564300	-2.66264700	1.42341900
H	1.19148100	-2.41934000	2.24106000
C	0.26601900	-0.59645100	1.56648400
H	0.98403800	-0.03085100	2.15473400
C	-1.11813800	-0.09106000	1.64000200
H	3.04510300	-1.18981900	0.71252900
C	1.32507600	-2.32771700	0.08348100
H	1.82285200	-3.30621300	0.10005600
H	0.63478600	-2.33144700	-0.76669000
O	3.07835400	-1.39681000	-1.36399700
H	3.59237300	-2.21083500	-1.27093400
C	-2.08025800	0.00919800	0.61088700
C	-1.83931900	-0.40071700	-0.73533000

C	-3.38628600	0.50667800	0.91125500
C	-2.82918800	-0.30990600	-1.69923600
H	-0.86147300	-0.78144600	-0.99664900
C	-4.36634400	0.59730400	-0.06300500
H	-3.60310100	0.82015000	1.92930900
C	-4.10047100	0.19096200	-1.37880900
H	-2.61712600	-0.63266900	-2.71497400
H	-5.34809700	0.98388900	0.19614200
H	-4.87122400	0.25898500	-2.14023500
H	-1.47334900	0.15447800	2.64449100

Absolute Free Energy: -696.564902 u.a.
Number of Imaginary Frequencies: 1 (344i)

Model compound MECP’ (T1/S0)

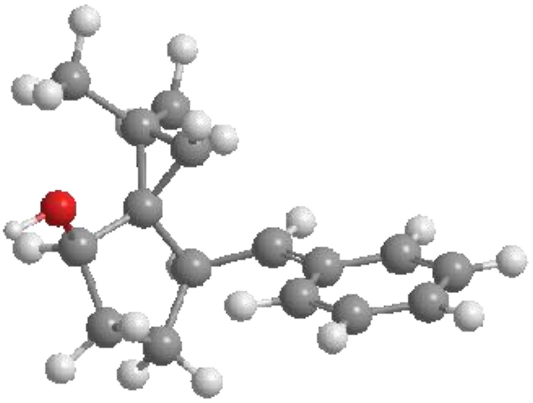

C	-2.26501580	-1.21208720	-0.38041440
C	-1.50787690	0.02182980	0.14935770
C	-1.01638350	-0.74691300	-1.01852090
C	-2.31396980	-2.49798810	0.43673560
C	-3.57427990	-0.96537250	-1.13210510
C	-2.06177230	1.44486780	0.11198740
H	-0.77737920	-0.46120140	-2.03588830
H	-2.56767240	-3.34299810	-0.21373180
H	-1.36119280	-2.72821830	0.91485840
H	-3.08499980	-2.43438220	1.21373490
H	-4.41635470	-0.92803990	-0.42843580
H	-3.55103060	-0.02917320	-1.69069470
H	-3.76541040	-1.78765600	-1.83106200
C	-0.34313560	1.34579710	1.87736220
H	0.71320400	1.57724110	2.03433010
H	-0.83612040	1.45225280	2.84881200
C	-0.56007050	-0.11111830	1.35635780
H	-1.03842540	-0.68836600	2.15595040
C	0.64152600	-0.92077450	0.89837240
H	0.64523820	-1.95645410	1.22927550
C	1.86131690	-0.49312430	0.29897190
C	2.94479430	-1.41560780	0.23338070
C	2.07842420	0.79133090	-0.27114240
C	4.15833920	-1.07381350	-0.34278700
H	2.80863120	-2.40849530	0.65436500
C	3.29663870	1.12527500	-0.84704360
H	1.27040580	1.50886020	-0.29241960
C	4.34947160	0.20354380	-0.88470940
H	4.96381100	-1.80241540	-0.37140430
H	3.42809210	2.11310340	-1.28024500
H	5.29950460	0.47233050	-1.33598080
H	-3.01537100	1.47118940	0.66700630
C	-0.99734980	2.28489540	0.84442210
H	-1.41125310	3.19111790	1.29993260
H	-0.27852740	2.60739770	0.08405800
O	-2.26907790	1.86949220	-1.23519540
H	-2.72739740	2.71918950	-1.20811010

Absolute Free Energy: -696.600968 u.a.

Model compound 2a’ (S0)

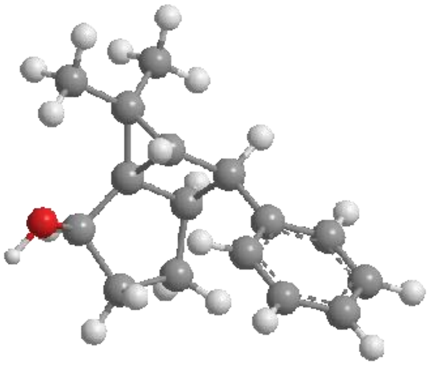

C	-2.31312400	-1.03382100	-0.35533300
C	-1.61026400	0.09651100	0.33542000
C	-0.90530800	-0.65237400	-0.80038800
C	-2.62508000	-2.34457900	0.35084000
C	-3.39656600	-0.64662600	-1.35434300
C	-1.85412500	1.58101100	0.46442200

H	-0.75531300	-0.31058100	-1.81976800
H	-2.75718500	-3.14681000	-0.38428500
H	-1.84495200	-2.66012500	1.04703200
H	-3.55899900	-2.25757000	0.91822700
H	-4.35904700	-0.52719500	-0.84334800
H	-3.16945600	0.29469200	-1.85626300
H	-3.51463200	-1.43434600	-2.10753000
C	0.07997800	0.96423400	1.86967500
H	1.17343700	0.96378800	1.88911500
H	-0.26170100	1.09767500	2.90218600
C	-0.51397600	-0.35377700	1.32294500
H	-0.82218200	-1.01453600	2.13971900
C	0.21814400	-1.11400400	0.14169200
H	-2.63762500	1.76768100	1.21995100
C	-0.48507500	2.08972700	0.97690000
H	-0.55555100	3.05128300	1.49773000
H	0.14841600	2.23160200	0.09580000
O	-2.22941800	2.15494000	-0.78533100
H	-2.37403400	3.09883100	-0.64159200
C	1.61973300	-0.64506100	-0.16143100
C	1.90066600	0.38359700	-1.07089600
C	2.69513900	-1.22789200	0.52786200
C	3.21206400	0.81594300	-1.28274000
H	1.09229800	0.85346600	-1.62059600
C	4.00428100	-0.79734500	0.32194700
H	2.49686300	-2.03033900	1.23466500
C	4.26894500	0.22967800	-0.58756800
H	3.40491800	1.61280200	-1.99566200
H	4.81890700	-1.26638400	0.86673300
H	5.28845300	0.56499400	-0.75398900
H	0.25013700	-2.19977300	0.30050400

Absolute Free Energy: -696.697082 u.a.
Number of Imaginary Frequencies: 0

6. Synthesis of housane 2a at 1.0 mmol scale.

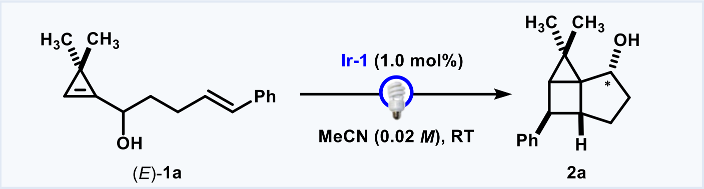

The representative procedure D was followed using cyclopropene **1a** (231.0 mg, 1.01 mmol) and **Ir-1** (10.0 mg, 1.0 mol%) in MeCN (0.02 M). After 3.5 h, analysis of the crude mixture indicated the disappearance of **1a** (NMR yield = 76%, *dr* = 4.5:1). Purification by flash chromatography (deactivated SiO_2, hexanes:EtOAc = 20:1) enabled the isolation **2a** (168 mg, 73%, colorless oil) as a mixture of diastereoisomers.

Photo-reactor employed.

This experiment was performed with 456 nm LED lamp (Kessil® – PR-160-456nm) which were installed at 4 cm from the Schlenk flask, equipped with a fun cooling system and a magnetic stirrer (~ 300 rpm). The material of Schlenk tubes is borosilicate glass and they were used without any filter.

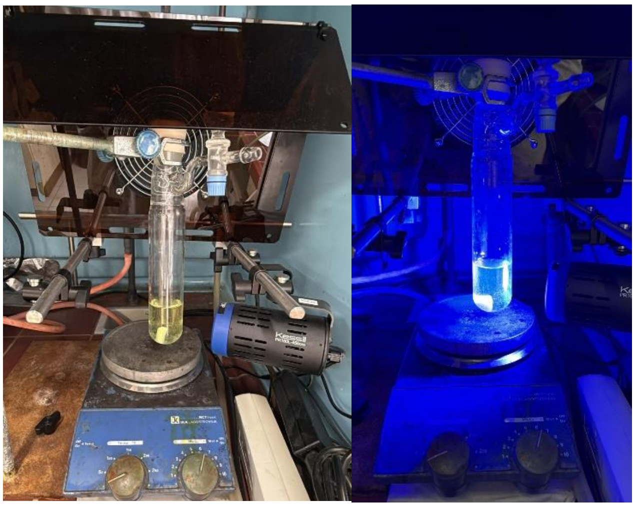

7. Derivatizations of housanes 2: Steglich reaction and Swern oxidation.

Steglich reaction.

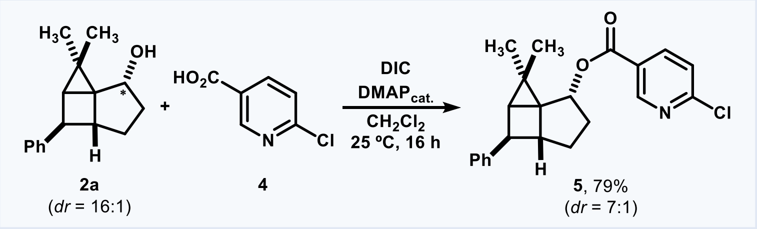

To a solution of housane **2a** (14.8 mg, 0.065 mmol, *dr* = 16:1) in CH_2Cl_2 (2.0 mL), 6-chloronicotinic acid (**4**) (22.9 mg, 1.45 mmol), DIC (19 mg, 1.45 mmol) and DMAP (4.0 mg, 0.013 mmol) were added at 0 °C and the resulting mixture was stirred at ambient temperature overnight. The solvent was removed under reduced pressure and the resulting residue was purified by flash chromatography (deactivated SiO_2, hexane/EtOAc = 50:1 to 20:1) to afford compound **5** (18.8 mg, 79%, *dr* = 7:1) as a colorless oil.

Characterization data for the mayor isomer (**5**):

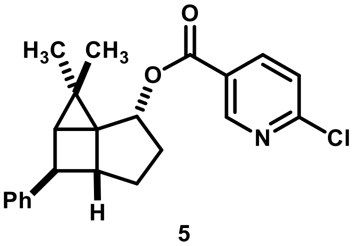

5

(1*R,3*S**,4*R**,5*R**,8*R**)-2,2-Dimethyl-4-phenyltricyclo[3.3.0.0^{1,3}]octan-8-yl 6-chloronicotinate** (**5**):

^{1}H-NMR (300 MHz, $CDCl_3$) δ = 9.01 (dd, *J* = 2.4, 0.8 Hz, 1H), 8.24 (dd, *J* = 8.3, 2.4 Hz, 1H), 7.43 (dd, *J* = 8.3, 0.8 Hz, 1H), 7.35 – 7.23 (m, 2H), 7.22 – 7.09 (m, 3H), 5.60 (dd, *J* = 5.5, 2.3 Hz, 1H), 3.39 (t, *J* = 4.3 Hz, 1H), 2.53 – 2.36 (m, 1H), 1.86 – 2.30 (m, 4H), 1.76 (dd, *J* = 5.2, 1.2 Hz, 1H), 1.03 (s, 3H), 0.82 (s, 3H).

^{13}C-NMR (75 MHz, $CDCl_3$) δ = 163.9, 155.6, 151.1, 143.3, 139.5, 127.9, 125.9, 125.7, 125.4, 124.2, 74.8, 43.9, 41.2, 40.6, 36.7, 32.6, 25.7, 25.5, 21.8, 18.6.

HRMS (ESI) m/z: $[M+H]^+$ Calcd for $[C_{22}H_{23}ClNO_2]^+$ 368.1412; found 368.1407 (of the mixture of diastereoisomers).

Swern oxidation.

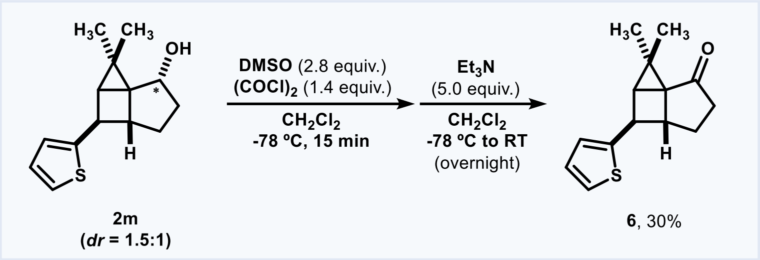

To a solution of oxalyl chloride (26 µL, 0.304 mmol, 1.4 equiv.) in CH_2Cl_2 (1.0 mL) at –78 °C, a solution of DMSO (44 µL, 0.610 mmol, 2.8 equiv.) in CH_2Cl_2 (1.0 mL) was added dropwise and the resulting mixture was stirred for 5 min at this temperature. A solution of housane **2m** (51.0 mg, 0.218 mmol, *dr* = 1.5:1) in CH_2Cl_2 (1.0 mL) was added dropwiseto the mixture and stirred for 20 min. Then, Et_3N (152 µL, 1.09 mmol, 5.0 equiv.) was added and the resulting mixture was stirred and allowed to warm slowly to ambient temperature overnight. The mixture was diluted with Et_2O (10 mL) and water was added (10 mL). The layers were separated and the organic layer was consecutively washed with a cold solution of HCl (10 mL, 1.0 M), saturated solution of $NaHCO_3$ (15 mL), water (10 mL) and brine (10 mL) and dried over Na_2SO_4. After filtration and removal of the solvent, the resulting residue was purified by flash column chromatography (deactivated SiO_2, hexanes:EtOAc = 20:1) to afford compound **6** (15.2 mg, 30%) as a colorless oil.

Characterization data for compound **6**:

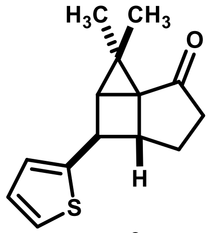

6

(1*R,3*S**,4*R**,5*R**)-2,2-Dimethyl-4-(thiophen-2-yl)tricyclo[3.3.0.0^{1,3}]octan-8-one** (**6**):

^{1}H-NMR (300 MHz, $CDCl_3$) δ = 7.16 (d, *J* = 5.1 Hz, 1H), 6.92 (dd, *J* = 5.1, 3.4 Hz, 1H), 6.80 – 6.61 (m, 1H), 3.65 – 3.34 (m, 1H), 2.79 – 2.41 (m, 5H), 2.08 – 1.89 (m, 1H), 1.43 (s, 3H), 1.13 (s, 3H).

^{13}C-NMR (75 MHz, $CDCl_3$) δ = 215.5, 145.8, 126.6, 123.4, 123.3, 44.8, 41.9, 41.6, 40.3, 38.6, 35.3, 28.1, 20.4, 19.3.

HRMS (EI) m/z: $[M]^+$ Calcd for $[C_{14}H_{16}O_2S]^+$ 232.0924; found 232.0922.

8. Crystallographic data for compound 2k.

Suitable crystals from compound **2k** were obtained by slow diffusion of pentane into a CH_2Cl_2 solution of **2k** at –20 °C.

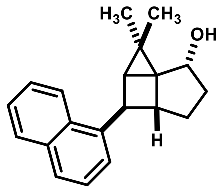

2k

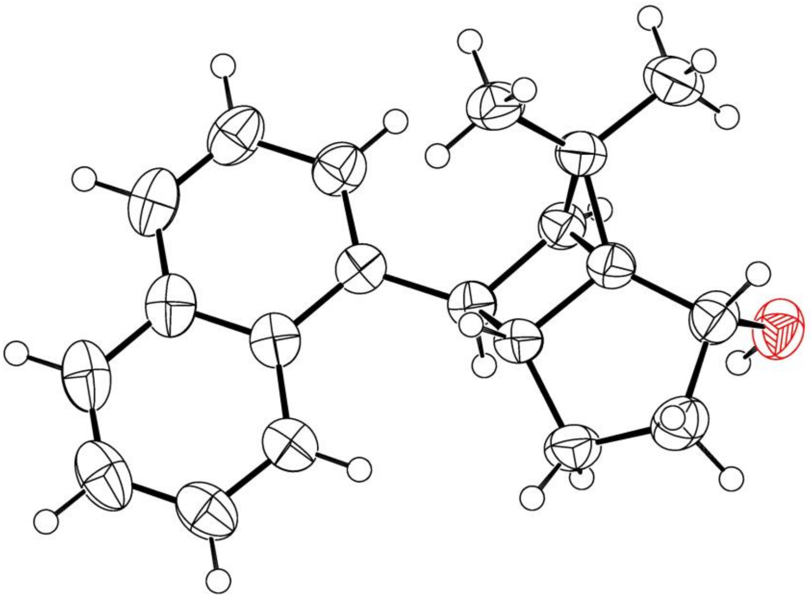

[The ellipsoids contours are shown at 50% probability lever]

2k: empirical formula $C_{20}H_{22}O$, M_r = 278.37. T = 150.0 (2) K, λ = 1.54184 Å

Crystal system, space group: triclinic, *I-4,* unit cell dimensions: $a = b$ = 20.6600 (4), c = 7.5201 (2) Å, $\alpha = \beta = \gamma$ = 90°, V = 3209.85 (15) Å^3, Z = 8, ρ_{calcd} = 1.152 Mgm^{-3}, μ = 0.527 mm^{-1}, F(000) = 1200, crystal size: 0.399 x 0.310 x 0.224 mm

θ range data collection: 3.025-69.588º, index ranges: -25≤h≤24, -24≤k≤25, -8≤l≤9, reflections collected/unique = 7561/2711

R_{int} = 0.0388, completeness to 2θ = 67.684 (99.9%)

Absorption correction: semiempirical from equivalents, max. and min. transmission = 1.000 and 0.938

Refinement method: full matrix least-squares on F^2, data/restraints/parameters = 2711/0/207, goodness-of-fit on F^2 = 1.103, final R indices [I>2σ(I)]: R_1 = 0.0425, wR_2 = 0.1210, R indices (all data): R_1 = 0.0450, wR_2 = 0.1235, extinction coefficient = 0.0016 (3), largest difference peak and hole = 0.138 and – 0.154 eÅ^{-3}.

Deposit number: CCDC2410203.

X-ray data were collected on a D8 Venture Bruker diffractometers (Mo radiation, λ = 0.71073 Å). The crystals were mounted under oil in a MiTeGen mount and cooled to 273(2) K with an open-flow nitrogen gas (Oxford Cryosystems).

9. Synthesis and characterization of starting aldehydes S2.

Known aldehydes **S2** were prepared according to reported procedures as indicated above. The aldehydes **S2** indicated below have not been described and were prepared according to the following procedures.

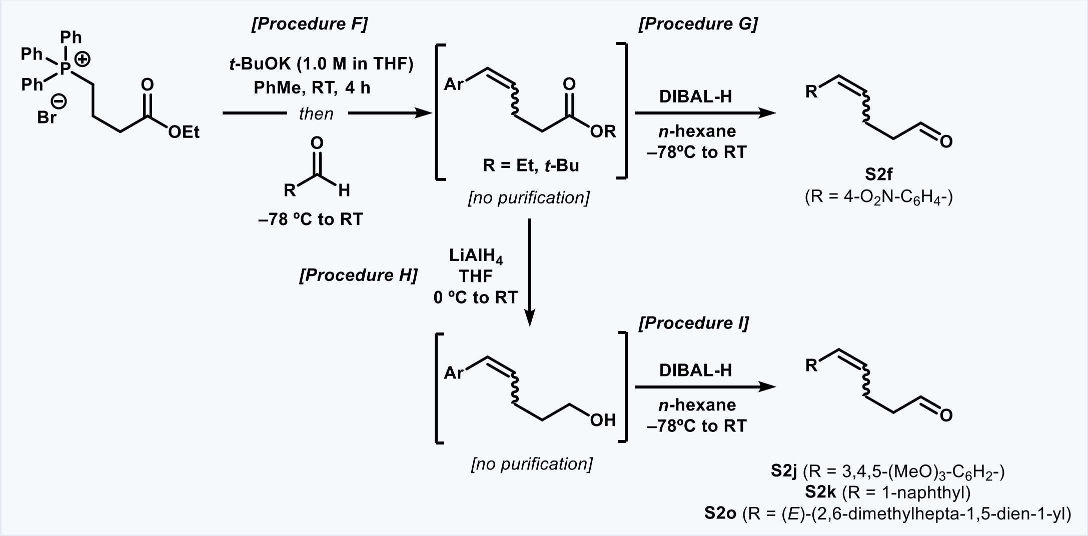

Representative procedure F.

To a suspension of (4-ethoxy-4-oxobutyl)triphenylphosphonium bromide[36] in toluene (ca. 0.25 M), a solution of t-BuOK (2.0 equiv.) in THF (1.0M) was added dropwise at ambient temperature and stirred 4 h (red color was developed). The reaction mixture was cooled to –78 °C and a solution of the corresponding aldehyde (1.0 equiv.) in THF (ca. 5.0 mL) was added dropwise. The resulting mixture was slowly allowed to warm to ambient temperature and stirred overnight. A saturated solution of NH_4Cl (50 mL) and Et_2O (100 mL) were added and the phases were separated. The water layer was extracted with Et_2O (2 x 50 mL) and the collected organic phases were successively washed with water (50 mL) and brine (50 mL) and dried over anhydrous Na_2SO_4. After filtration and removal of the solvent, the corresponding alkene was obtained as mixtures of *Z:E* isomers, along with the *t*-butyl ester (*Z:E* isomers) arising from a transesterification reaction. The mixture was employed for the next step without further purification.

Representative procedure G.

To a solution of the esters mixture (1.0 equiv.) in *n*-hexane (0.1 M) at –78 °C, a solution of DIBAL-H (1.0 equiv., 1.0 M in *n*-hexane) was added dropwise. The resulting mixture was stirred at this

[36] Maulucci, N.; Chini, M. G.; Di Micco, S.; Izzo, I.; Cafaro, E.; Russo, A.; Gallinari, P.; Paolini, C.; Nardi, M. C.; Casapullo, A.; Riccio, R.; Bifulco, G.; De Riccardis, F. Molecular Insights into Azumamide E Histone Deacetylases Inhibitory Activity. *J. Am. Chem. Soc.* **2007**, *129*, 3007–3012.

temperature until the dissapearance of the starting material (TLC analysis) and quenched with saturated aqueous solution of Rochelle's salt. The mixture was warmed up to ambient temperature and Et_2O (50 mL) was added. The organic phase was successively washed with water (2 x 30 mL) and brine (60 mL). The organic phase was dried over anhydrous Na_2SO_4. After filtration and removal of the solvent, the resulting residue was purified by flash column chromatography (SiO_2, hexanes:EtOAc = 10:1) to afford the corresponding aldehyde as a mixture of *Z:E* isomers.

Representative procedure H.

To a solution of the esters mixture (1.0 equiv.) in THF (ca. 0.15 M) at 0 °C, $LiAlH_4$ (1.3 equiv.) was added in small portions. The resulting suspension was allowed to warm to ambient temperature and stirred for 90 min. At 0 °C, water (mL x mg $LiAlH_4$), NaOH (15%w, mL x mg $LiAlH_4$) and water (3mL x mg $LiAlH_4$) were consecutively added, the mixture was stirred for 15 min at ambient temperature, filtered through a short pad of Celite®, and the pad washed with MeOH. The solvent was removed and the residue was used for the next step without further purification.

Representative procedure I.

To a solution of oxalyl chloride (4.0 equiv.) in CH_2Cl_2 (0.5 M) at –78 °C, a solution of DMSO (3.0 equiv.) in CH_2Cl_2 (3.0 mL) was added dropwise. After 5 minutes, a solution of the corresponding alcohol (1.0 equiv.) in CH_2Cl_2 (ca. 0.1 M) was added. The resulting solution was stirred at –78 °C for 15 minutes and was allowed to warm to –40 °C and stirred at this temperature for 1 h. Then, a solution of Et_3N (15-20 equiv.) in CH_2Cl_2 (5.0 mL) was added dropwise and the resulting solution was allowed to wart to ambient temperature during 4 h. Water (20 mL) was added and the phases were separated. The aqueous phase was extracted with CH_2Cl_2 (2 x 30 mL). The organic phases were collected and dried over anhydrous Na_2SO_4. After filtration and removal of the solvent, the residue was purified by flash column chromatography (SiO_2, hexanes:EtOAc) to afford the corresponding aldehydes.

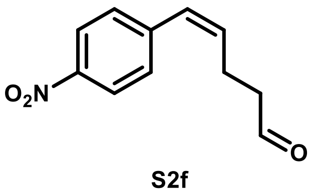

S2f

5-(4-Nitrophenyl)pent-4-enal (**S2f**): The representative procedure F was followed using 4-nitrobenzaldehyde (0.58 g, 2.84 mmol), (4-ethoxy-4-oxobutyl)triphenylphosphonium bromide

(2.28 g, 5.00 mmol) and *t*-BuOK (5.0 mL, 1.0 M in THF). A mixture of Z/E mixtures of ethyl and *t*-butyl esters (665 mg) was obtained and used for the reduction according to the representative procedure G. After purification by flash column chromatography (SiO_2, hexanes:EtOAc = 5:1) yielded **S2f** (0.140 mg, 24 % for three steps, *Z:E* =3:1) as a yellow oil.

^{1}H NMR (400 MHz, $CDCl_3$) δ = 9.78 (s, 1H, *E*), 9.73 (s, 1H, *Z*), 8.11 (d, *J* = 8.4 Hz, 7H), 8.06 (d, *J* = 8.4 Hz, 2H, *Z*), 7.42 – 7.28 (m, 2H, *Z* + m, 2H, *E*), 6.51 – 6.27 (m, 1H, *Z* + m, 2H, *E*), 5.76 (dt, *J* = 11.7, 7.6 Hz, 1H, *Z*), 2.70 – 2.50 (m, 4H, *Z* + m, 4H, *E*).

^{13}C NMR (101 MHz, $CDCl_3$) δ = 201.2 (*E*), 201.0 (*Z*), 146.3 (*Z*), 143.8 (*Z*), 134.1 (*Z*), 133.7 (*E*), 129.4 (*Z*), 129.2 (*E*), 128.3 (*Z*), 126.5 (*E*), 123.9 (*E*), 123.5 (*Z*), 43.4 (*Z*), 42.7 (*E*), 25.5 (*E*), 21.3 (*Z*) (two *ipso* signals from *E*-isomer were not allocated).

HRMS (ESI) m/z: $[M+H]^+$ Calcd for $[C_{11}H_{12}NO_2]^+$ 206.0812; found 206.0815.

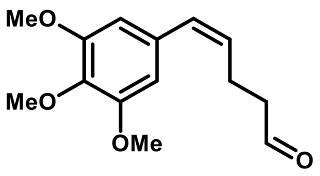

S2j

5-(3,4,5-Trimethoxyphenyl)pent-4-enal (**S2j**): The representative procedure F was followed using 3,4,5-trimethoxybenzaldehyde (1.50 g, 7.65 mmol), (4-ethoxy-4-oxobutyl)triphenylphosphonium bromide (4.55 g, 9.94 mmol) and *t*-BuOK (10 mL, 1.0 M in THF). A mixture of Z/E mixtures of ethyl and *t*-butyl esters (1.99 g) was obtained and used for the reduction according to the representative procedure H. After the reduction with $LiAlH_4$, crude alcohol (1.21 g) was directly used for Swern oxidation following the representative procedure I. Purification by flash column chromatography (SiO_2, hexanes:EtOAc = 5:1) yielded **S2j** (0.97 g, 50% for three steps, *Z:E* =7:1) as a yellow oil.

^{1}H NMR (300 MHz, $CDCl_3$, only singals for the *Z*-isomer are listed) δ = 9.76 (d, *J* = 1.5 Hz, 1H), 6.47 (s, 2H), 6.39 (dd, *J* = 11.8, 1.8 Hz, 2H), 5.55 (dt, *J* = 11.2, 6.8 Hz, 1H), 3.84 (bs, 9H), 2.75 – 2.62 (m, 2H), 2.61 – 2.51 (m, 2H).

^{13}C NMR (75 MHz, $CDCl_3$) δ = 201.6, 153.0, 137.0, 132.7, 130.3, 129.8, 105.9, 60.9, 56.1, 43.8, 21.4.

HRMS (ESI) m/z: $[M+H]^+$ Calcd for $[C_{14}H_{19}O_4]^+$ 251.1278; found 251.1274.

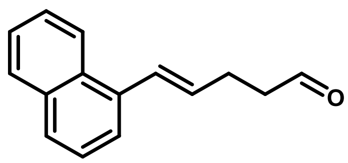

S2g

5-(Naphthalen-1-yl)pent-4-enal (**S2k**): The representative procedure I was followed using 5-(1-naphthalenyl)-4-penten-1-ol[18] (0.44 g, 2.38 mmol, mixture of *E/Z* isomers). Purification by flash column chromatography (SiO_2, hexanes:EtOAc = 5:1) yielded **S2k** (0.17 g, 32 %, *E:Z* =6:1) as a yellow oil.

^{1}H NMR (300 MHz, $CDCl_3$, only data for the *E*-isomer are listed) δ = 9.89 (s, 1H), 8.18 – 8.17 (m, 1H), 7.94 – 7.83 (m, 1H), 7.80 (d, *J* = 8.2 Hz, 1H), 7.61 – 7.51 (m, 3H), 7.48 (d, *J* = 7.8 Hz, 1H), 7.21 (d, *J* = 15.5 Hz, 1H), 6.33 – 6.17 (m, 1H), 2.81 – 2.61 (m, 4H).

^{13}C NMR (75 MHz, $CDCl_3$, only data for the *E*-isomer are listed) δ = 201.8, 135.1, 133.6, 131.5, 131.1, 128.5, 128.4, 127.7, 126.0, 125.8, 125.7, 123.8, 123.7, 43.4, 25.9.

HRMS (ESI) m/z: $[M+H]^+$ Calcd for $[C_{15}H_{15}O]^+$ 211.1117; found 211.1118.

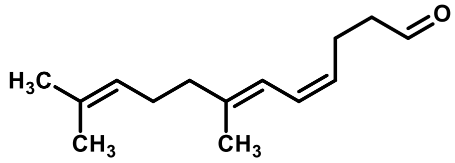

S2o

(4*Z*,6*E*)-7,11-dimethyldodeca-4,6,10-trienal (**S2o**): The representative procedure F was followed using geranial (0.85 g, 5.58 mmol), (4-ethoxy-4-oxobutyl)triphenylphosphonium bromide (7.26 g, 7.26 mmol) and *t*-BuOK (11.2 mL, 1.0 M in THF). A mixture of *Z/E* mixtures of ethyl and *t*-butyl esters (0.98 g) was obtained and used for the reduction according to the representative procedure H. After the reduction with $LiAlH_4$, crude alcohol (0.76 g) was directly used for Swern oxidation following the representative procedure I. Purification by flash column chromatography (SiO_2, hexanes:EtOAc = 10:1) yielded **S2o** (0.32 g, 27% for three steps, *Z:E* =4.5:1) as a yellow oil.

^{1}H NMR (400 MHz, $CDCl_3$, only signals from the *Z*-isomer are listed) δ = 9.79 (s, 1H), 6.24 (t, *J* = 11.1 Hz, 1H), 6.07 (d, *J* = 11.3 Hz, 1H), 5.38 – 5.24 (m, 1H), 5.11 (bs, 1H), 2.53 (bs, 4H), 2.11 (bs, 4H), 1.76 (s, 3H), 1.69 (s, 3H), 1.62 (s, 3H).

^{13}C NMR (101 MHz, $CDCl_3$) δ = 202.1, 140.0, 131.7, 126.6, 126.1, 123.9, 119.4, 43.8, 40.3, 26.6, 25.7, 20.3, 17.7, 16.5.

HRMS (ESI) m/z: $[M+H]^+$ Calcd for $[C_{14}H_{23}O]^+$ 207.1743; found 207.1747.

10. ^{1}H-, ^{13}C-NMR and selected 2D-NMR spectra for new compounds

^{1}H-NMR of compound (*E*)-1a (300 MHz, CDCl$_3$)

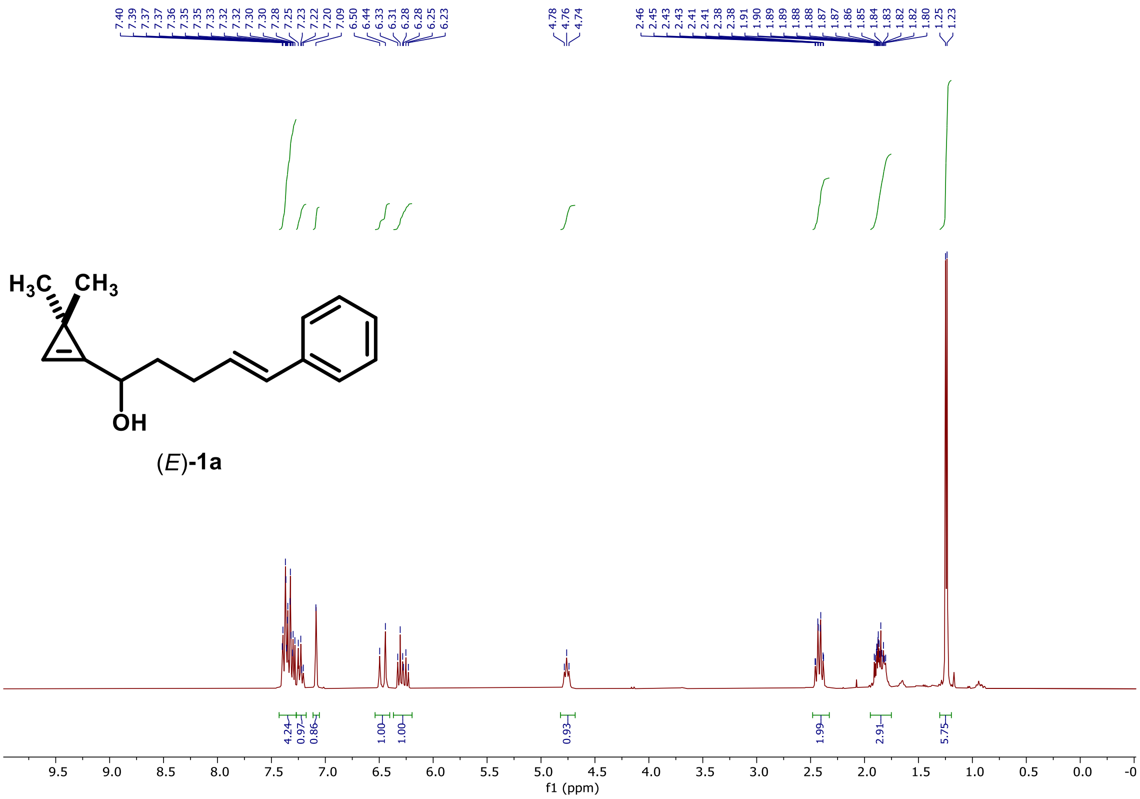

^{13}C-NMR of compound (*E*)-1a (75 MHz, CDCl$_3$)

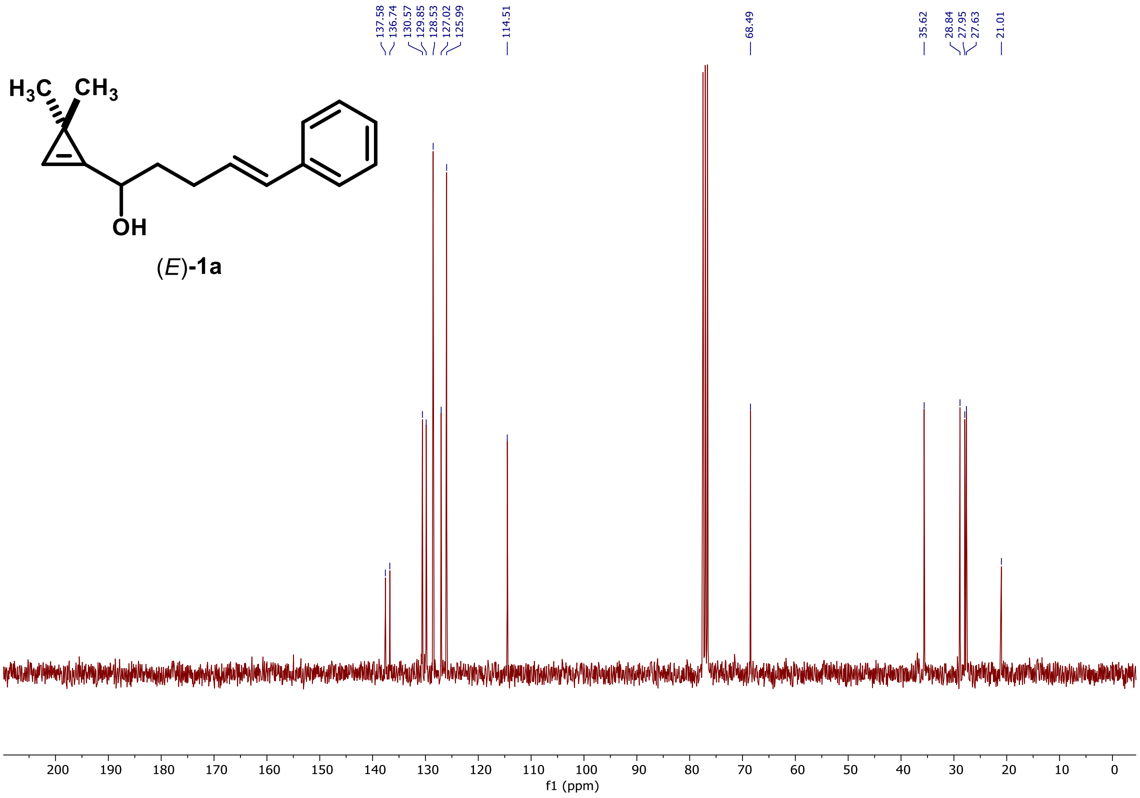

^{1}H-NMR of compound (*Z*)-1a (300 MHz, $CDCl_3$)

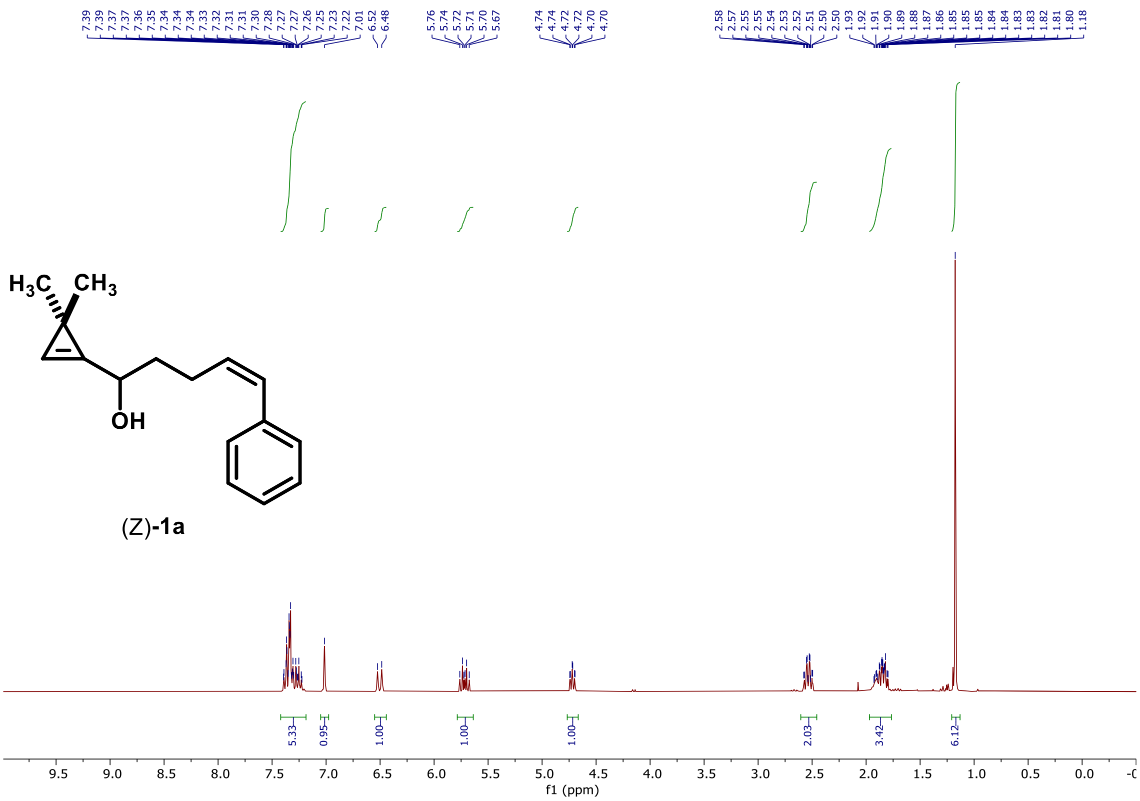

^{13}C-NMR of compound (*E*)-1a (75 MHz, $CDCl_3$)

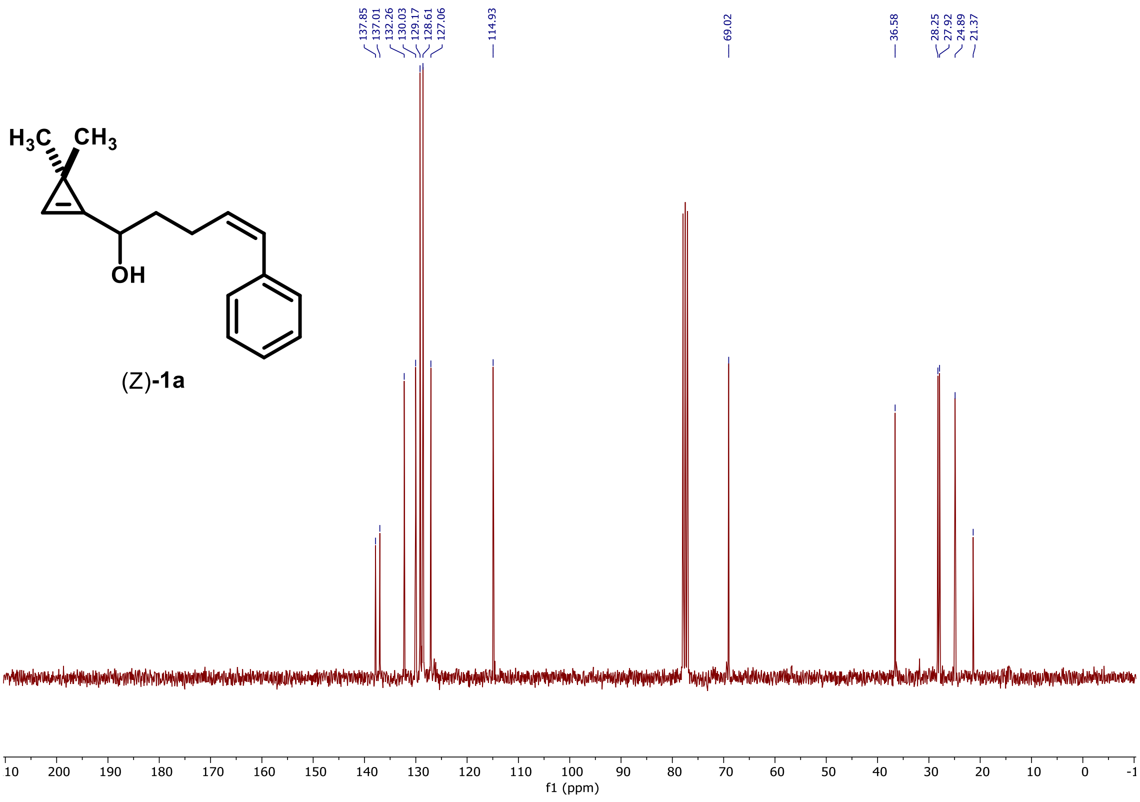

^{1}H-NMR of compound 1b (300 MHz, $CDCl_3$)

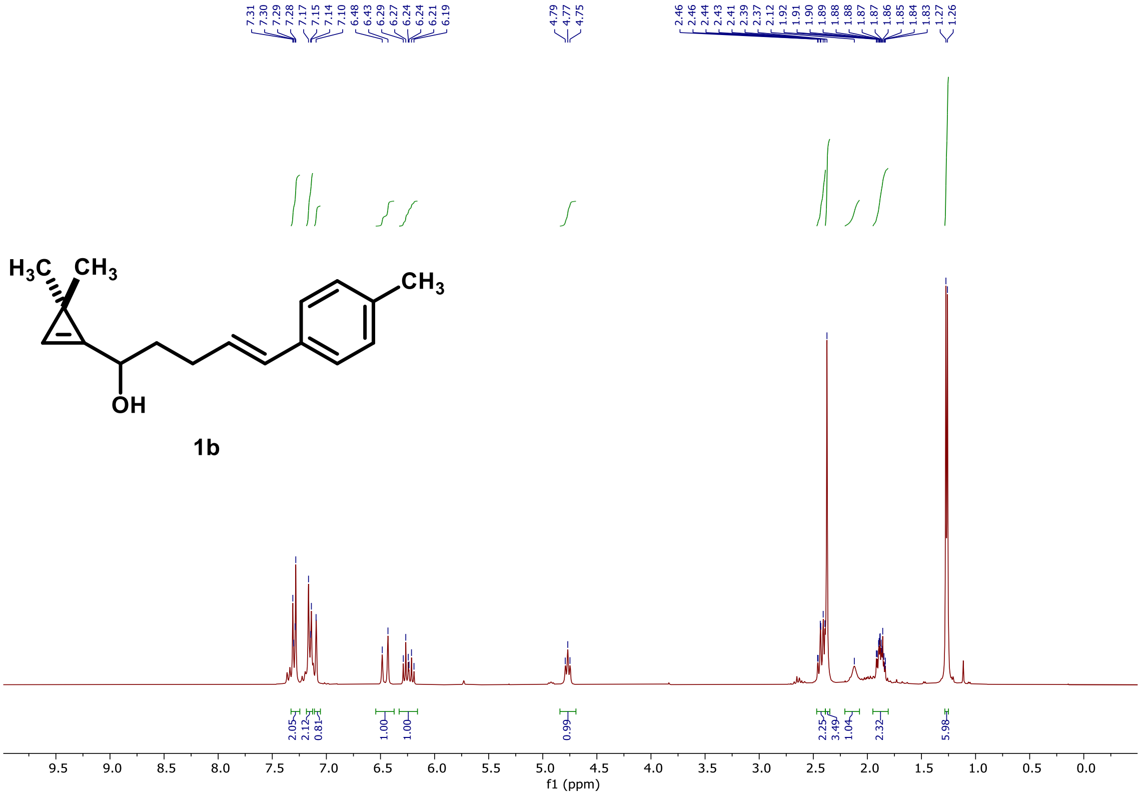

^{13}C-NMR of compound 1b (75 MHz, $CDCl_3$)

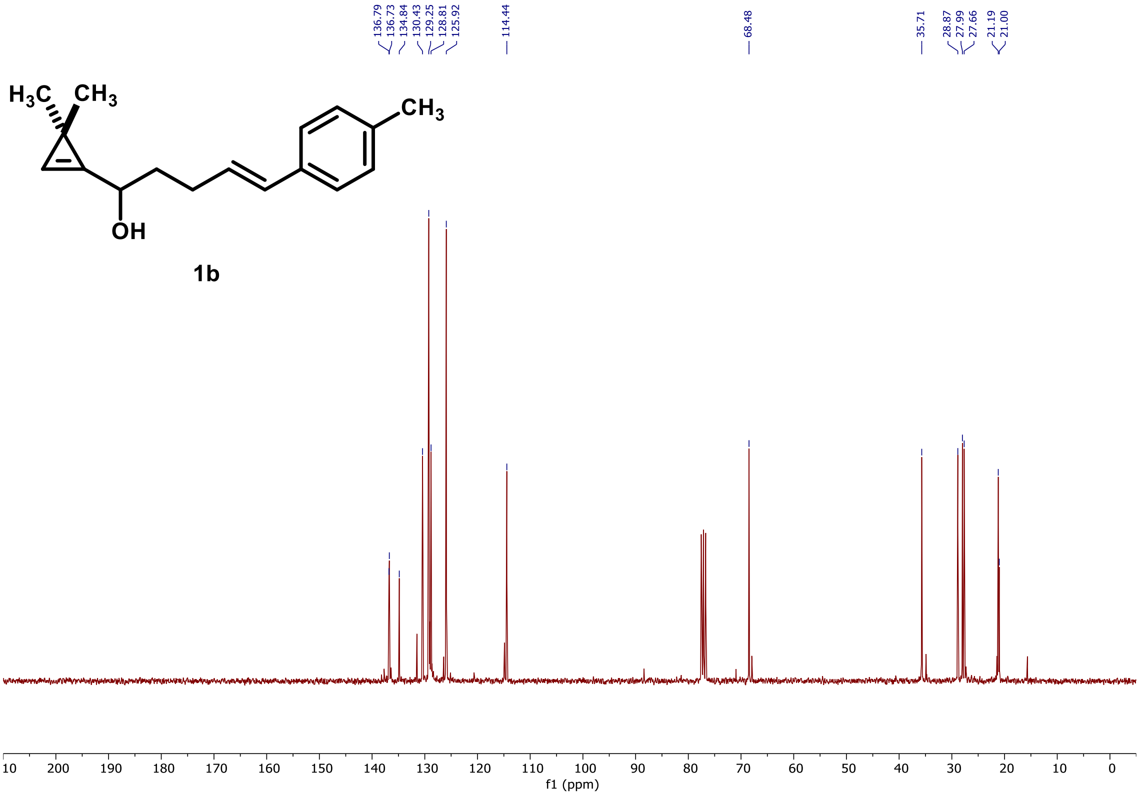

^{1}H-NMR of compound 1c (300 MHz, $CDCl_3$)

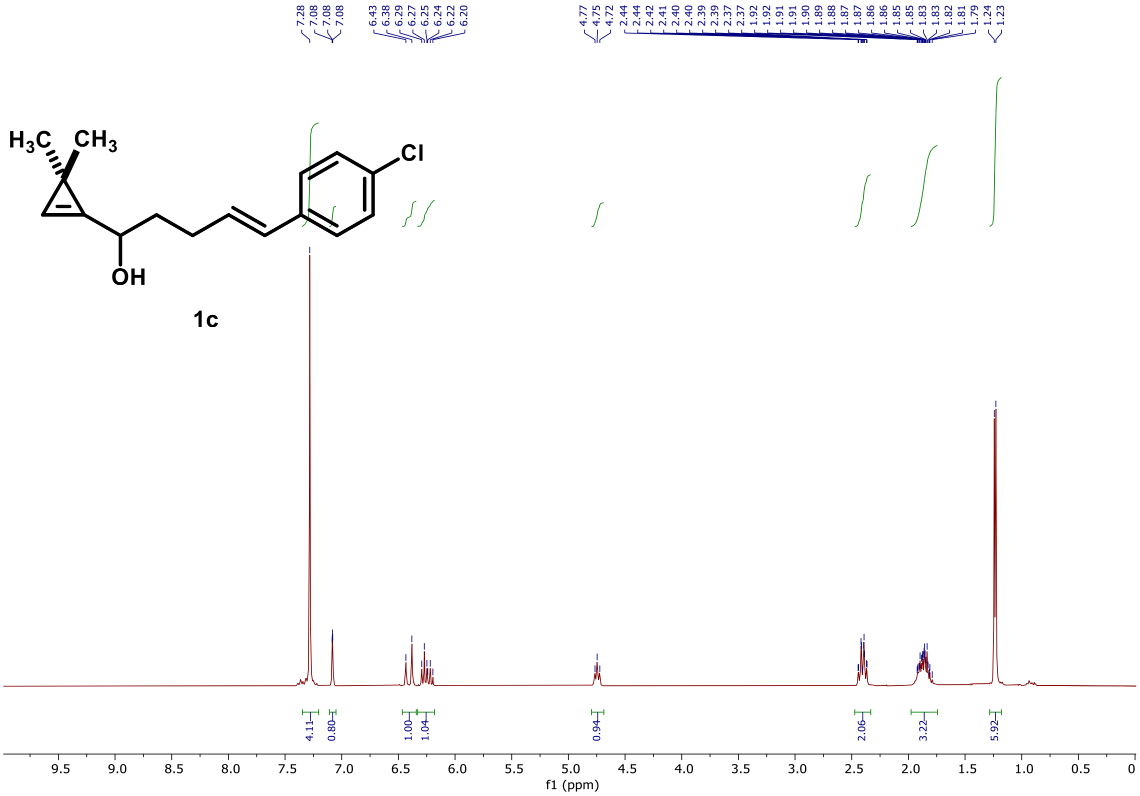

^{13}C-NMR of compound 1c (75 MHz, $CDCl_3$)

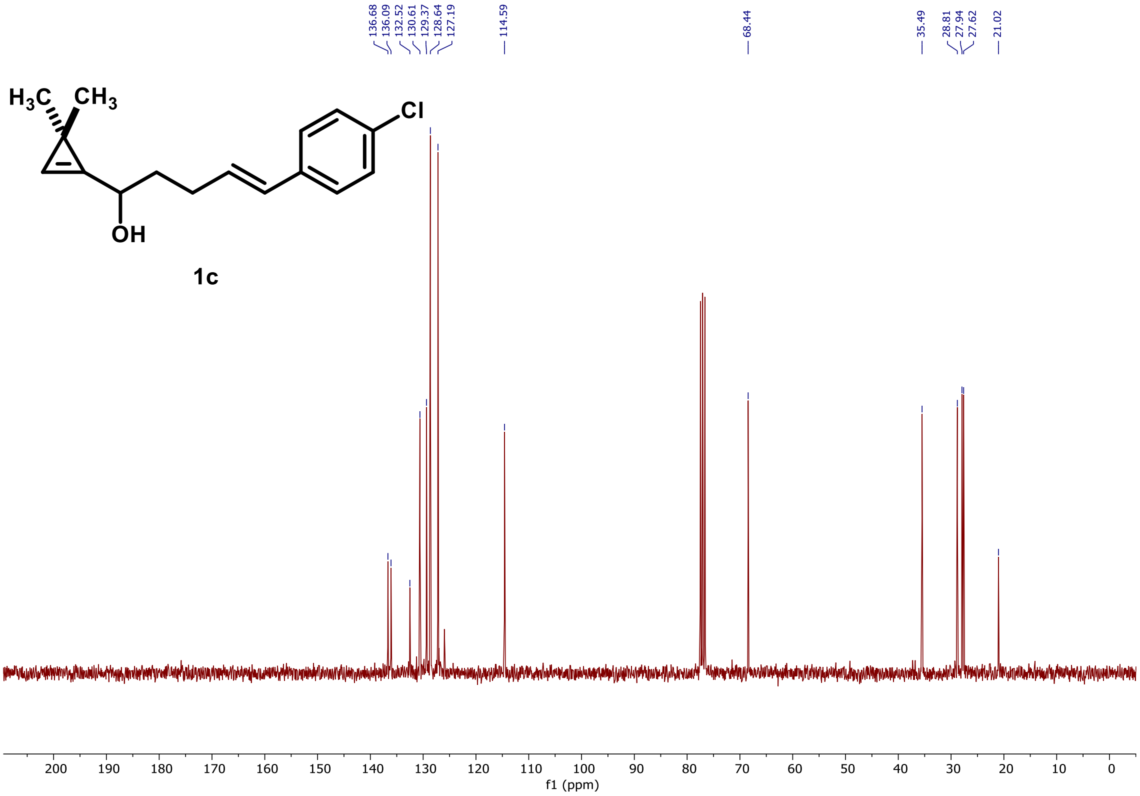

^{1}H-NMR of compound 1d (300 MHz, $CDCl_3$)

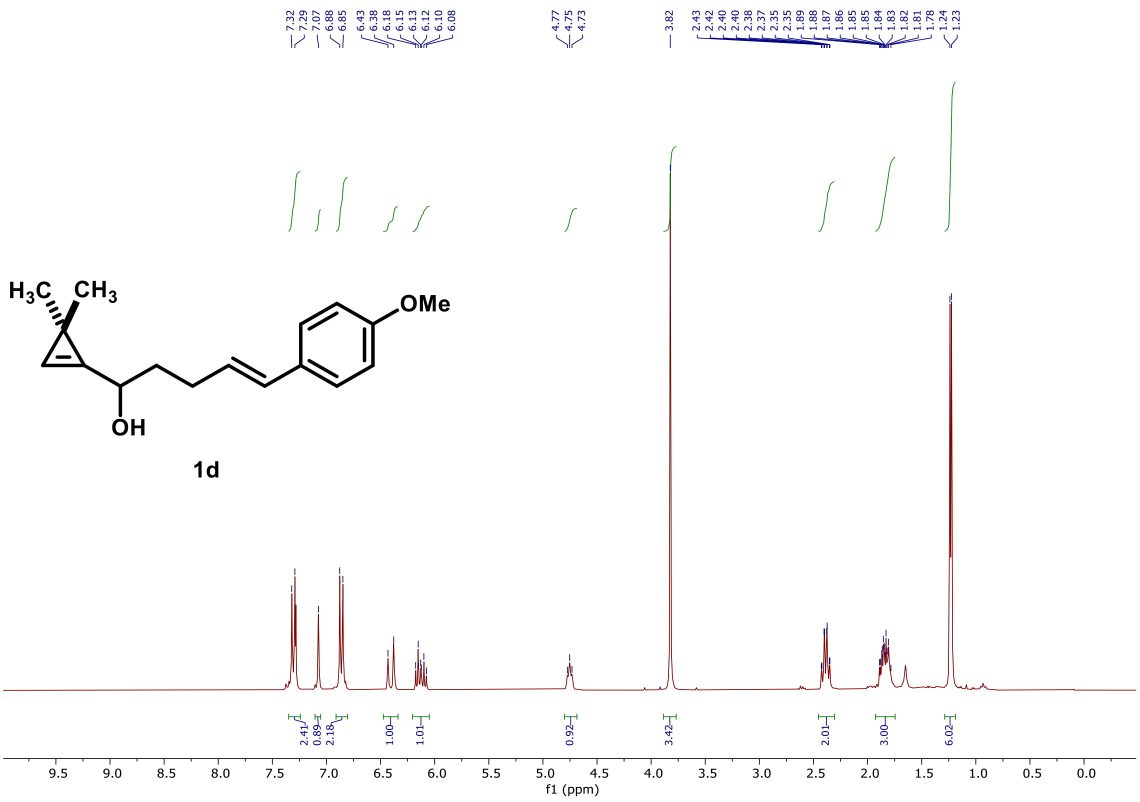

^{13}C-NMR of compound 1d (75 MHz, $CDCl_3$)

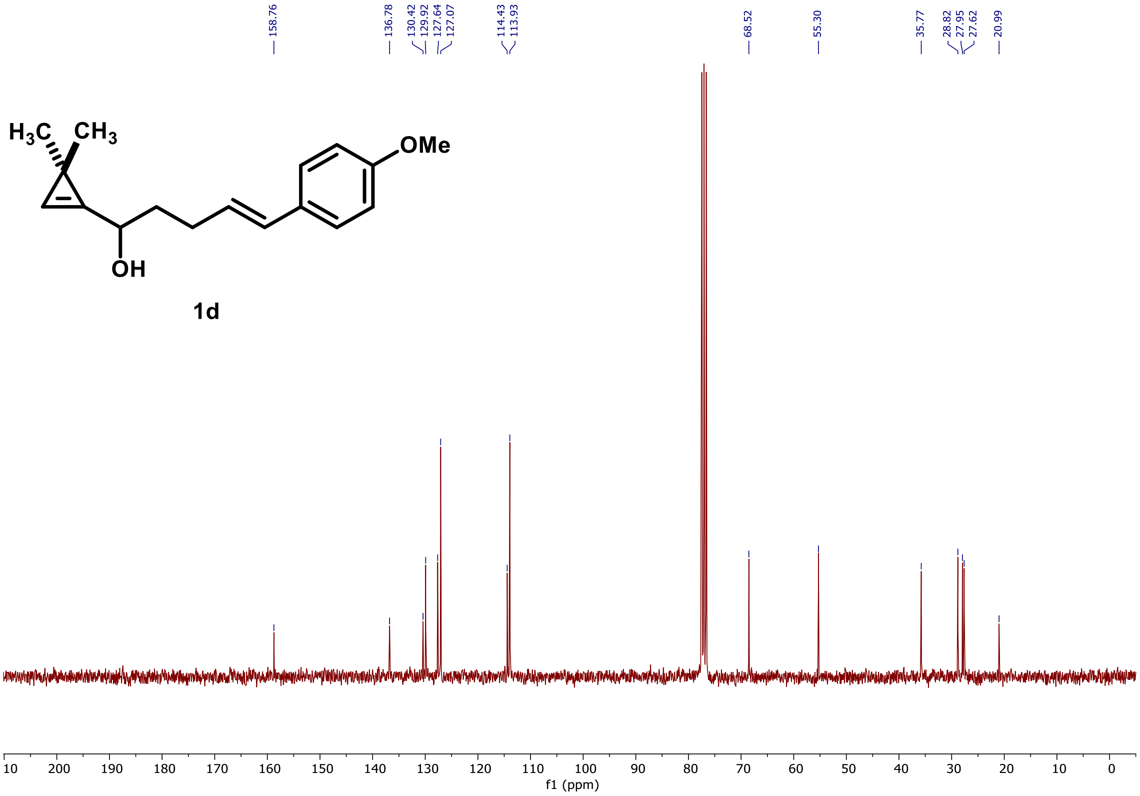

^{1}H-NMR of compound 1e (300 MHz, $CDCl_3$)

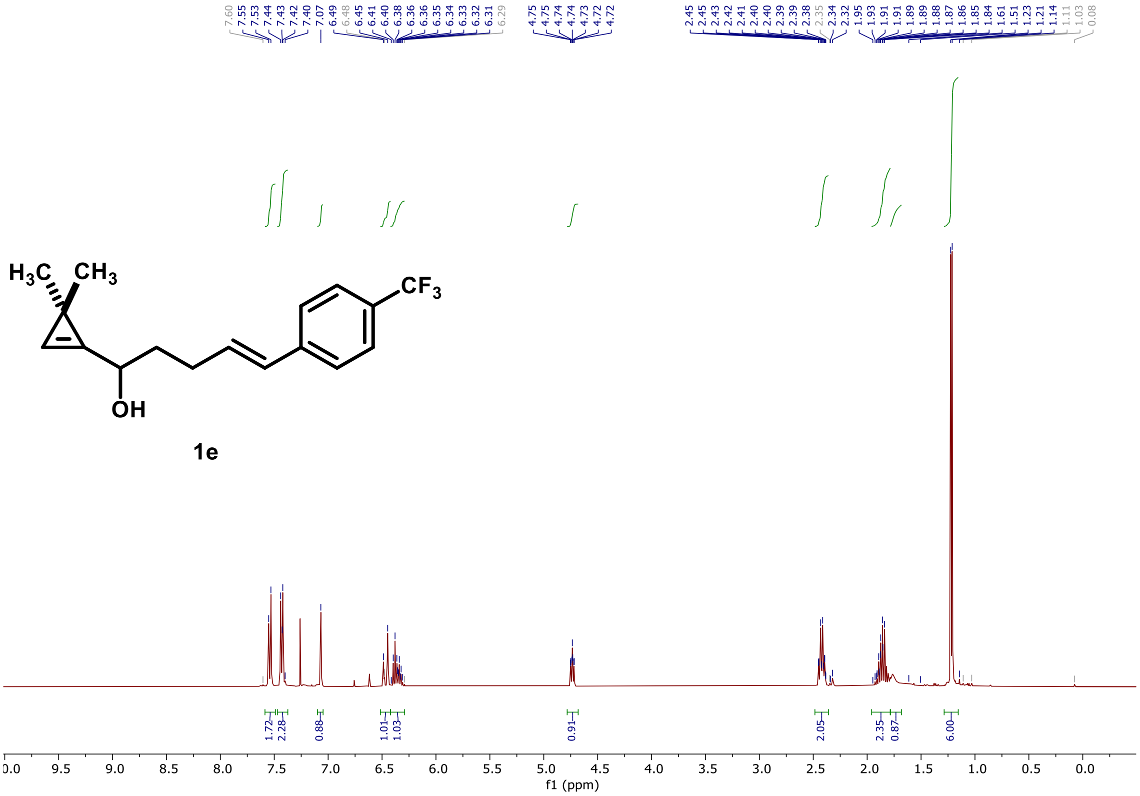

^{13}C-NMR of compound 1e (75 MHz, $CDCl_3$)

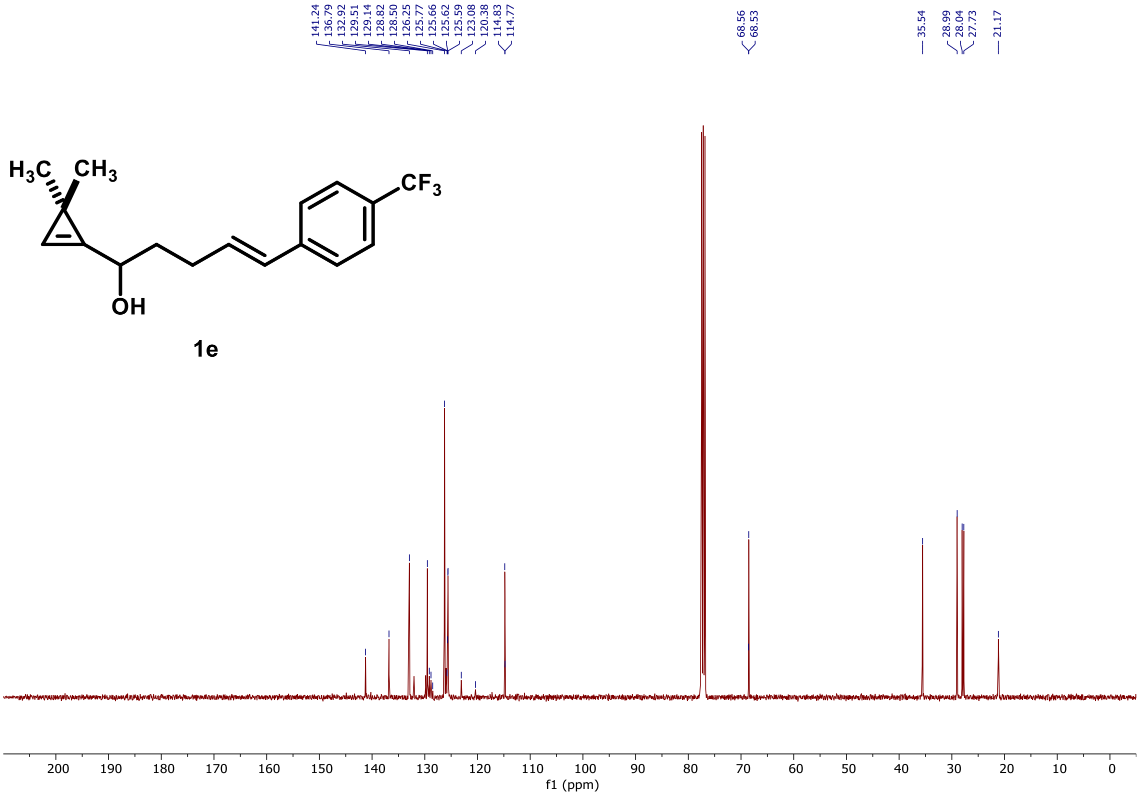

^{1}H-NMR of compound 1f (300 MHz, $CDCl_3$)

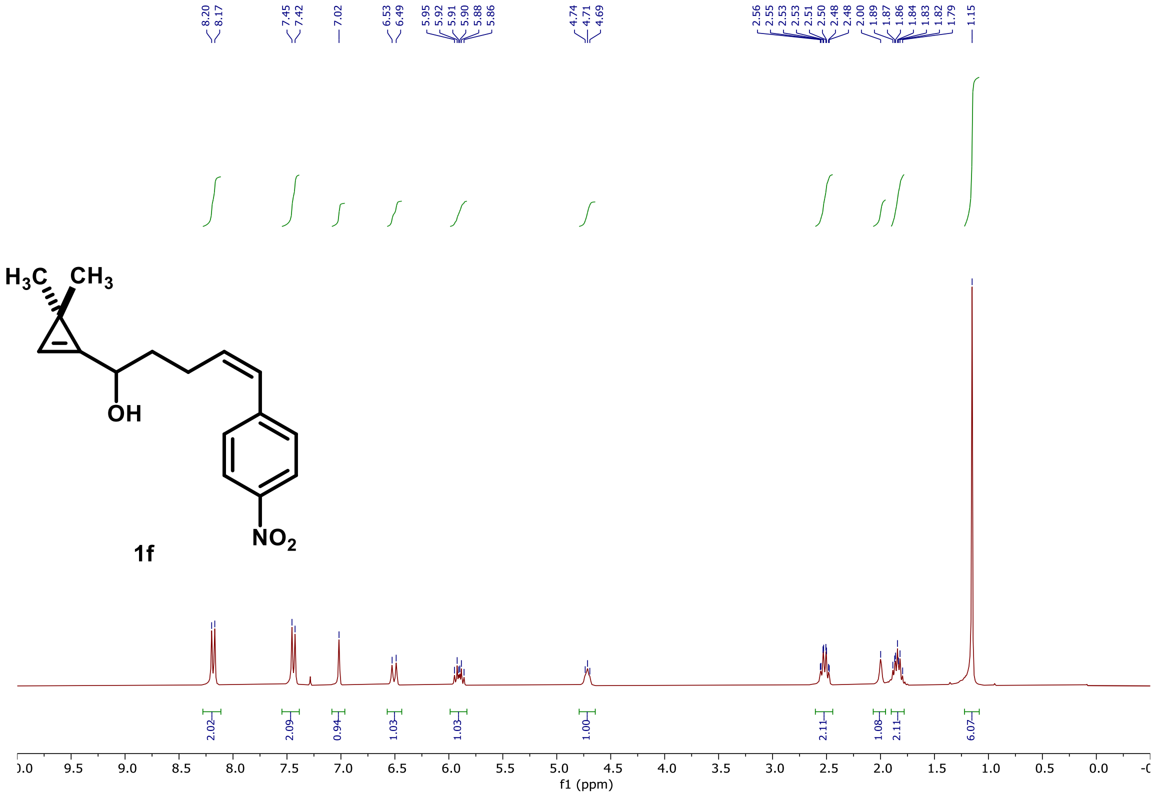

^{13}C-NMR of compound 1f (101 MHz, $CDCl_3$)

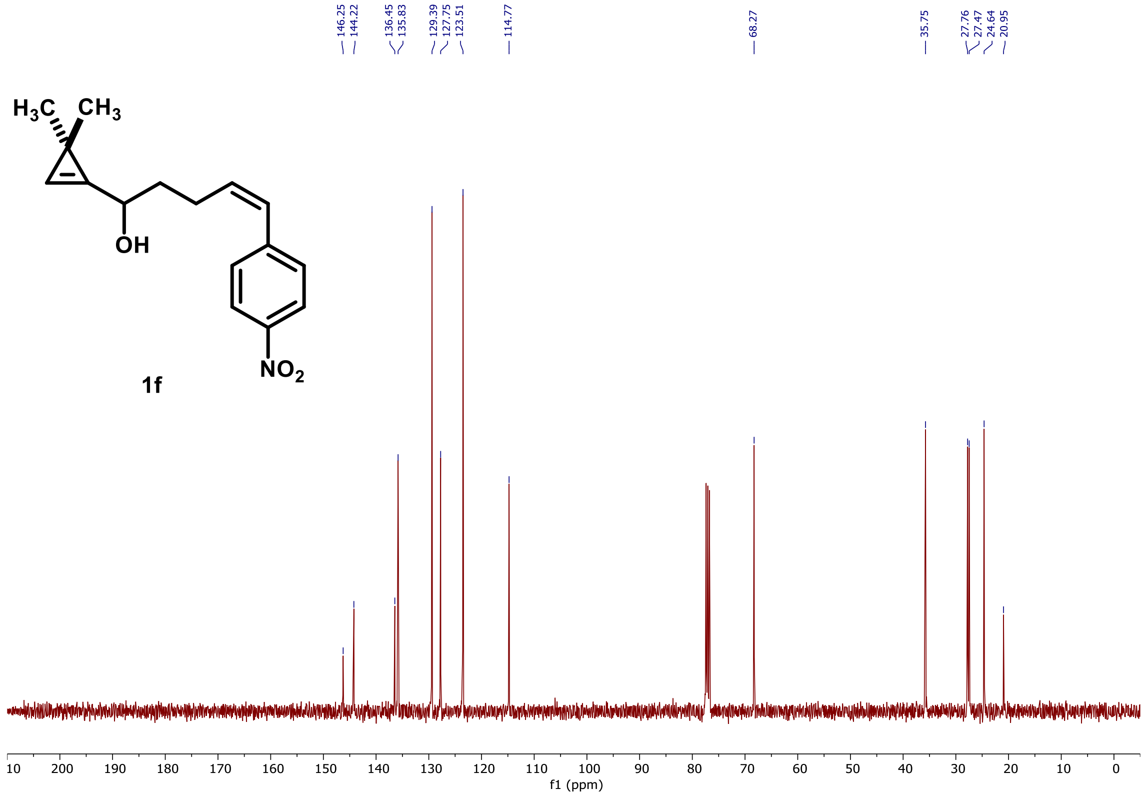

^{1}H-NMR of compound 1g (300 MHz, $CDCl_3$)

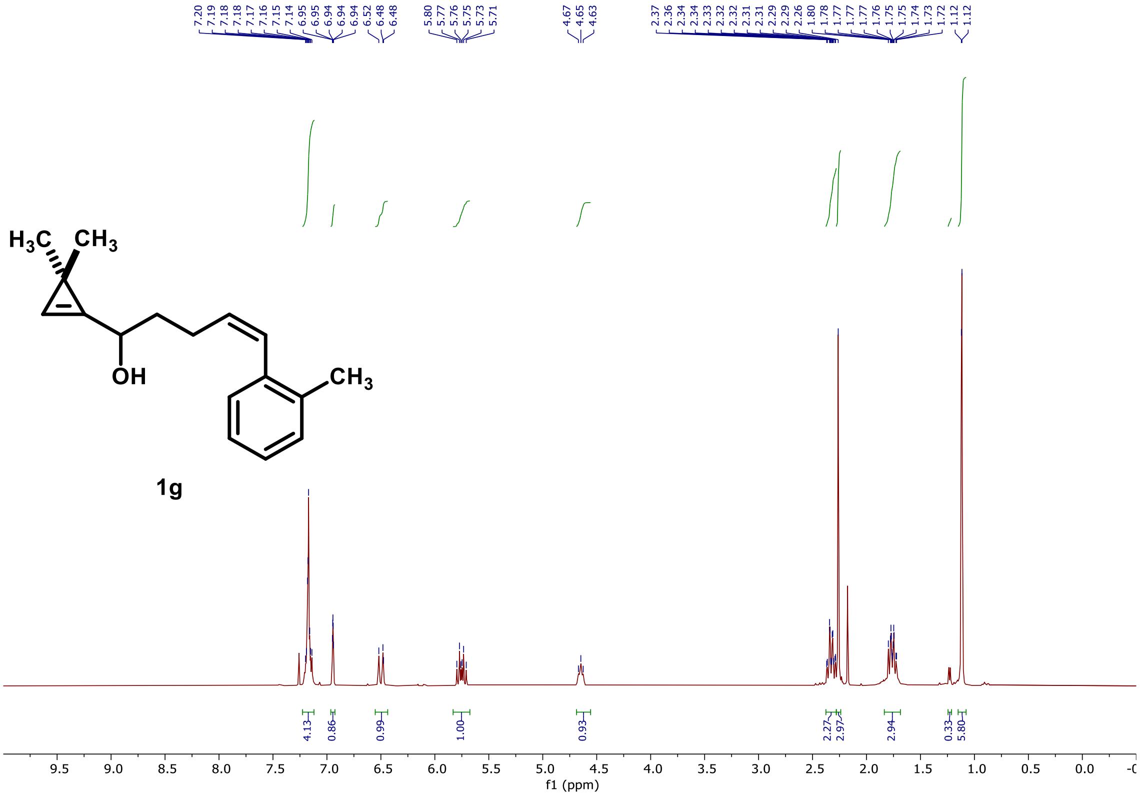

^{13}C-NMR of compound 1g (75 MHz, $CDCl_3$)

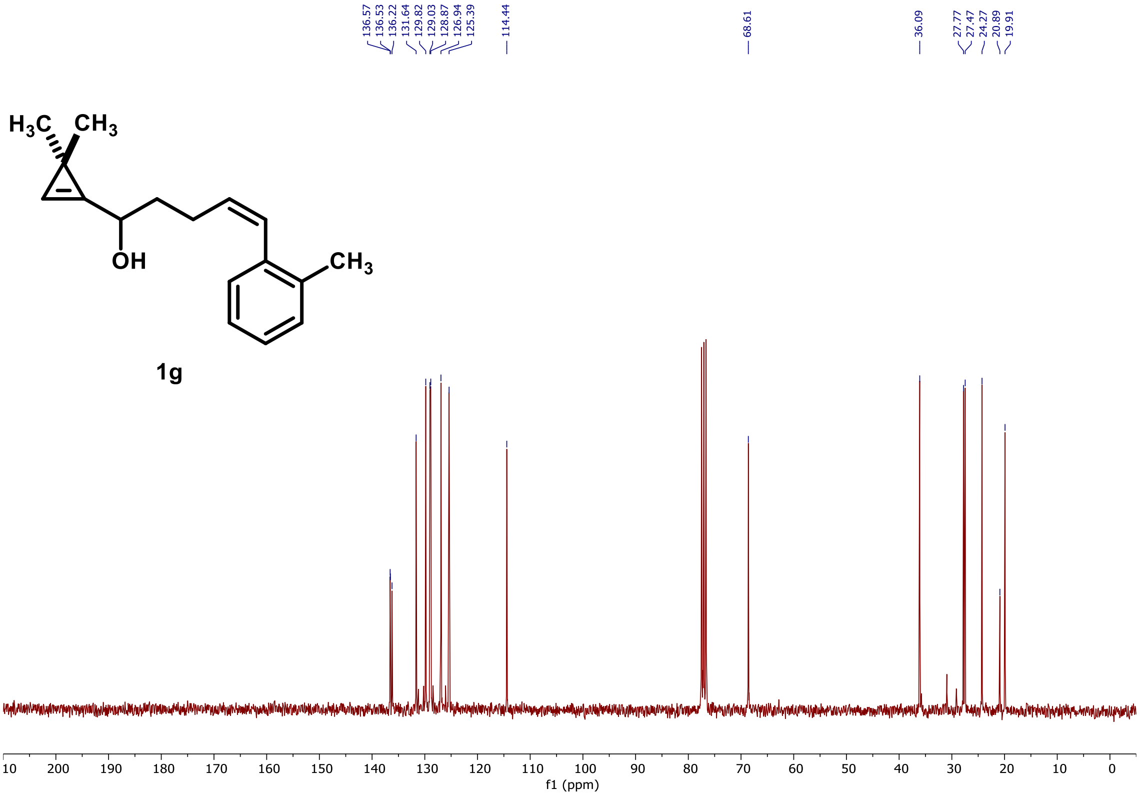

^{1}H-NMR of compound 1h (300 MHz, $CDCl_3$)

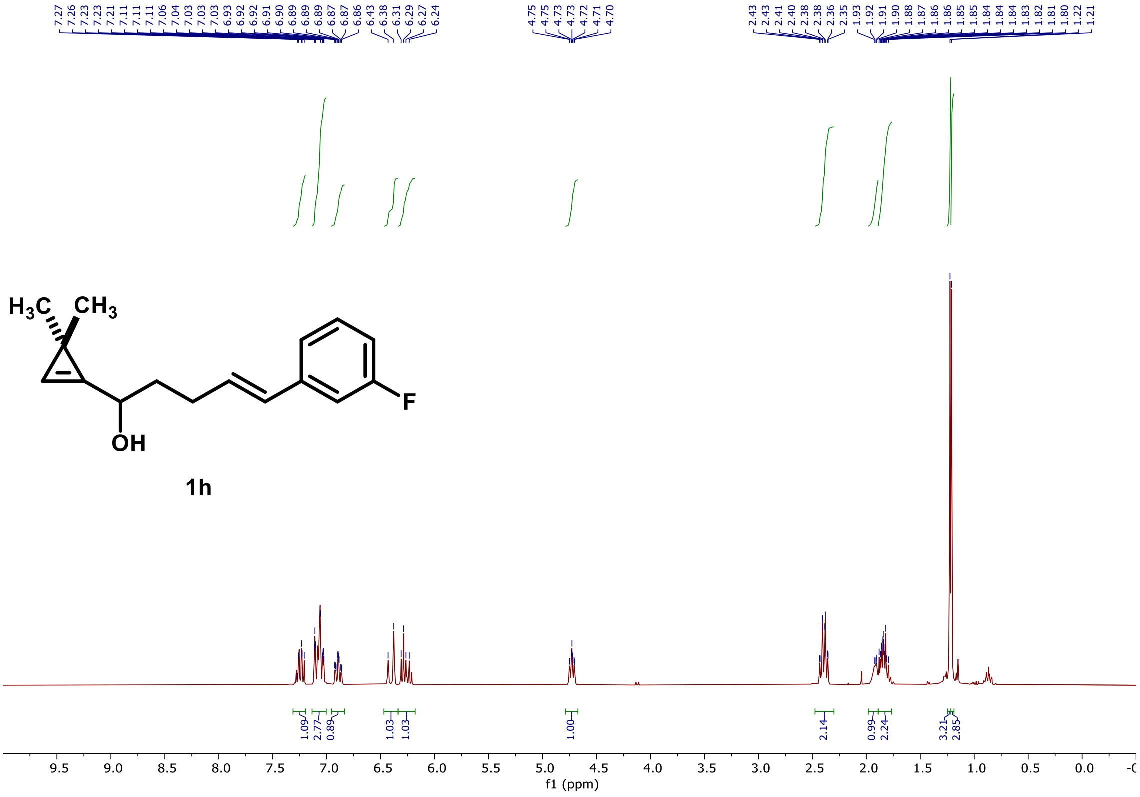

^{13}C-NMR of compound 1h (75 MHz, $CDCl_3$)

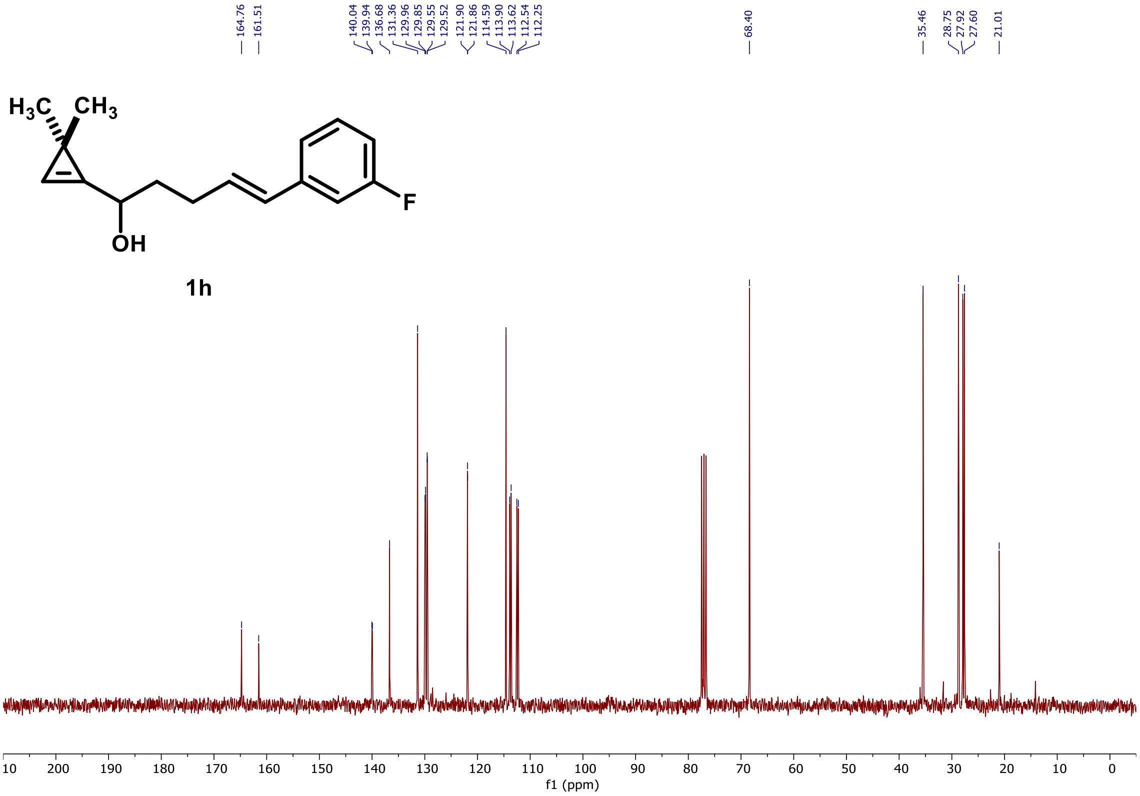

^{1}H-NMR of compound 1h (300 MHz, $CDCl_3$)

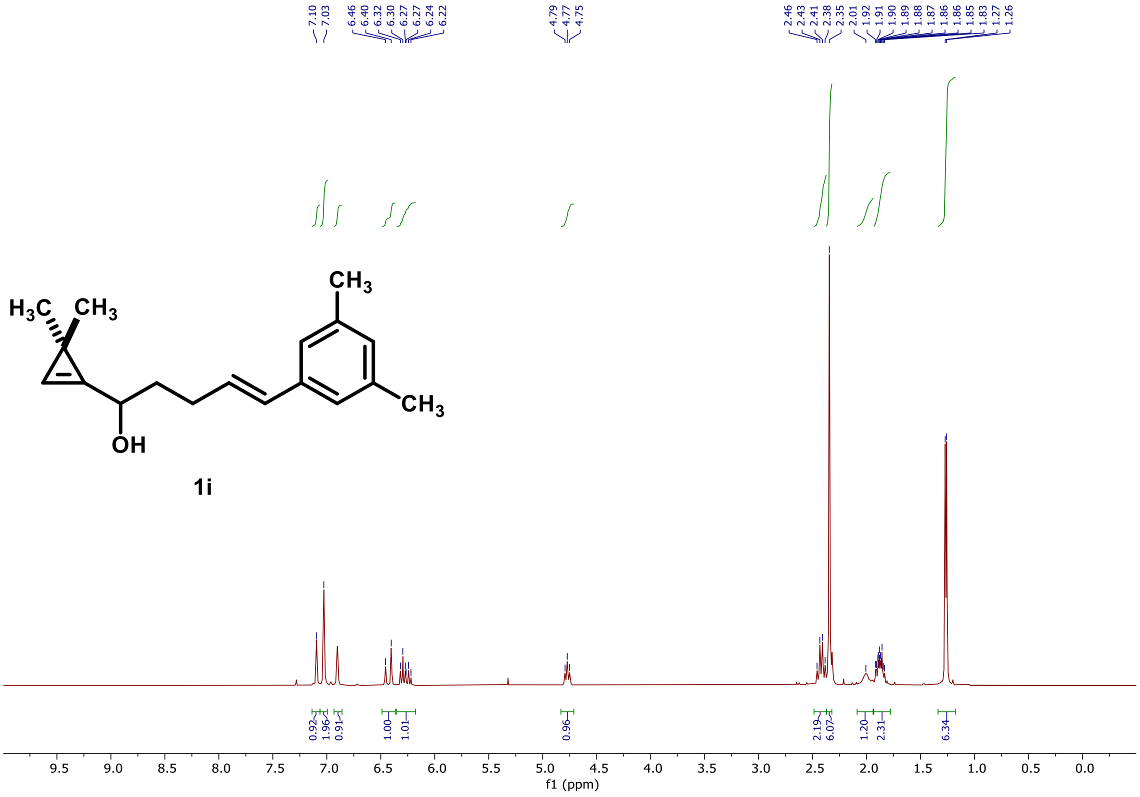

^{13}C-NMR of compound 1i (75 MHz, $CDCl_3$)

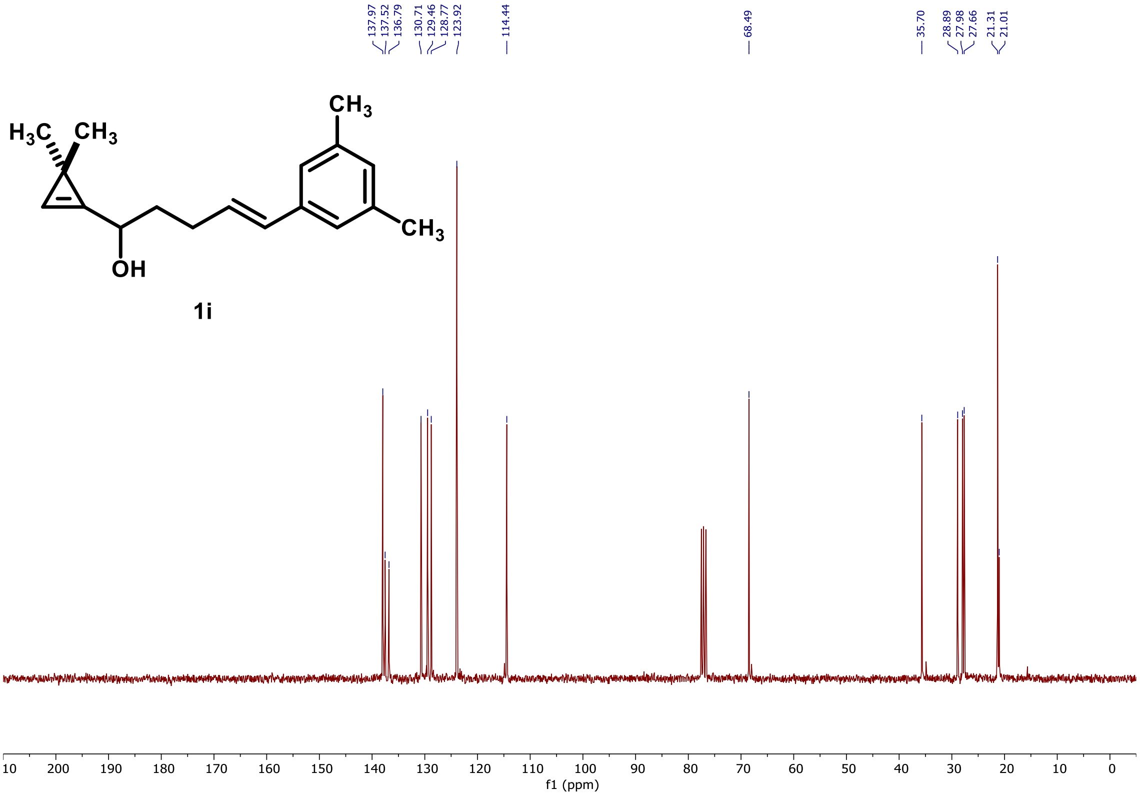

^{1}H-NMR of compound 1j (300 MHz, $CDCl_3$)

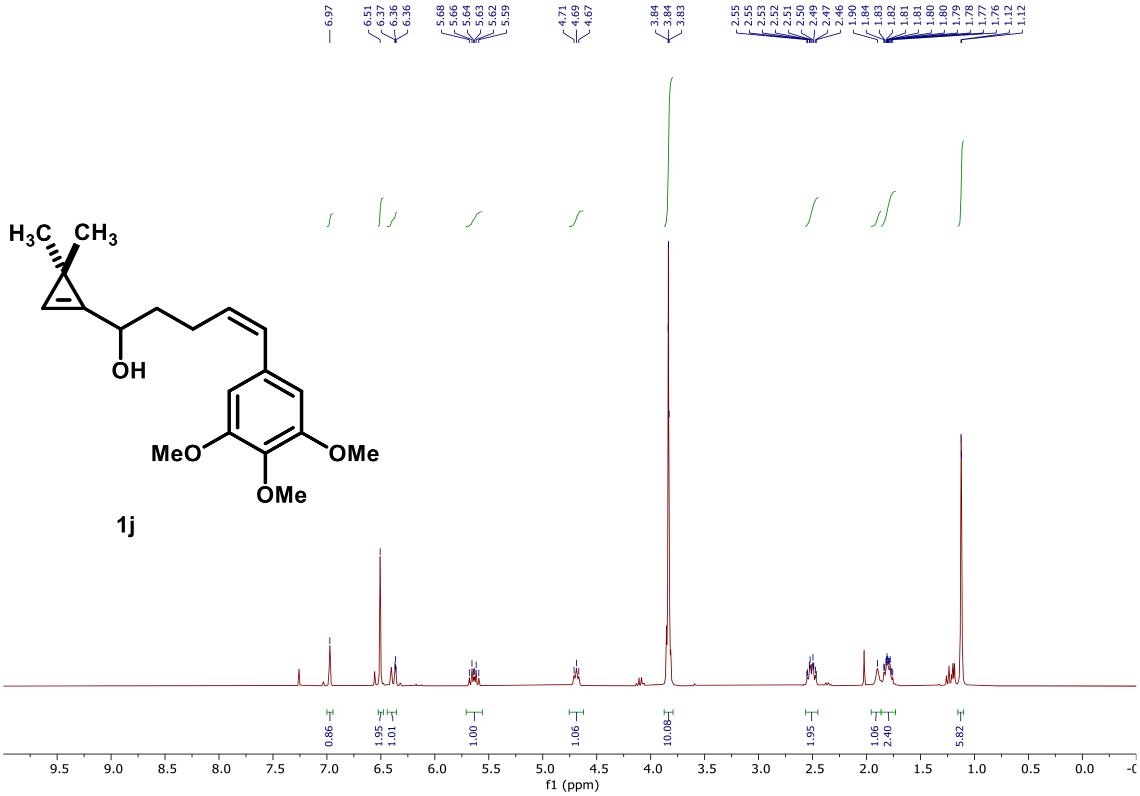

^{13}C-NMR of compound 1j (75 MHz, $CDCl_3$)

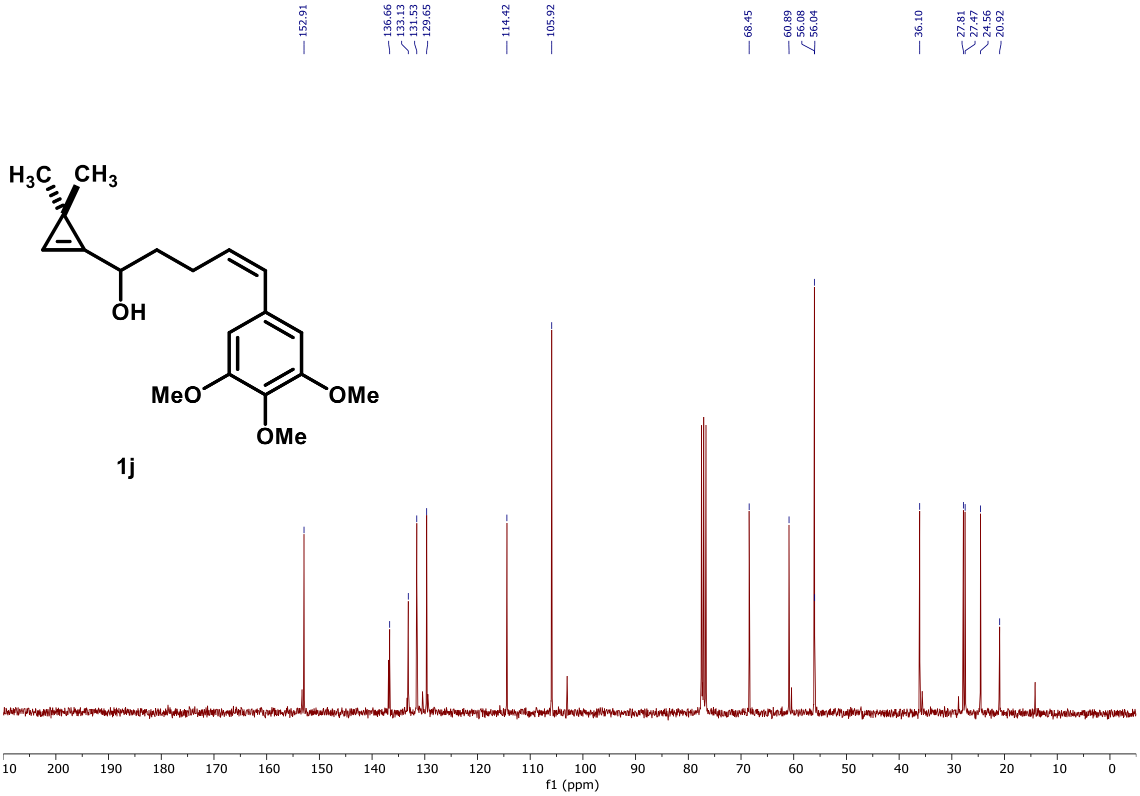

^{1}H-NMR of compound 1k (300 MHz, $CDCl_3$)

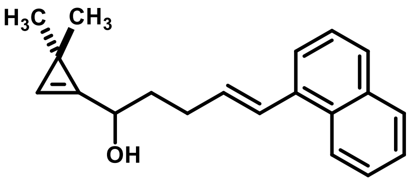

1k

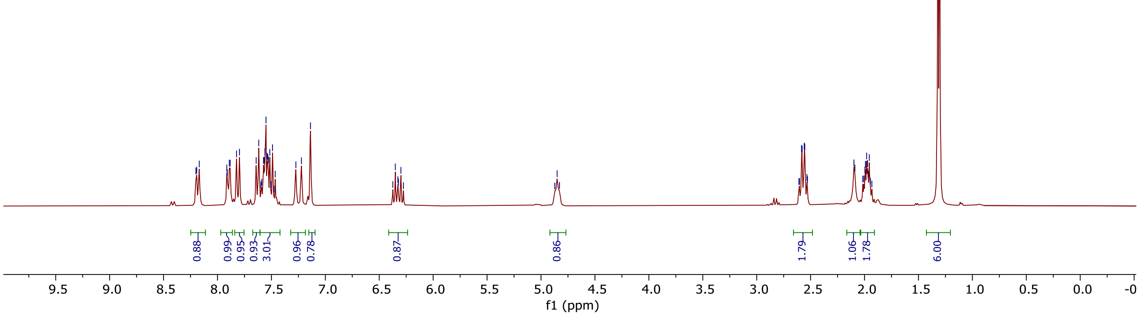

^{13}C-NMR of compound 1k (75 MHz, $CDCl_3$)

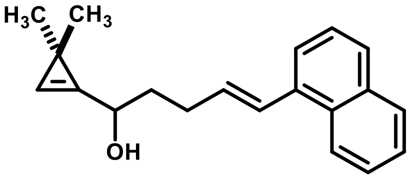

1k

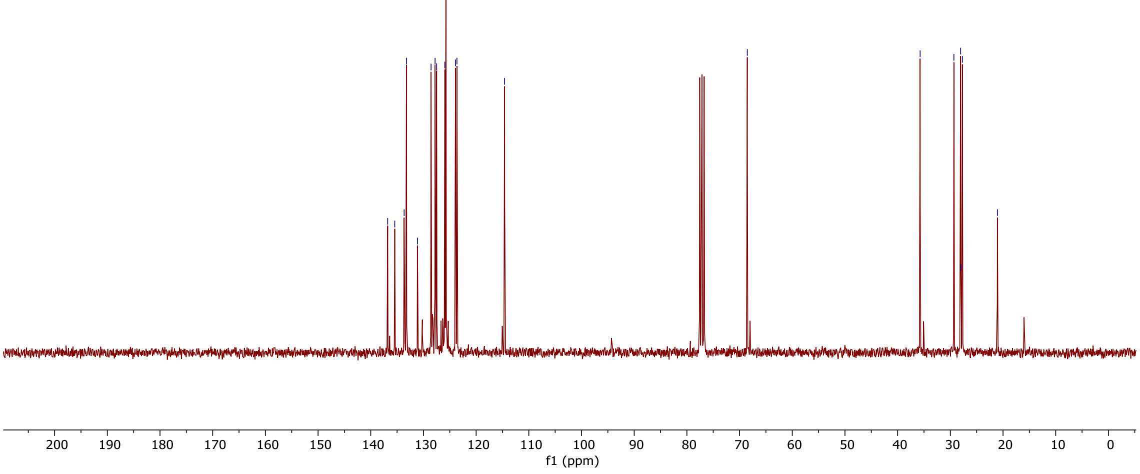

^{1}H-NMR of compound 1l (300 MHz, $CDCl_3$)

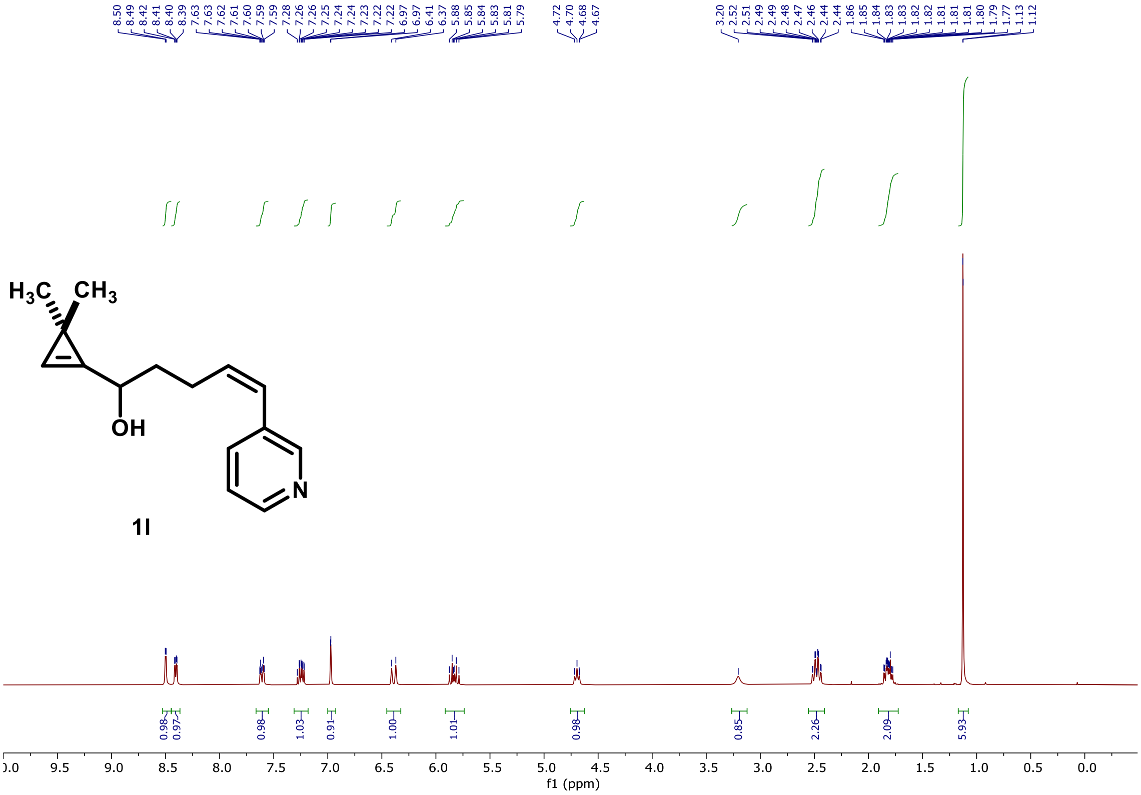

^{13}C-NMR of compound 1l (75 MHz, $CDCl_3$)

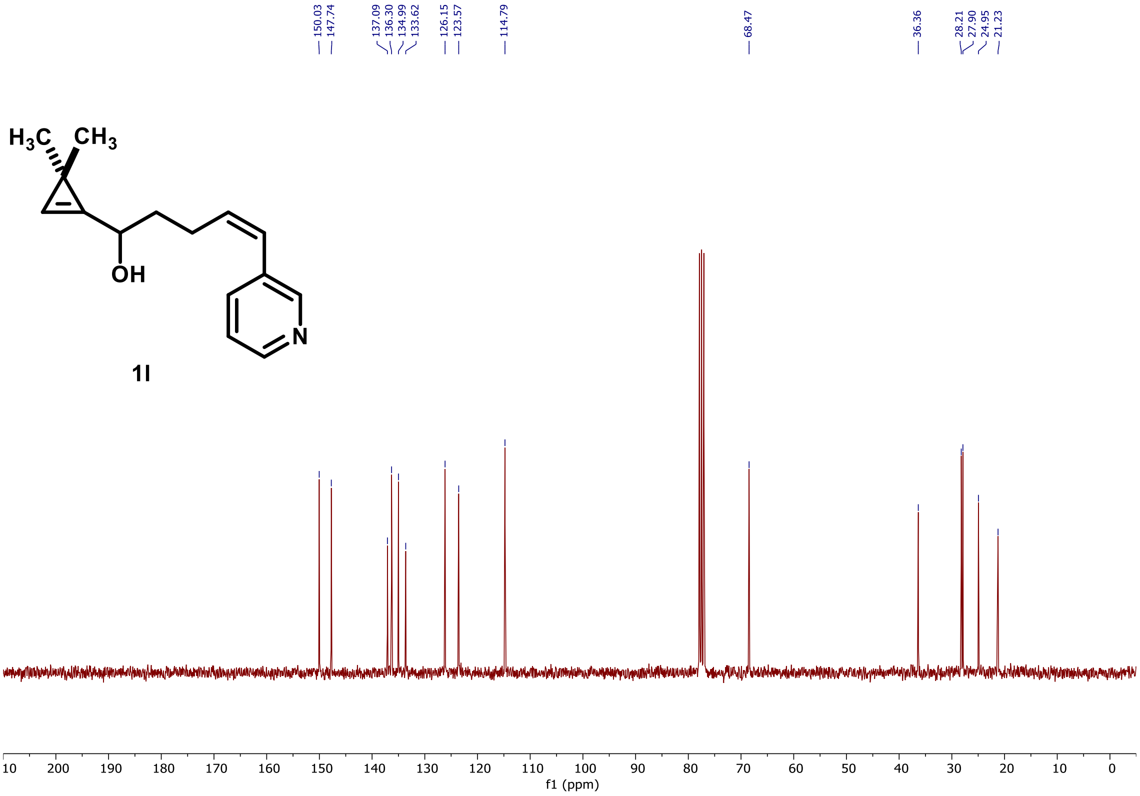

^{1}H-NMR of compound 1m (300 MHz, $CDCl_3$)

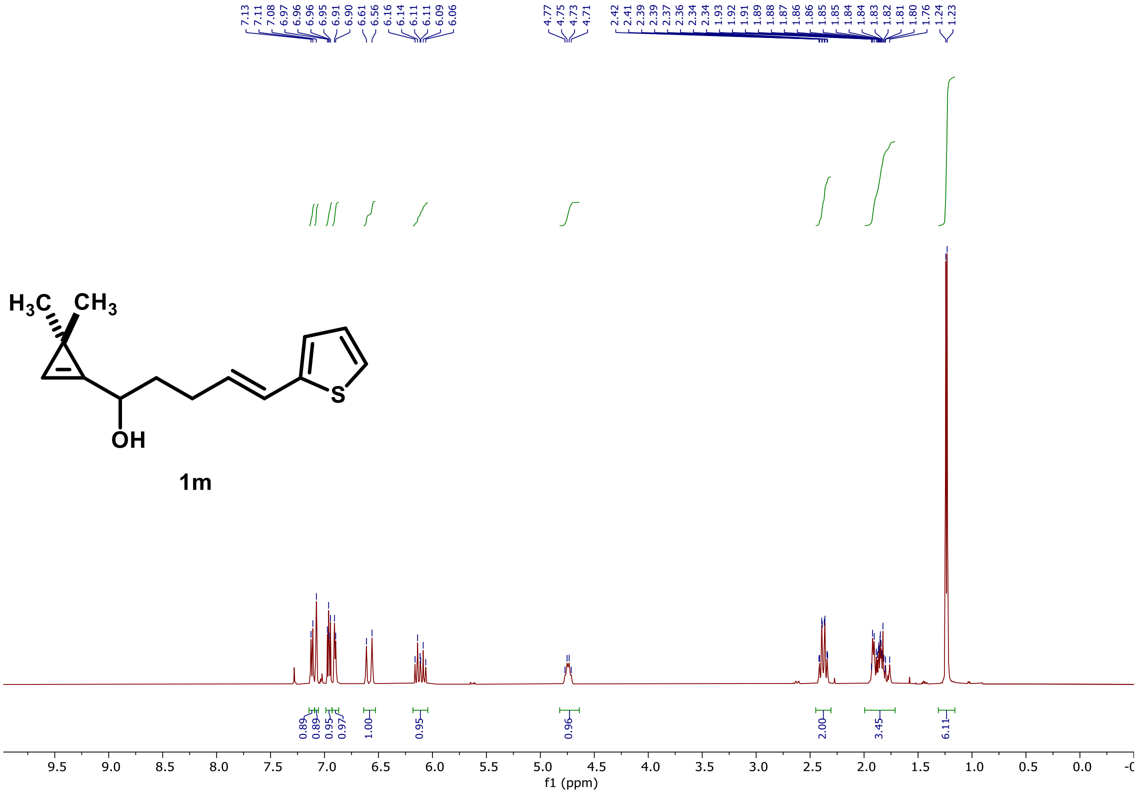

^{13}C-NMR of compound 1m (75 MHz, $CDCl_3$)

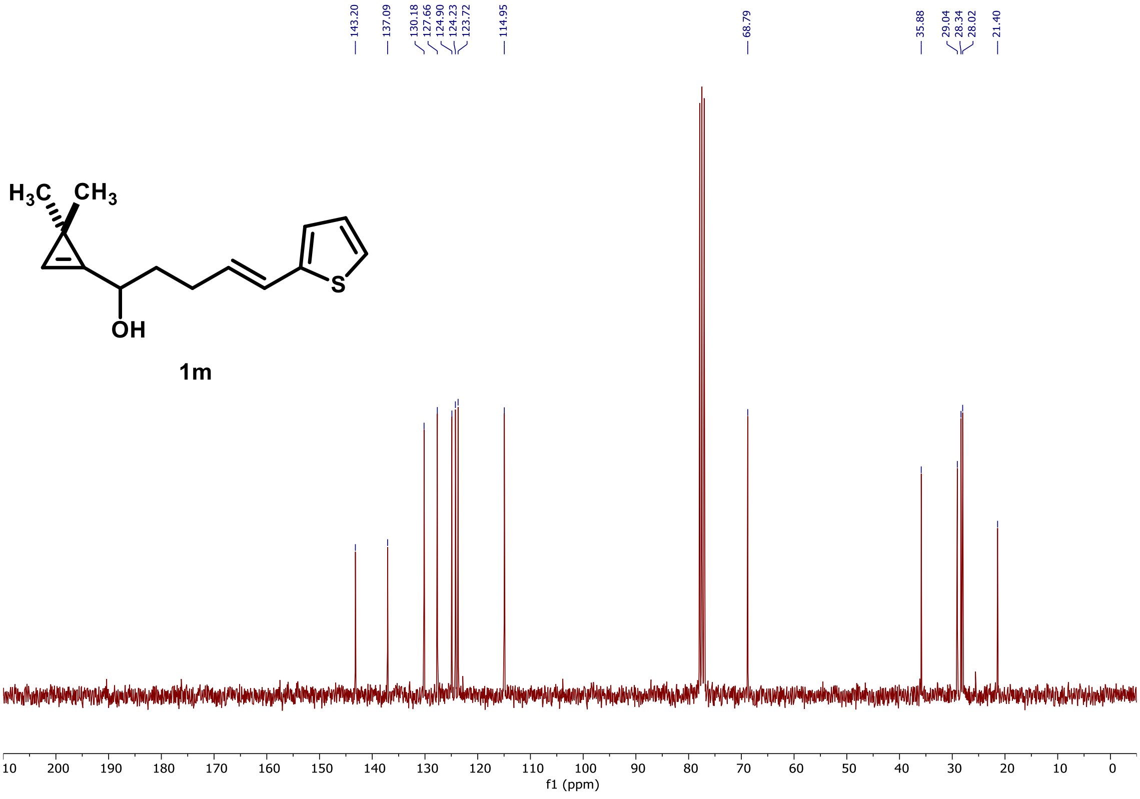

^{1}H-NMR of compound 1n (300 MHz, $CDCl_3$)

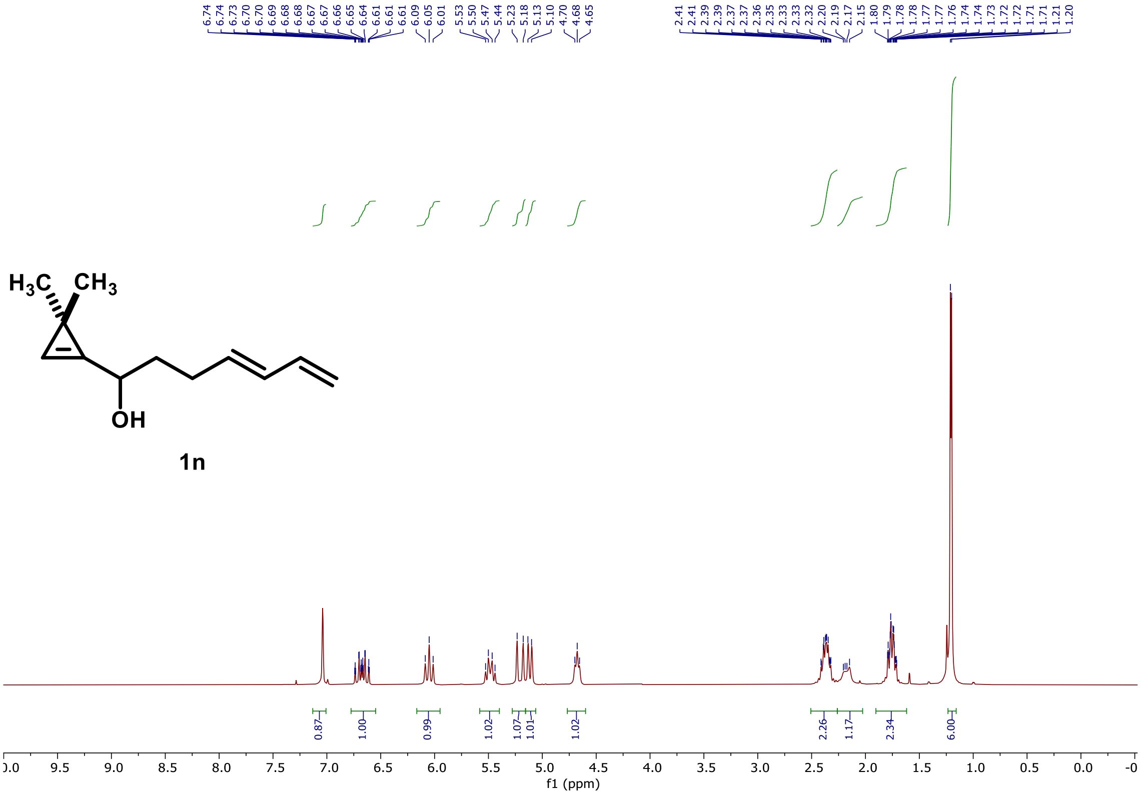

^{13}C-NMR of compound 1n (101 MHz, $CDCl_3$)

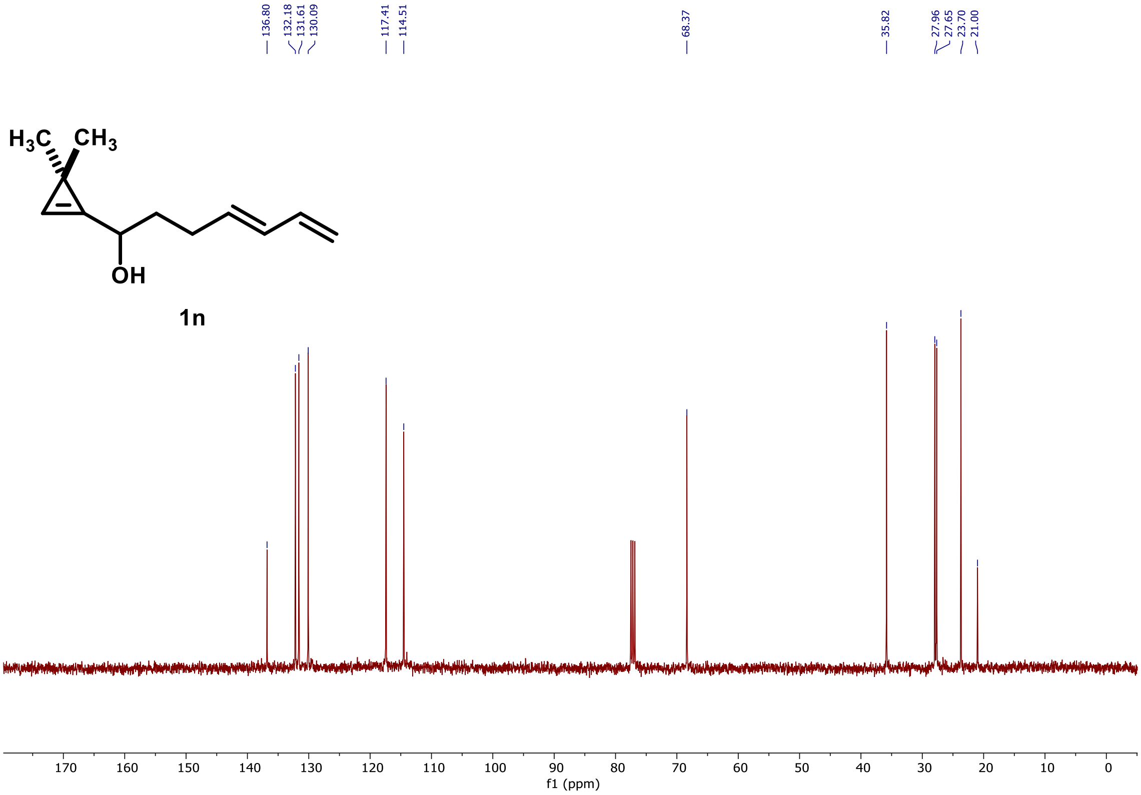

^{1}H-NMR of compound 1o (300 MHz, $CDCl_3$)

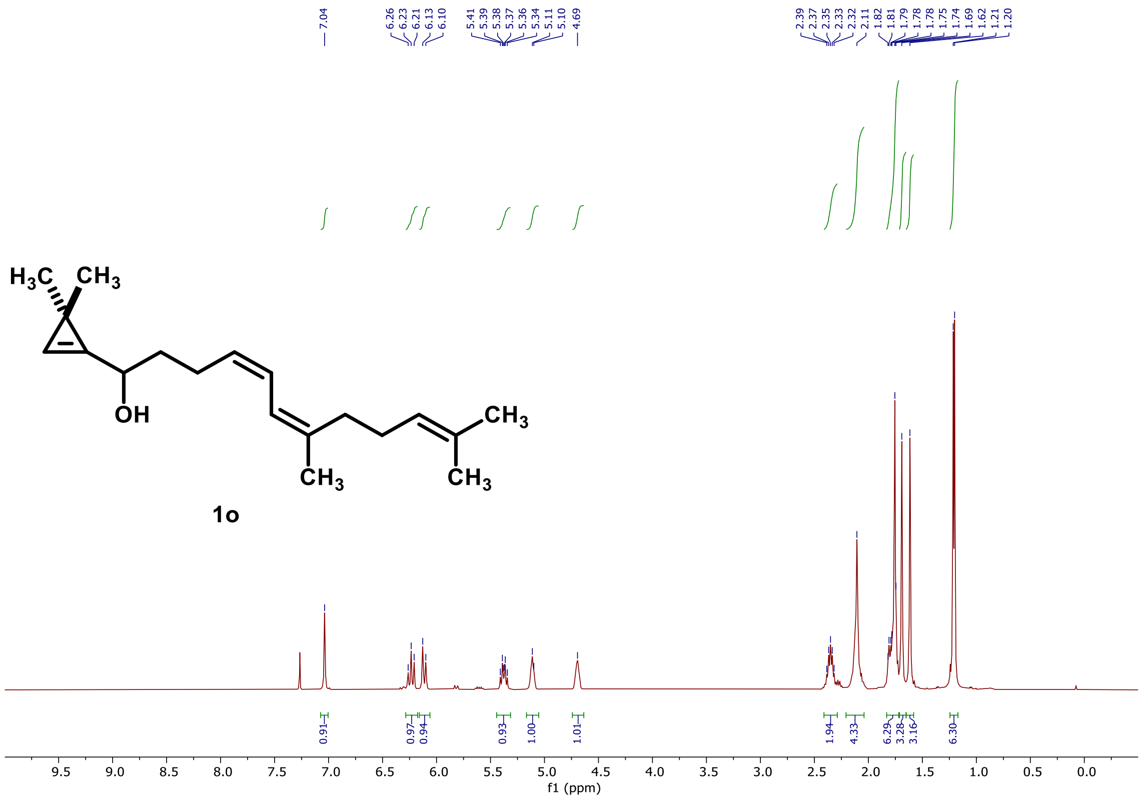

^{13}C-NMR of compound 1o (101 MHz, $CDCl_3$)

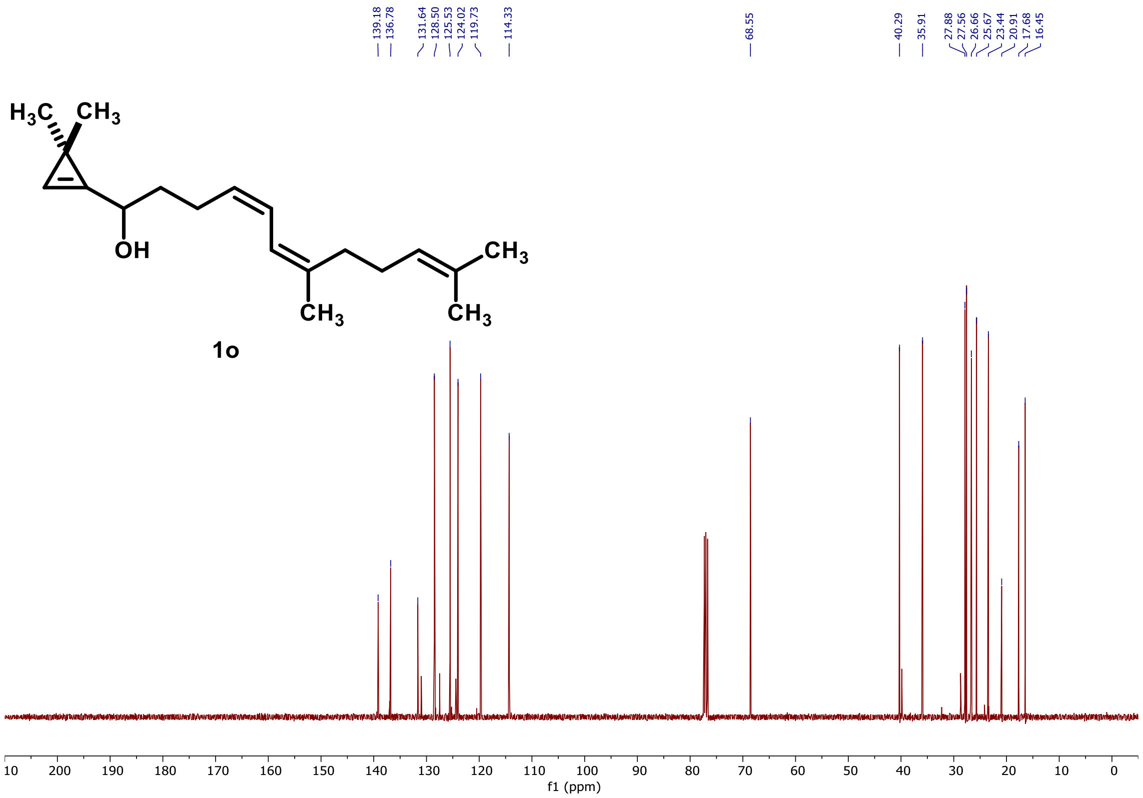

^{1}H-NMR of compound S2p (300 MHz, $CDCl_3$)

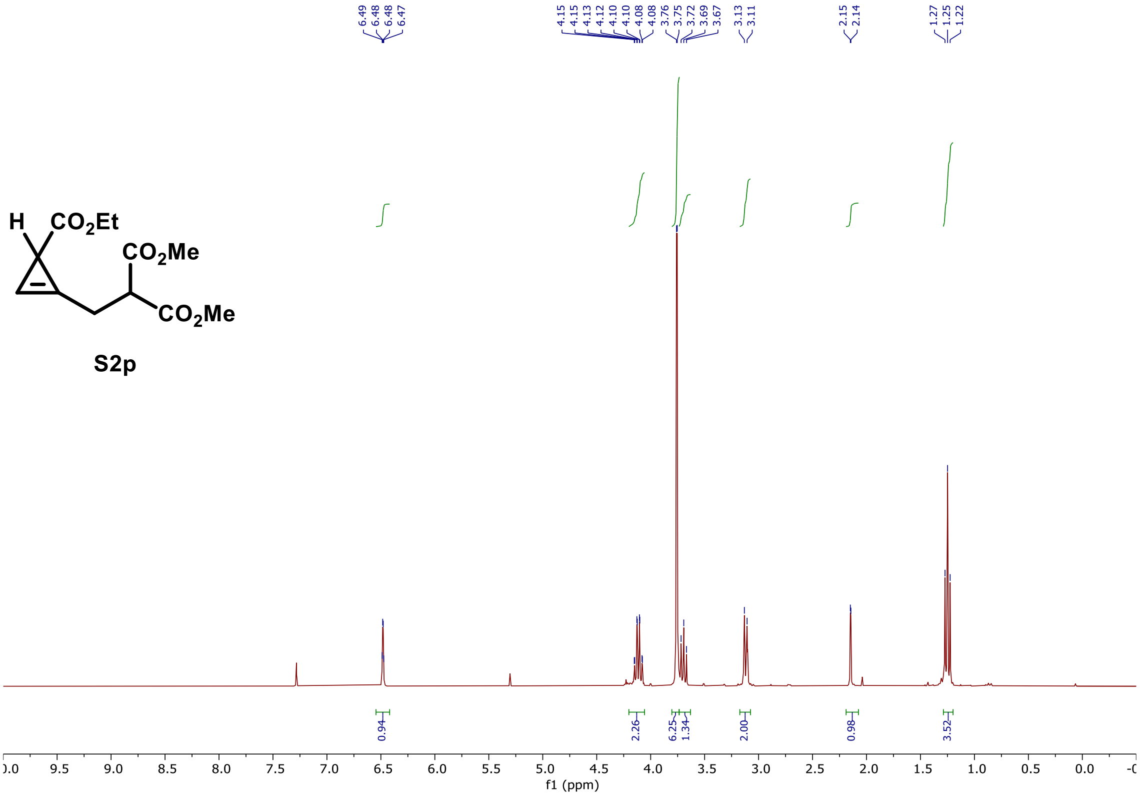

^{13}C-NMR of compound S2p (75 MHz, $CDCl_3$)

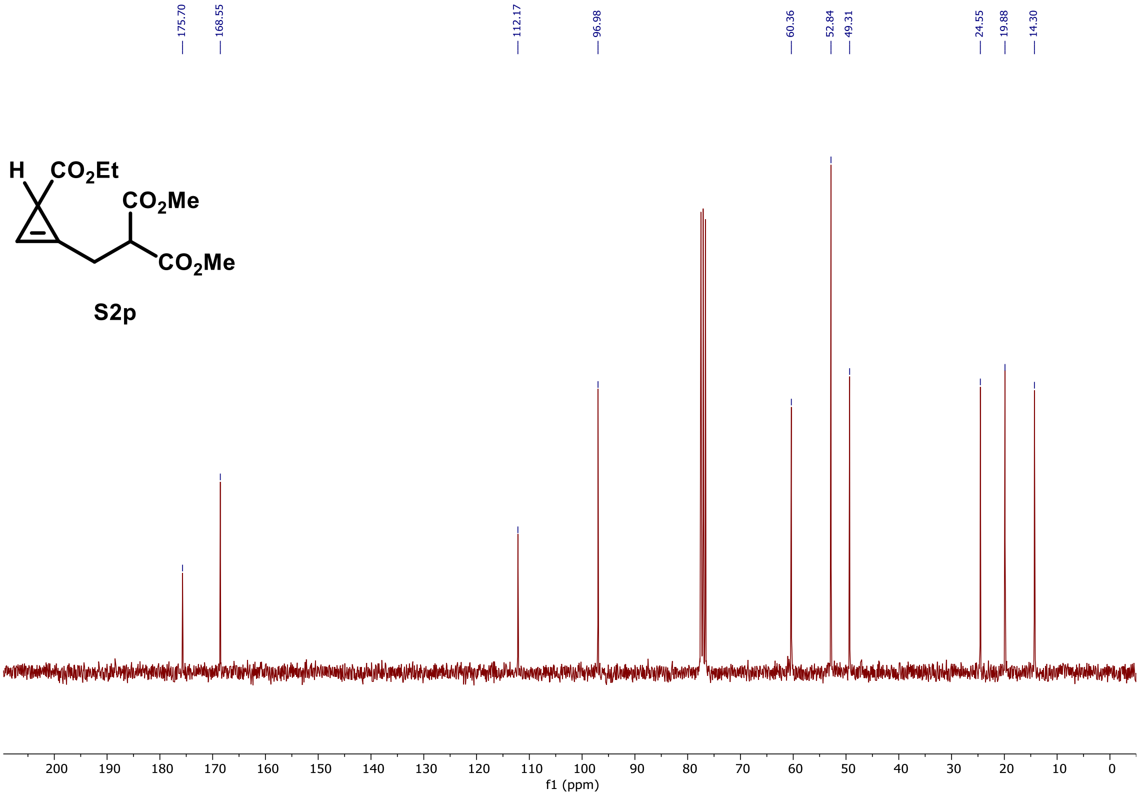

^{1}H-NMR of compound 1p (300 MHz, $CDCl_3$)

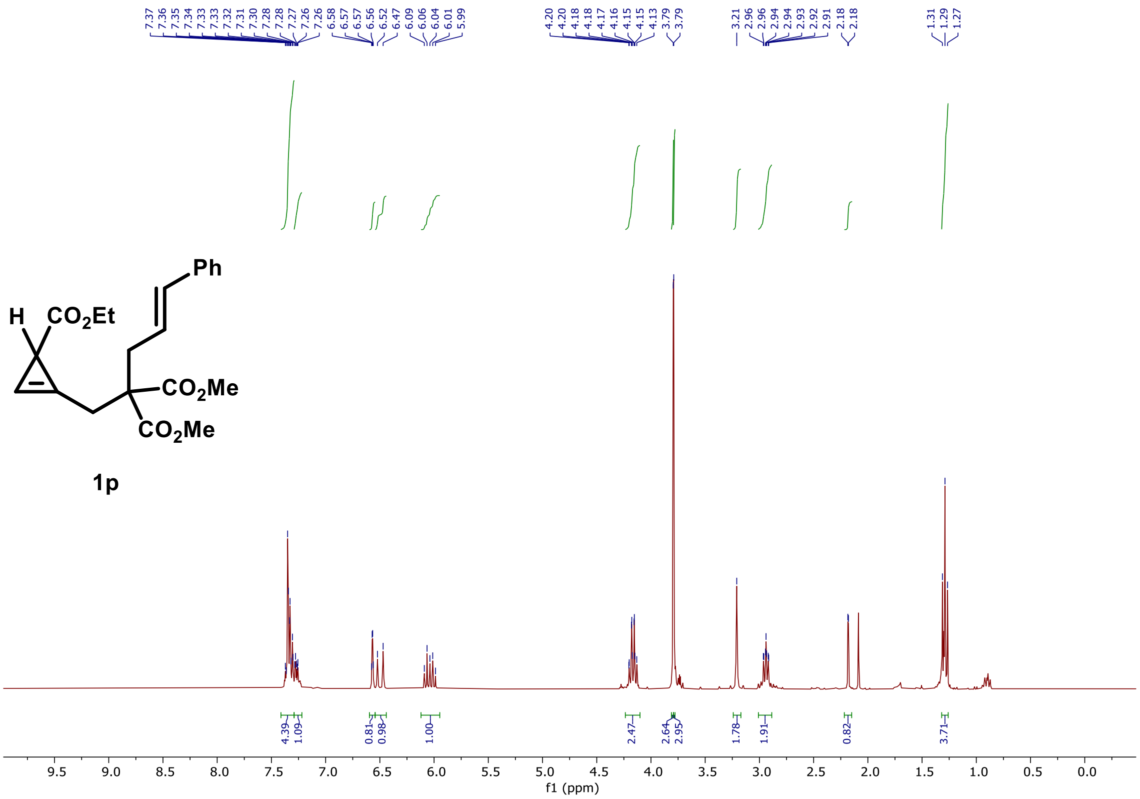

^{13}C-NMR of compound 2p (75 MHz, $CDCl_3$)

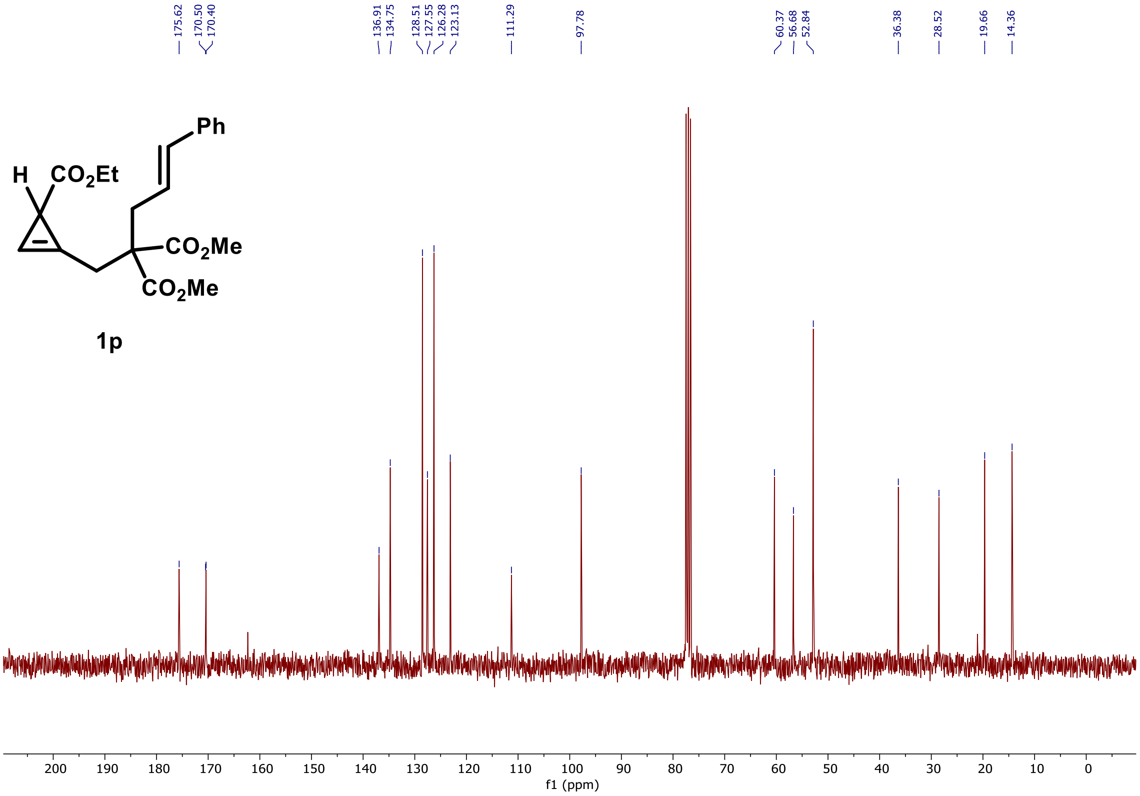

^{1}H-NMR of compound 1q (300 MHz, $CDCl_3$)

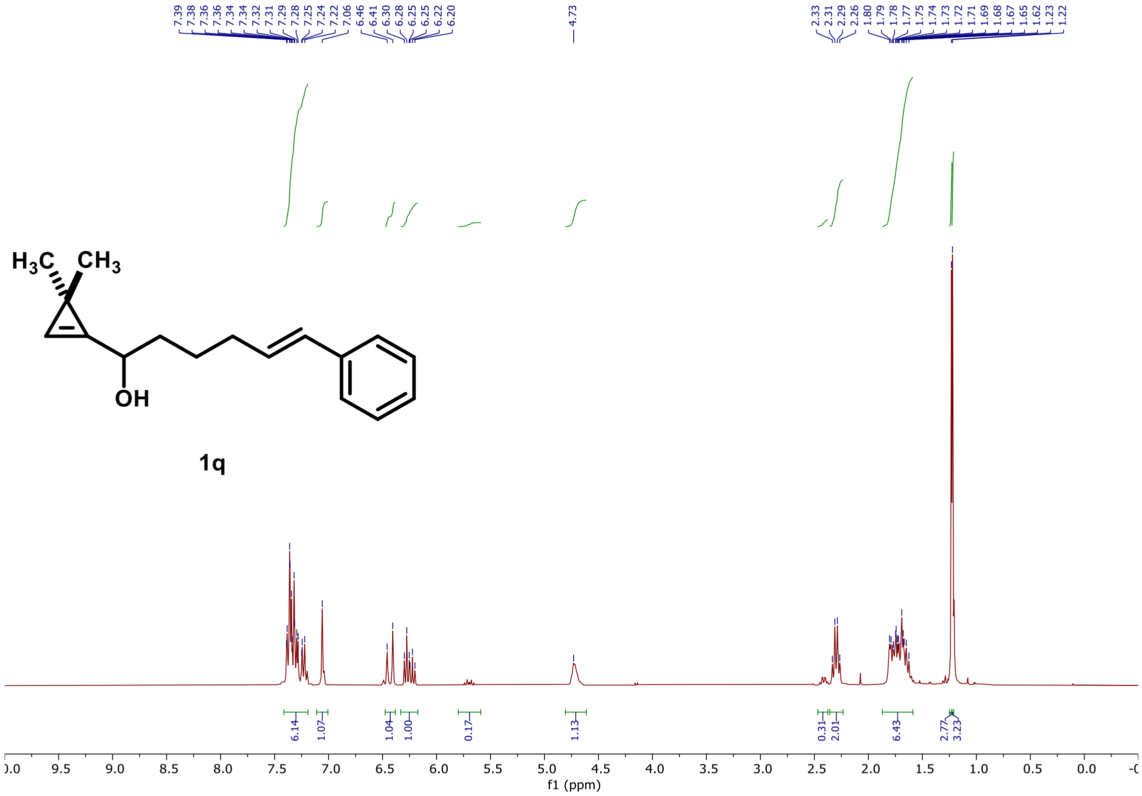

^{13}C-NMR of compound 1q (75 MHz, $CDCl_3$)

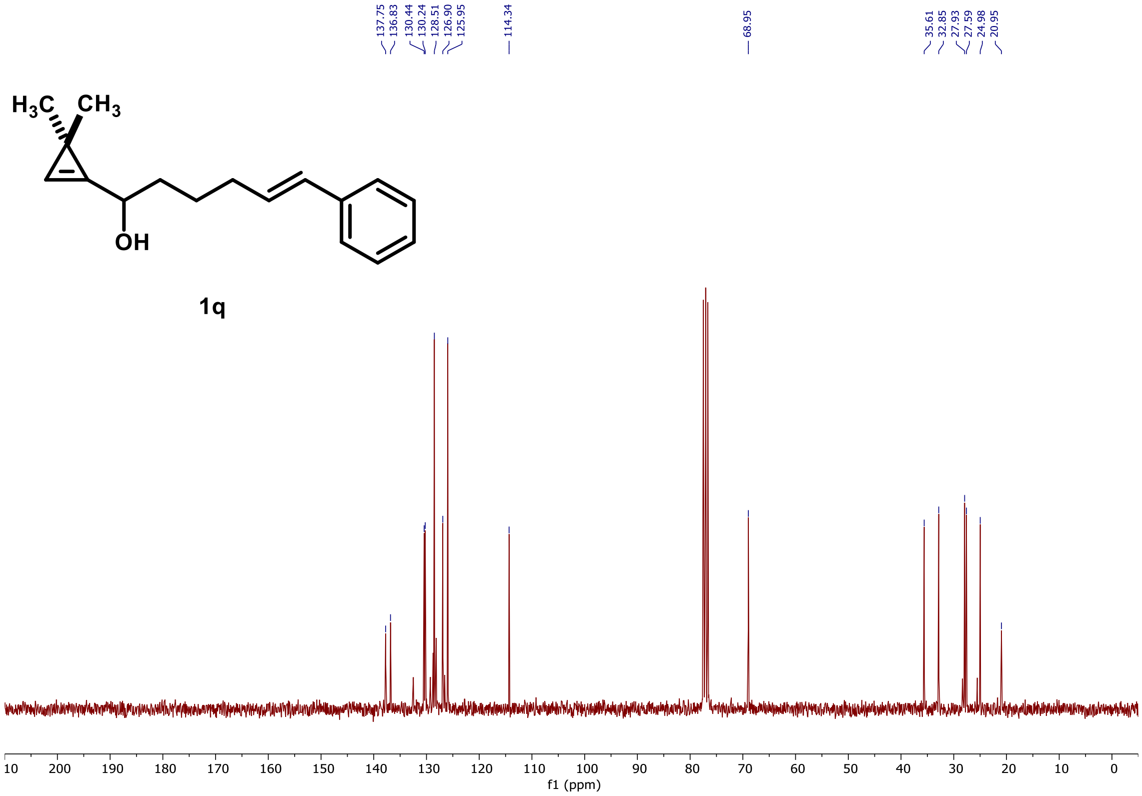

^{1}H-NMR of compound 1r (300 MHz, $CDCl_3$)

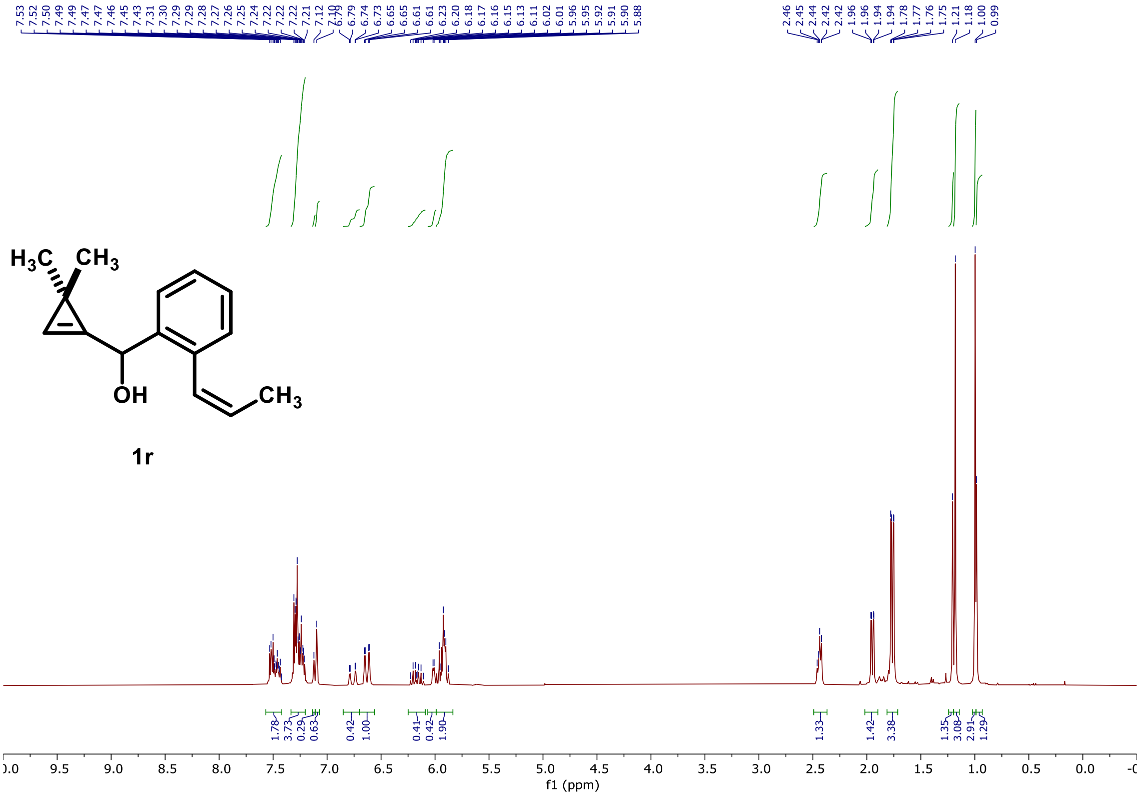

^{13}C-NMR (75 MHz, $CDCl_3$)

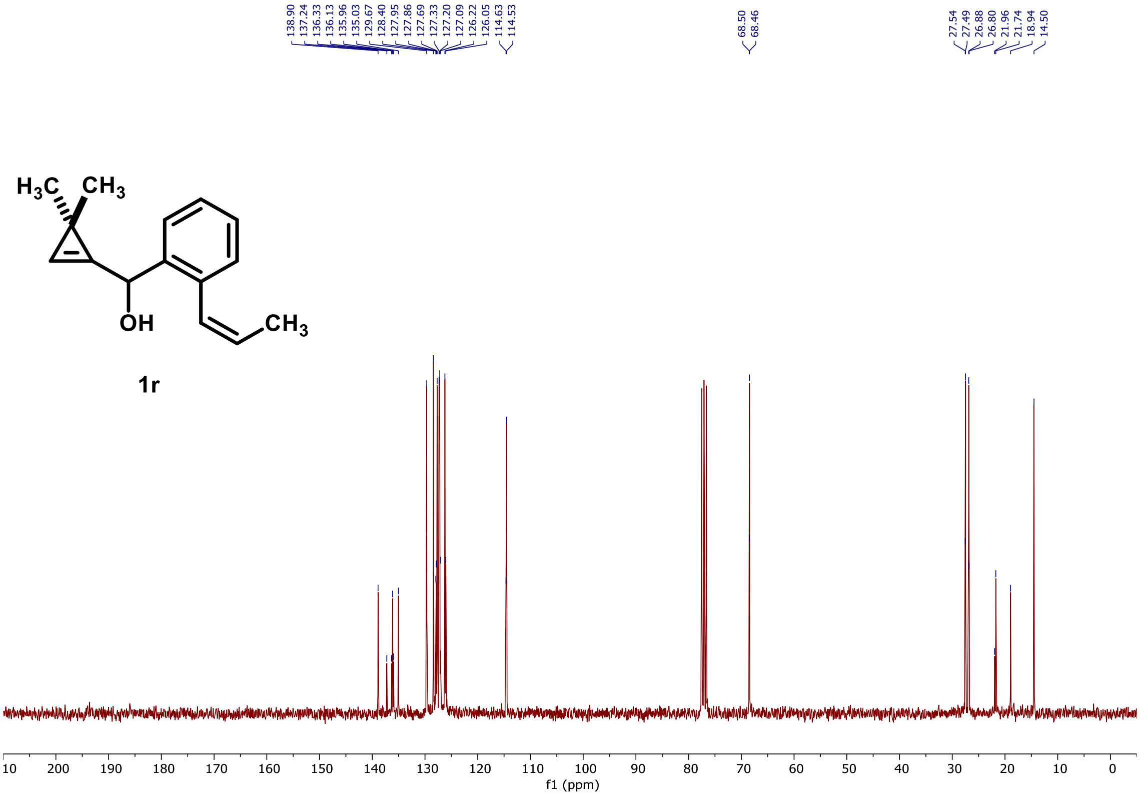

^{1}H-NMR of compound 1s (300 MHz, $CDCl_3$)

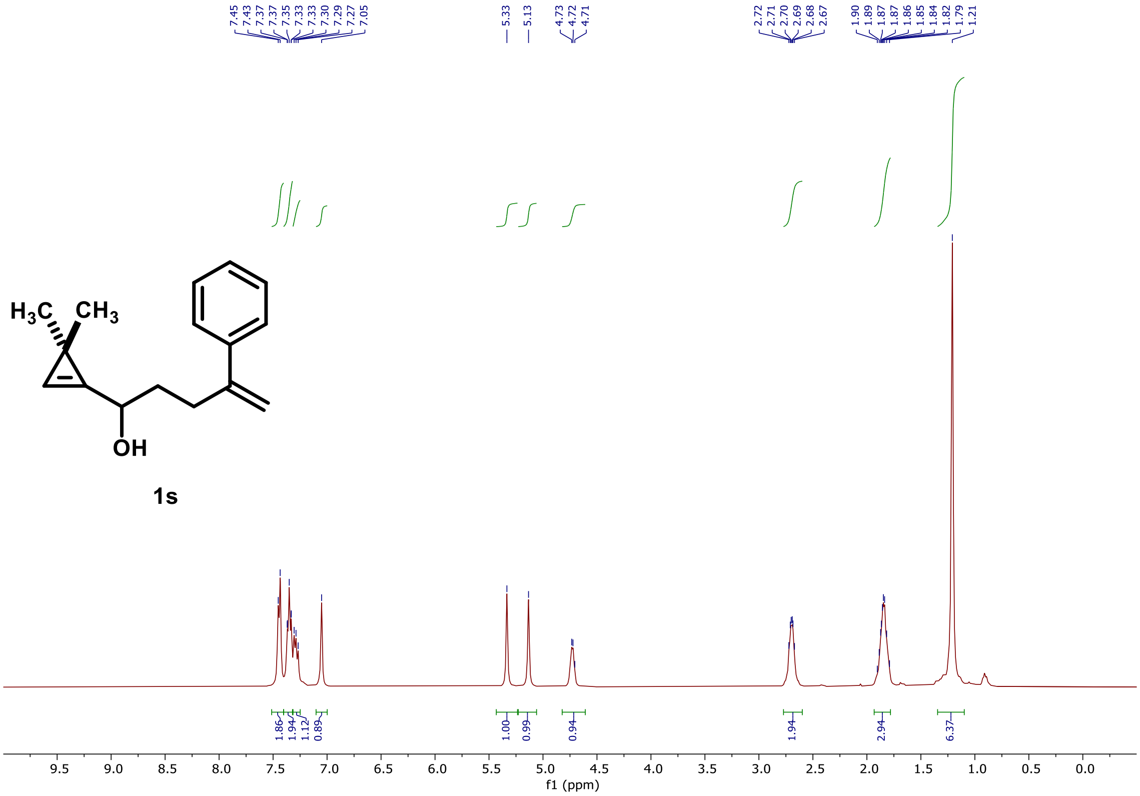

^{13}C-NMR of compound 1s (101 MHz, $CDCl_3$)

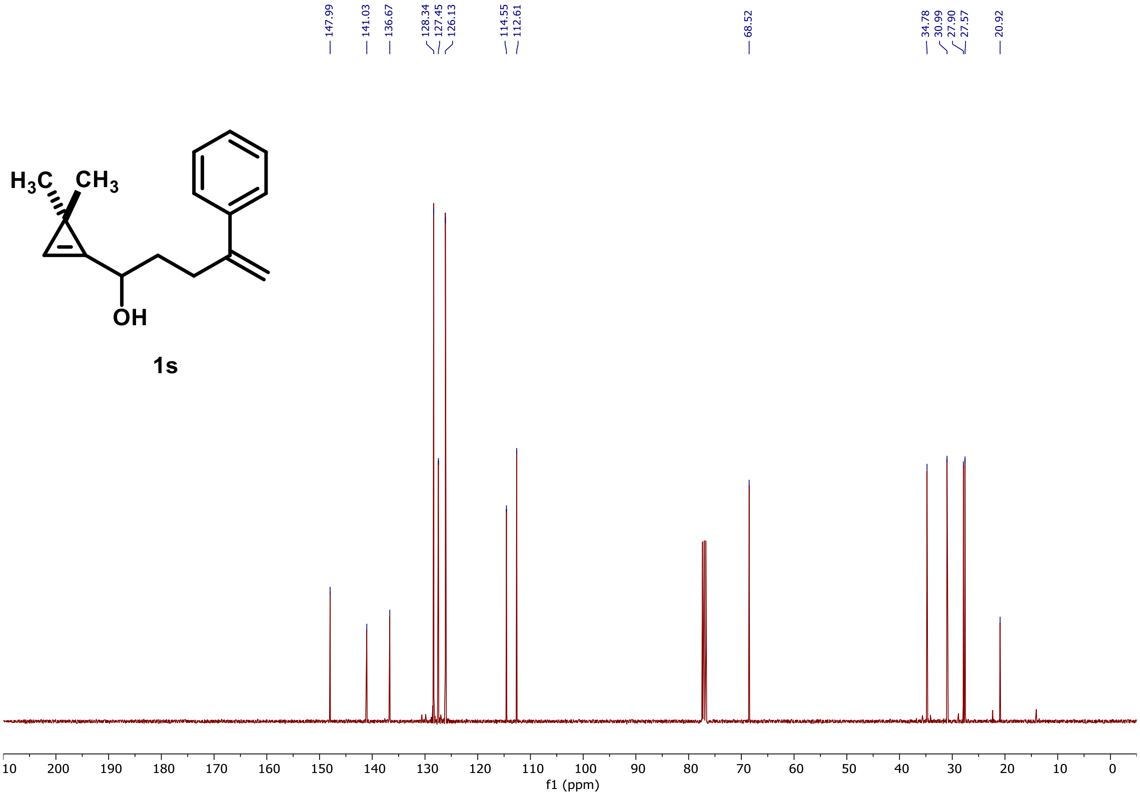

^{1}H-NMR of compound 2a (300 MHz, $CDCl_3$)

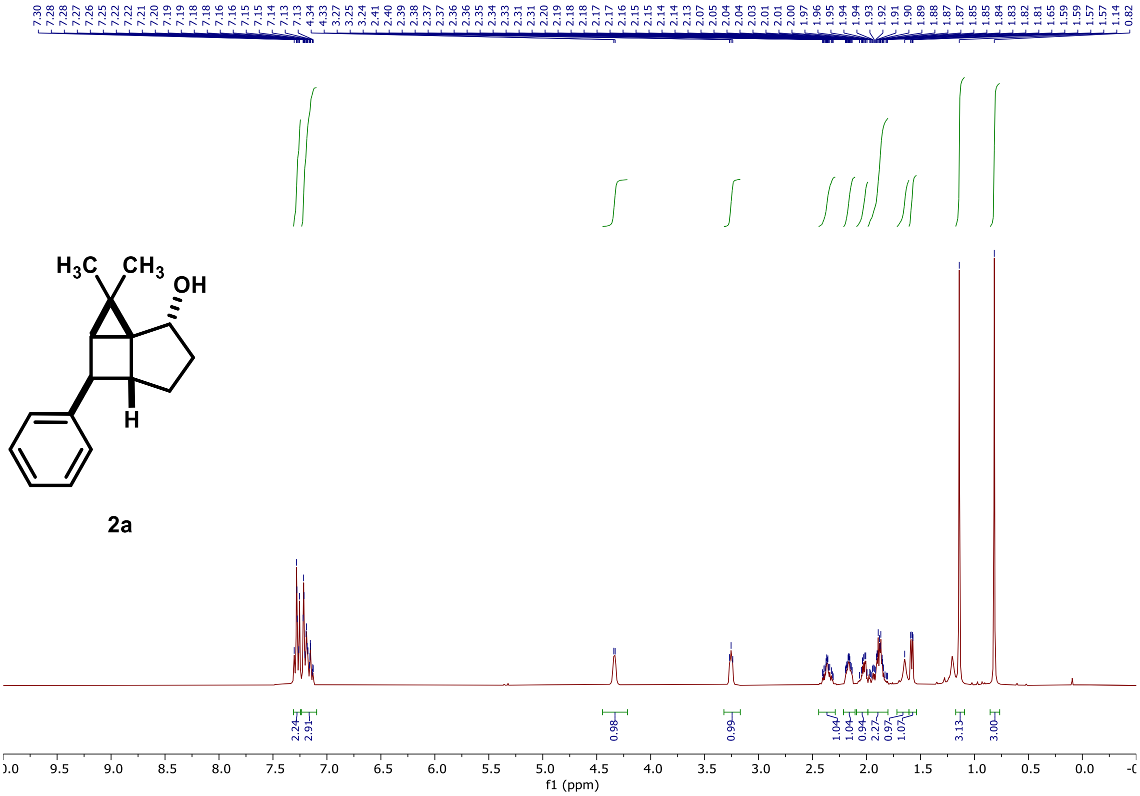

^{13}C-NMR of compound 2a (75 MHz, $CDCl_3$)

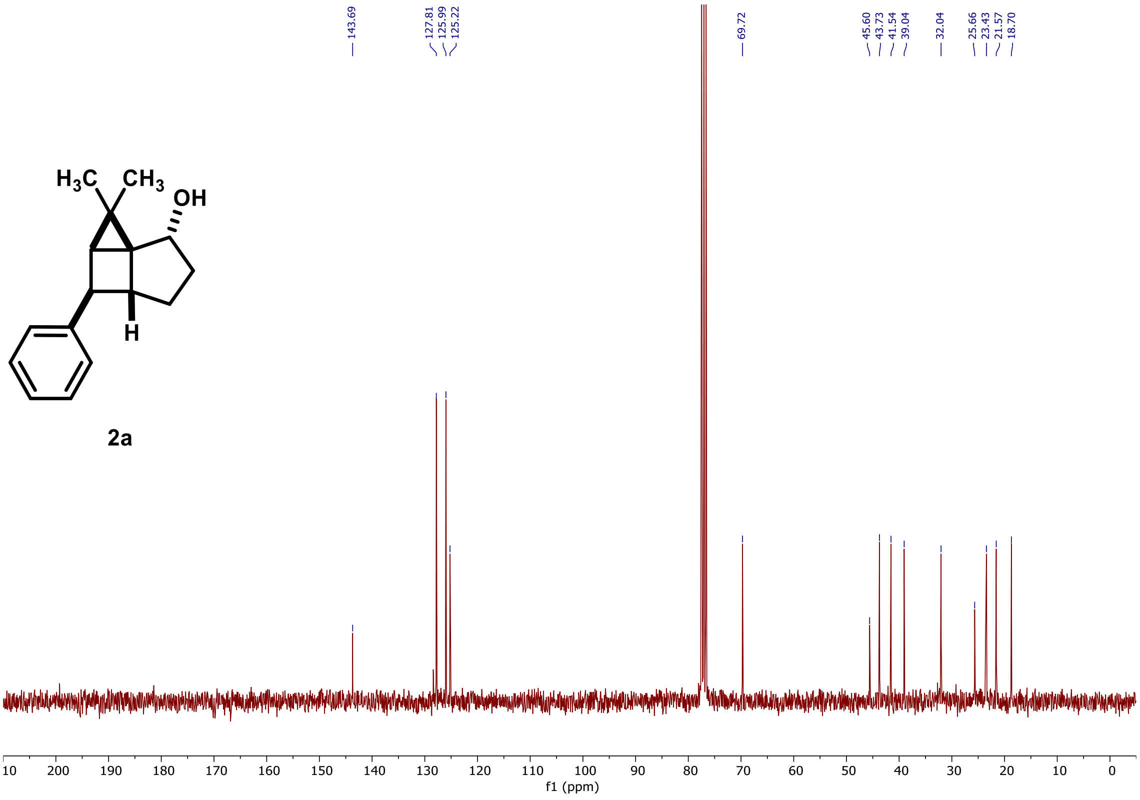

Atom label	H (ppm, Hz)	C (ppm)
1		45.6
2		25.7
3	1.58 (dd, *J* = 5.1, 1.4 Hz, 1H)	23.4
4	3.25 (t, *J* = 4.5 Hz, 1H)	43.7
5	2.10 – 2.23 (m, 1H)	41.5
6	1.79 – 1.97 (m, 1H), 2.30 – 2.42 (m, 1H)	32.1
7	1.79 – 1.97 (m, 1H), 1.97 – 2.10 (m, 1H)	39.0
8	4.34 (d, *J* = 3.7 Hz, 1H)	69.7
9	1.14 (s, 3H)	21.6
10	0.82 (s, 3H)	18.7
11		143.7
12–14	7.12 – 7.23 (m, 3H), 7.24 – 7.33 (m, 2H)	125.2, 126.0, 127.8,
OH	1.65 (bs, 1H)	

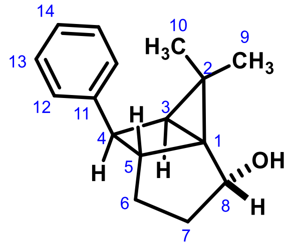

2a

^{1}H-^{13}C HSQC of compound 2a ($CDCl_3$)

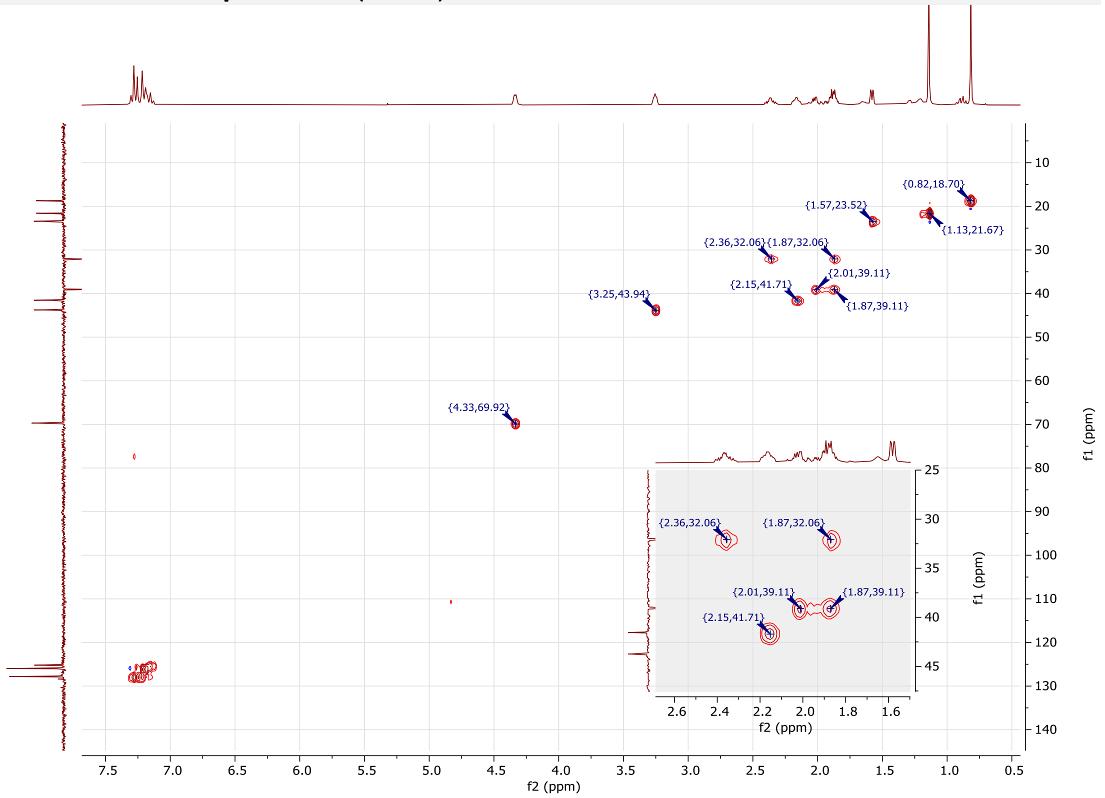

^{1}H-^{1}H COSY of compound 2a (CDCl$_3$)

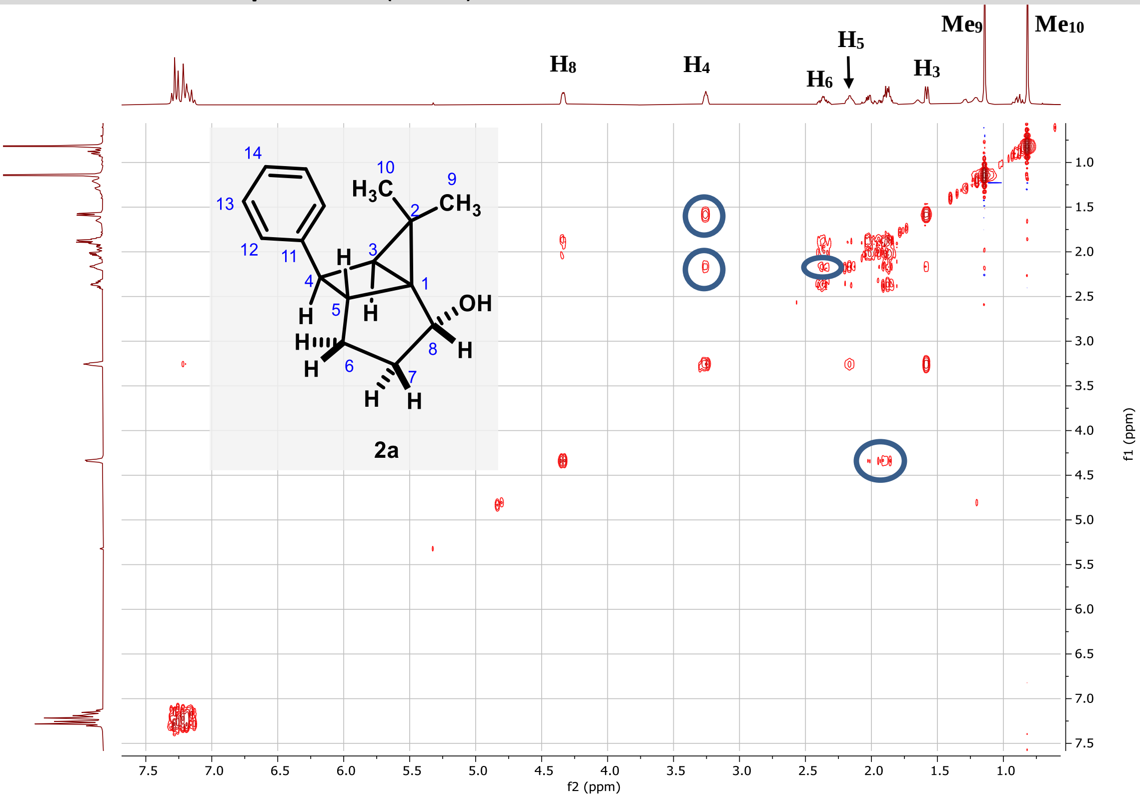

Relevant signals (COSY): $^3J_{H,H}$ **(H$_8$-H$_7$)**, $^3J_{H,H}$ **(H$_4$-H$_5$)**, $^3J_{H,H}$ **(H$_4$-H$_3$)**, $^3J_{H,H}$ **(H$_5$-H$_6$)**.

^{1}H-^{13}C HMBC of compound 2a (CDCl$_3$):

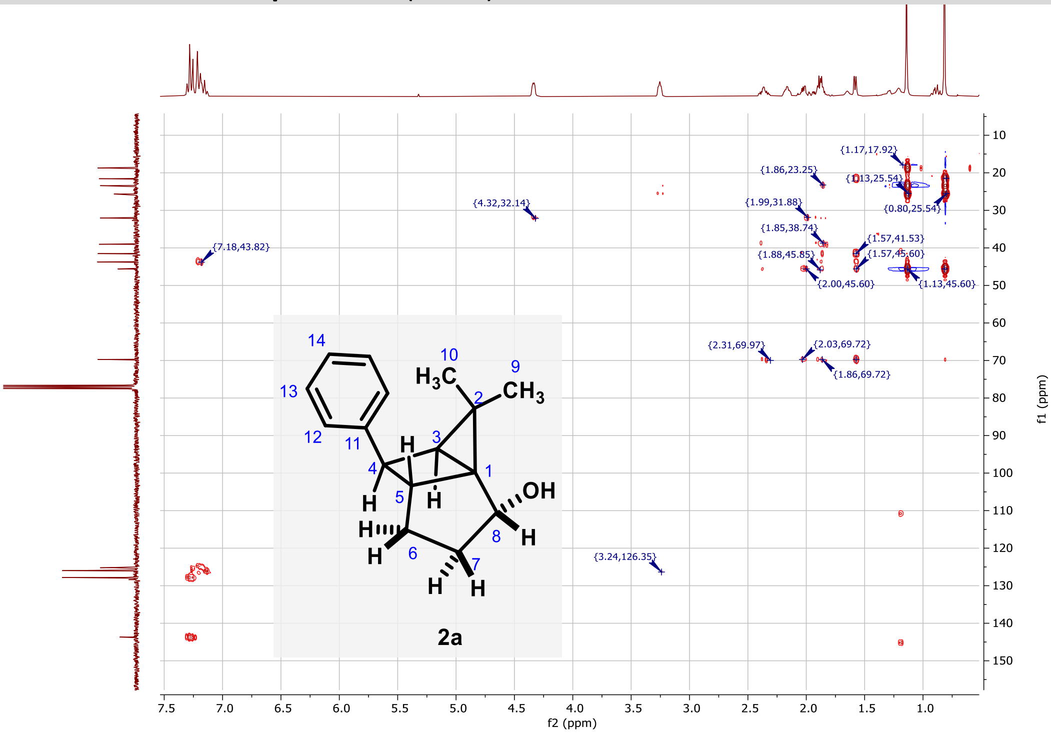

Relevant signals (HMBC): $^3J_{H,C}$ **(H$_{12}$-C$_4$)**, $^3J_{H,C}$ **(H$_8$-C$_6$)**, $^3J_{H,C}$ **(H$_6$-C$_1$)**.

^{1}H-^{1}H NOESY of compound 2a ($CDCl_3$)

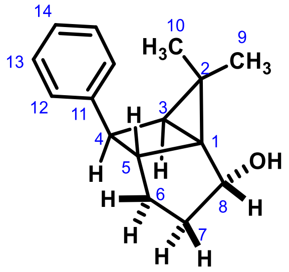

2a

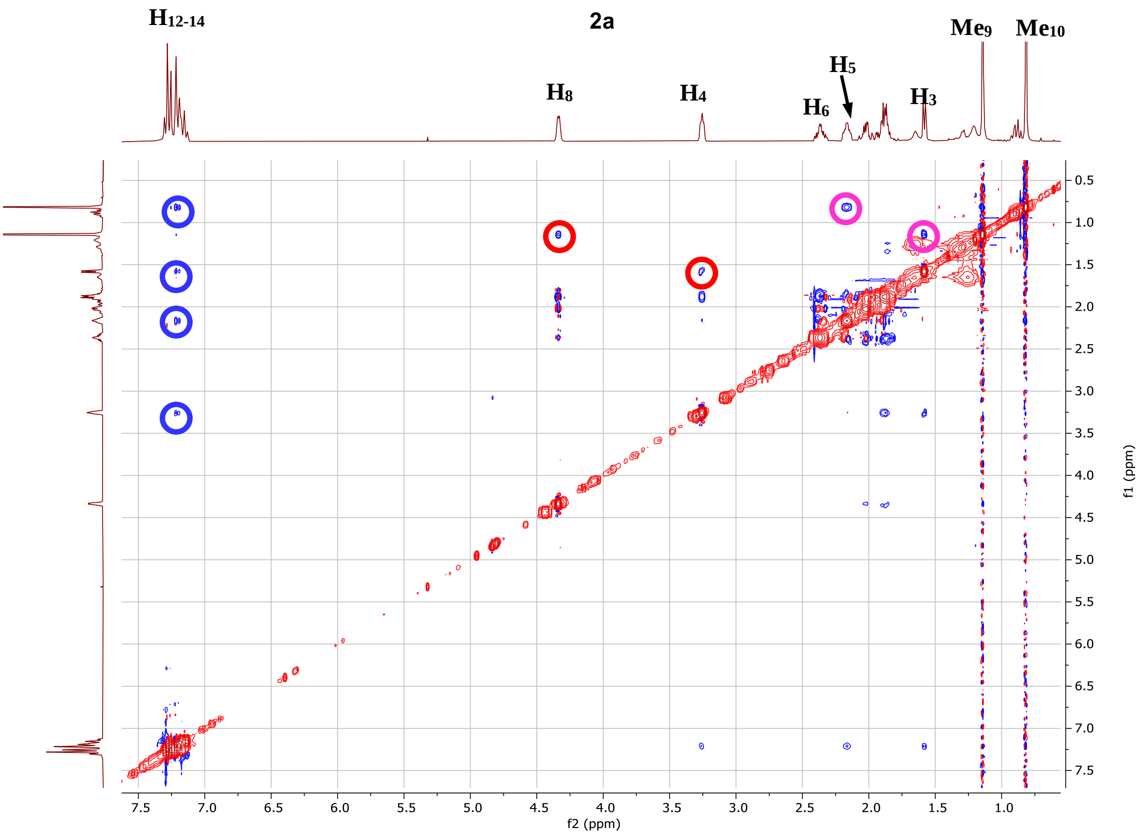

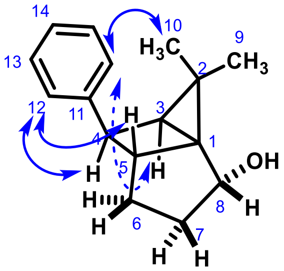

2a

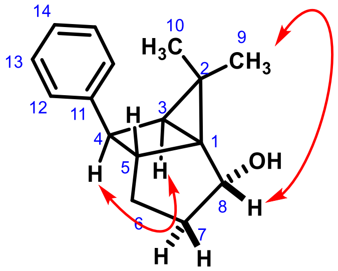

2a

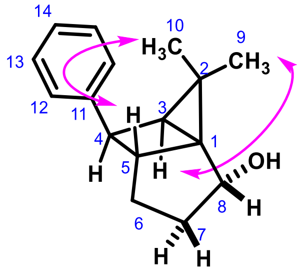

2a

^{1}H-NMR of compound 2a' (600 MHz, $CDCl_3$)

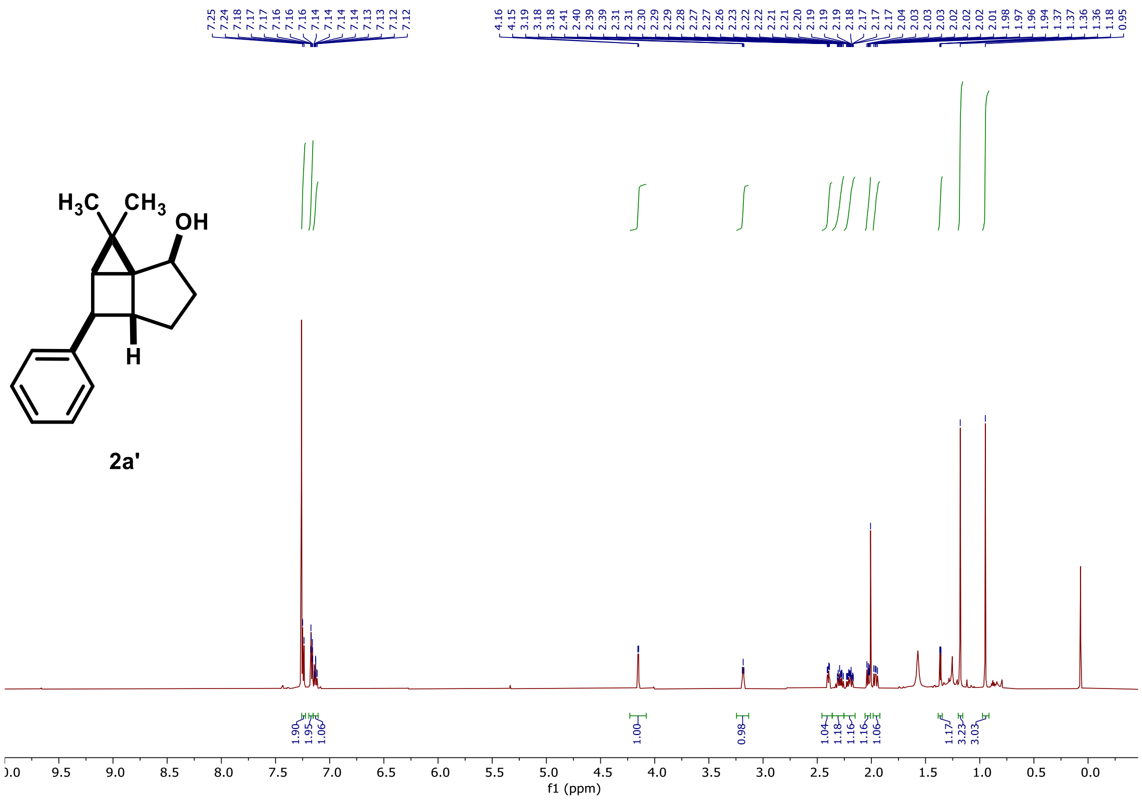

^{13}C-NMR of compound 2a' (101 MHz, $CDCl_3$)

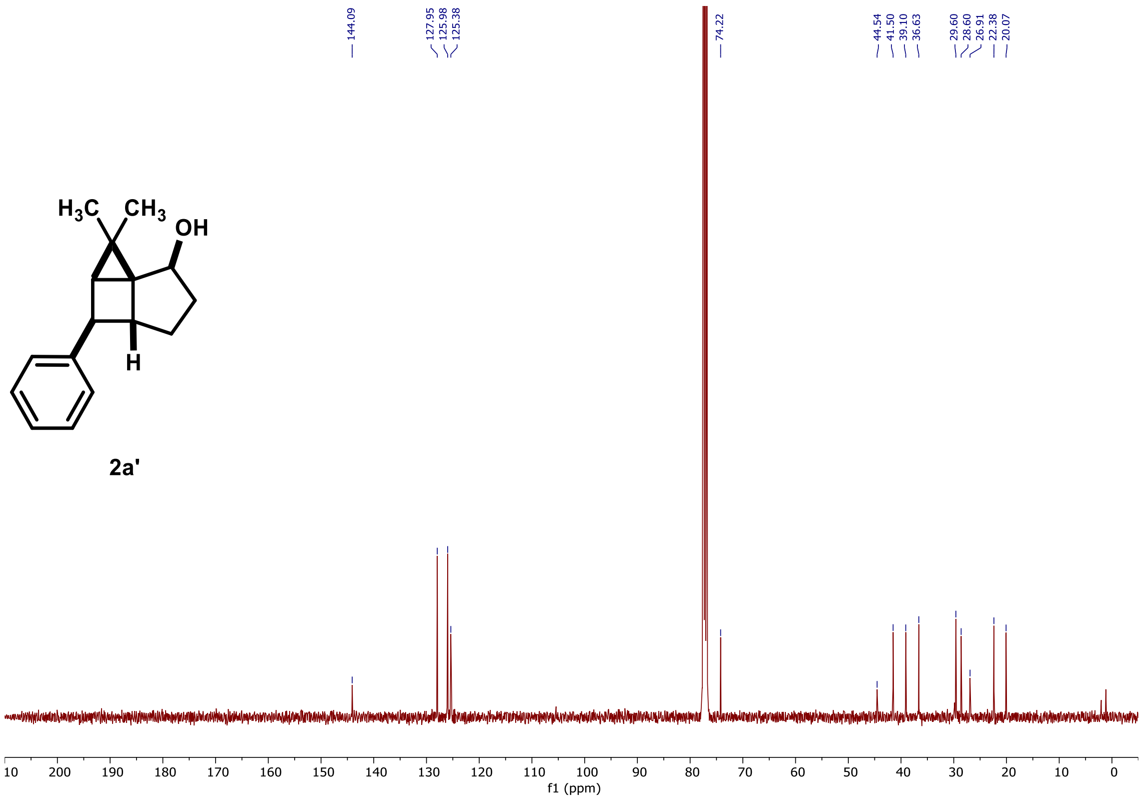

^{1}H-^{13}C HSQC of compound **2a’** ($CDCl_3$)

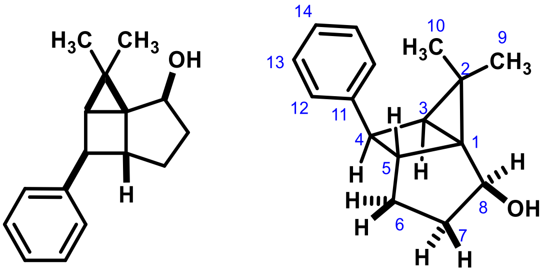

2a' **2a'**

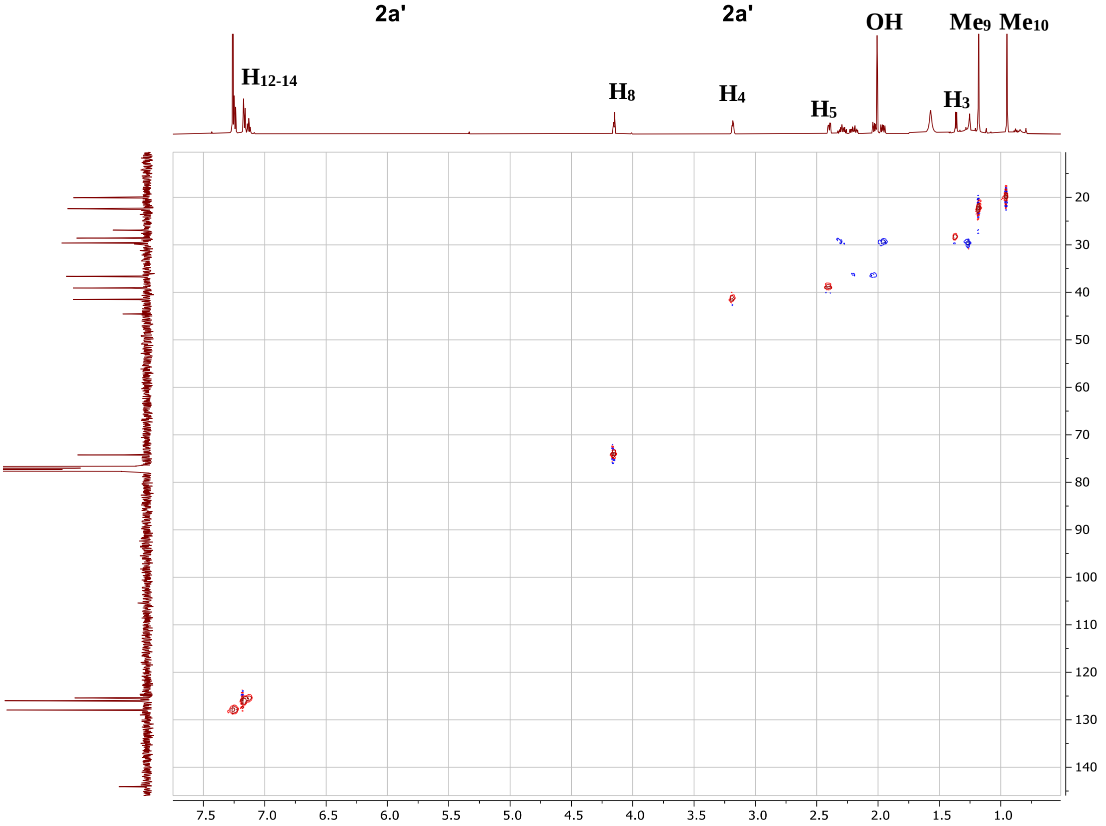

Selective nOe experiments of compound 2a’ (CDCl$_3$)

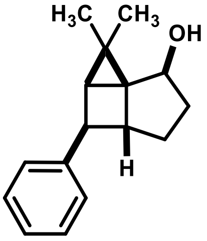

2a'

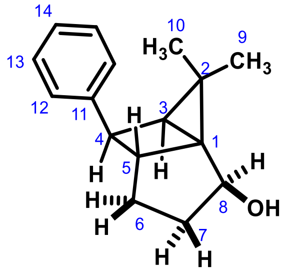

2a'

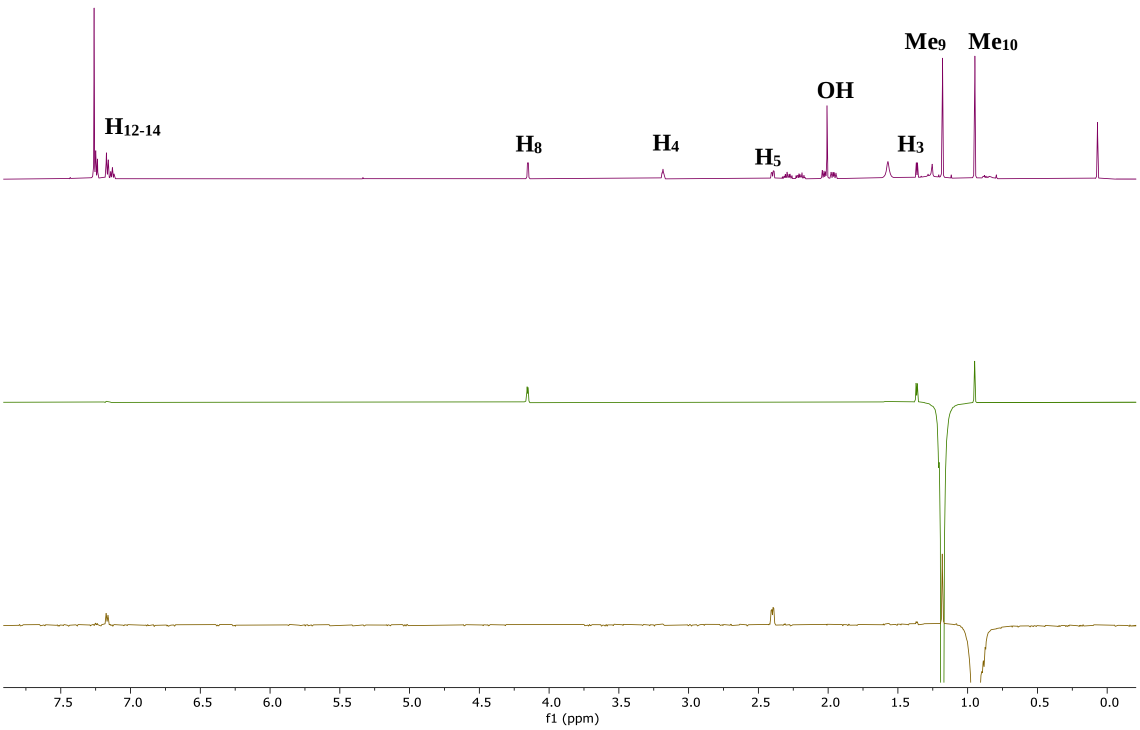

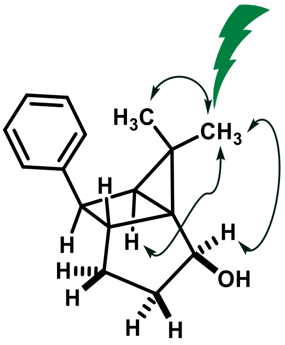

2a'

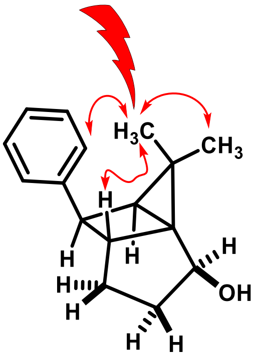

2a'

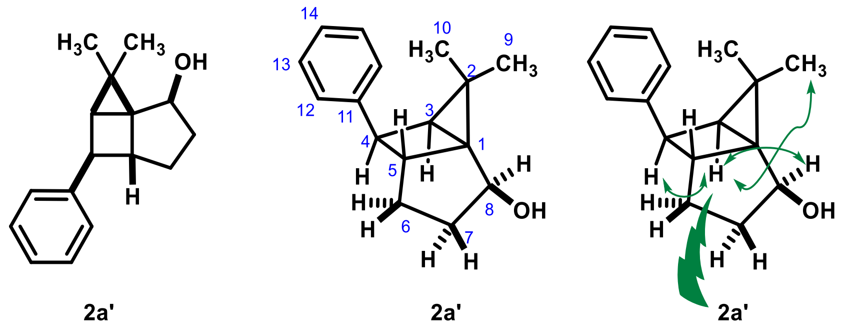
H3C
CH3
OH
H
2a'
14
10
9
13
H3C
2
CH3
12
11
H
3
4
1
5
H
H
8
H
H
OH
6
7
H
H
H
2a'
H3C
CH3
H
H
H
H
OH
H
H
H
H
2a'

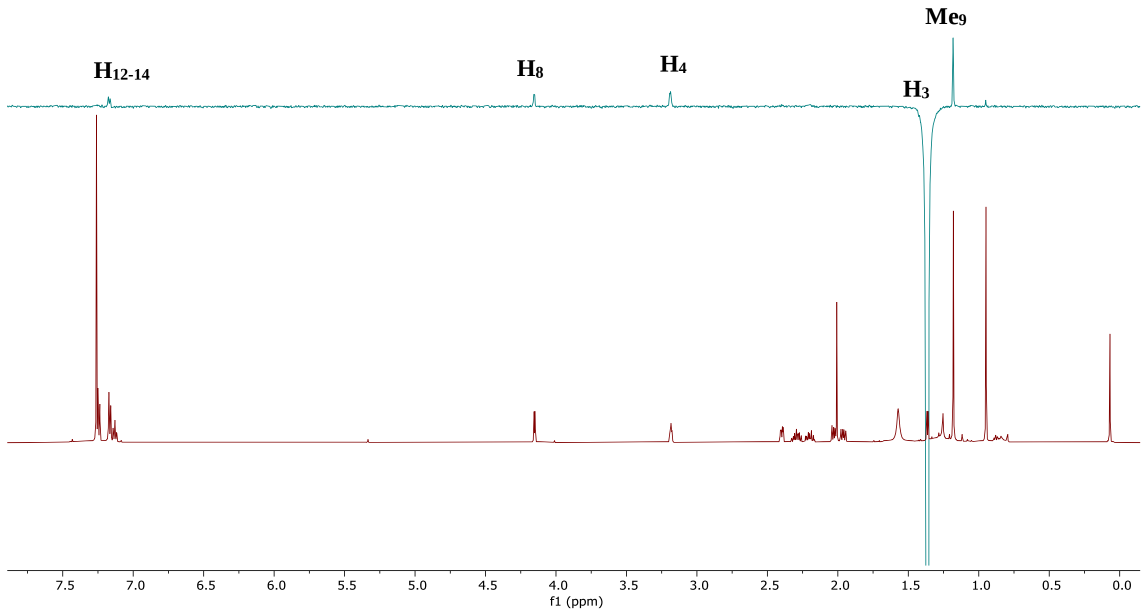
Me9
H12-14
H8
H4
H3
7.5
7.0
6.5
6.0
5.5
5.0
4.5
4.0
3.5
3.0
2.5
2.0
1.5
1.0
0.5
0.0
f1 (ppm)

^{1}H-NMR of compound 2b (300 MHz, $CDCl_3$)

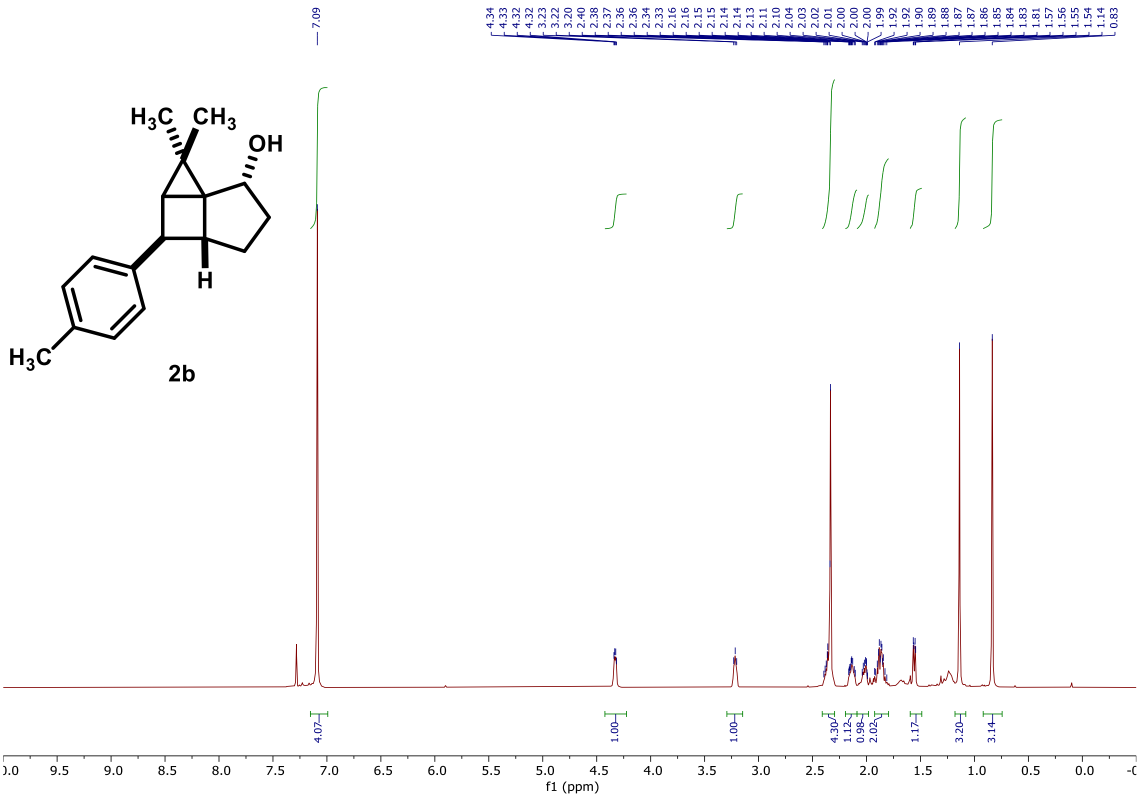

^{13}C-NMR of compound 2b (75 MHz, $CDCl_3$)

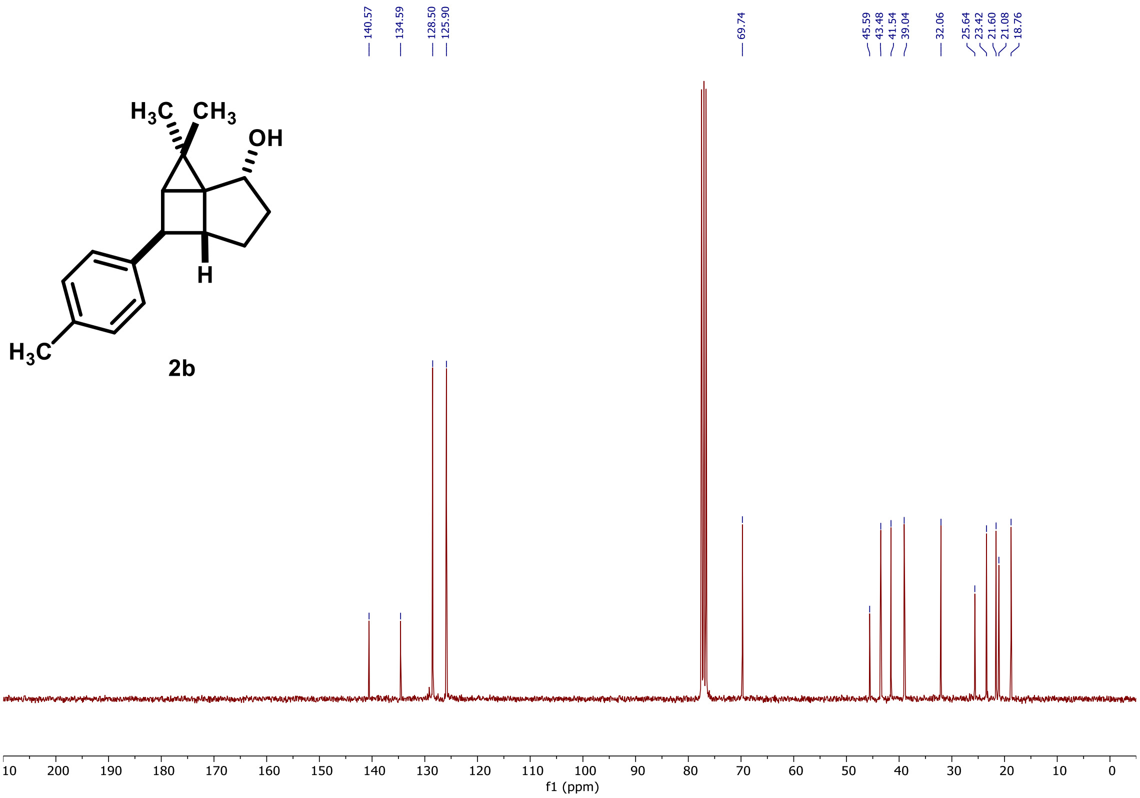

^{1}H-NMR of compound 2b’ (400 MHz, $CDCl_3$)

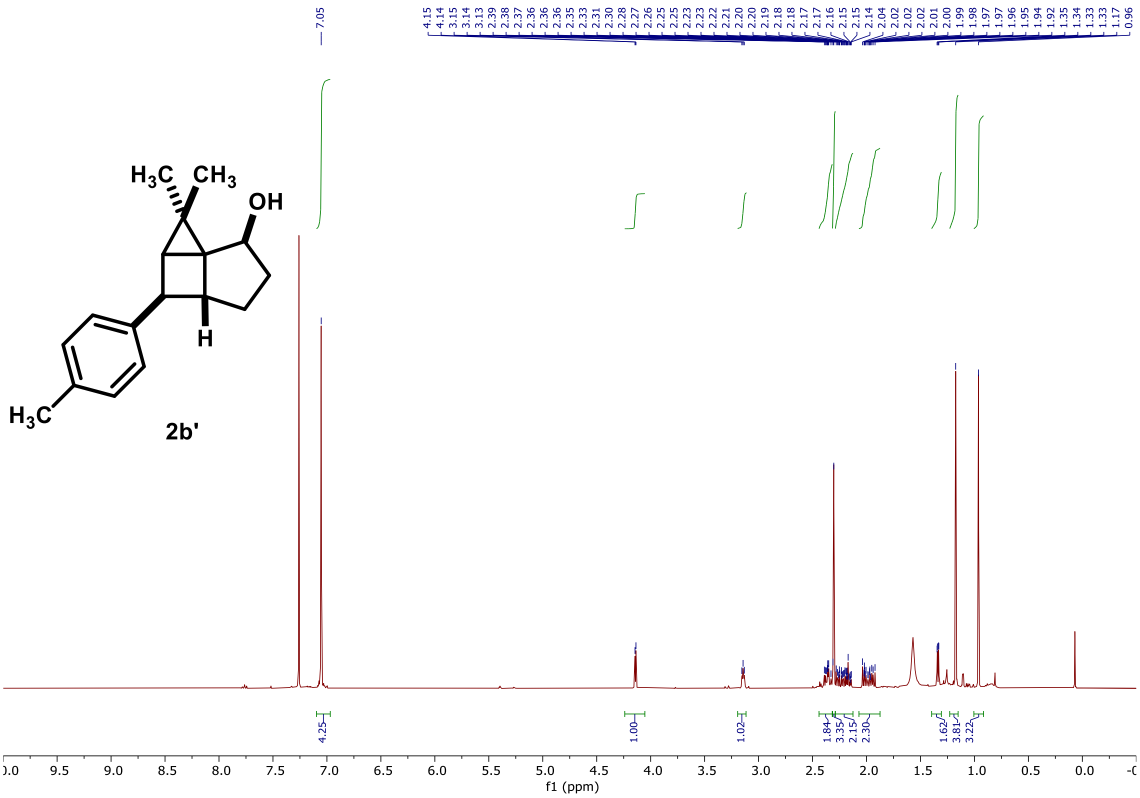

^{13}C-NMR of compound 2b’ (101 MHz, $CDCl_3$)

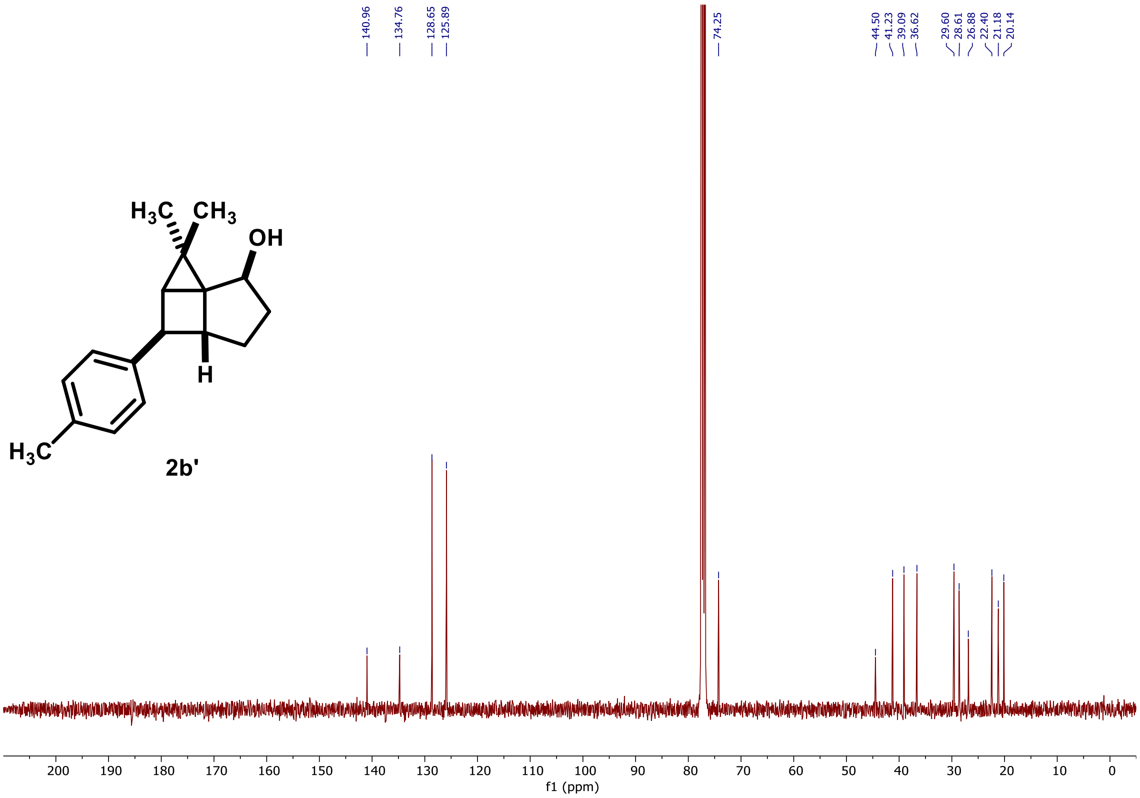

^{1}H-NMR of compound 2c (300 MHz, $CDCl_3$)

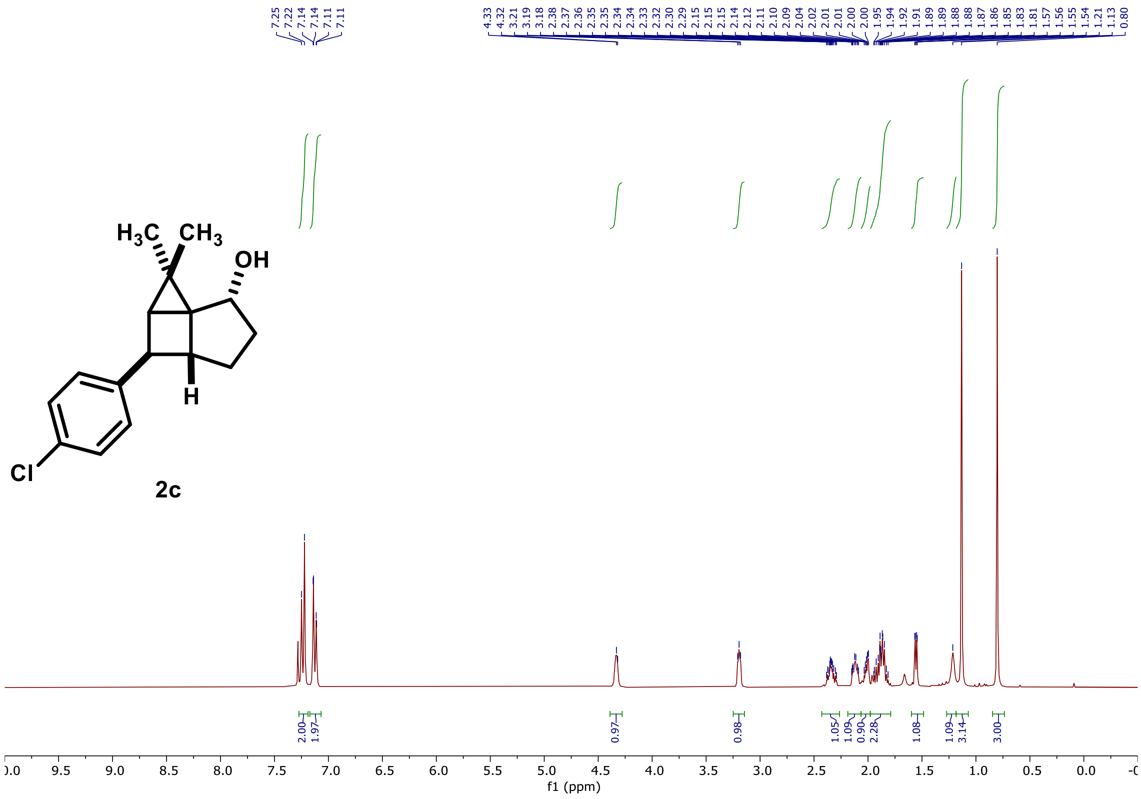

^{13}C-NMR of compound 2c (75 MHz, $CDCl_3$)

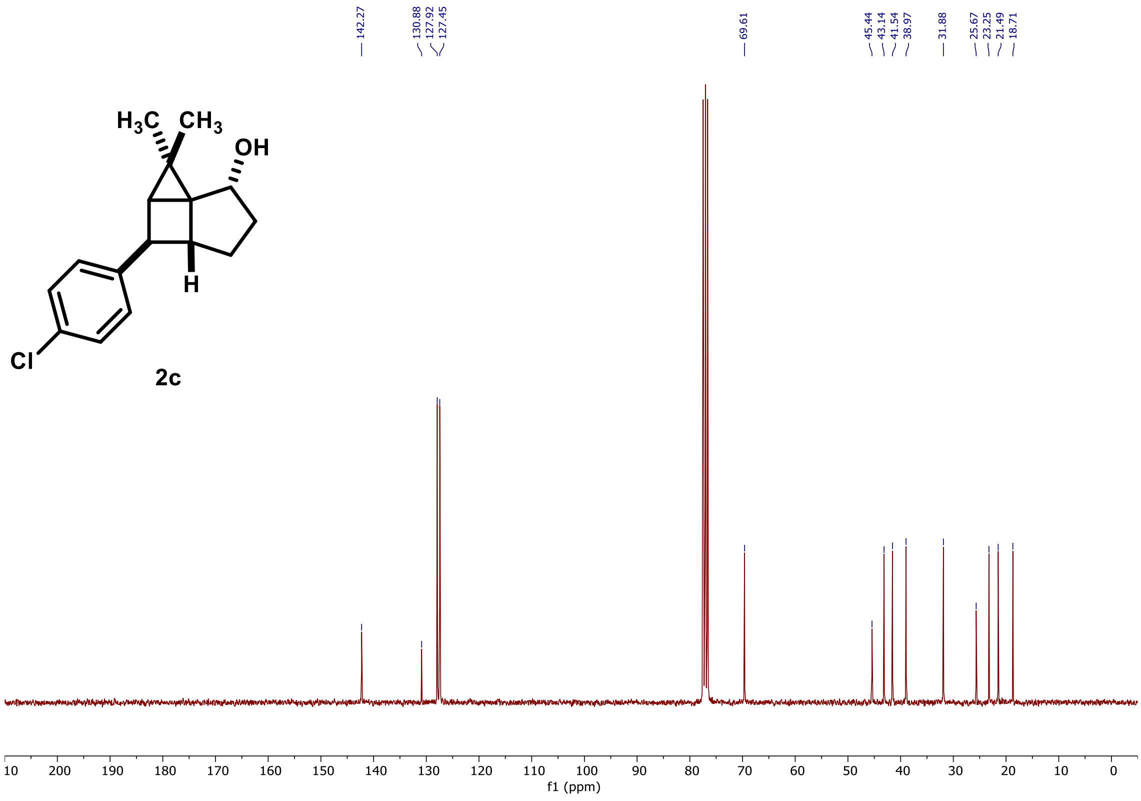

^{1}H-NMRof compound 2c’ (400 MHz, $CDCl_3$)

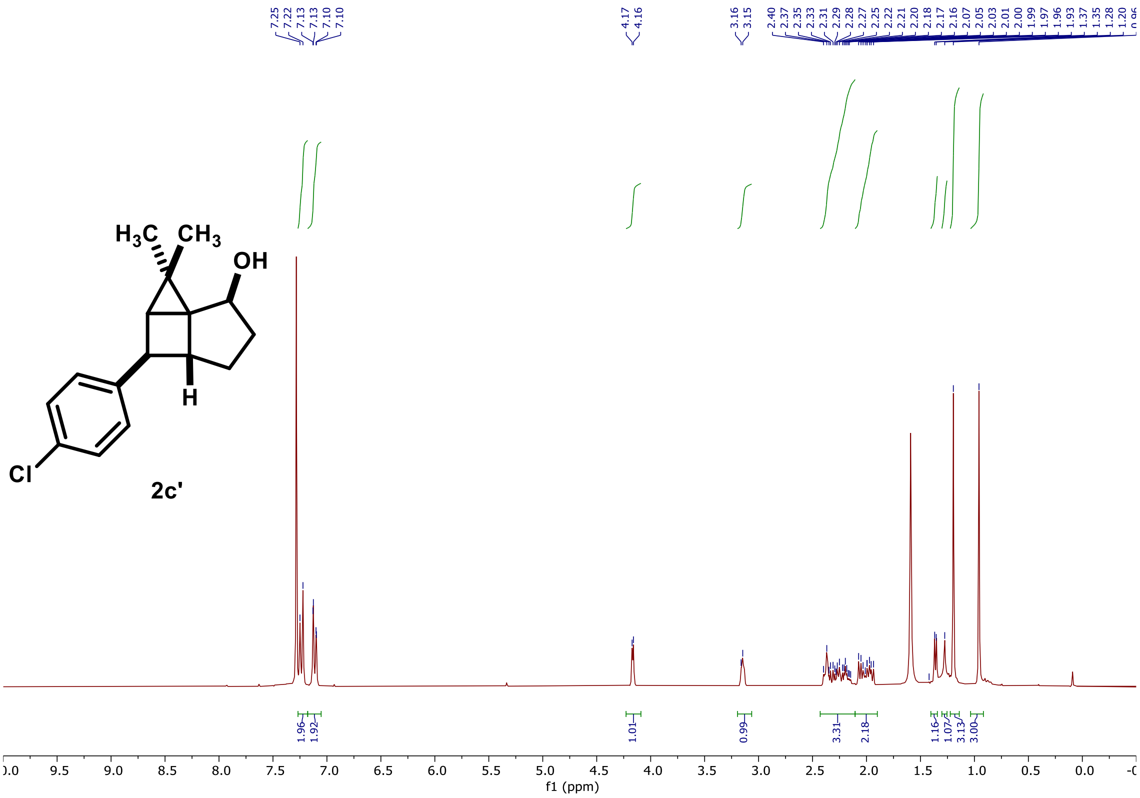

^{13}C-NMR of compound 2c’ (75 MHz, $CDCl_3$)

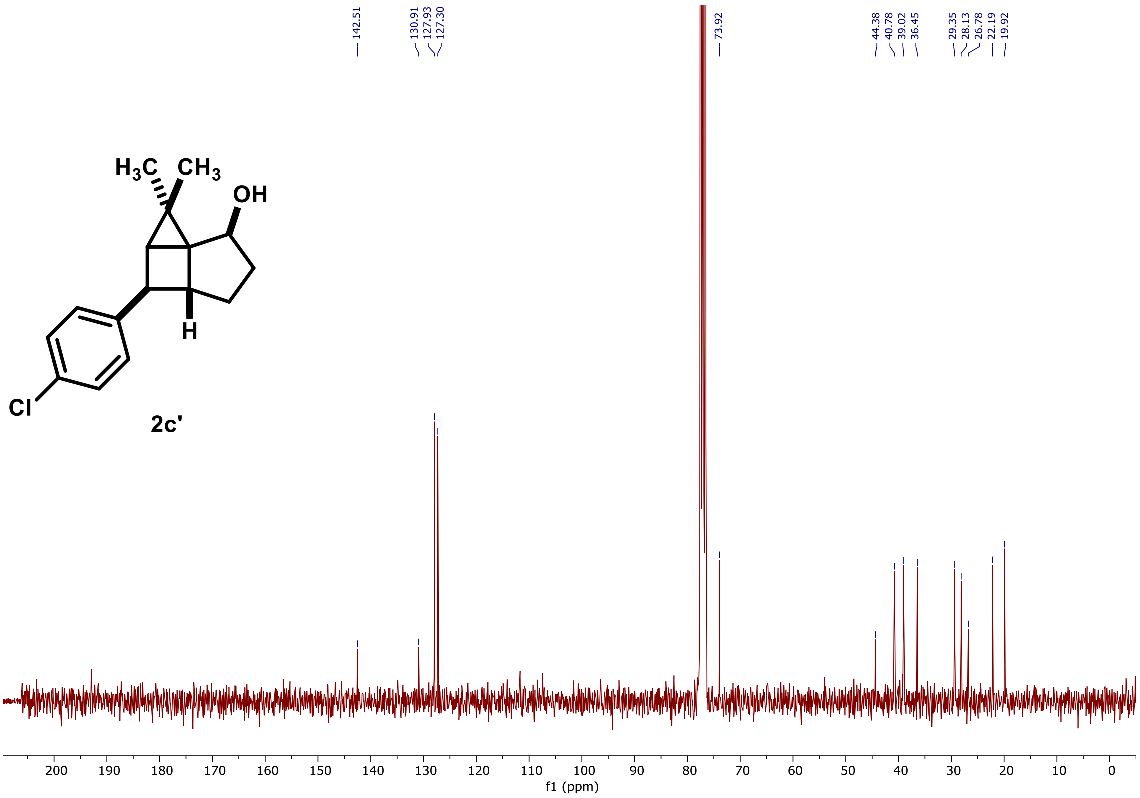

^{1}H-NMR of compound 2d (300 MHz, $CDCl_3$)

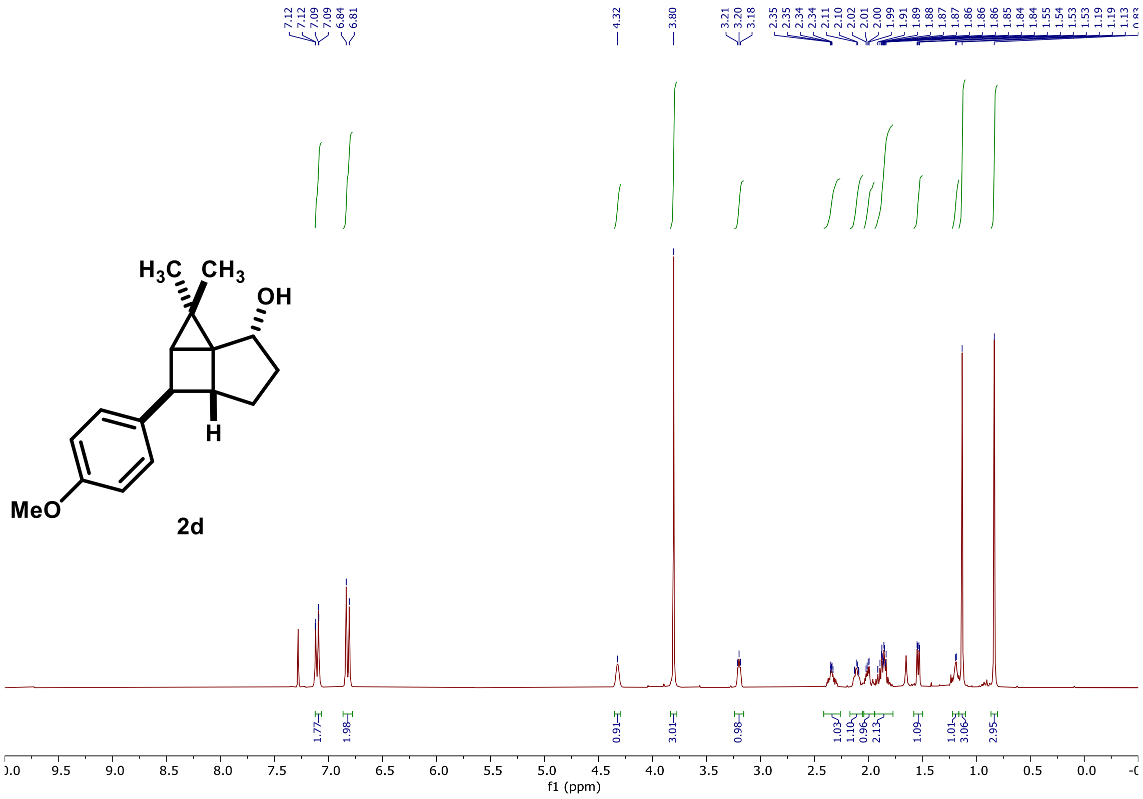

^{13}C-NMR of compound 2d (75 MHz, $CDCl_3$)

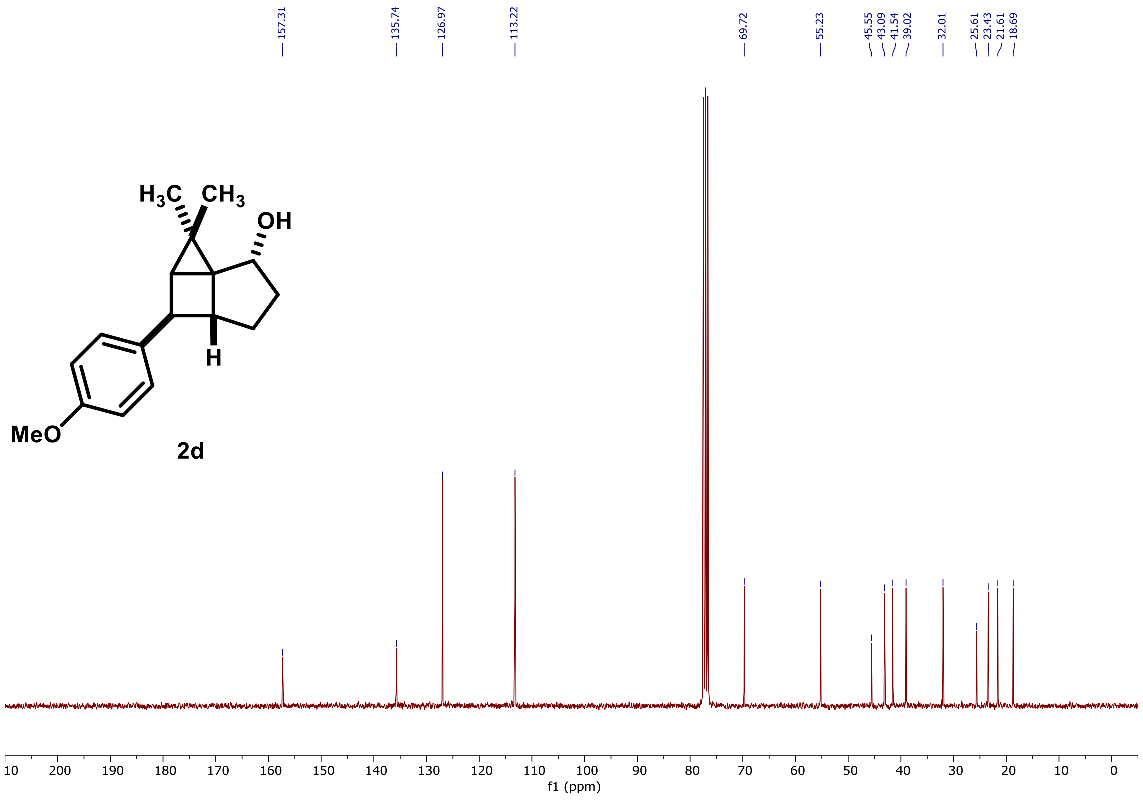

^{1}H-NMR of compound 2d' (400 MHz, $CDCl_3$)

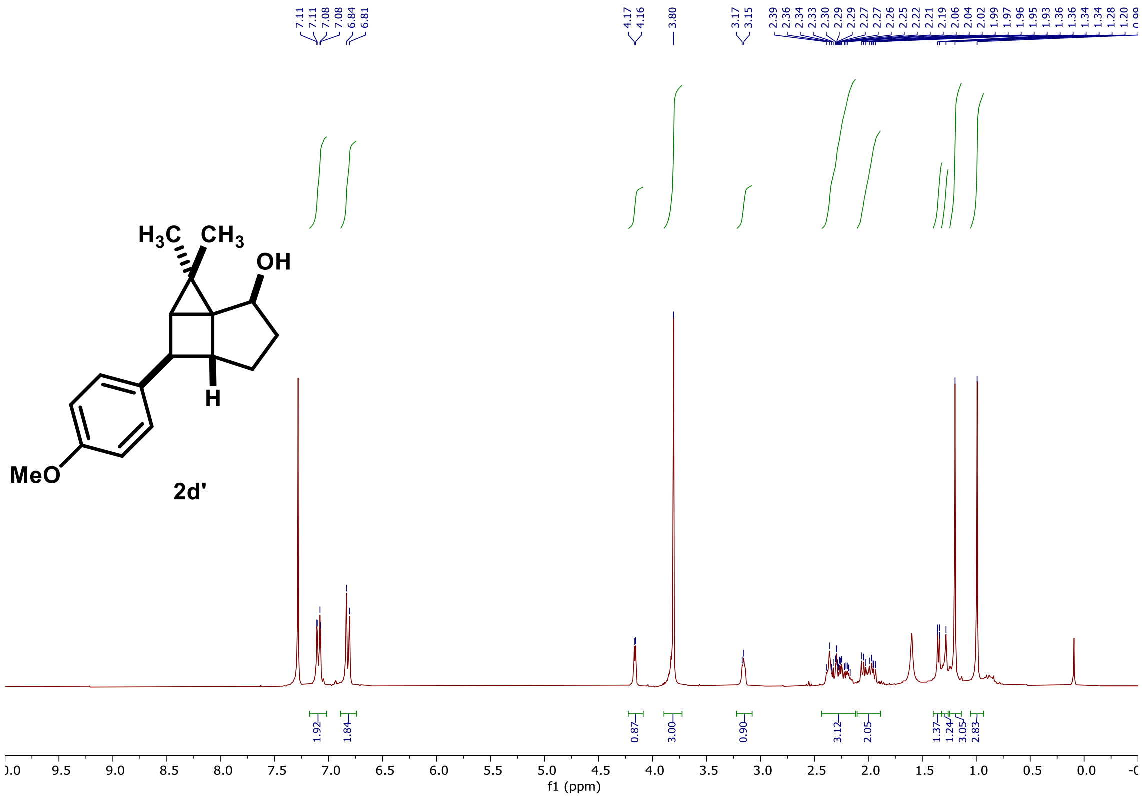

^{13}C-NMR of compound 2d' (101 MHz, $CDCl_3$)

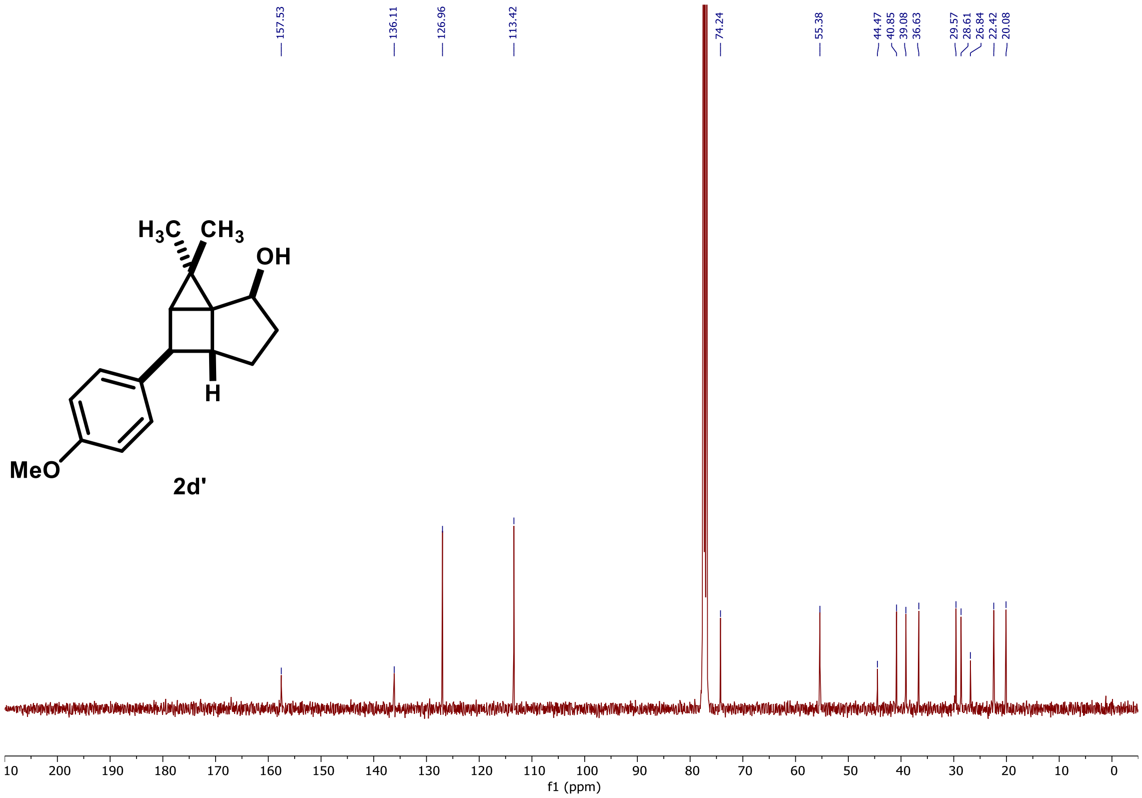

^{1}H-NMR of compound 2e (300 MHz, $CDCl_3$)

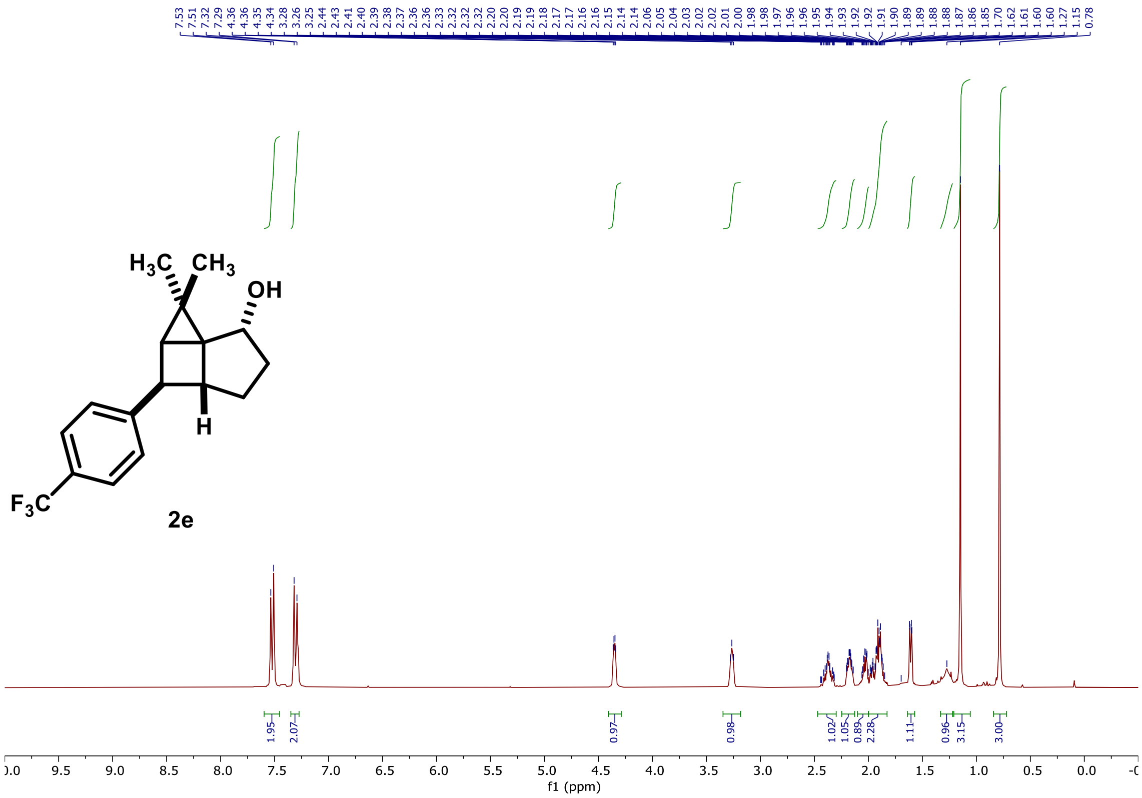

^{13}C-NMR of compound 2e (75 MHz, $CDCl_3$)

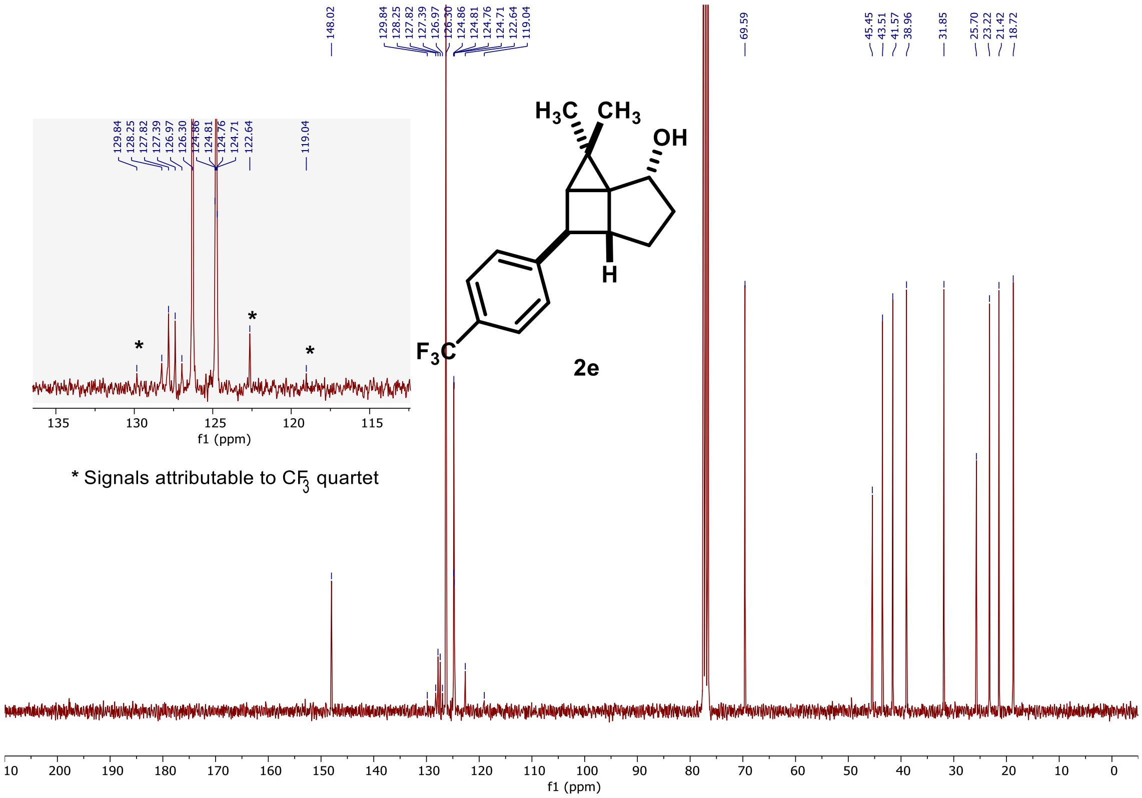

^{1}H-NMR of compound 2f (300 MHz, CD_2Cl_2)

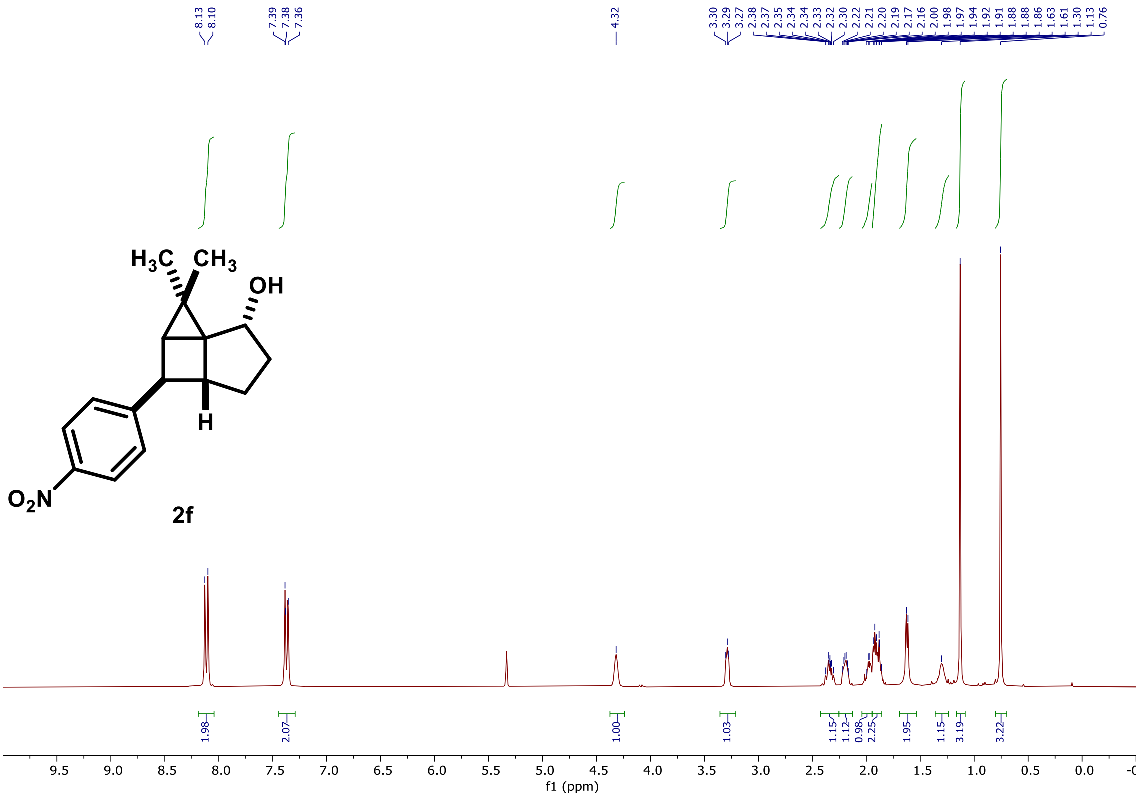

^{13}C-NMR of compound 2f (75 MHz, CD_2Cl_2)

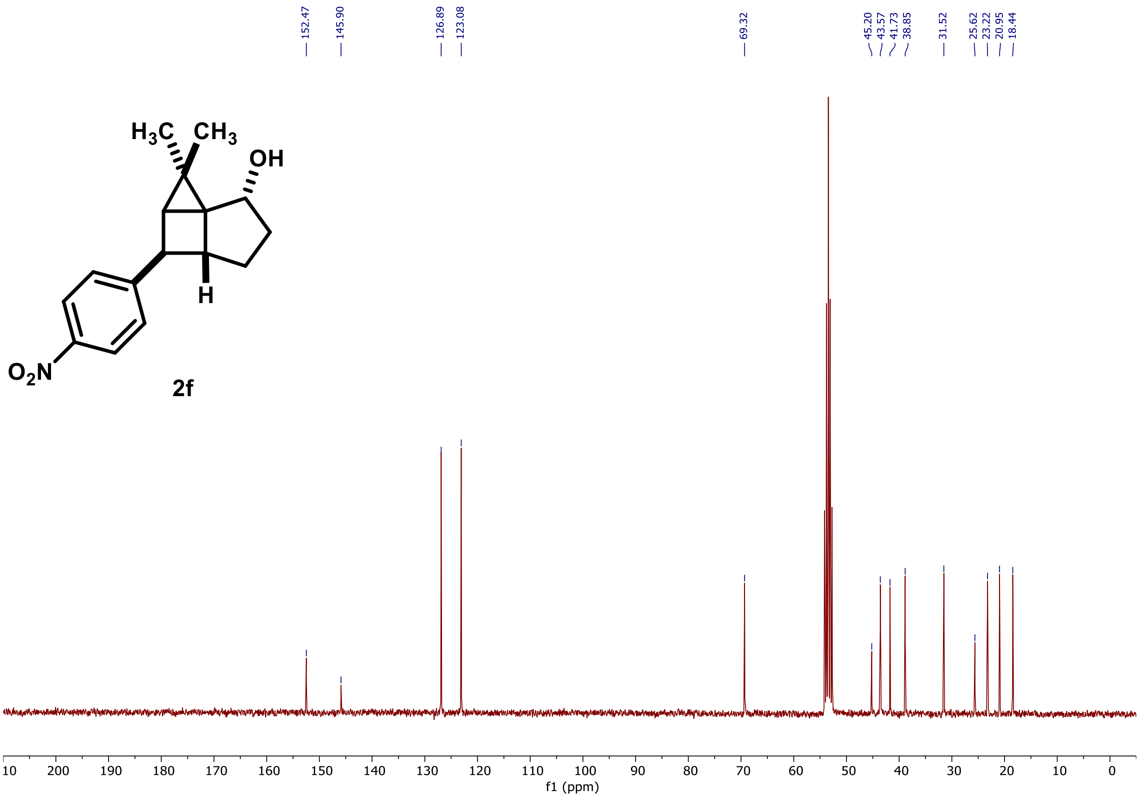

^{1}H-NMR of compound 2g (300 MHz, $CDCl_3$)

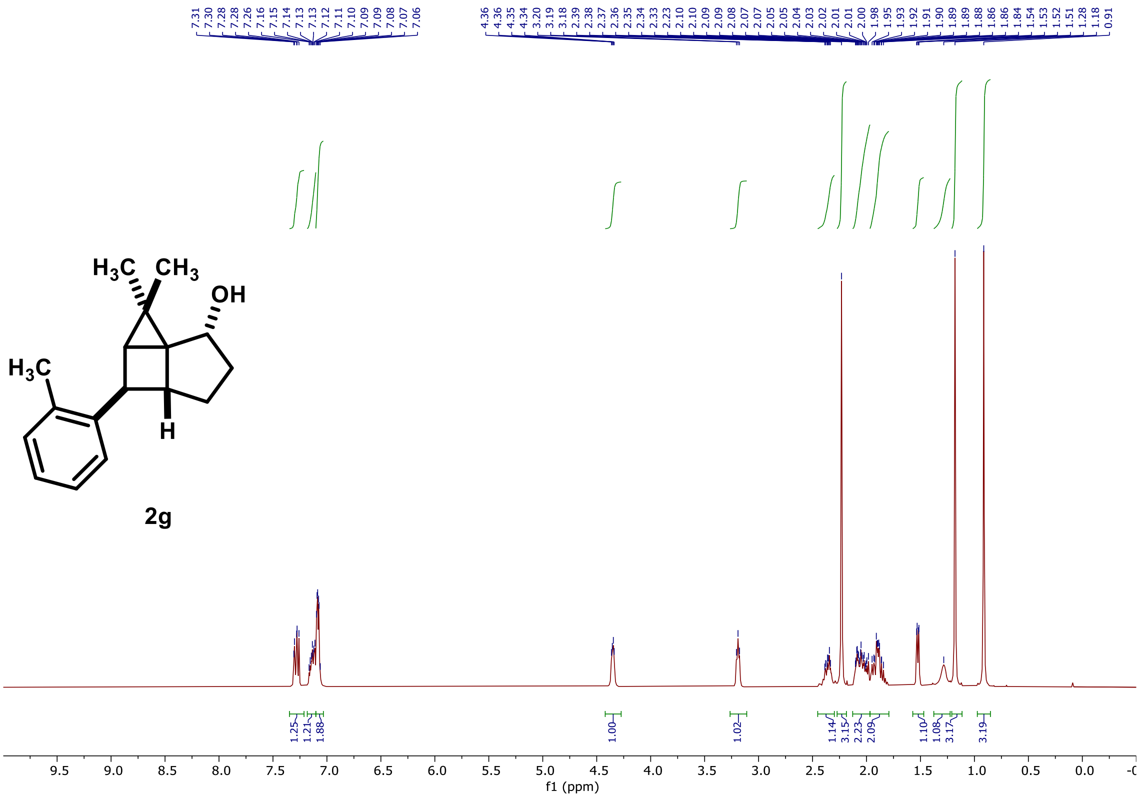

^{13}C-NMR of compound 2g (75 MHz, $CDCl_3$)

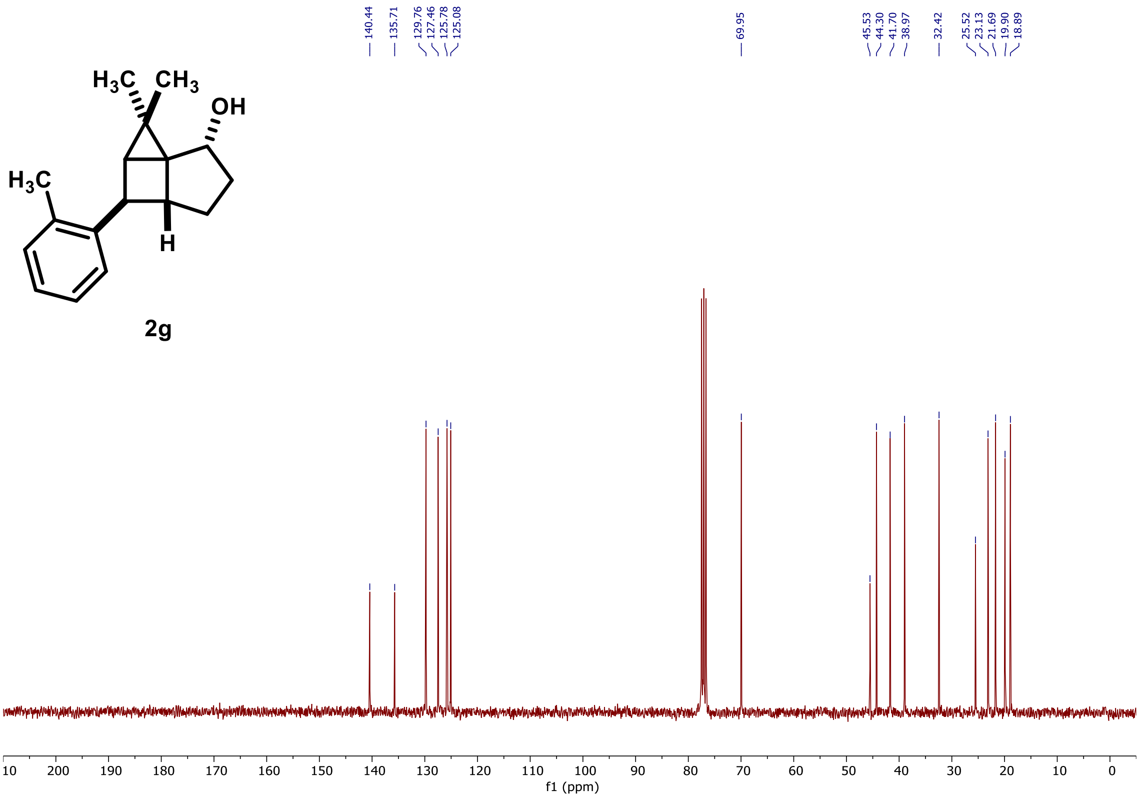

^{1}H-NMR of compound 2g’ (400 MHz, $CDCl_3$)

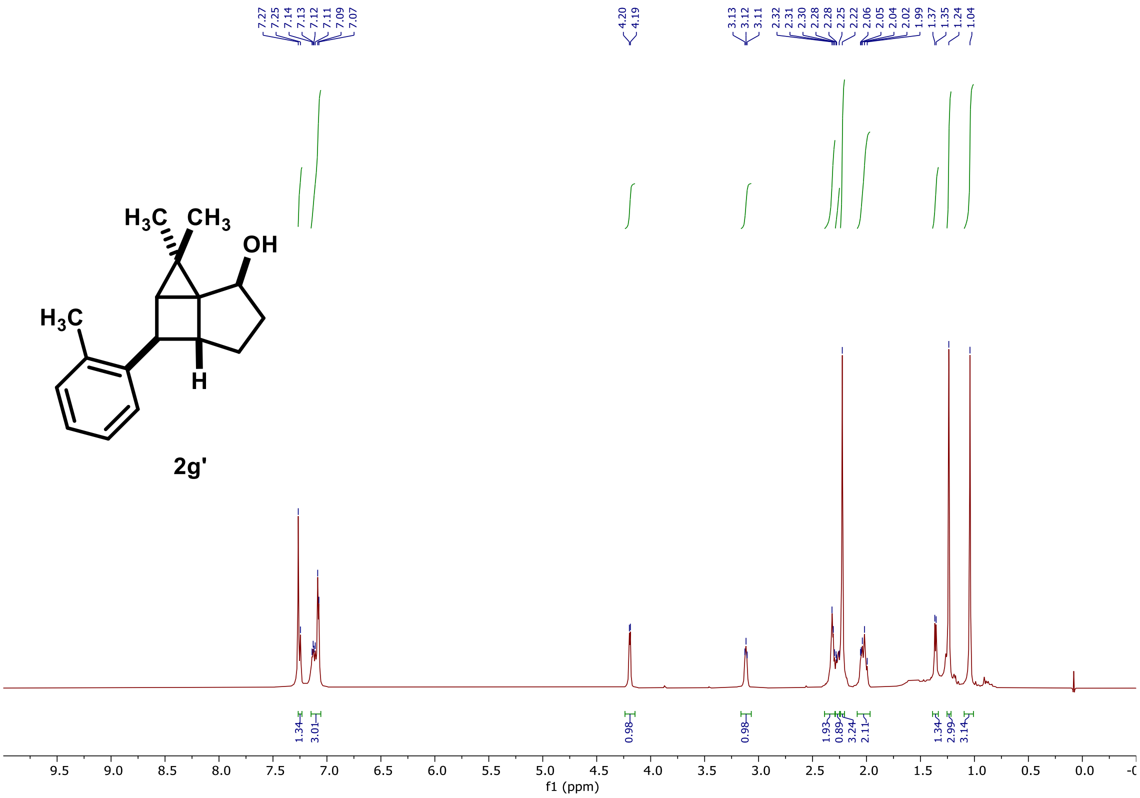

^{13}C-NMR of compound 2g’ (101 MHz, $CDCl_3$)

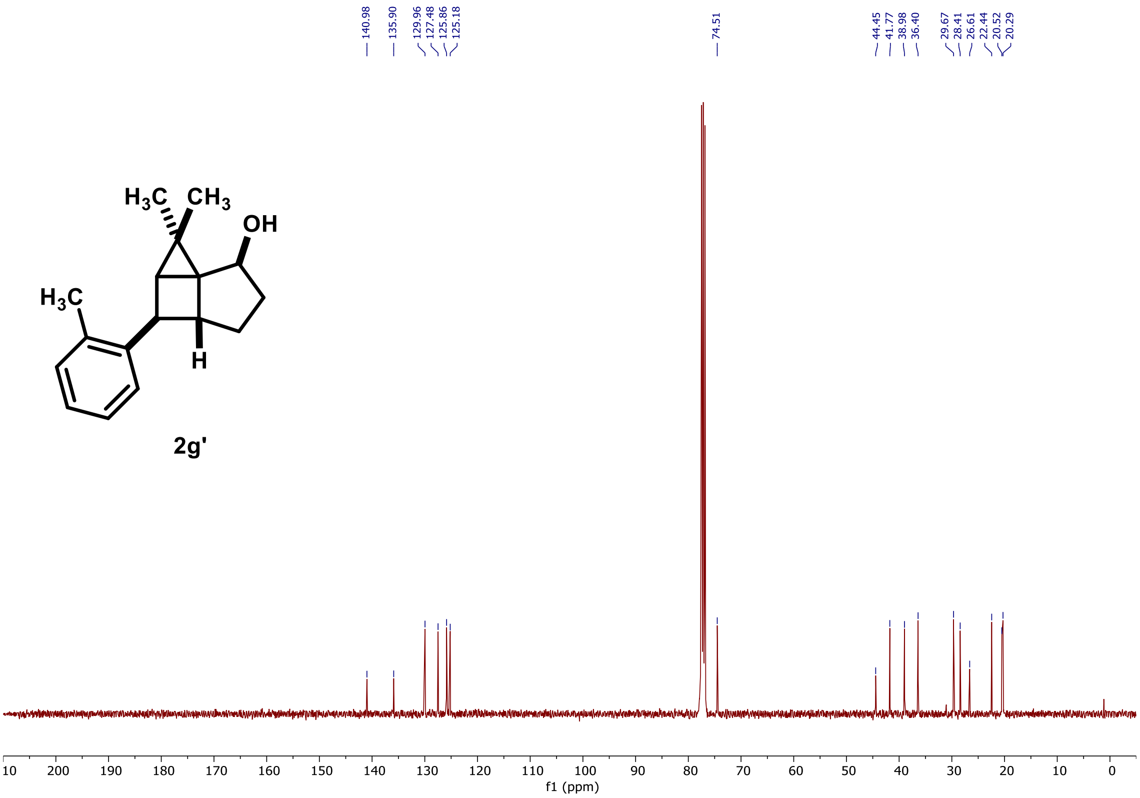

^{1}H-NMR of compound 2h (400 MHz, $CDCl_3$)

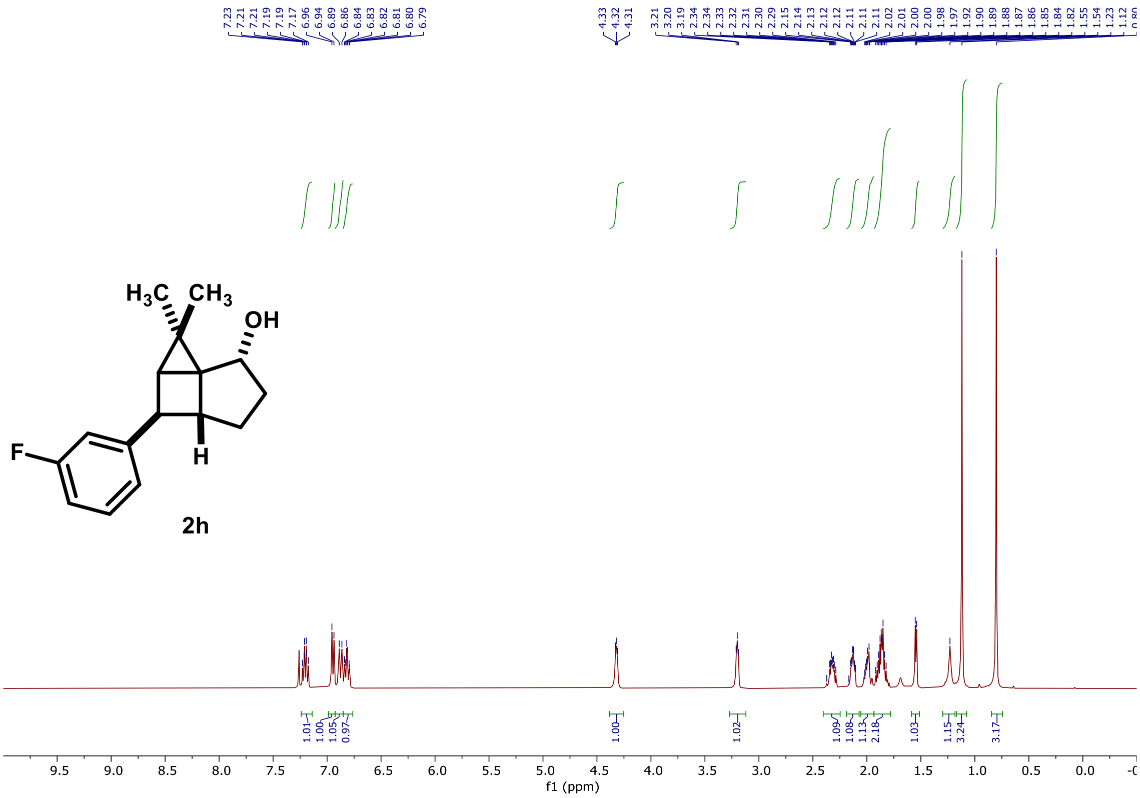

^{13}C-NMR of compound 2h (101 MHz, $CDCl_3$)

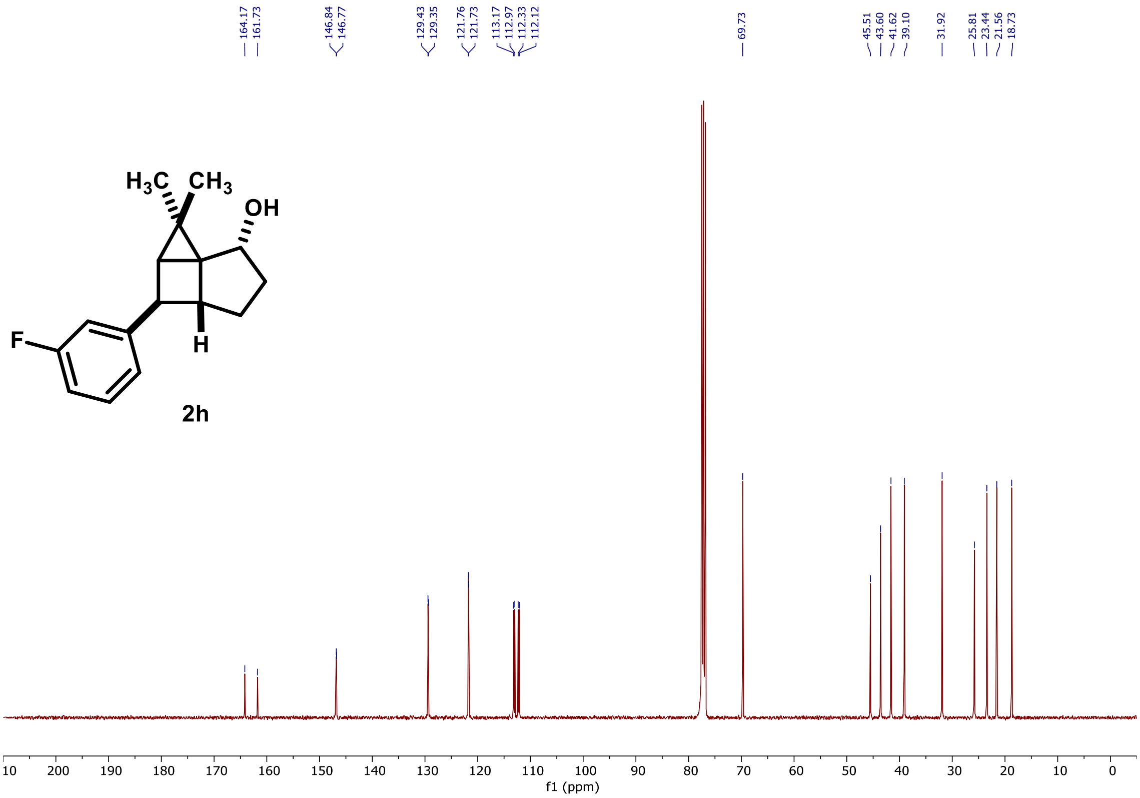

^{1}H-NMR of compound 2h (400 MHz, $CDCl_3$)

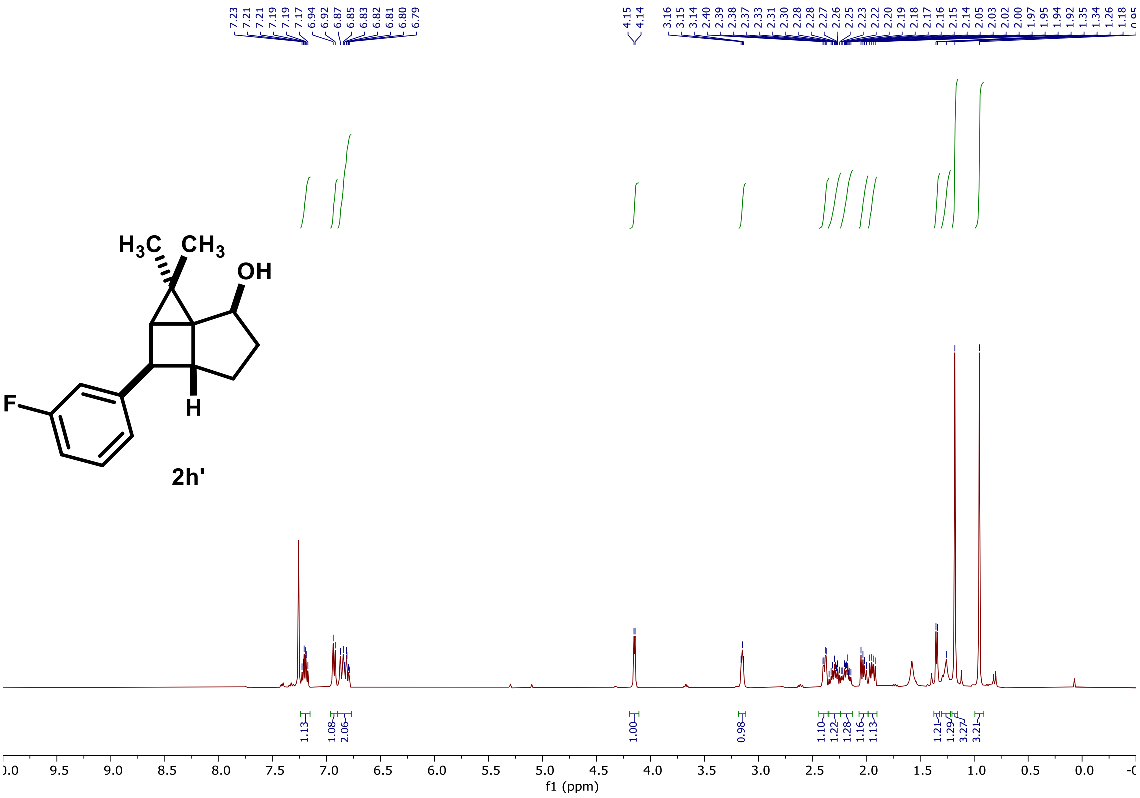

^{13}C-NMR of compound 2h’ (101 MHz, $CDCl_3$)

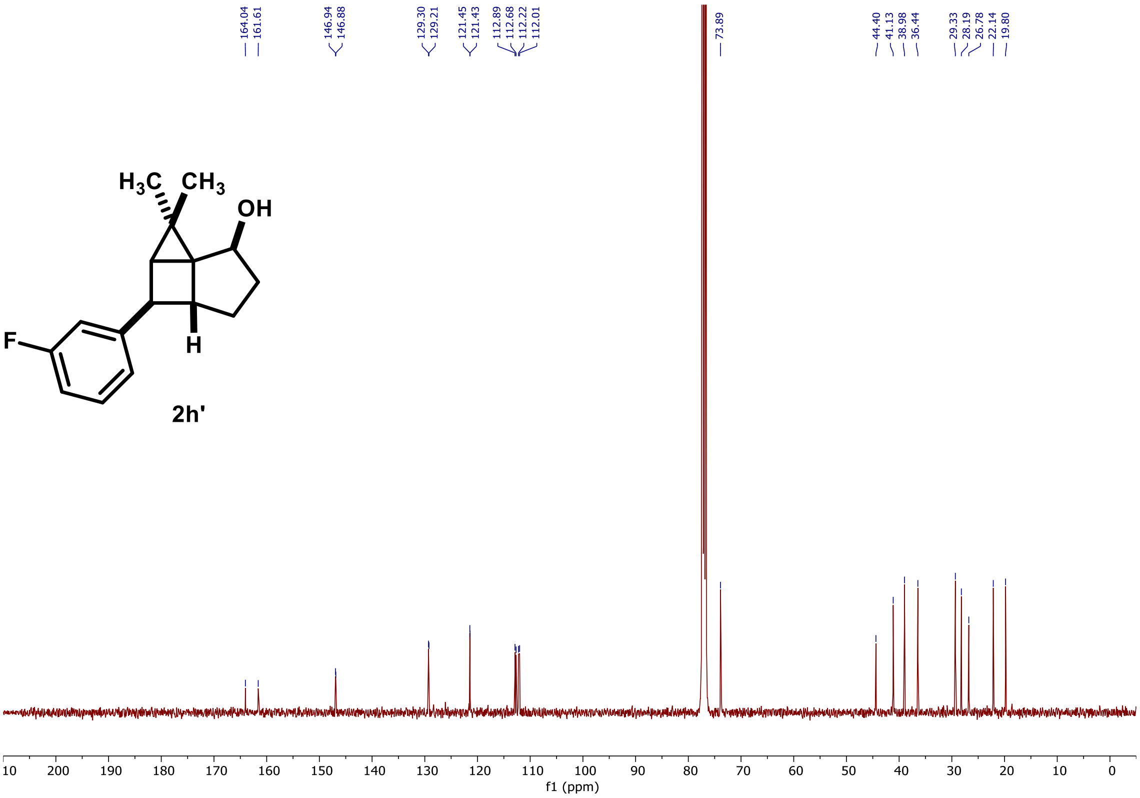

^{1}H-NMR of compound 2i (300 MHz, $CDCl_3$)

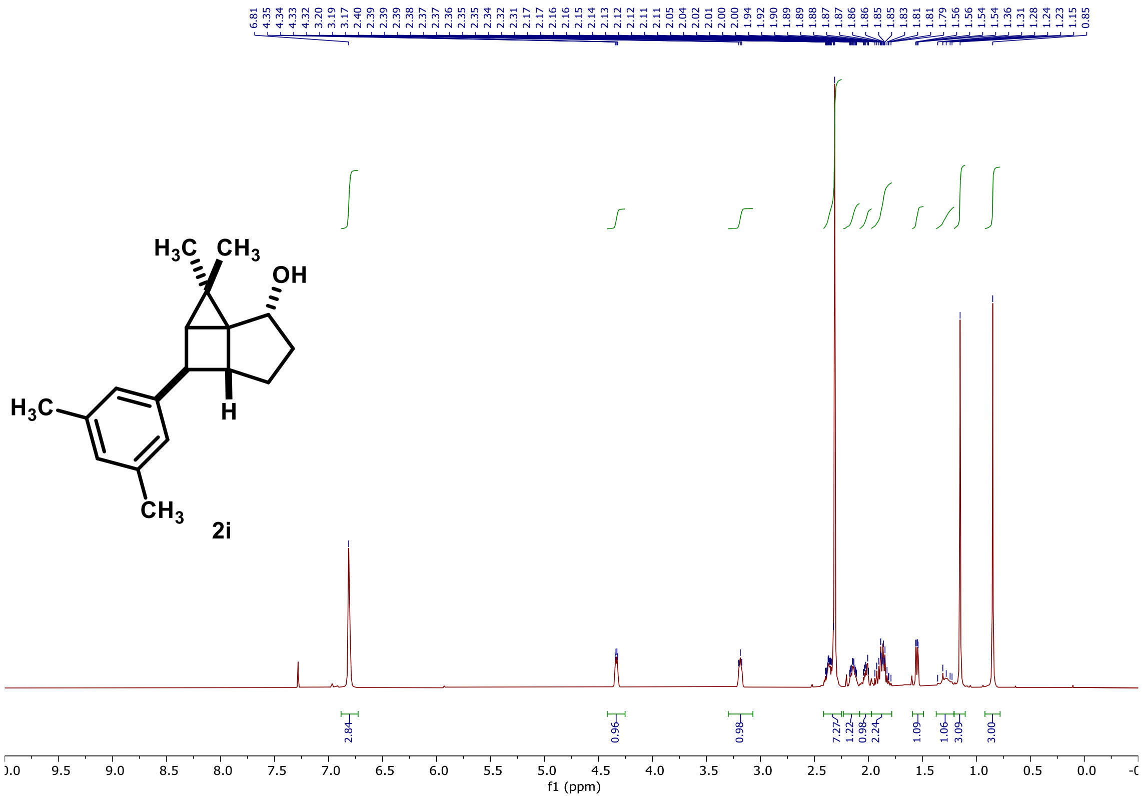

^{13}C-NMR of compound 2i (75 MHz, $CDCl_3$)

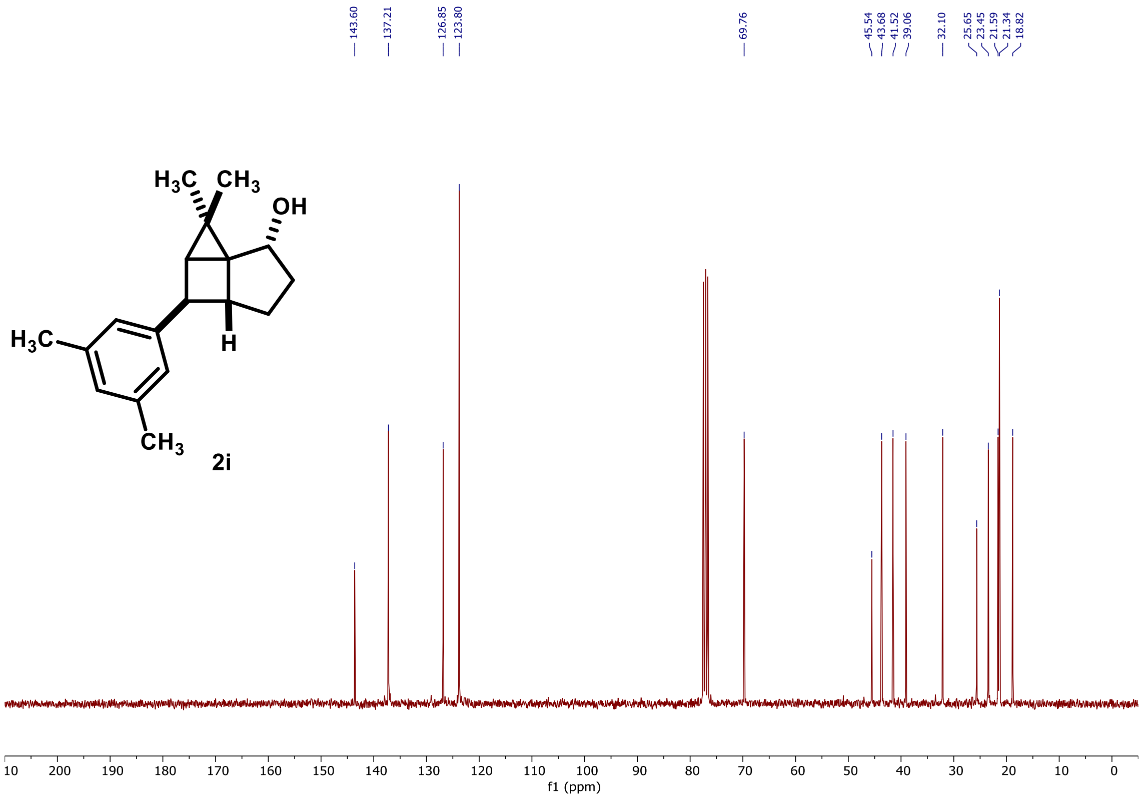

^{1}H-NMR of compound 2i' (400 MHz, $CDCl_3$)

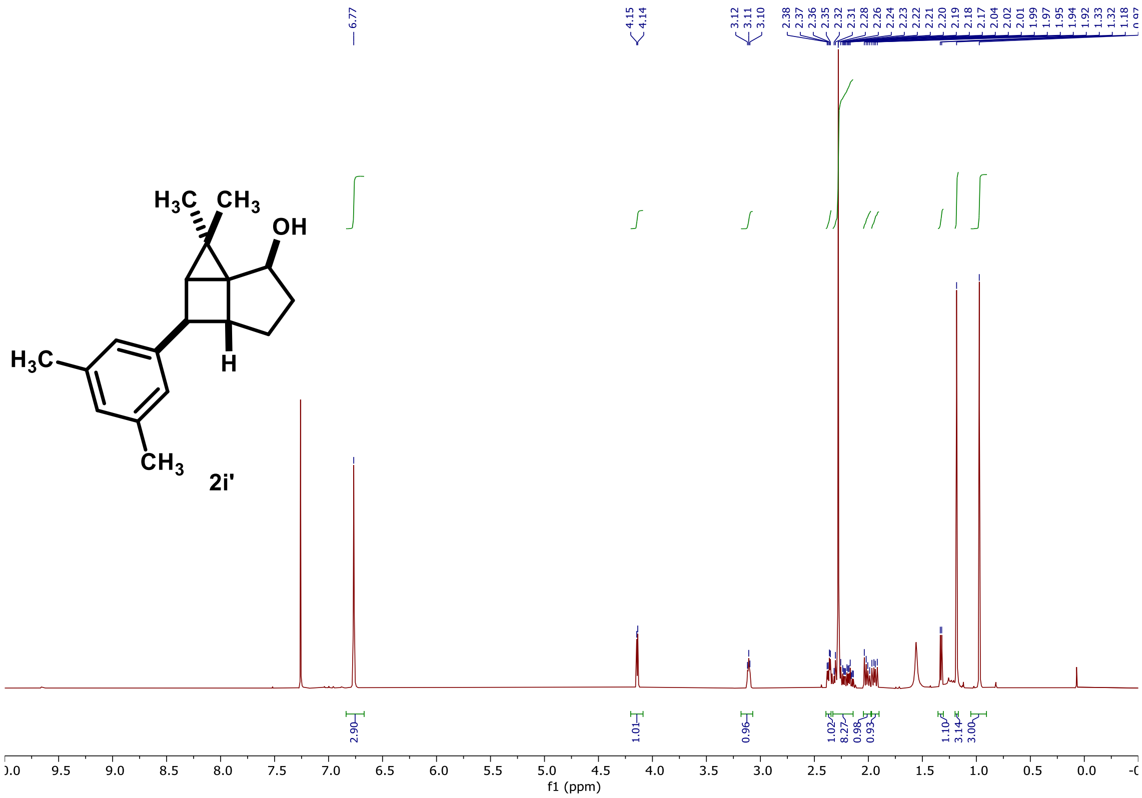

^{13}C-NMR of compound 2i' (101 MHz, $CDCl_3$)

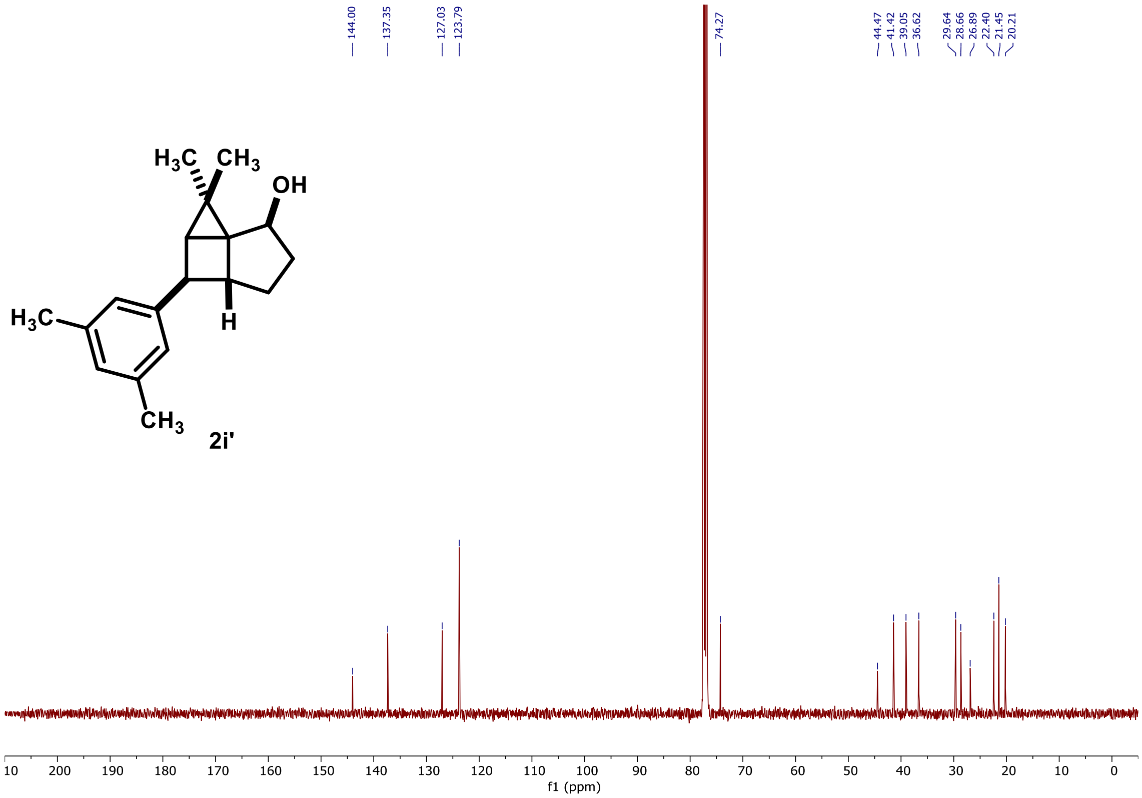

^{1}H-NMR of compound 2j (300 MHz, $CDCl_3$)

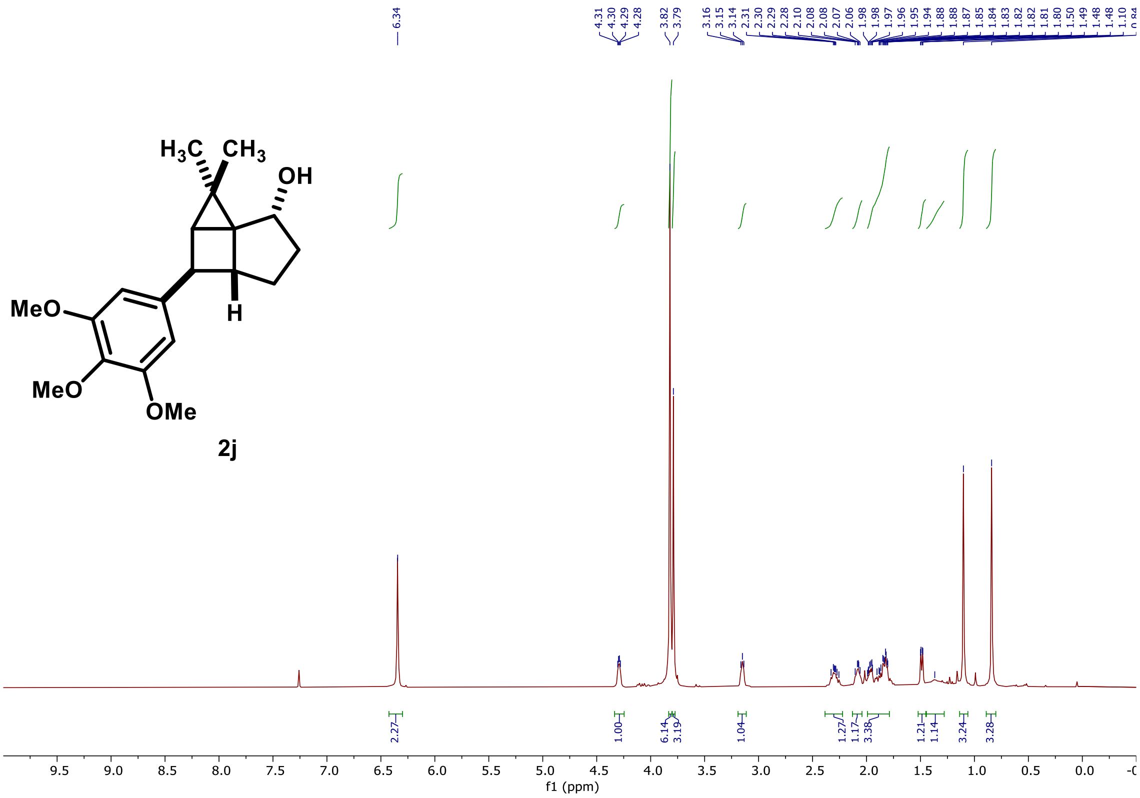

^{13}C-NMR of compound 2j (75 MHz, $CDCl_3$)

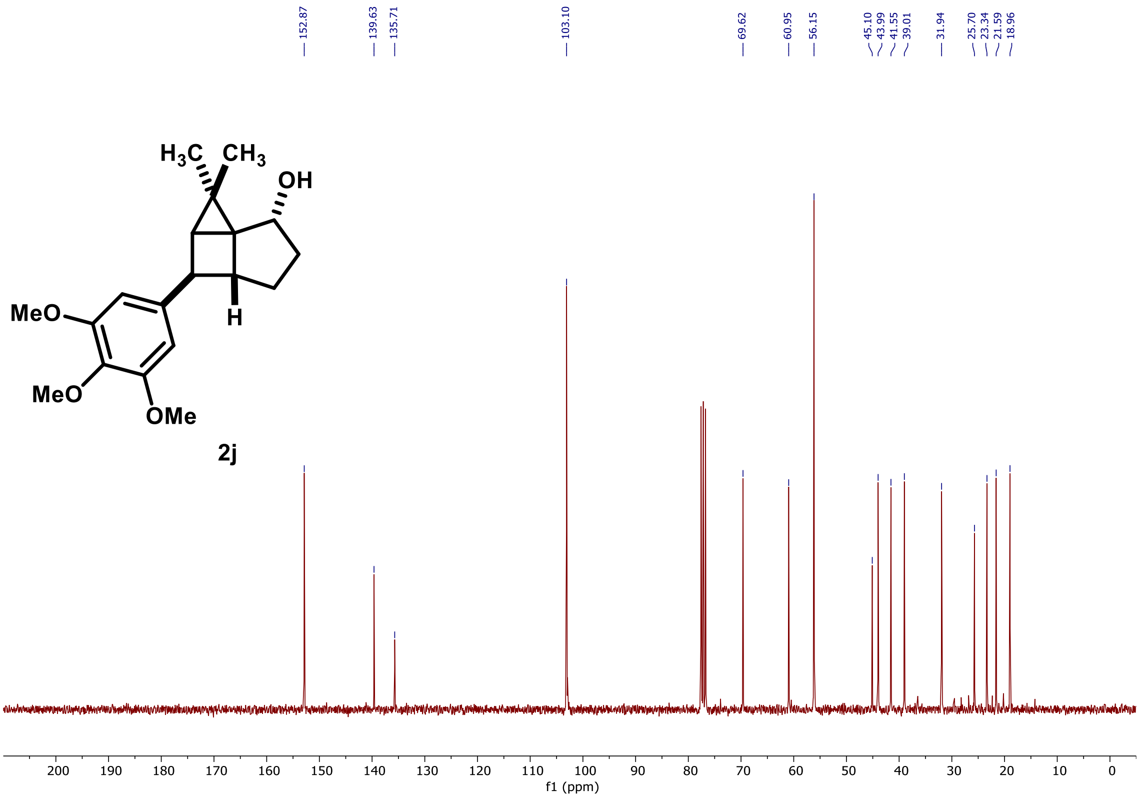

^{1}H-NMR of compound 2k (300 MHz, $CDCl_3$)

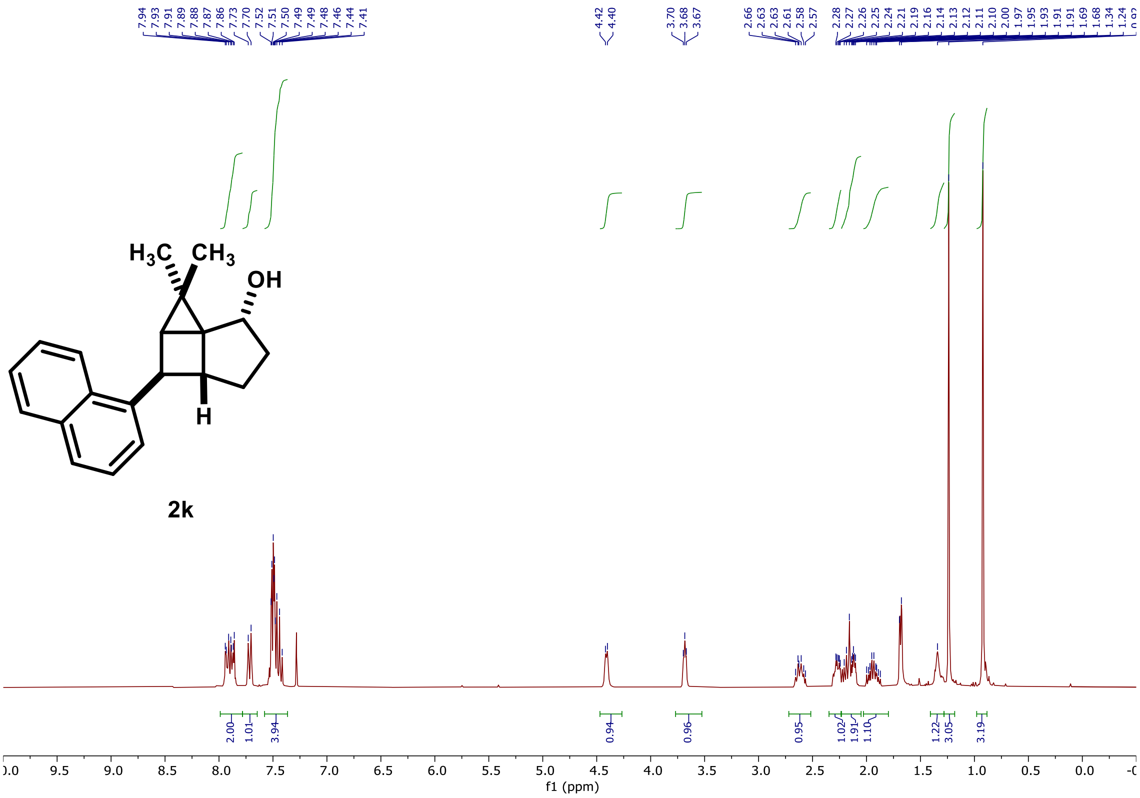

^{13}C-NMR of compound 2k (75 MHz, $CDCl_3$)

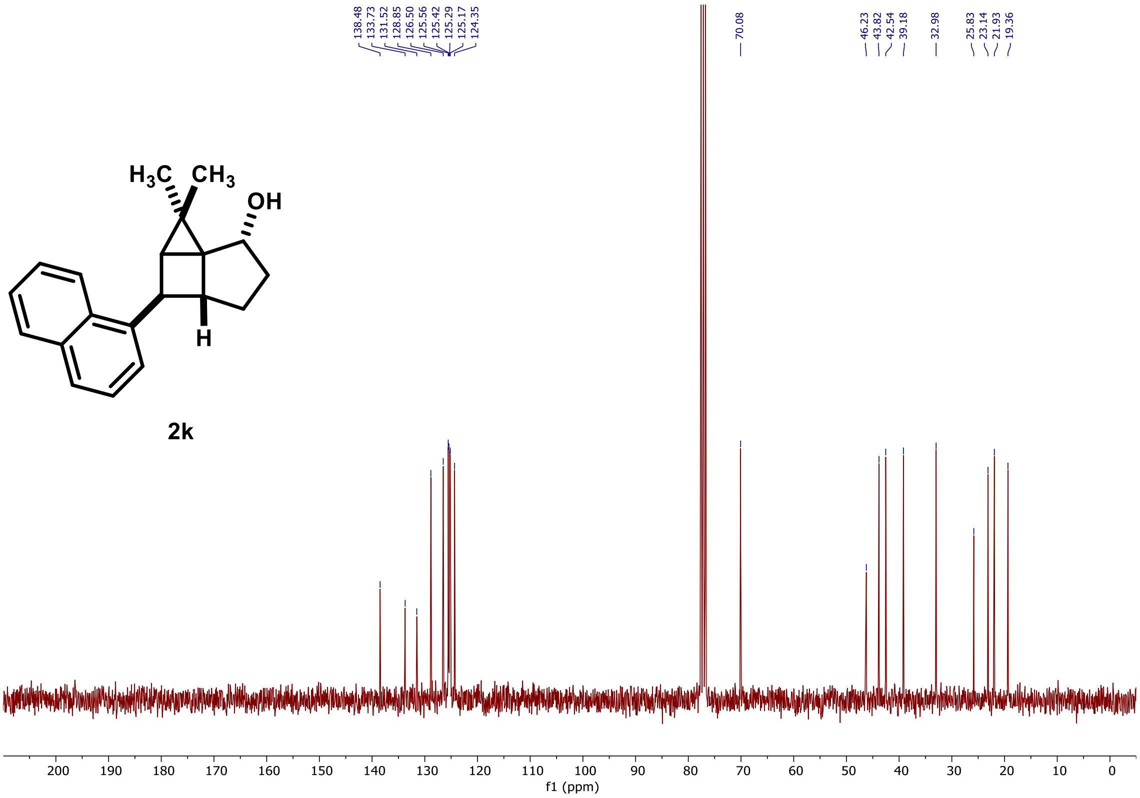

^{1}H-NMR of compound 2k’ (400 MHz, $CDCl_3$)

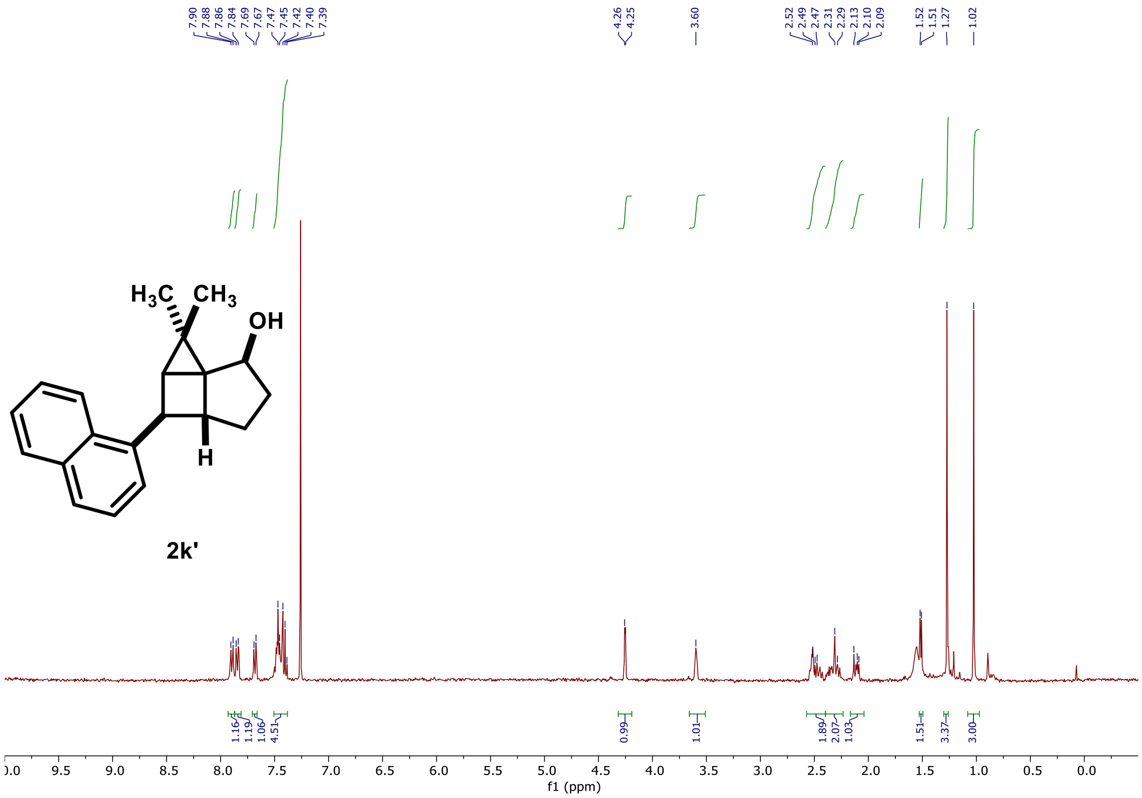

^{13}C-NMR of compound 2k’ (101 MHz, $CDCl_3$)

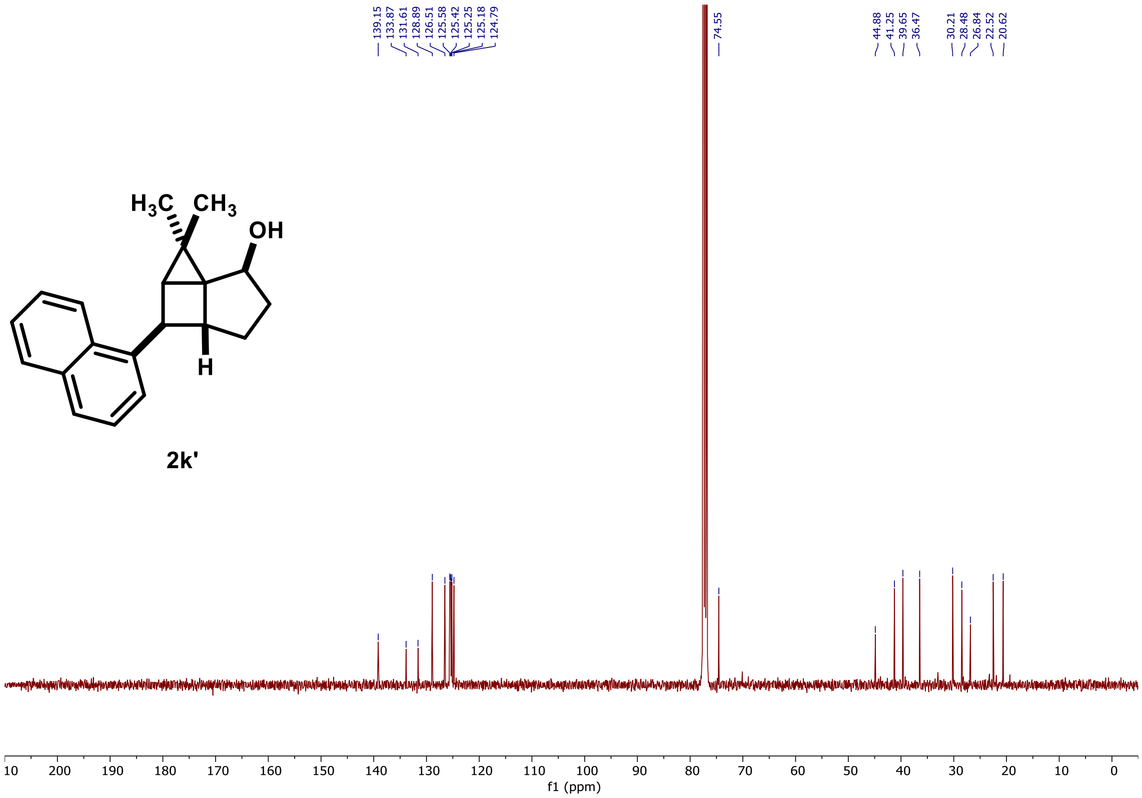

^{1}H-NMR of compound 2l (400 MHz, $CDCl_3$, mixture of diastereoisomers)

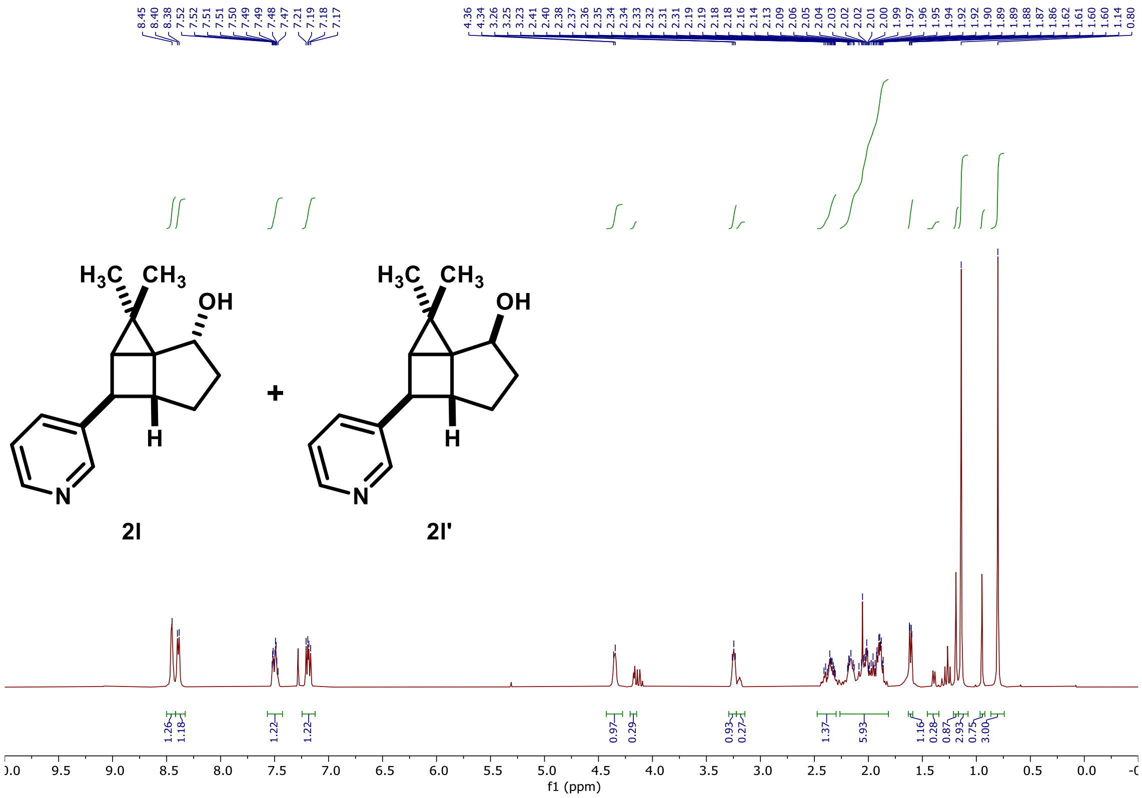

^{13}C-NMR of compound 2l (101 MHz, $CDCl_3$, mixture of diastereoisomers)

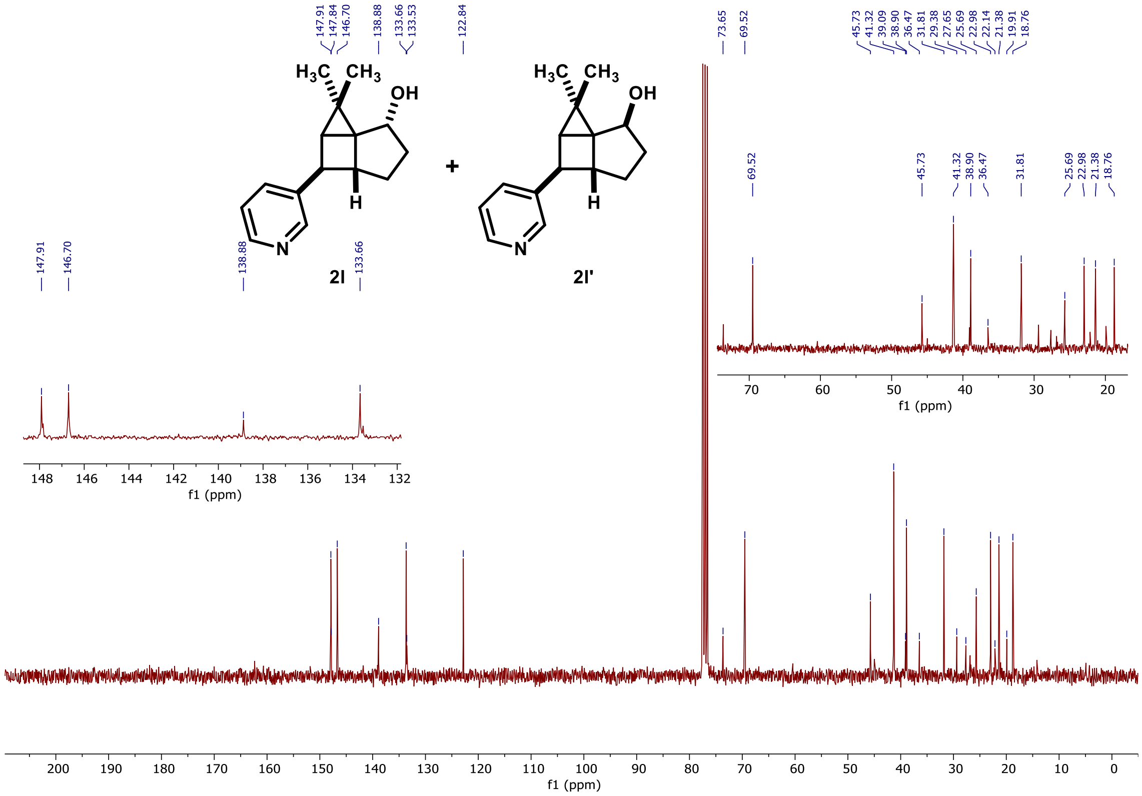

^{1}H-NMR of compound 2m (300 MHz, $CDCl_3$)

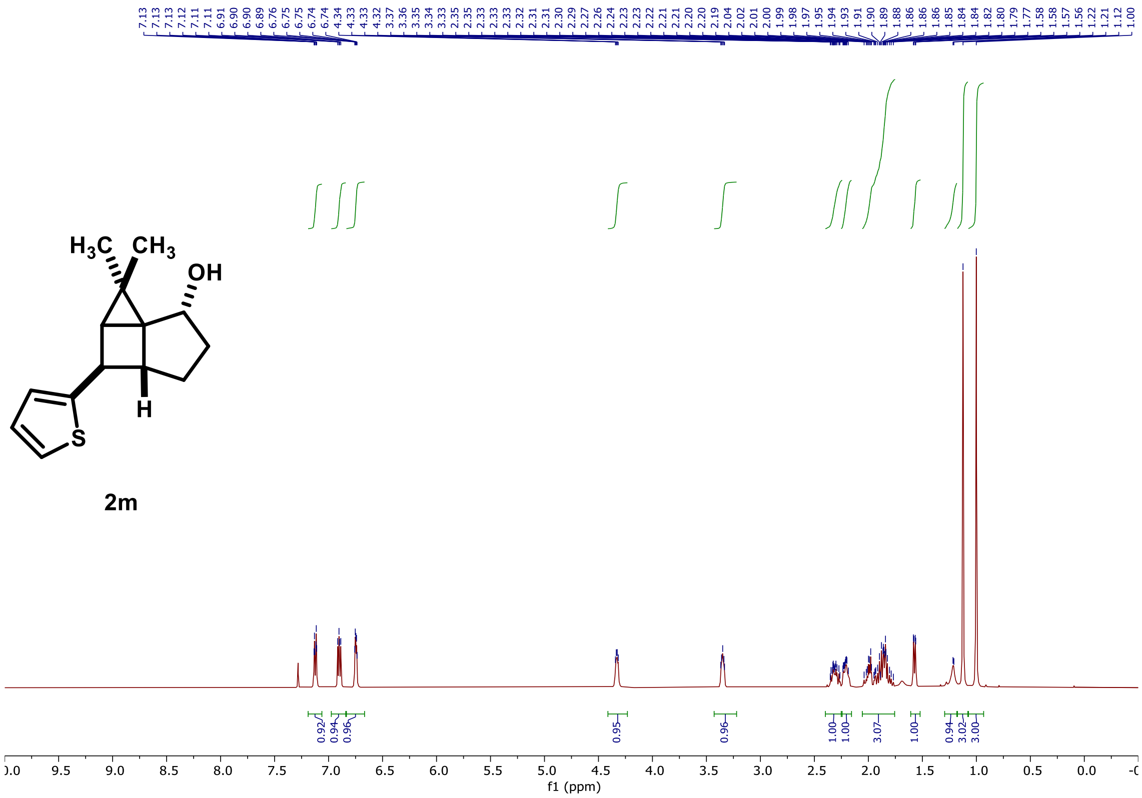

^{13}C-NMR of compound 2m (75 MHz, $CDCl_3$)

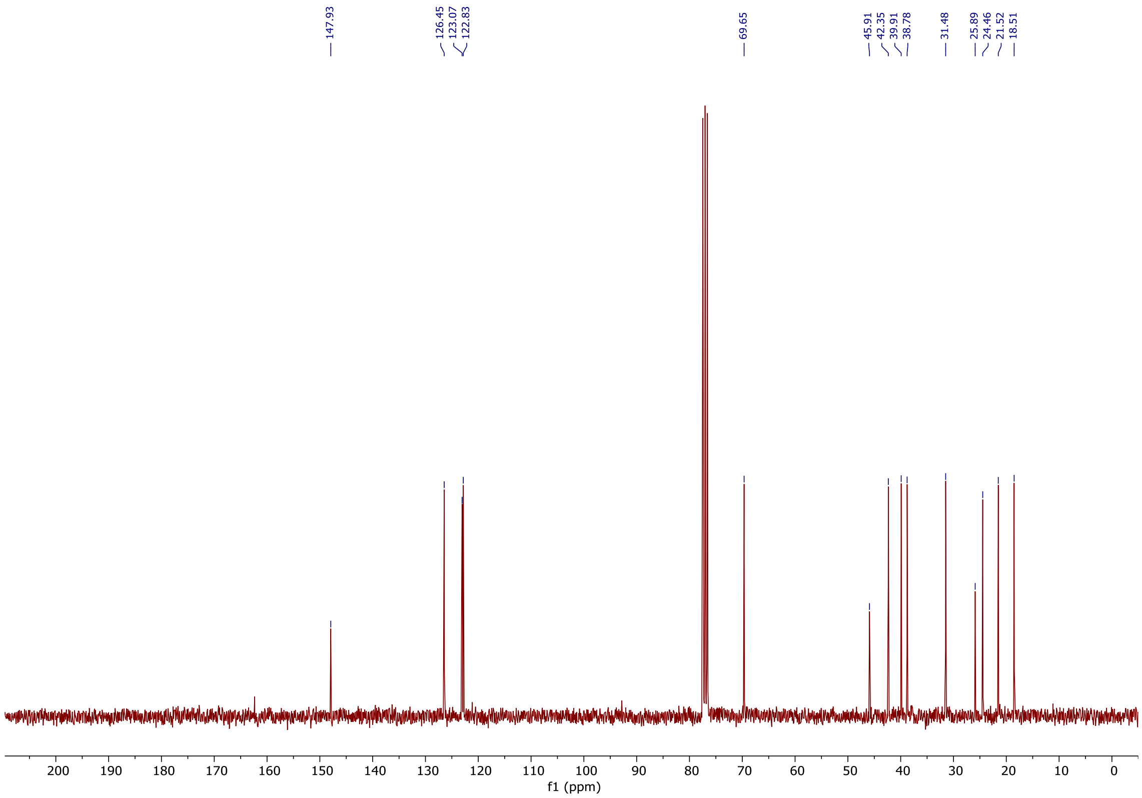

^{1}H-NMR of compound 2m' (400 MHz, $CDCl_3$)

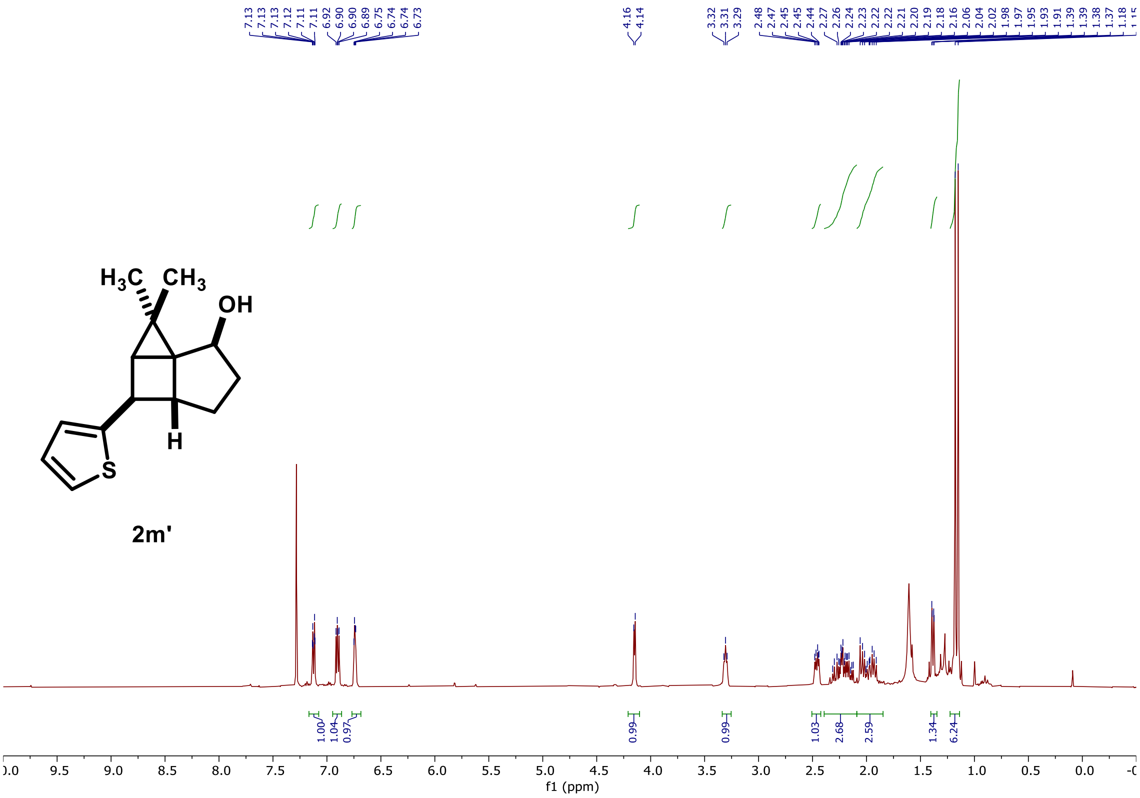

^{13}C-NMR of compound 2m (101 MHz, $CDCl_3$)

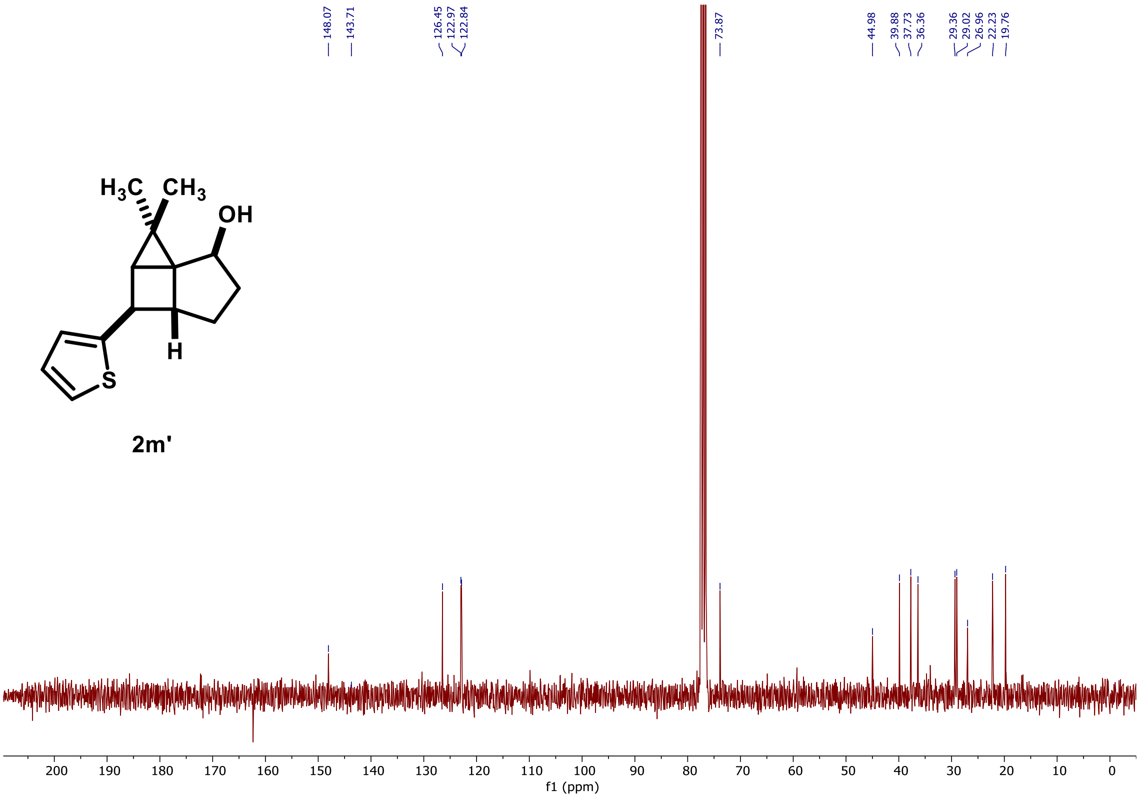

^{1}H-NMR of compound 2n (300 MHz, $CDCl_3$)

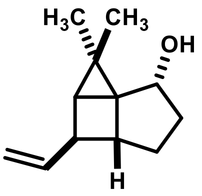

2n

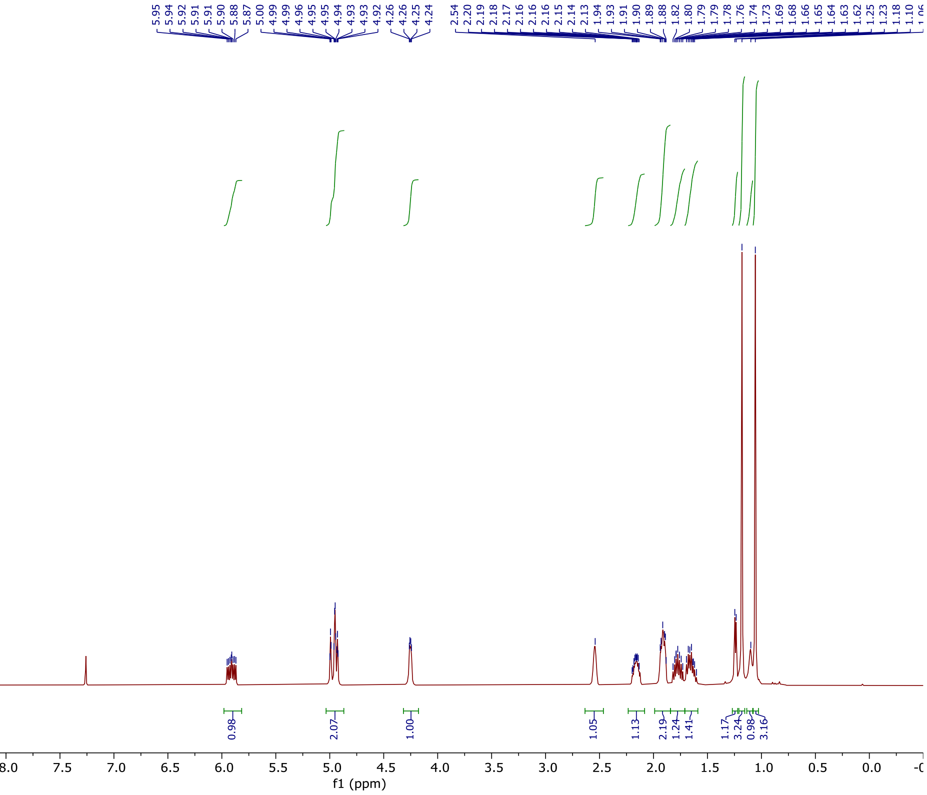

^{13}C-NMR of compound 2n (101 MHz, $CDCl_3$)

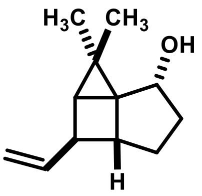

2n

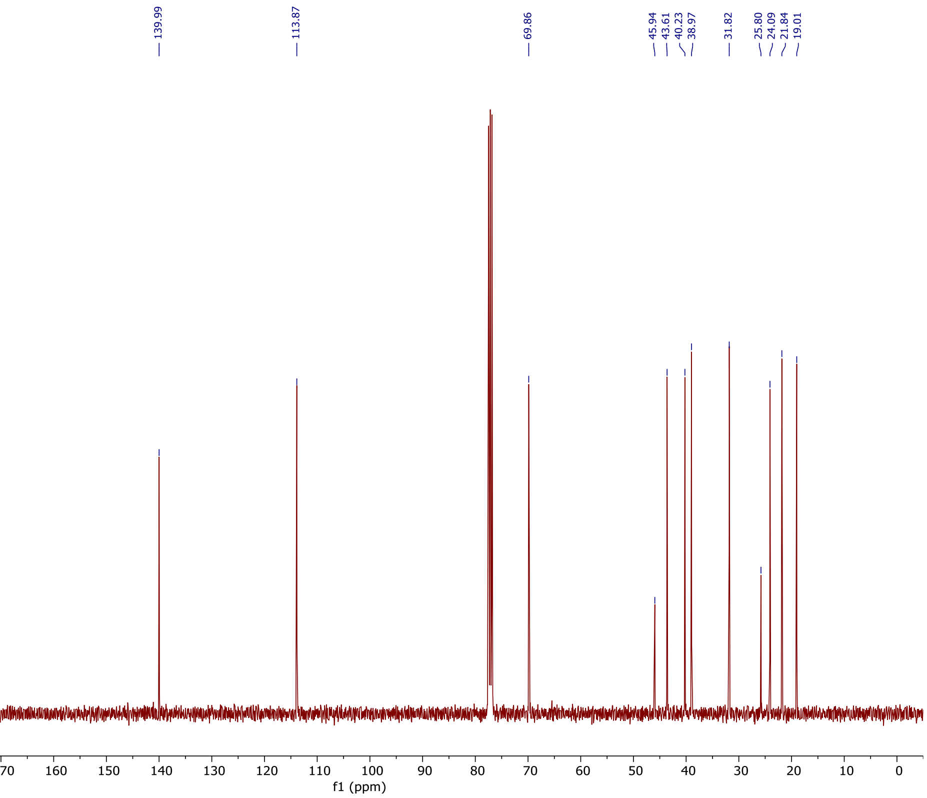

^{1}H-NMR of compound 2n’ (400 MHz, $CDCl_3$)

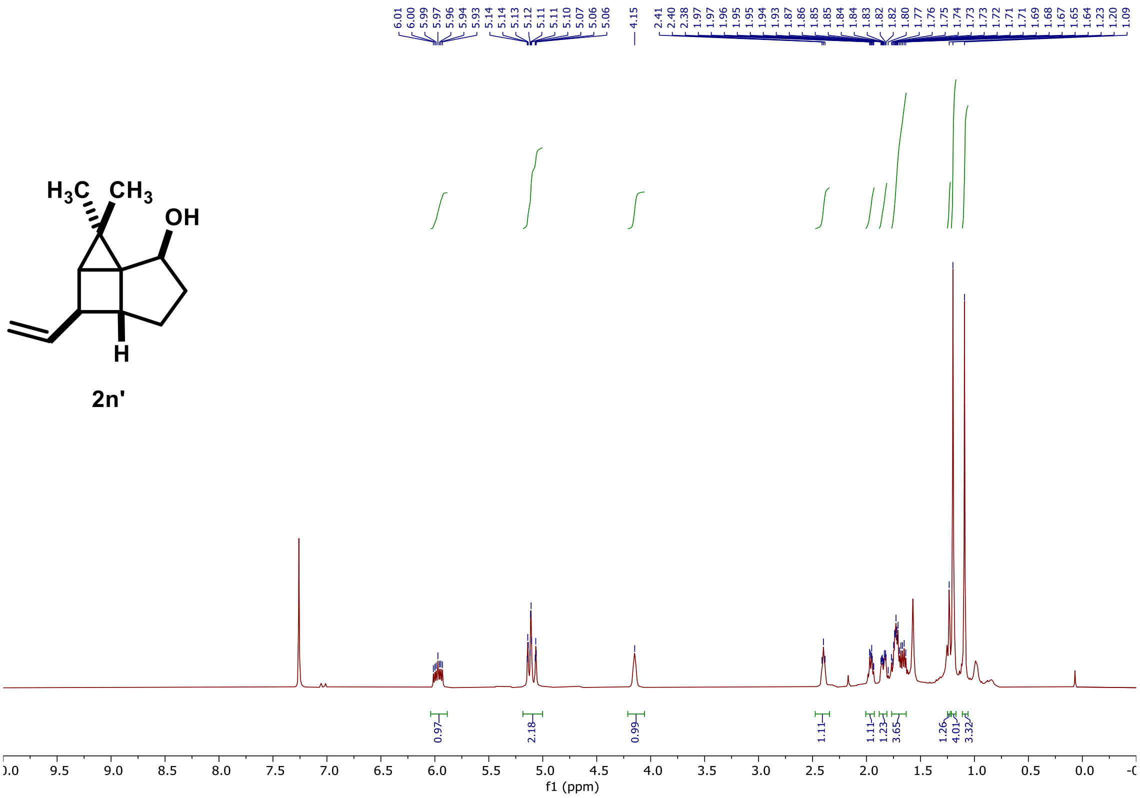

^{13}C-NMR of compound 2n’ (101 MHz, $CDCl_3$)

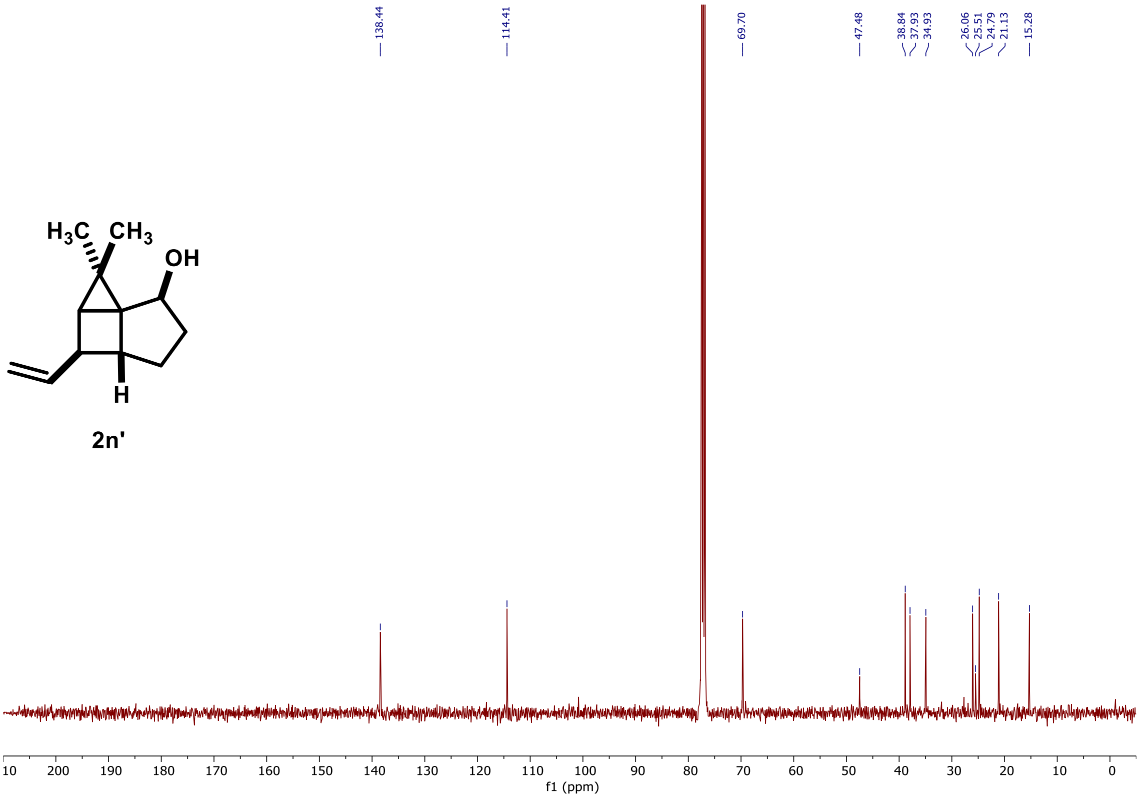

^{1}H-NMR of compound 2o (400 MHz, $CDCl_3$, for all collected fractions from flash chromatography)

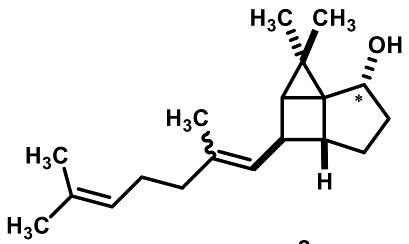

2o
[4 diastereoisomers]

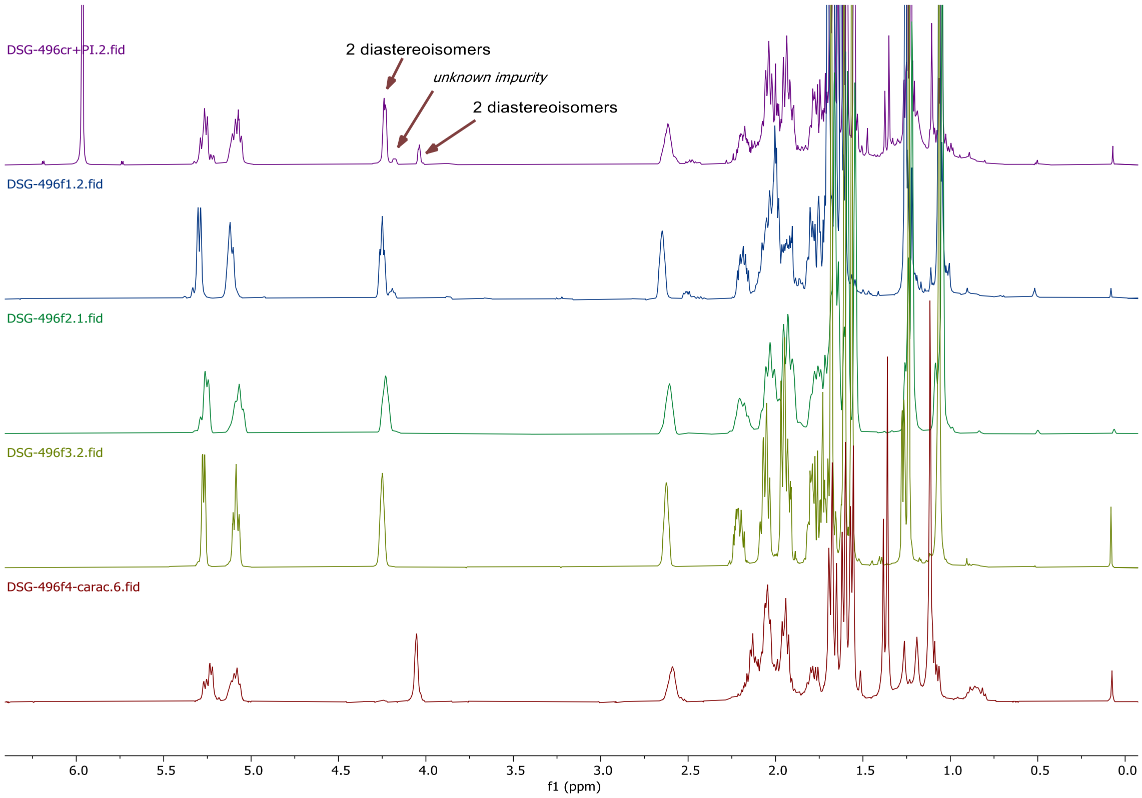

^{1}H-NMR of compound 2o-F1 (400 MHz, $CDCl_3$, major diastereoisomer, fraction-1)

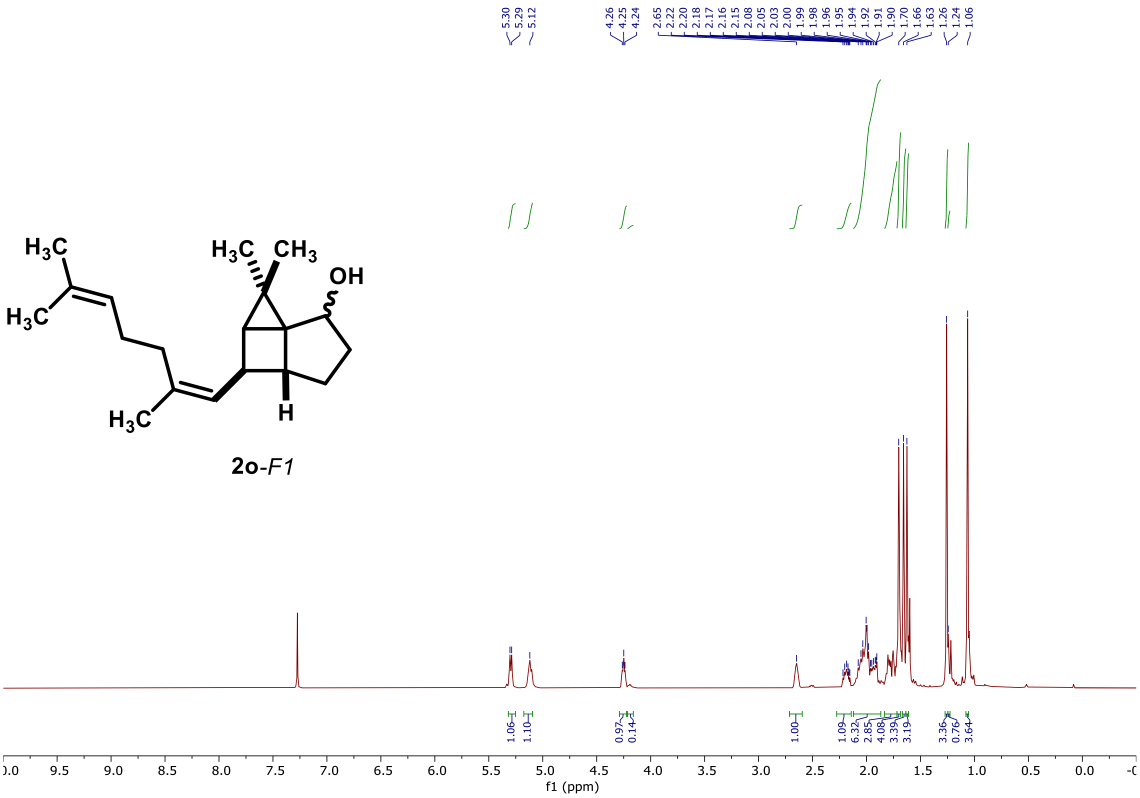

^{13}C-NMR of compound 2o-F1 (101 MHz, $CDCl_3$, major diastereoisomer, fraction-1)

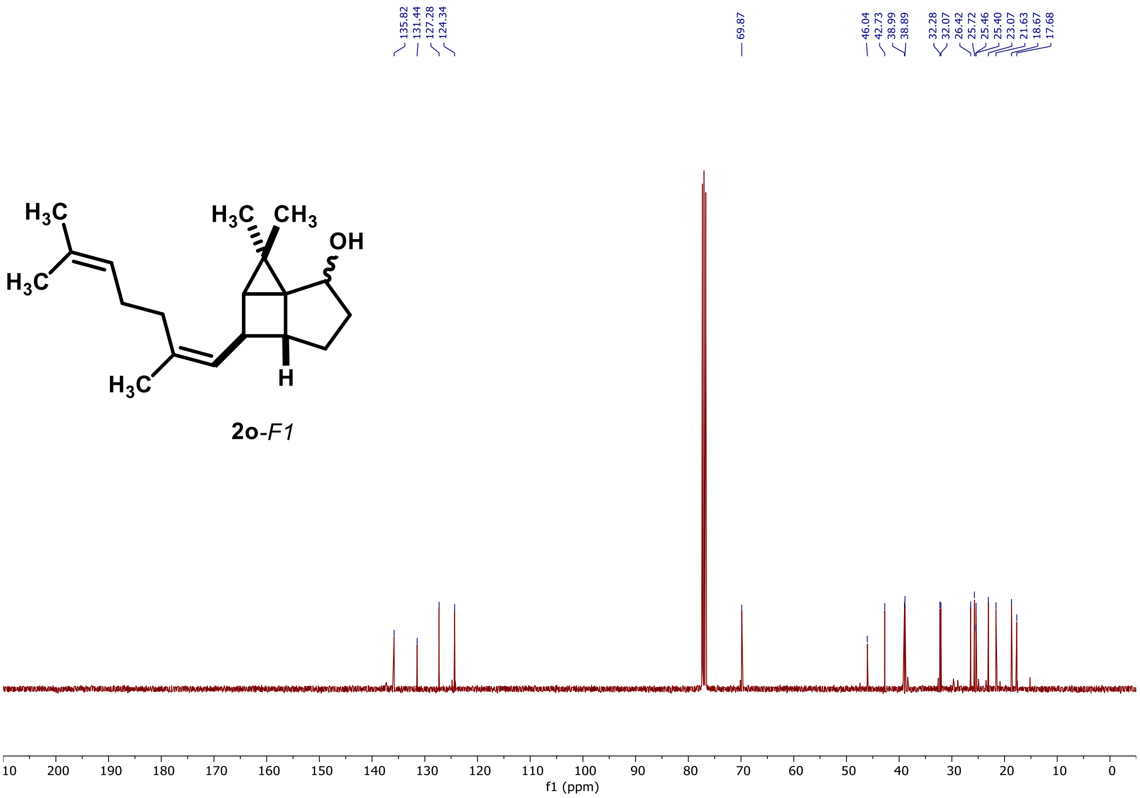

Selective nOe experiments of compound 2o-F1 ($CDCl_3$)

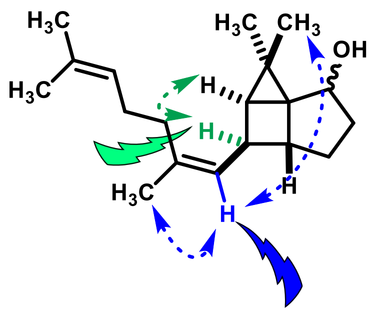

2o-*F1*

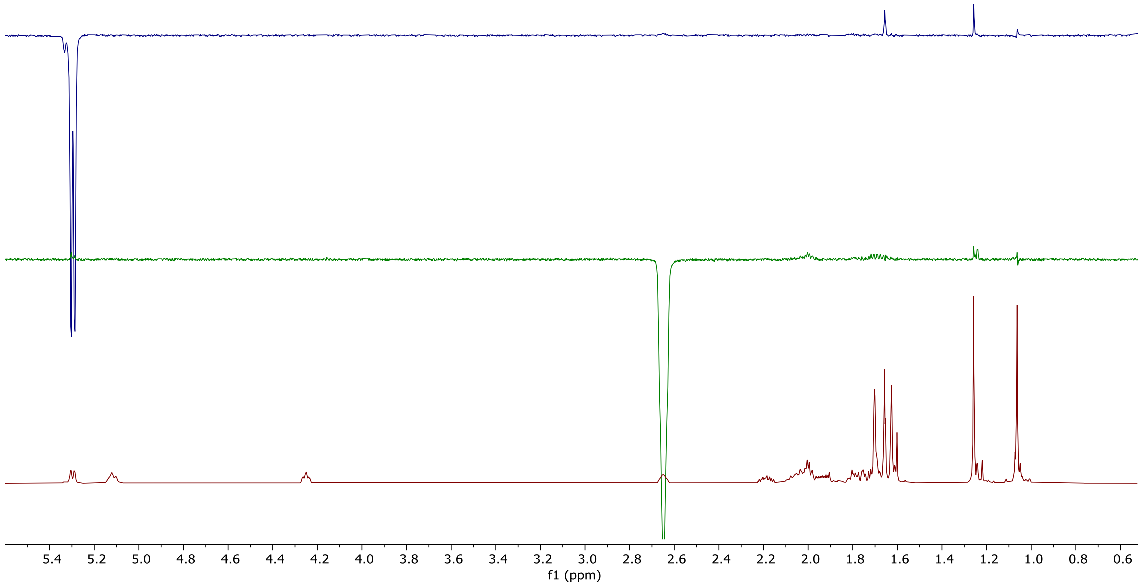

^{1}H-NMR of compound 2o-F3 (400 MHz, $CDCl_3$, fraction-3)

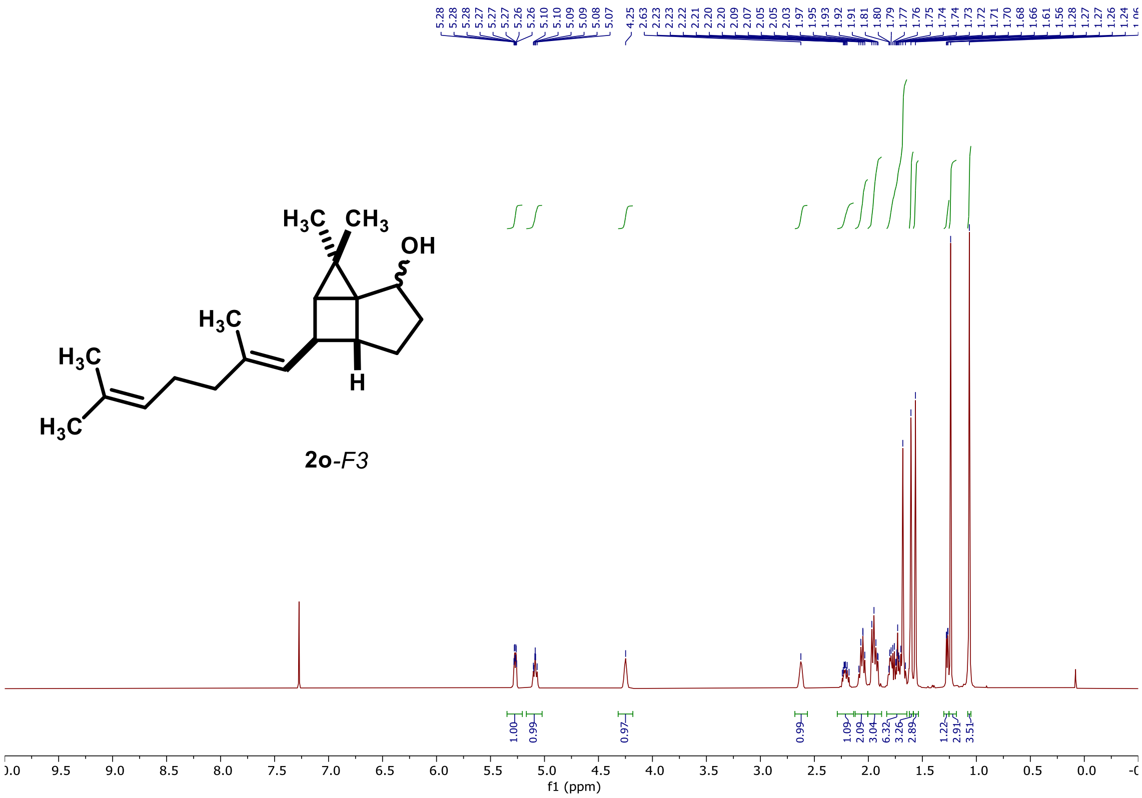

^{13}C-NMR of compound 2o-F3 (75 MHz, $CDCl_3$)

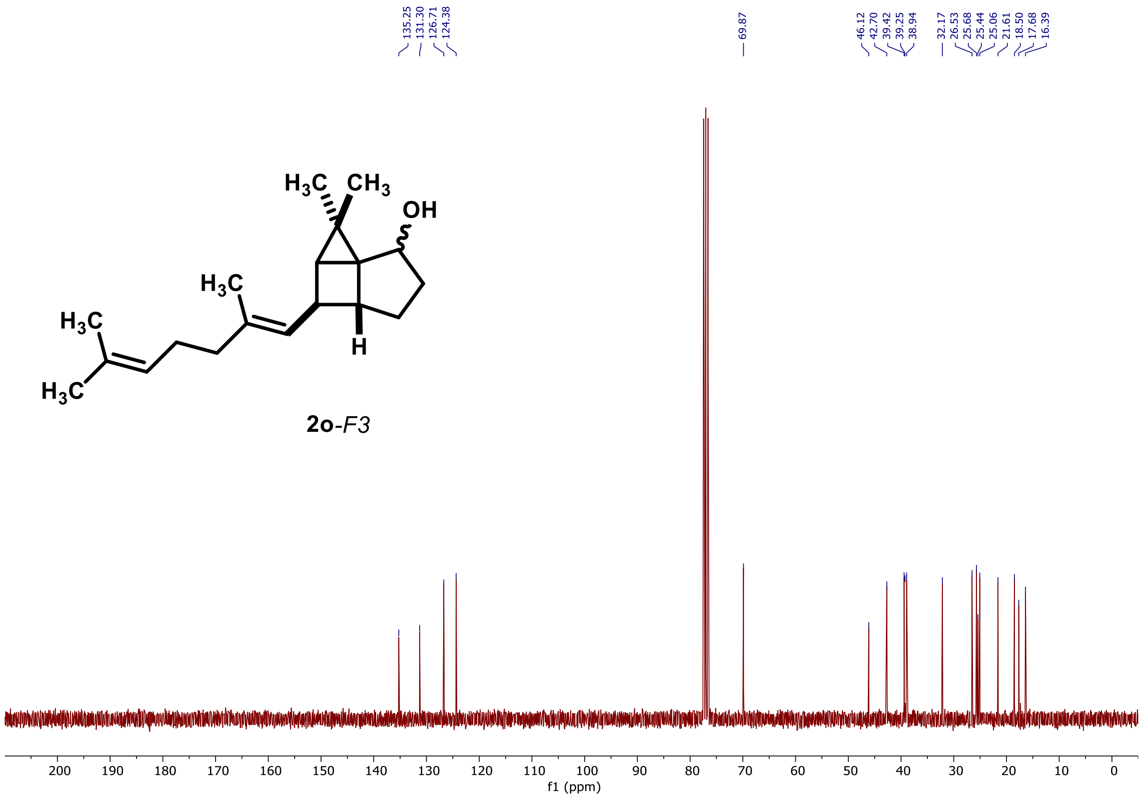

Selective nOe experiments of compound 2o-F3 (CDCl₃)

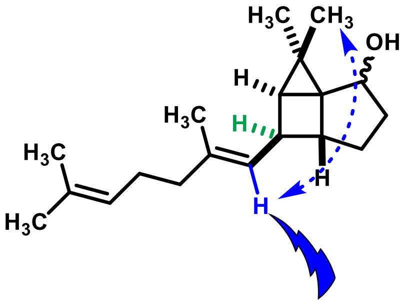

2o-*F3*

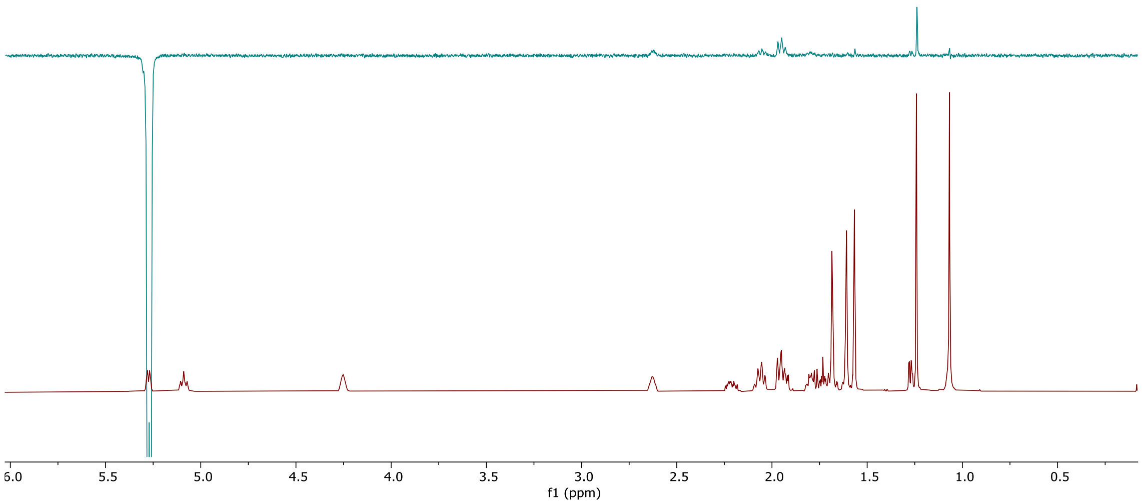

^{1}H-NMR of compound 2o-F4 (400 MHz, $CDCl_3$, mixture of diastereoisomers, fraction-4)

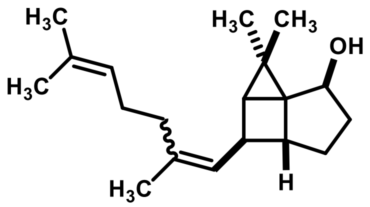

2o-*F4*

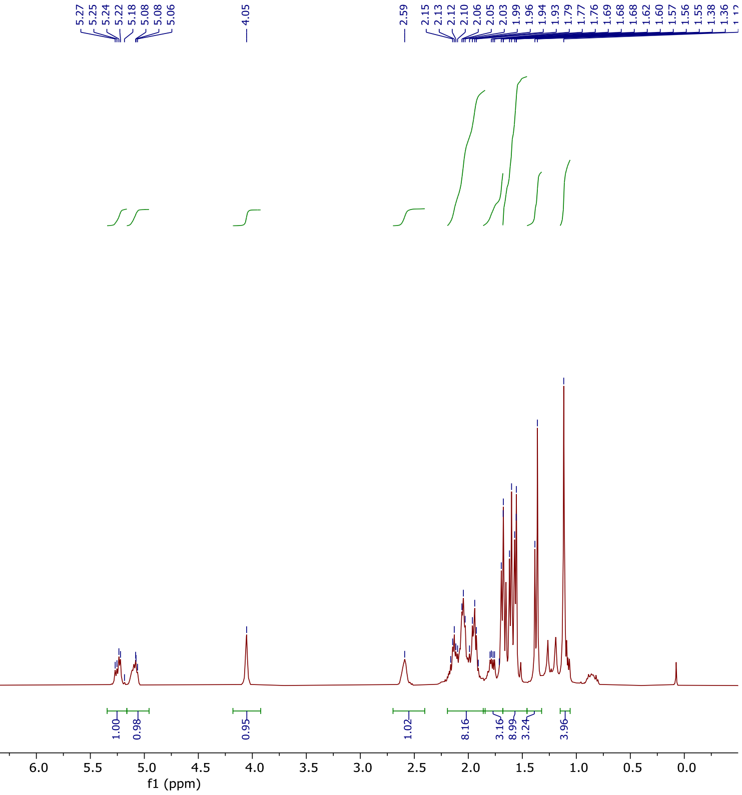

^{13}C-NMR of compound 2o-F4 (101 MHz, $CDCl_3$, mixture of diastereoisomers, fraction-4)

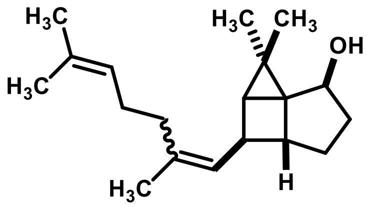

2o-*F4*

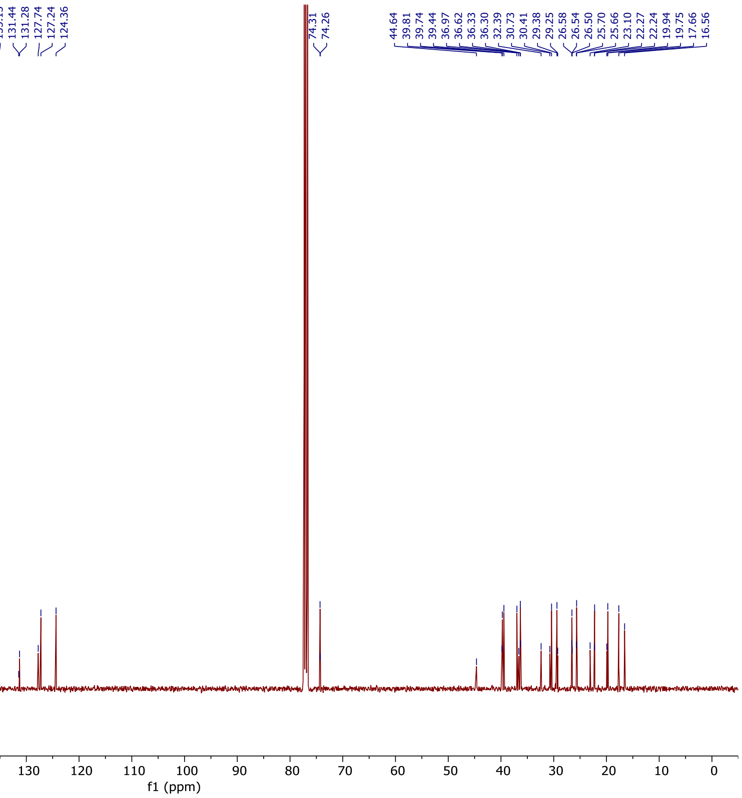

^{1}H-NMR of compound 2p (300 MHz, $CDCl_3$)

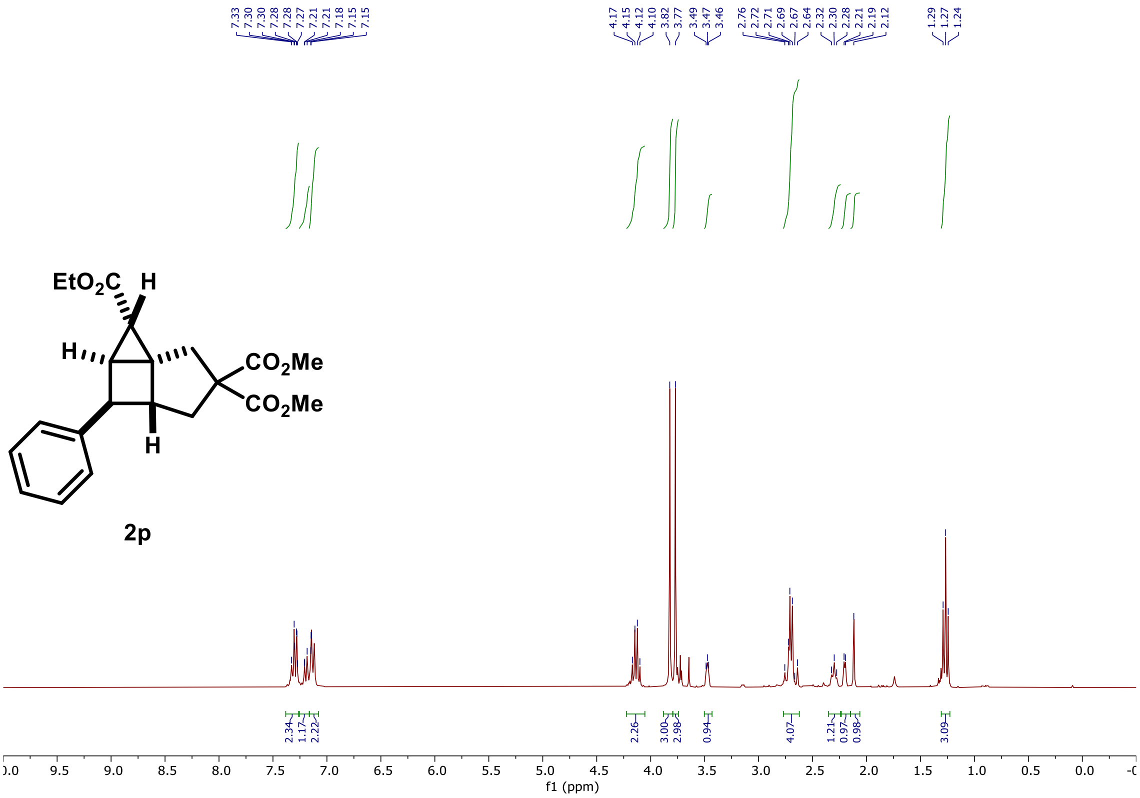

^{13}C-NMR of compound 2p (75 MHz, $CDCl_3$)

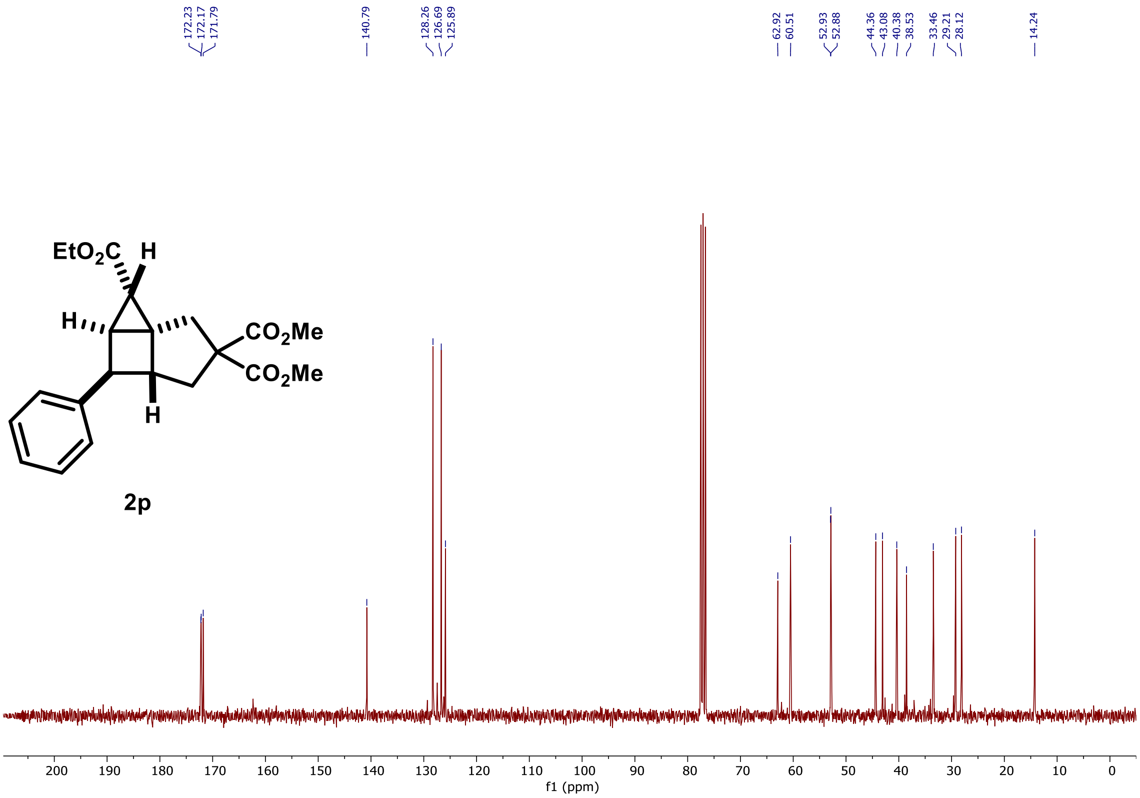

Atom label	**H** (ppm, Hz)	**C** (ppm)
1		38.5
2	2.12 (s, 1H)	29.2
3	2.20 (d, *J* = 4.4 Hz, 1H)	28.1
4	3.47 (bs, 1H)	43.1
5	2.24 – 2.37 (m, 1H)	44.4
6 / 8	2.60 – 2.80 (m, 4H)	33.5, 40.4
7		62.9
9	1.14 (s, 3H)	140.8
10 – 14	7.08 – 7.15 (m, 2H), 7.20 (d, *J* = 7.8 Hz, 1H), 7.26 – 7.35 (m, 2H)	125.9, 126.7, 128.3

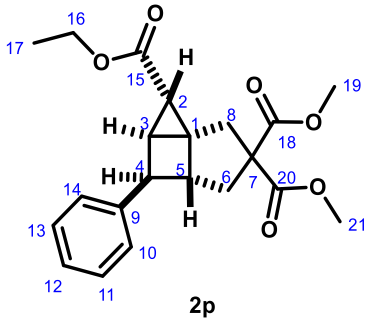

2p

^{1}H-^{13}C HSQC of compound 2p (CDCl$_3$)

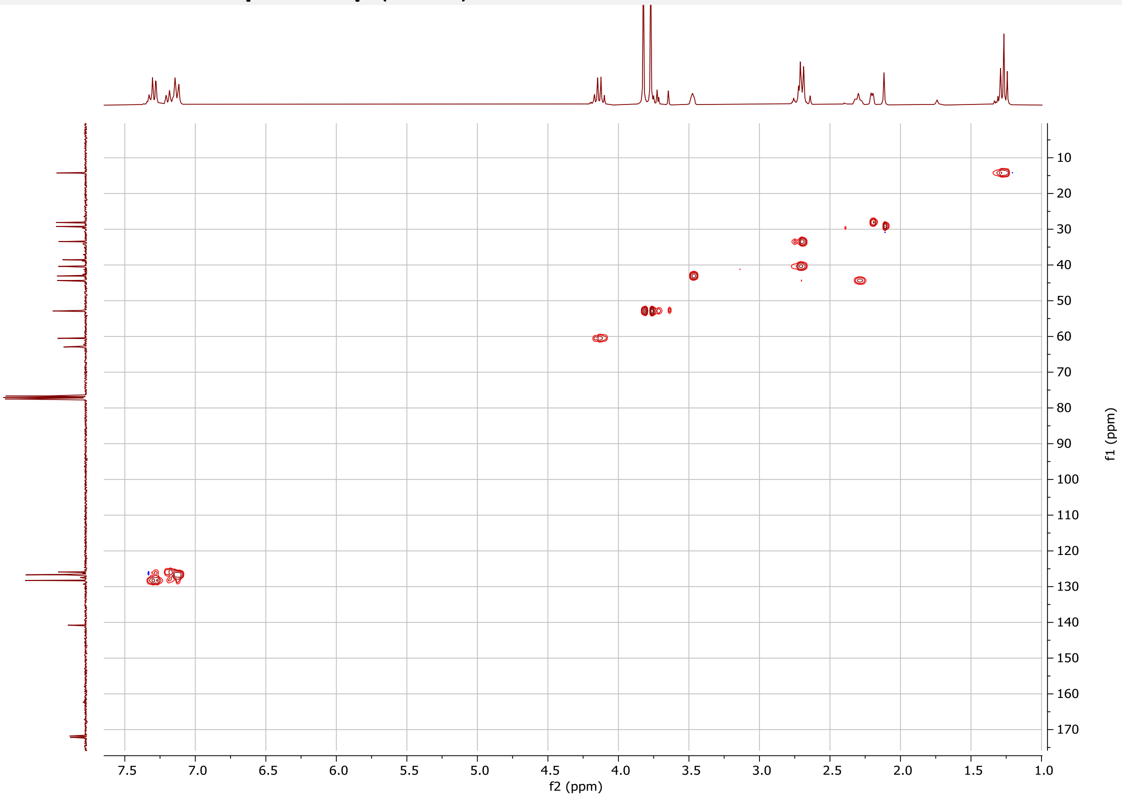

^{1}H-^{1}H **COSY of compound 2p** (CDCl$_3$, only aliphatic region is shown)

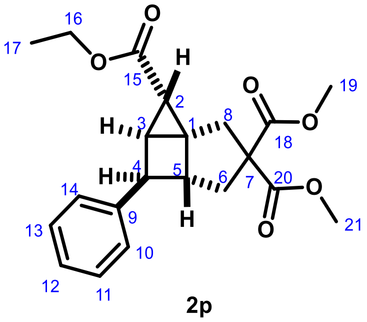

2p

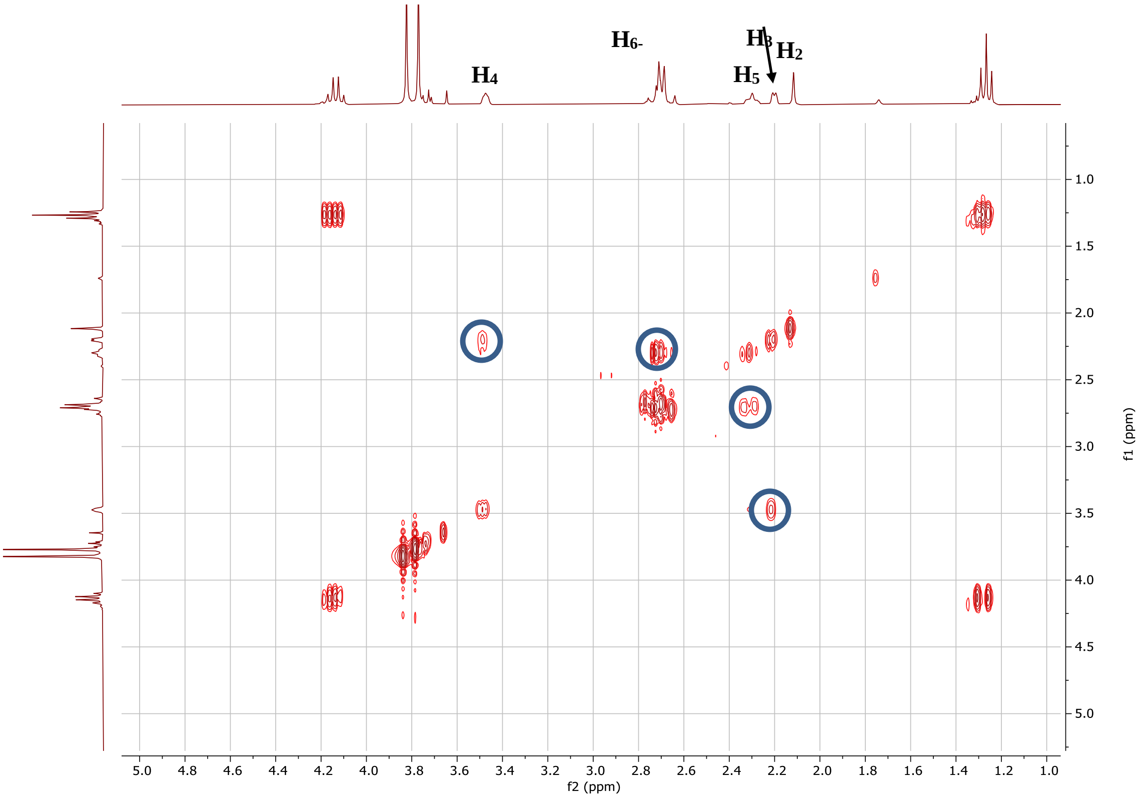

Relevant signals (COSY): **$^3J_{H,H}$ (H$_3$-H$_4$)**, **$^3J_{H,H}$ (H$_5$-H$_{6,8}$)**.

2D NOESY of compound 2p (CDCl$_3$)

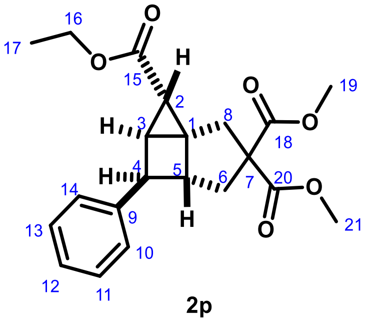

2p

C

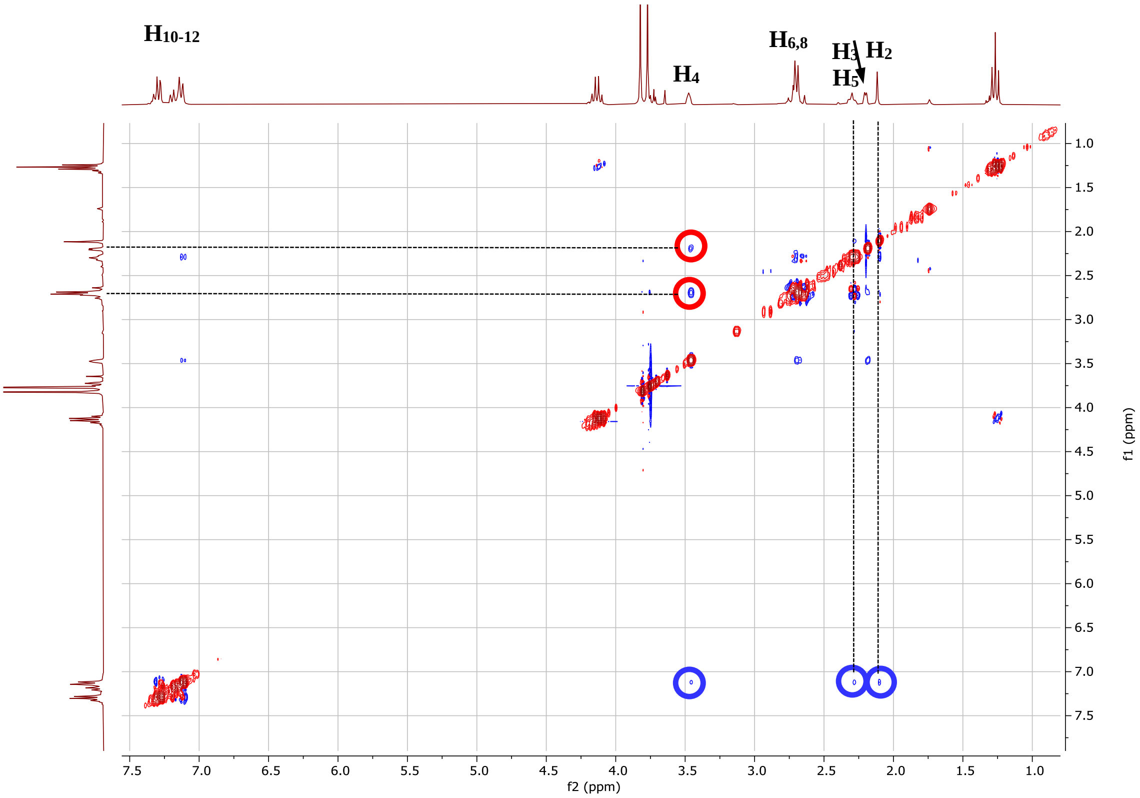

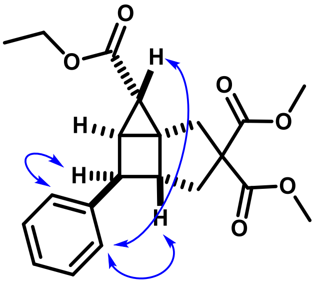

2p

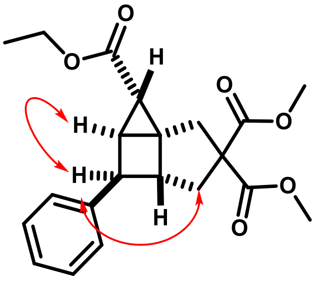

2p

^{1}H-NMR of compound 5 (300 MHz, $CDCl_3$)

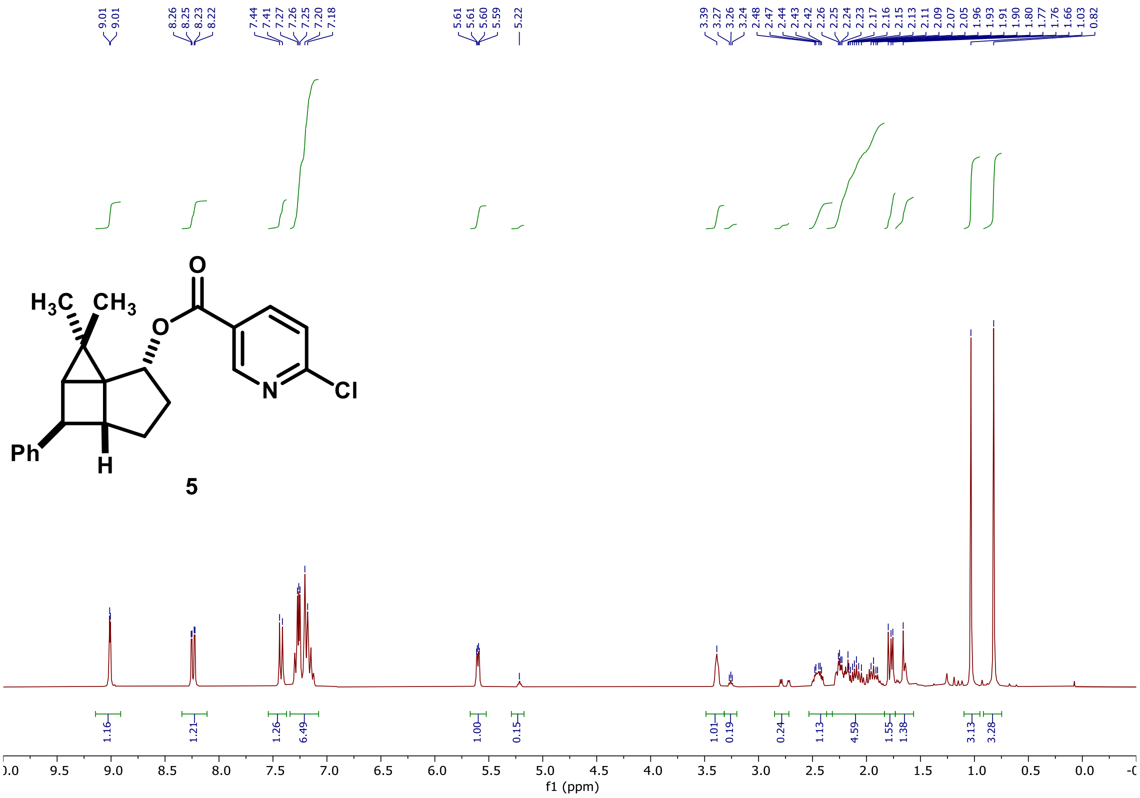

^{13}C-NMR of compound 5 (75 MHz, $CDCl_3$)

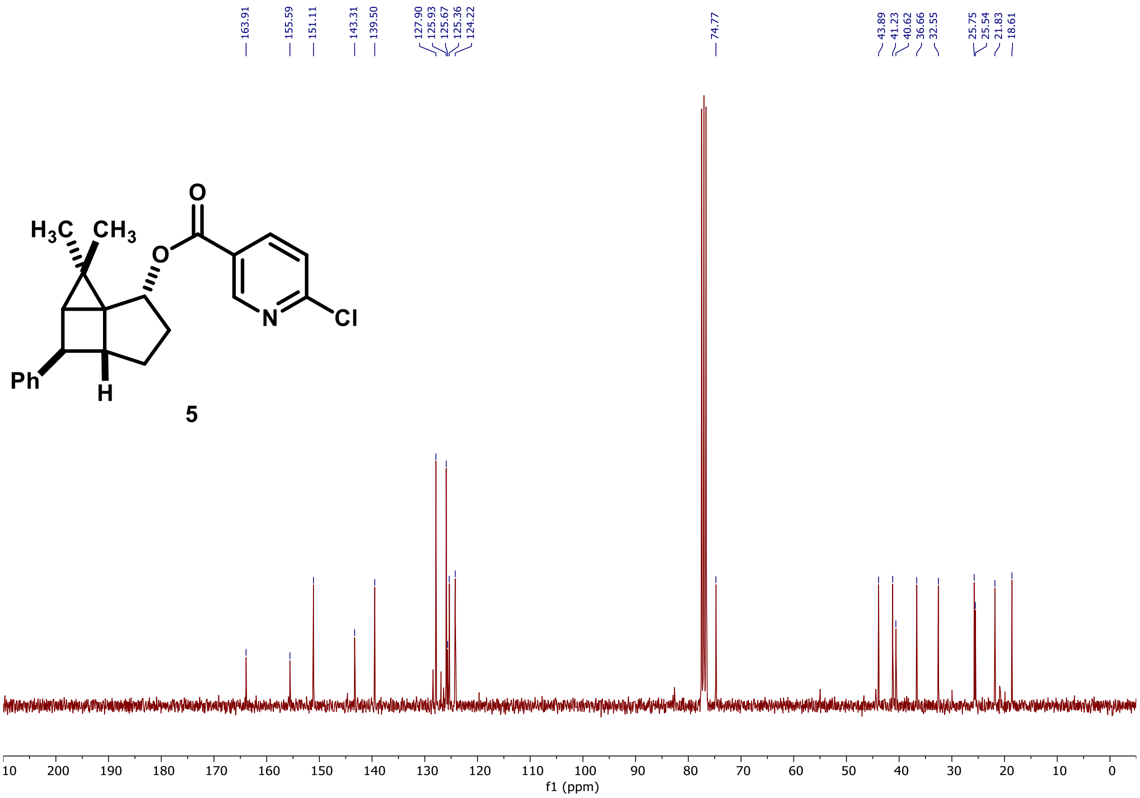

^{1}H-NMR of compound 6 (300 MHz, $CDCl_3$)

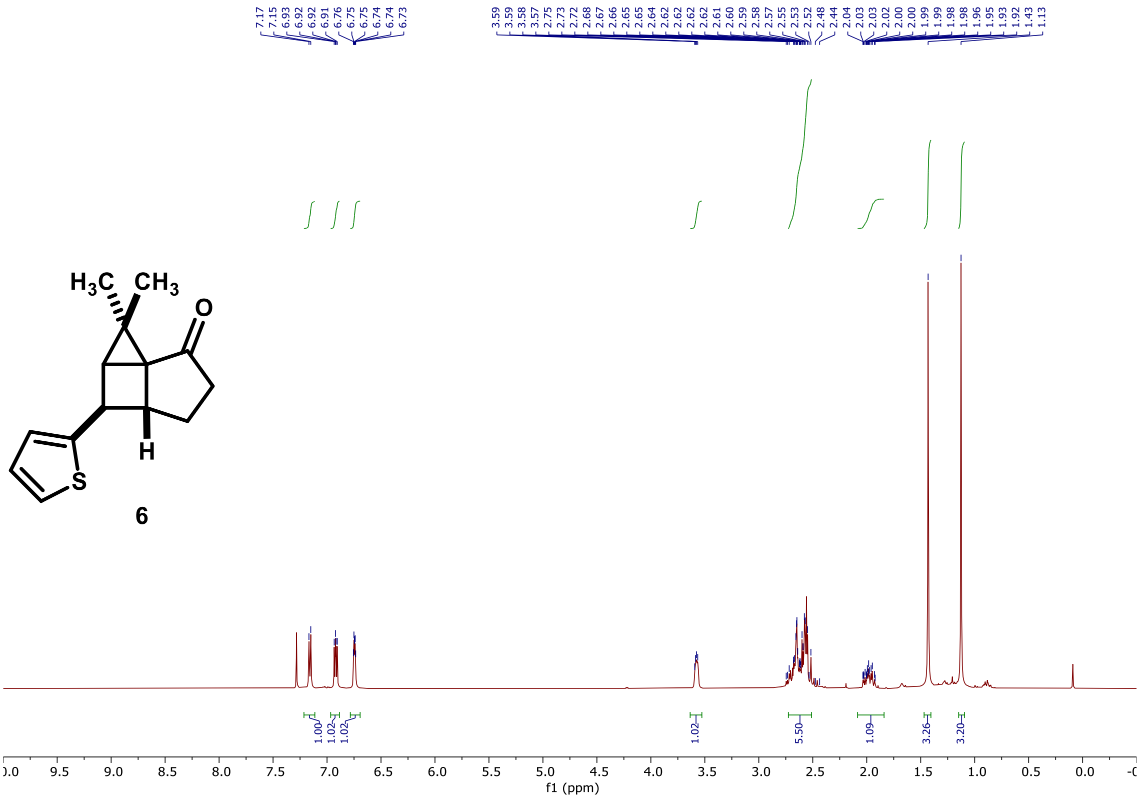

^{13}C-NMR of compound 6 (75 MHz, $CDCl_3$)

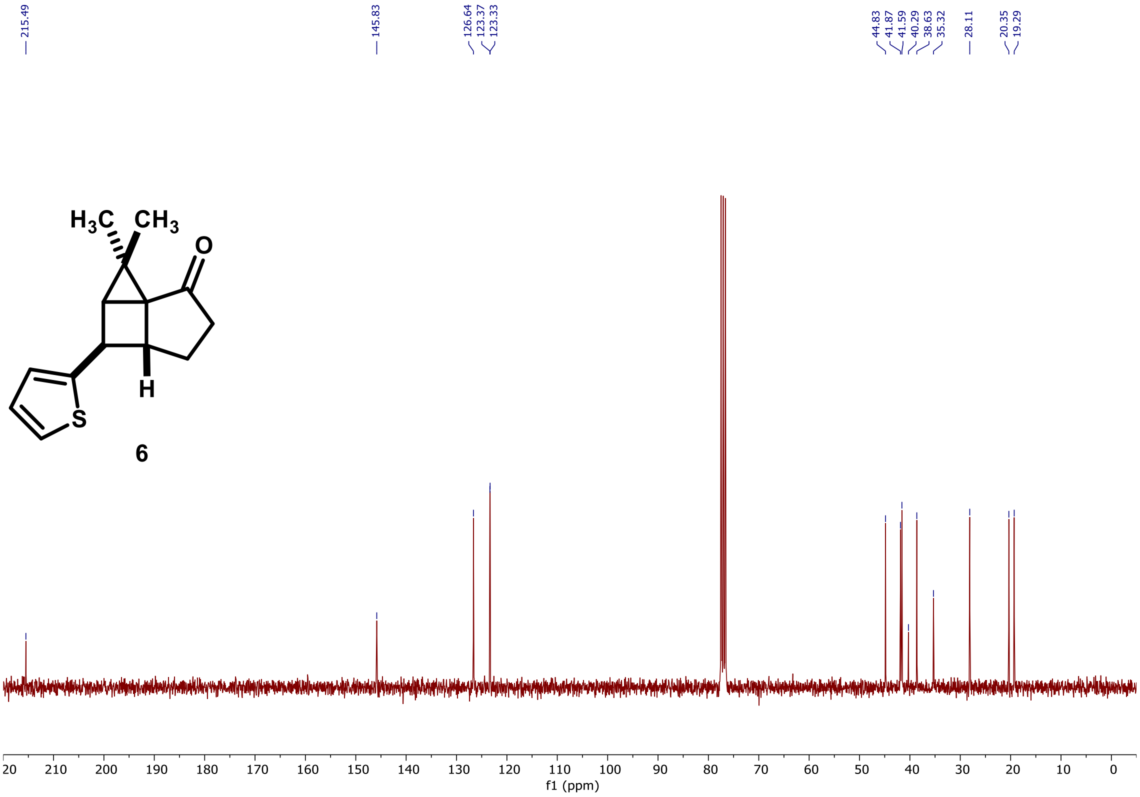

^{1}H NMR of compound S2f (400 MHz, $CDCl_3$)

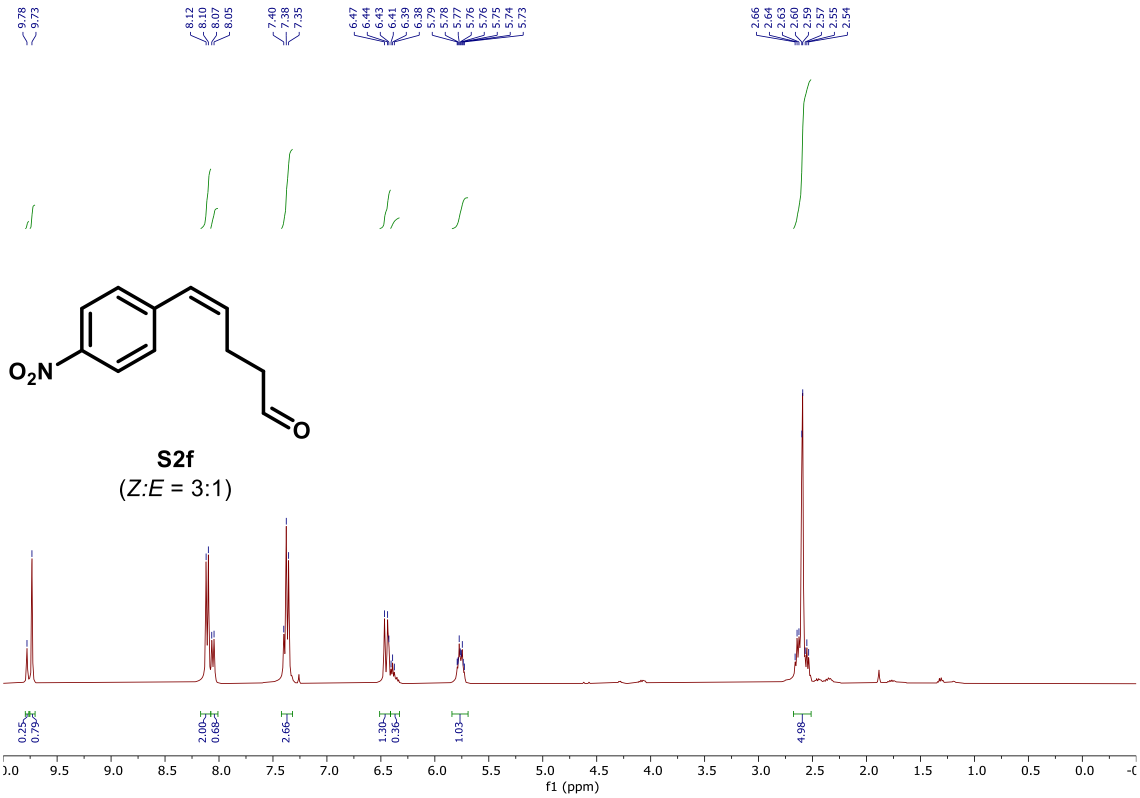

^{13}C NMR of compound 2Sf (101 MHz, $CDCl_3$)

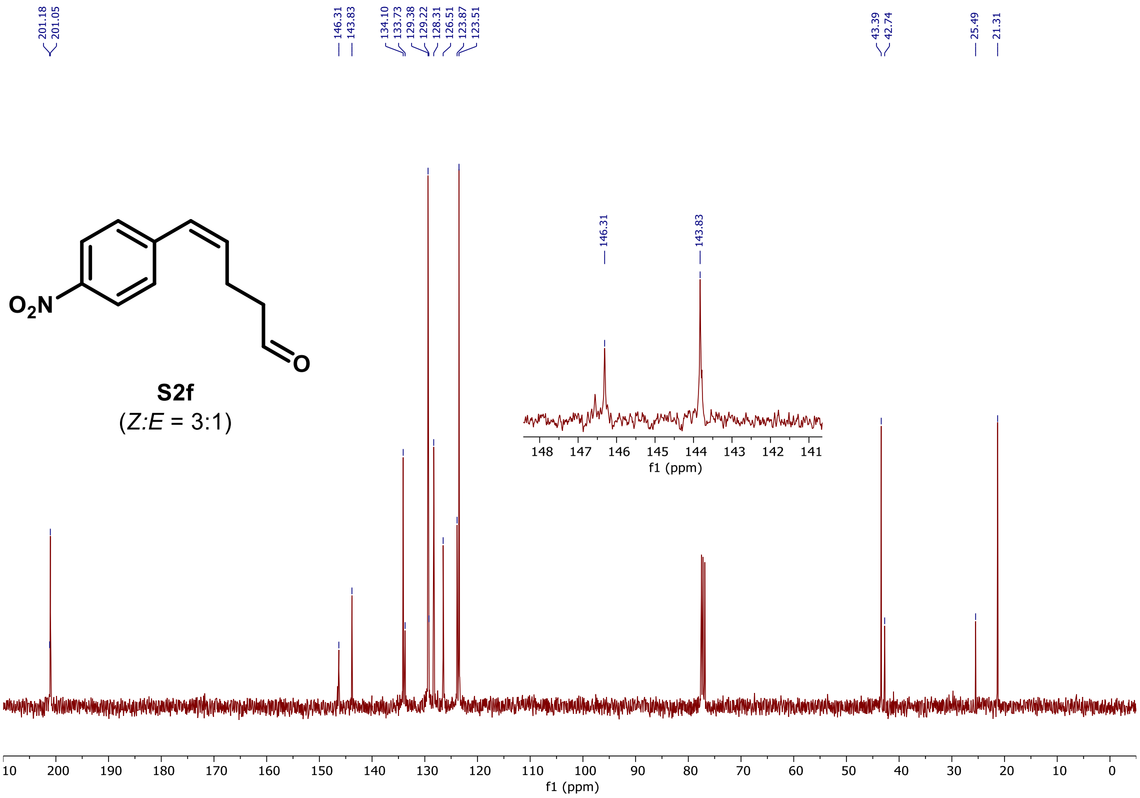

^{1}H NMR of compound S2j (300 MHz, $CDCl_3$)

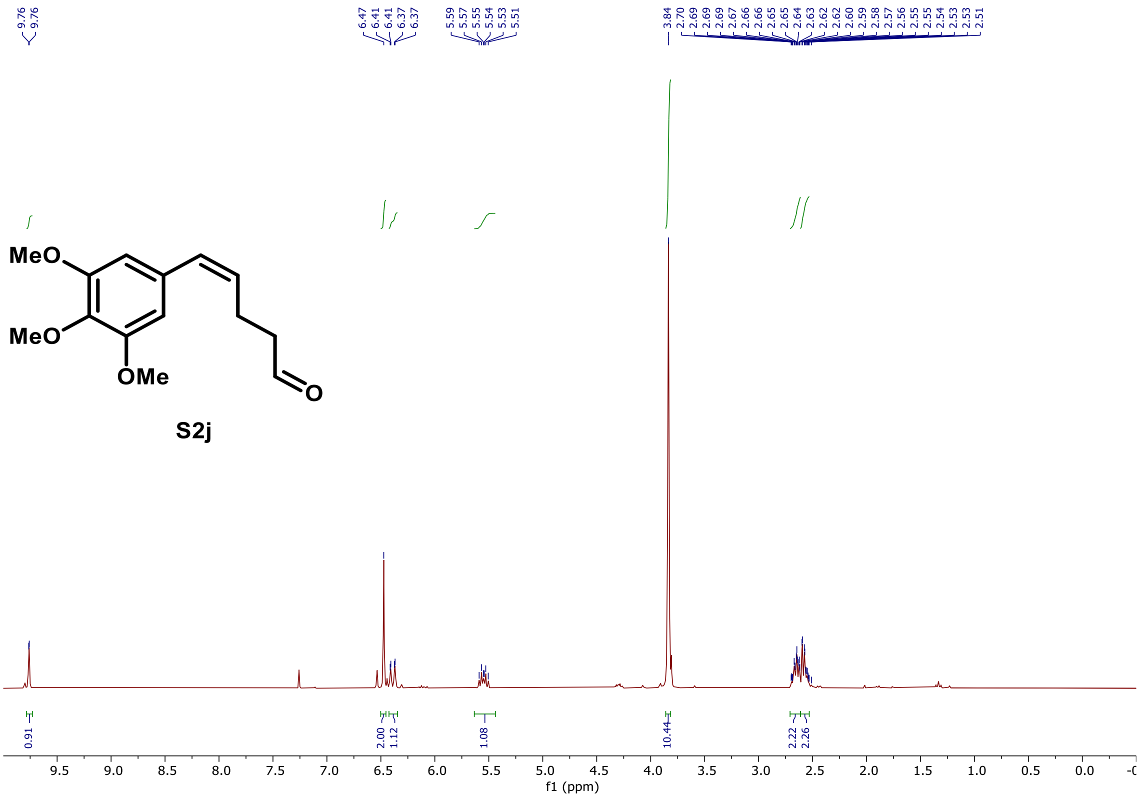

^{13}C NMR of compound S2j (75 MHz, $CDCl_3$)

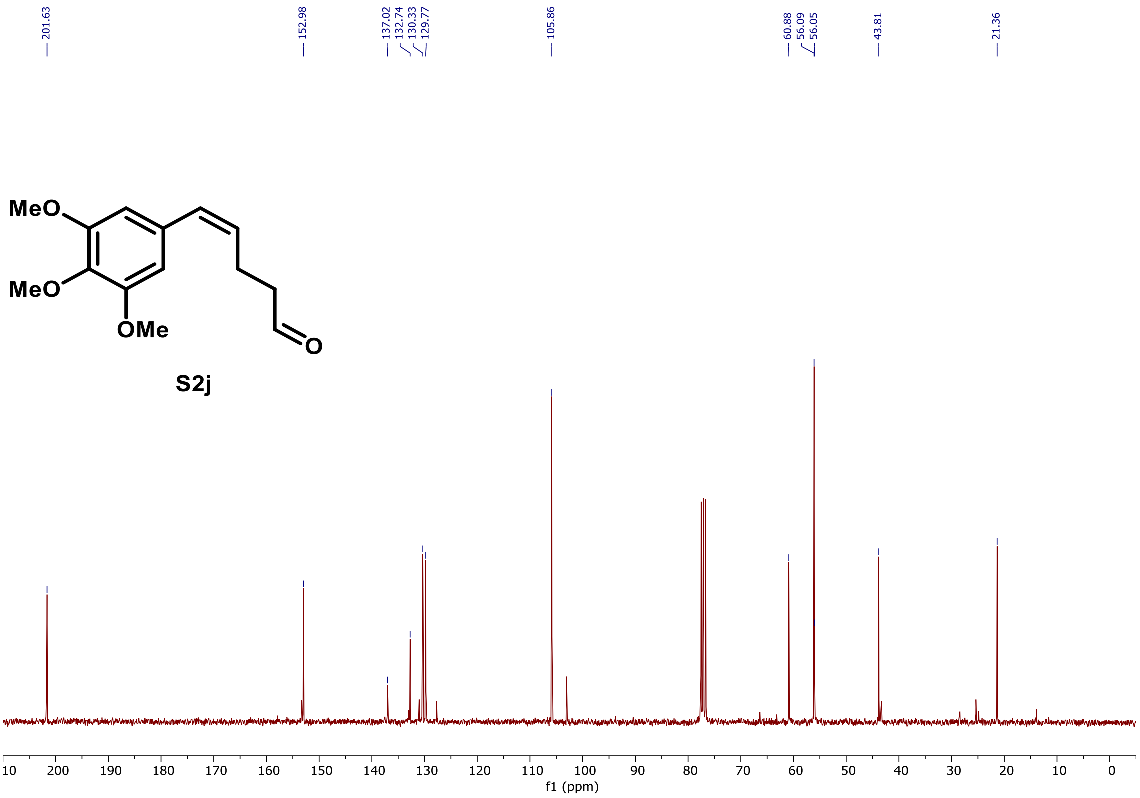

^{1}H NMR of compound S2k (300 MHz, $CDCl_3$)

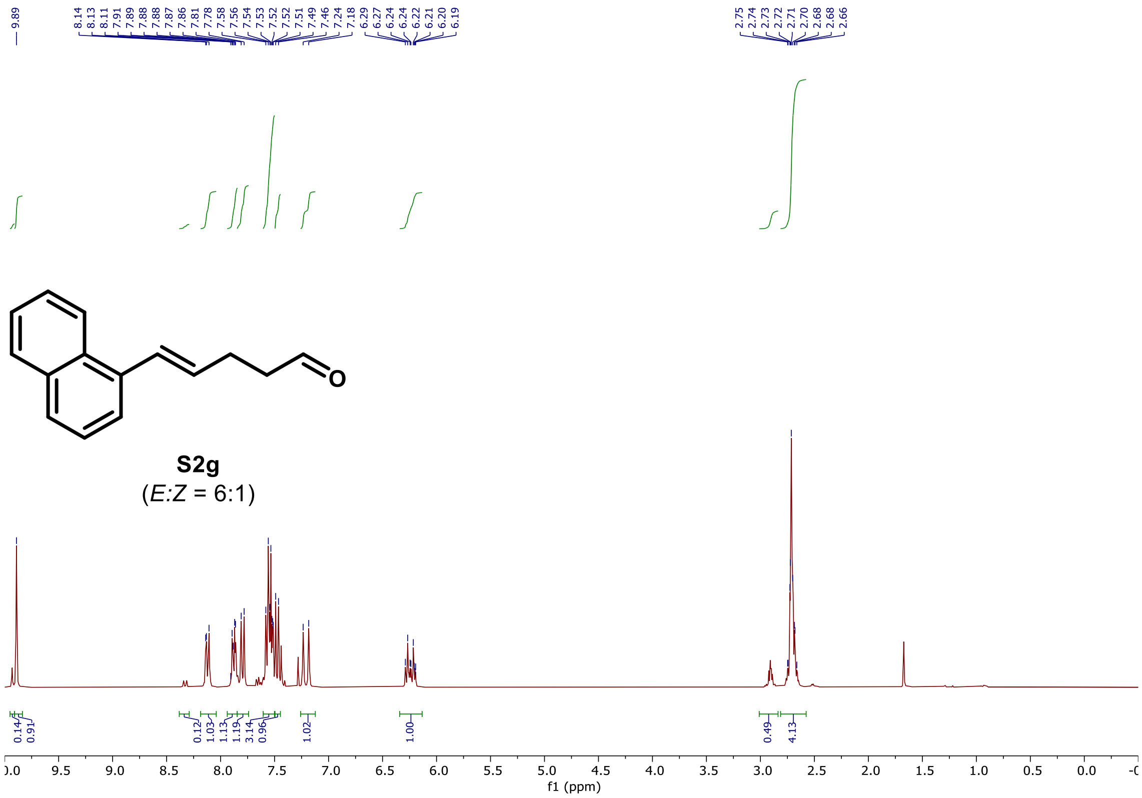

^{13}C NMR of compound S2k (75 MHz, $CDCl_3$)

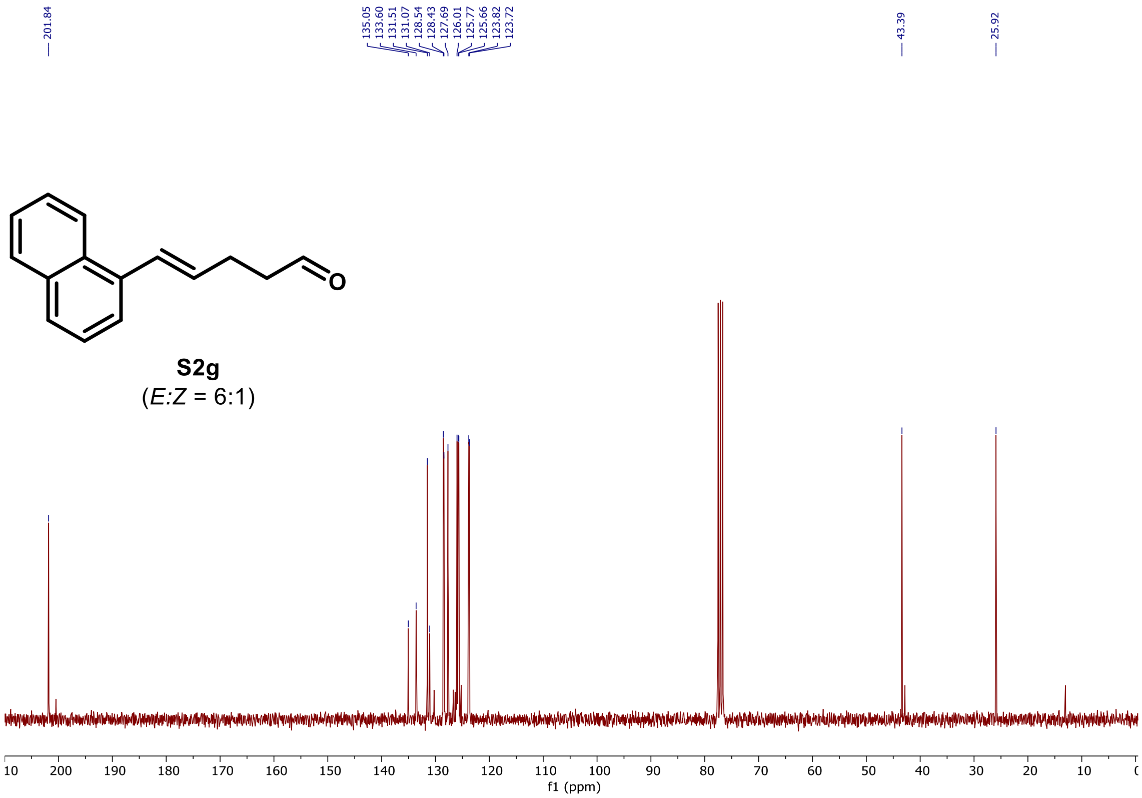

^{1}H NMR of compound S2o(400 MHz, $CDCl_3$)

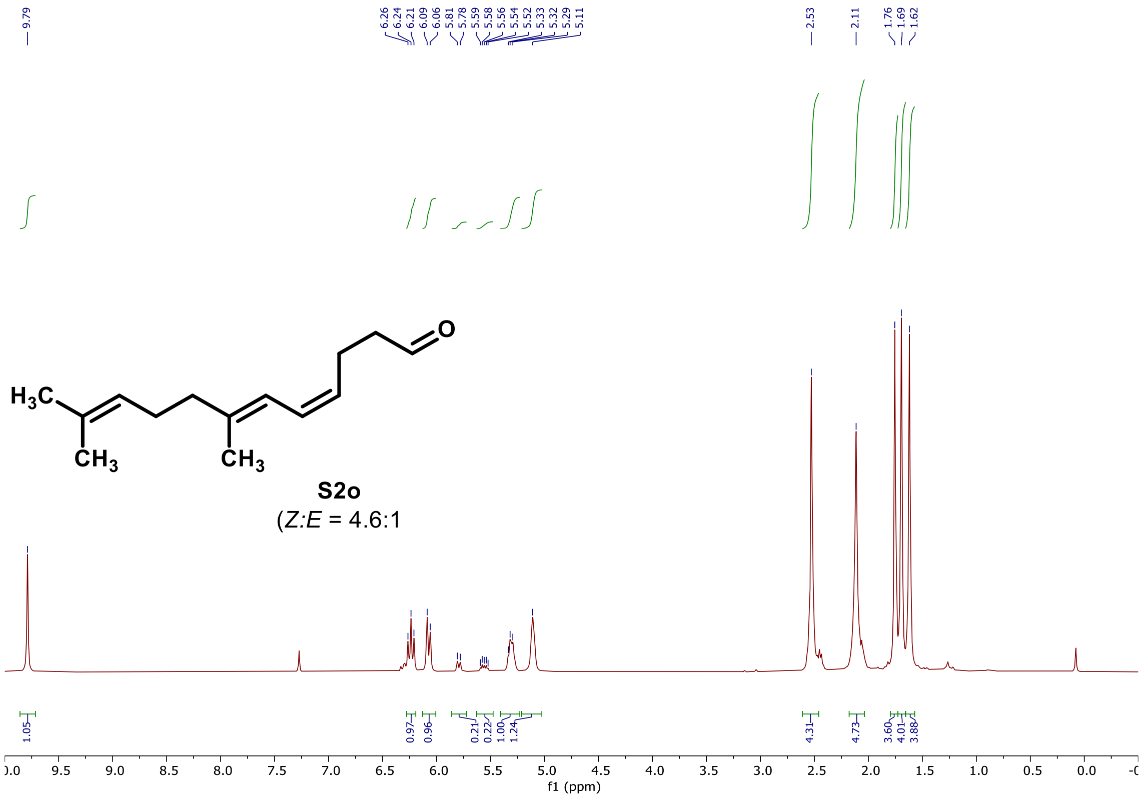

^{13}C NMR of compound S2o (101 MHz, $CDCl_3$)

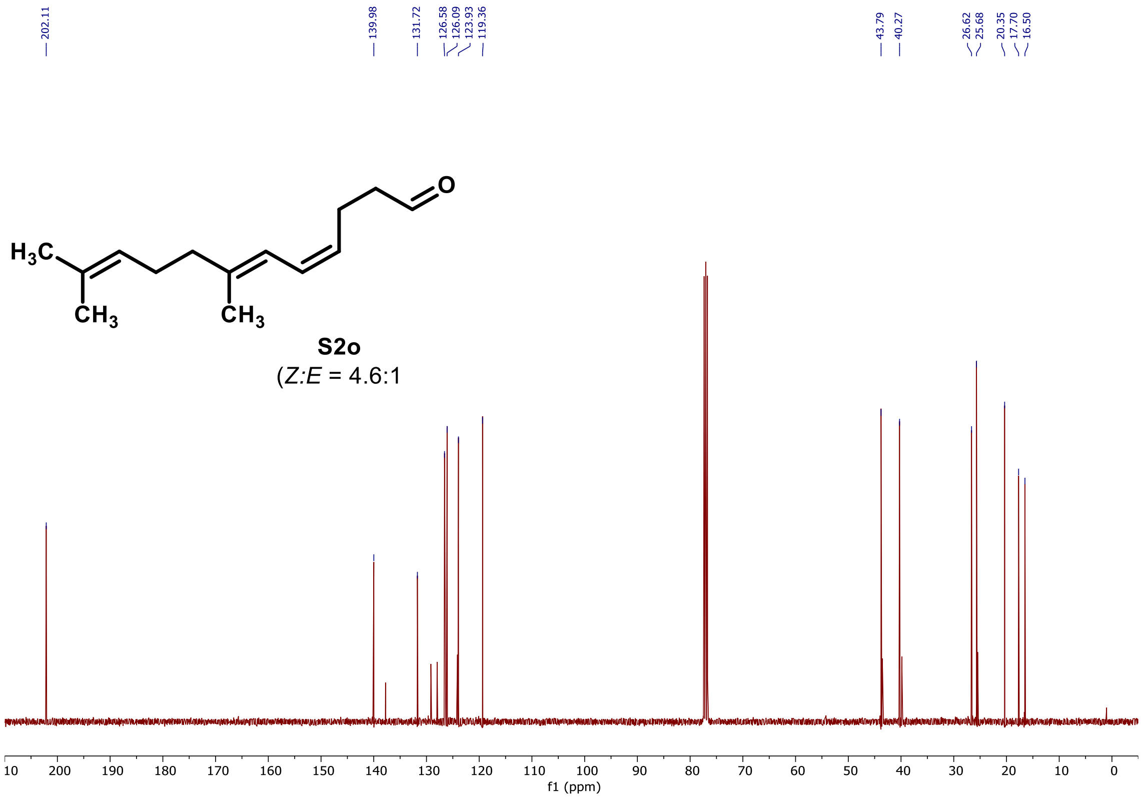

Supplement: Supplementary file 1 [file ol5c00468_si_001.pdf]
